# The Varied Frustrated Lewis Pair Reactivity of the Germylene Phosphaketene (CH{(CMe)(2,6‐^*i*^Pr_2_C_6_H_3_N)}_2_)GePCO

**DOI:** 10.1002/chem.202200666

**Published:** 2022-03-24

**Authors:** Yile Wu, Zhao Zhao, Ting Chen, Jingjie Tan, Zheng‐Wang Qu, Stefan Grimme, Yufen Zhao, Douglas W. Stephan

**Affiliations:** ^1^ Institute of Drug Discovery Technology Ningbo University Ningbo 315211 Zhejiang P. R. China; ^2^ Mulliken Center for Theoretical Chemistry University of Bonn Beringstr. 4 53115 Bonn Germany; ^3^ State Key Laboratory of Elemento-Organic Chemistry Nankai University 30071 Tianjin P. R. China; ^4^ Department of Chemistry Xiamen University Xiamen 361005 Fujian P. R. China; ^5^ Department of Chemistry University of Toronto 80 St. George St Toronto ON M5S3H6 Canada

**Keywords:** alkyne, DFT calculations, frustrated Lewis pairs, germylene, olefin

## Abstract

The germylene species (CH{(CMe)(2,6‐*i*Pr_2_C_6_H_3_N)}_2_)GePCO **1** is shown to react with the Lewis acids (E(C_6_F_5_)_3_ E=B, Al). Nonetheless, **1** participates in FLP chemistry with electron deficient alkynes or olefins, acting as an intramolecular FLP. In contrast, in the presence of B(C_6_F_5_)_3_ and an electron rich alkyne, **1** behaves as Ge‐based nucleophile to effect intermolecular FLP addition to the alkyne. This reactivity demonstrates that the reaction pathway is controlled by the nature of the electrophile and nucleophile generated in solution, as revealed by extensive DFT calculations.

## Graphical abstract

**The germylene species** (CH{(CMe)(2,6‐*i*Pr_2_C_6_H_3_N)}_2_)GePCO reacts with E(C_6_F_5_)_3_ (E=B, Al) and with alkynes or olefins, acting as either an intramolecular or intermolecular FLP controlled by the nature of electrophiles and nucleophiles generated in solution as revealed by extensive DFT calculations.

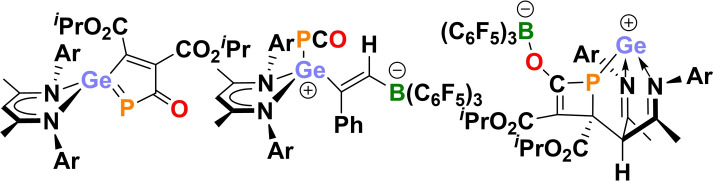

The emergence of frustrated Lewis pairs (FLPs) from the reactivity derived from combinations of sterically encumbered Lewis acids and bases has prompted broad interest.^[1]^ Such systems have been shown to effectively activate a wide variety of small molecules including H_2_, CO, CO_2_, NO and SO_2_ among others, providing new avenues to catalysis and synthetic chemistry. Among the FLP systems that have been described, a variety of Lewis bases including a wide range of P, N and carbene donors have been employed. On the other hand, the range of Lewis acids has mostly been limited to electron‐deficient boranes or alanes, although carbon‐based cationic Lewis acids have received lesser attention. Among these latter cases, trityl,^[2]^ benzhydrylium (**I**),^[3]^ *N*‐methylacridinium (**II**),[^3^, ^4^] trioxatriangulenium (**III**)^[5]^ and a Ru‐η^6^‐arene salt (**IV**),^[6]^ have been served as the Lewis acid in FLP reactivity. Meanwhile, Bazan et al.^[7]^ and Alcarazo^[8]^ have demonstrated that fullerene acts as a neutral carbon‐based Lewis acid while Alcarazo et al.^[9]^ have also exploited electron‐deficient allenes (**V**) as carbon‐based Lewis acid in FLP chemistry (Figure 1).

**Figure 1:**
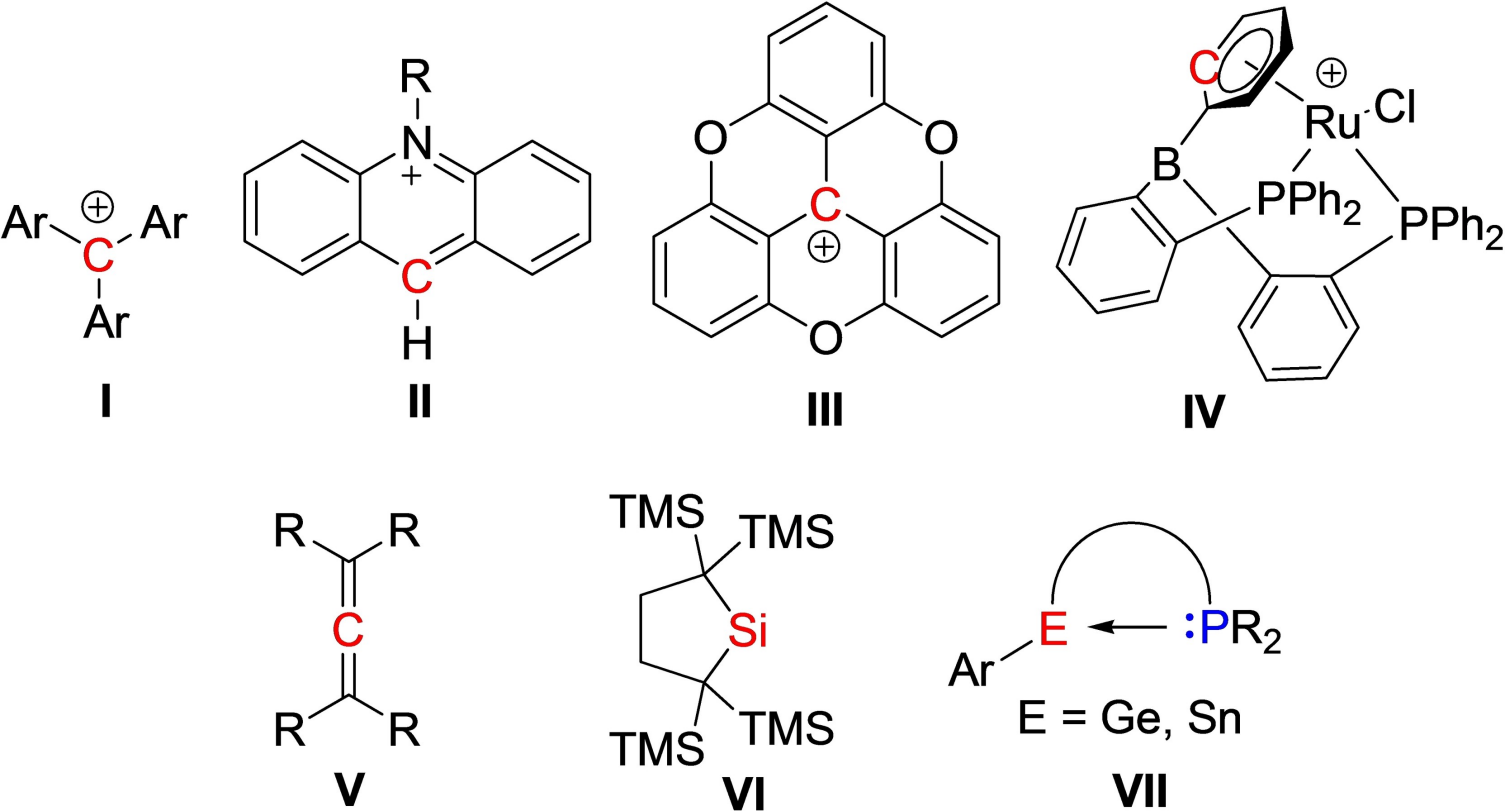
Examples of group‐14‐element‐based Lewis acids for FLP chemistry.

The ambiphilic nature of carbenes has also been exploited for FLP chemistry. The report of the activation of H_2_ by cyclo‐aminoalkyl carbenes (CAACs) by Bertrand^[10]^ can be viewed as FLP chemistry at a single site as the carbene carbon is both an electron donor and an acceptor. Among the heavier analogs, Müller and coworkers^[11]^ have shown that the combination of silylenes (**VI**) (Figure 1) with either Lewis acids or bases results in intermolecular FLPs capable of activating H_2_, further emphasizing the ambiphilic character. On the other hand, while the use of Ge(II), Sn(II) and Pb(II) centers have been exploited as Lewis acids in intramolecular FLP systems with P, N donors (**VII**) (Figure 1) to activate alkynes and alkenes,[^12^, ^13^] the use of such heavier cognates as Lewis bases has drawn lesser attention.^[14]^

In the last decade, Grützmacher and Goicoechea^[15]^ have pioneered the chemistry of 2‐phosphaethynolate anion (PCO^−^), the phosphorus analogue of the cyanate anion. This anion has proved to be very useful building block for phosphorus‐containing heterocycles.^[16]^ In addition, Grützmacher, Li and co‐workers demonstrated the electrophilic character of the carbon center in (R_2_N)_2_P−PCO and Ph_3_Si−PCO, as these species react with Lewis basic N‐heterocyclic carbenes affording carbene−phosphinidene adducts^[17]^ and carbene−phosphakene adducts.^[18]^ Furthermore, phosphaketene phosphine (R_2_N)_2_P−PCO and phosphaketene germylenes LGe−PCO have been shown to form phosphanyl supported phosphinidene^[19]^ and 1,3‐digerma‐2,4‐diphosphacyclobutadiene^[20]^ via the elimination of CO under UV photolysis, respectively. Grützmacher et al. also reported the construction of optoelectronic material precursors 1,3,4‐azadiphospholides from NaPCO, in which the nitrogen‐containing substrate acting as a nucleophile to attack the electrophilic carbon center of PCO.^[21]^ Recently, we have exploited Ph_3_GePCO as a versatile precursor for the facile synthesis of the phosphorus analogue of DMF, Me_2_PC(H)O,^[22]^ diphospha‐ureas,^[23]^ phosphaalkenes,^[24]^ and acylphosphines.^[25]^

Herein, we explore the chemistry of the related germylene species L^1^GePCO (**1**, L^1^=CH{(CMe)‐(2,6‐^*i*^Pr_2_C_6_H_3_N)}_2_). We demonstrate that this species exhibits varying FLP reactivity in combinations with Lewis acids and/or alkyne and olefin substrates. These findings are illuminated via state‐of‐the‐art DFT calculations.

The phosphaketene substituted germylene **1** was readily prepared from the corresponding [L^1^GeCl] compound and NaPCO.[^20b^, ^26^] This neutral derivative of the Ge(II) cation [Ge(Dipp_2_nacnac)]^+^ reported by Power and co‐workers in 2001^[27]^ incorporates nucleophilic P and O centers, an electrophilic C center and an ambiphilic Ge. Thus, a variety of chemo‐ and regioselectivity is anticipated in the reactivity of **1**. DFT calculations at the TPSS‐D3/def2‐TZVP+COSMO(toluene) level showed that the highest occupied (HOMO) and the lowest unoccupied (LUMO) molecular orbitals of **1** mainly consist of the Ge and P electron lone pairs and the delocalized π* anti‐bond of the Nacnac ligand backbone ring, respectively, with the HOMO‐LUMO gap being 2.3 eV (see Supporting Information). Since the HOMO is 0.55 eV above other occupied molecular orbitals, the Ge and P centers are expected to dominate the nucleophilic reactivity of **1**.

To probe this basicity of **1**, we began examining reactions with Lewis acids. The reaction of **1** with B(C_6_F_5_)_3_ was performed in toluene at −30 °C (Scheme 1). Upon mixing, the solution became violet and afforded violet crystals of **2** on standing. ^31^P NMR data showed resonances at 215.5, 125.6 ppm, while the ^11^B and ^19^F NMR spectra showed peaks at −7.5, −12.2 and −128.2, −132.0, −158.2, −159.4, −161.9, −164.2, −165.2, −165.9 ppm respectively. X‐ray crystallographic characterization revealed the formulation of **2** as the salt, [L^1^Ge]_2_[(C_6_F_5_)_3_B((C_6_F_5_)_3_BOCP)_2_] (Scheme 1, Figure 2). While the cations are planar L^1^Ge(II) units, the anion is derived from two PCOB fragments forming a four‐membered C_2_P_2_ ring, with two exocyclic OB and one exocyclic BP linkages. The observed C−P (1.8013(18), 1.7923(18), 1.8400(18), and 1.8315(18) Å), P−B (2.078(2) Å) and B−O (1.541(2) and 1.555(2) Å) bond lengths are similar to those previously seen in the anion derived from the reaction of NaPCO and B(C_6_F_5_)_3_.^[28]^

**Scheme 1:**
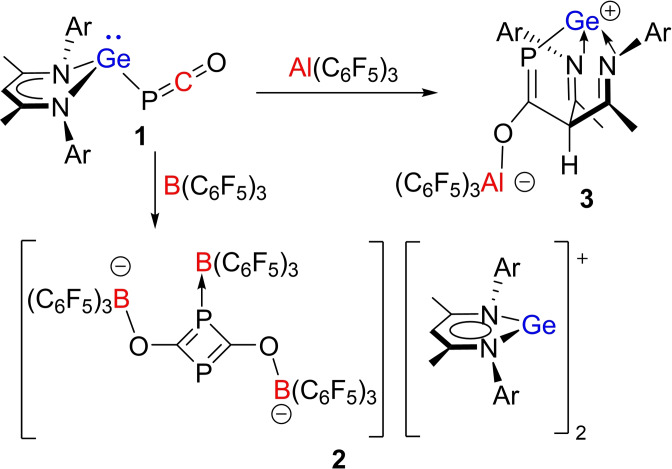
Synthesis of **2** and **3**. Ar=2,6‐*i*Pr_2_C_6_H_3_.

**Figure 2:**
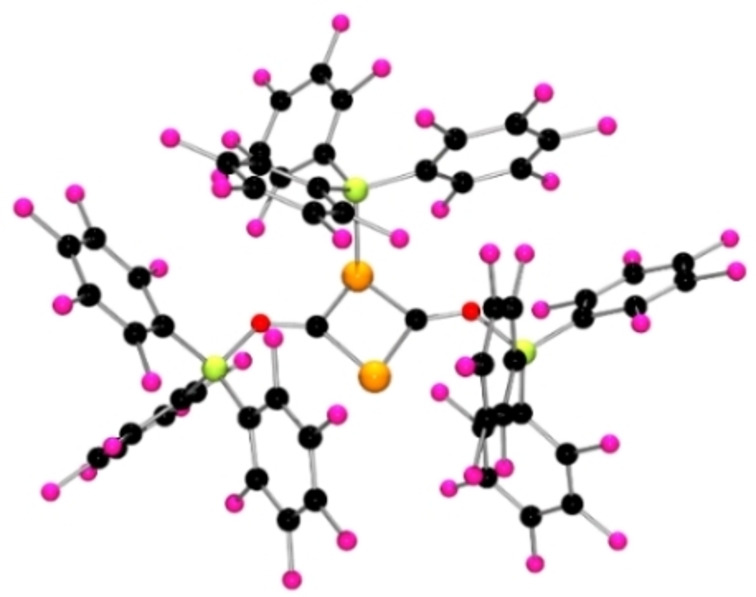
POV‐ray depiction of the molecular structure of the anion of **2**. Hydrogen atoms are omitted for clarity. C: black, F: hot pink, P: orange, N: blue, O: red, B: yellow‐green.

To probe the mechanism of these reactions, accurate dispersion‐corrected DFT calculations at the PW6B95‐D3/def2‐QZVP+COSMO‐RS//TPSS‐D3/def2‐TZVP+COSMO level of theory in toluene solution were performed (see Supporting Information).^[29]^ DFT calculations considering the interactions of **1** with B(C_6_F_5_)_3_ show that the binding free energies of B(C_6_F_5_)_3_ to the Ge, P, γ‐C and O sites of **1** are 5.2, −0.2, 19.5 and −1.7 kcal/mol, respectively. These data are consistent with reversible B(C_6_F_5_)_3_ binding to the phosphaketene oxygen as interactions with Ge or P centers are somewhat sterically hindered. B(C_6_F_5_)_3_ binding is expected to enhance the electrophilicity of the phosphaketene carbon and decrease the nucleophilicity of the Ge center thus weakening the Ge−P bond. Indeed, the dissociation of the PCOB adduct is only 7.4 kcal/mol endergonic generating the electrophilic [L^1^Ge]^+^ and nucleophilic [PCOB(C_6_F_5_)_3_]^−^ ions in solution. Further B(C_6_F_5_)_3_ binding to the P‐site of [PCOB(C_6_F_5_)_3_]^−^ is 3.5 kcal/mol endergonic, followed by nucleophilic addition of another [PCOB(C_6_F_5_)_3_]^−^ that is 21.6 kcal/mol more exergonic to afford the experimentally observed salt **2**.

In an analogous fashion, reaction of **1**, with 1 equivalent of Al(C_6_F_5_)_3_ in toluene at room temperature yielded colorless crystals of **3** after storage at −30 °C overnight. The molecular structure of **3** reveals that the *γ‐*carbon of the ligand backbone added to the electrophilic C of phosphaketene moiety directly upon coordination of the Al to oxygen atom (Scheme 1, Figure 3). The resulting Ge−P distance is 2.418(1) Å while the C−P distance is 1.707(6) Å suggestive of a C=P double bond. DFT calculations also show the transfer of Al(C_6_F_5_)_3_ to the phosphaketene oxygen site of **1** from Al(C_6_F_5_)_3_ ⋅ (toluene) is −12.5 kcal/mol exergonic affording the adduct **3 a** (see Supporting Information). Further ring‐closing to give a C−C bond to the *γ‐*carbon is 5.3 kcal/mol endergonic thus not favored in solution but likely favored in the solid state. The greater Lewis acidity of Al(C_6_F_5_)_3_ enhances the Lewis acidity of the CO fragment, prompting reaction with the basic *γ‐*carbon of the Nacnac ligand yielding the formation of **3**. In this case, the reaction can be viewed as a C/Al FLP addition to the CO fragment in **1**. Related 1,4‐additions involving both the *γ‐*carbon and the metal center has been seen in other systems containing magnesium, aluminium, germanium, and platinum.^[30]^

**Figure 3:**
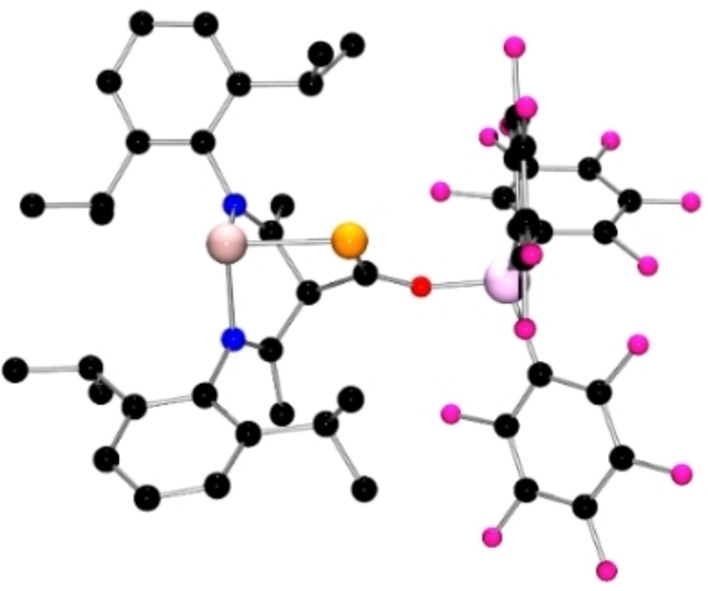
POV‐ray depiction of the molecular structure of **3**. Hydrogen atoms are omitted for clarity. C: black, F: hot pink, P: orange, N: blue, O: red, Ge: pale pink, Al: plum.

Compound **1** was also shown to react with the electron‐deficient alkyne diisopropyl but‐2‐ynedioate in toluene (Scheme 2), as slow addition led to an immediate color change from light yellow to dark purple. Workup and recrystallization from a hexane/toluene afforded purple crystals of **4**. The ^31^P NMR spectrum of **4** showed a high‐field singlet at −119.8 ppm (DFT ^31^P: −107.6 ppm). The absorption bands at 1519 cm^−1^ in the IR spectrum can be assigned to the PCO moiety (DFT 1550 cm^−1^). Single crystal XRD analysis revealed **4** (Figure 4a) to be a planar five‐membered C_3_PGe heterocycle in which the alkyne has added to the Ge and the carbonyl carbon of the PCO fragment. The newly formed Ge−C and C−C bond lengths are 1.946(5) and 1.343(6) Å, respectively. The formation of **4** amounts to the addition of the basic Ge and Lewis acidic carbonyl carbon to the alkyne, suggesting that **1** behaves as an intramolecular FLP in this case.

**Scheme 2:**
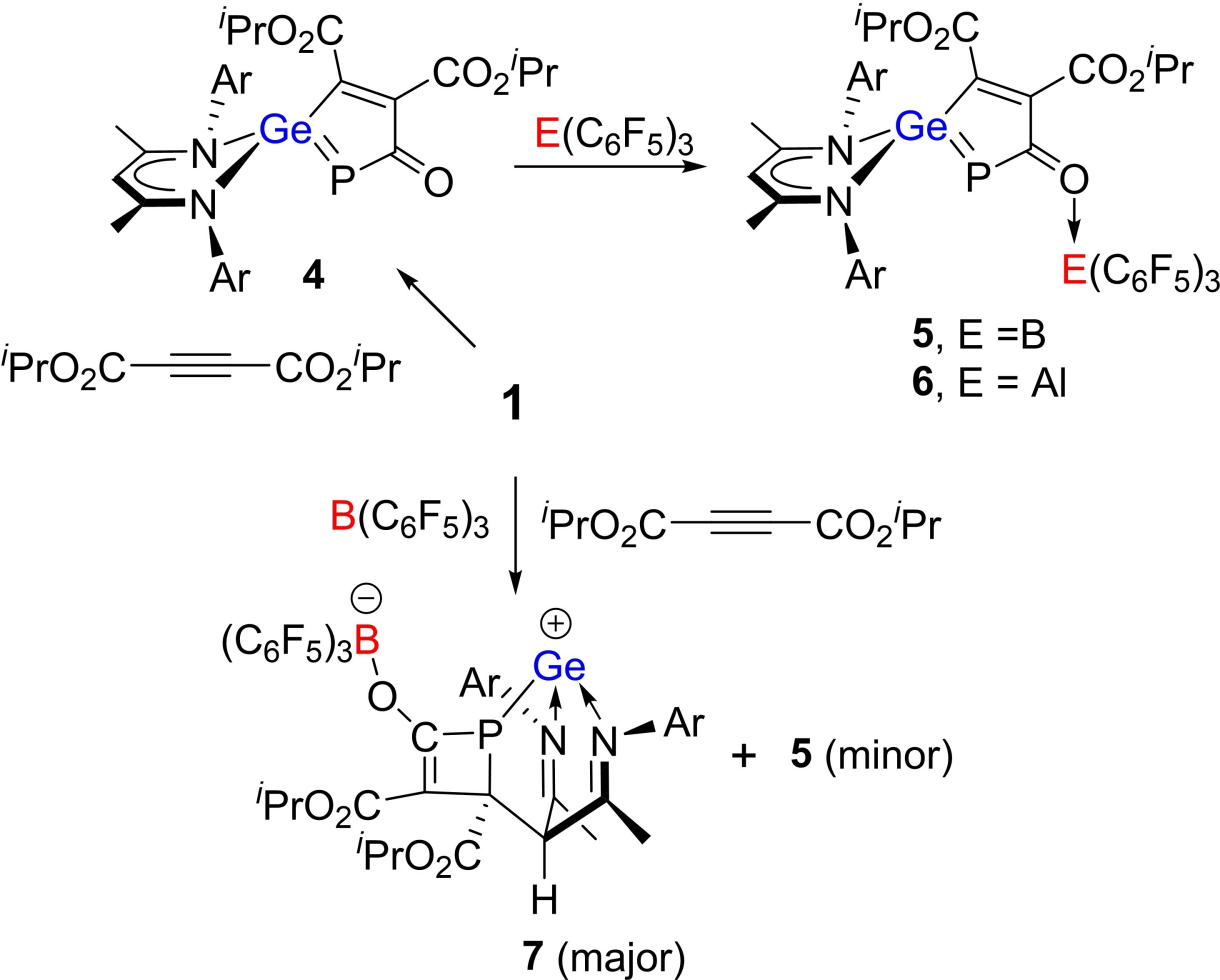
Reactions of **1**, diisopropyl‐but‐2‐ynedioate with/without B(C_6_F_5_)_3_.

**Figure 4:**
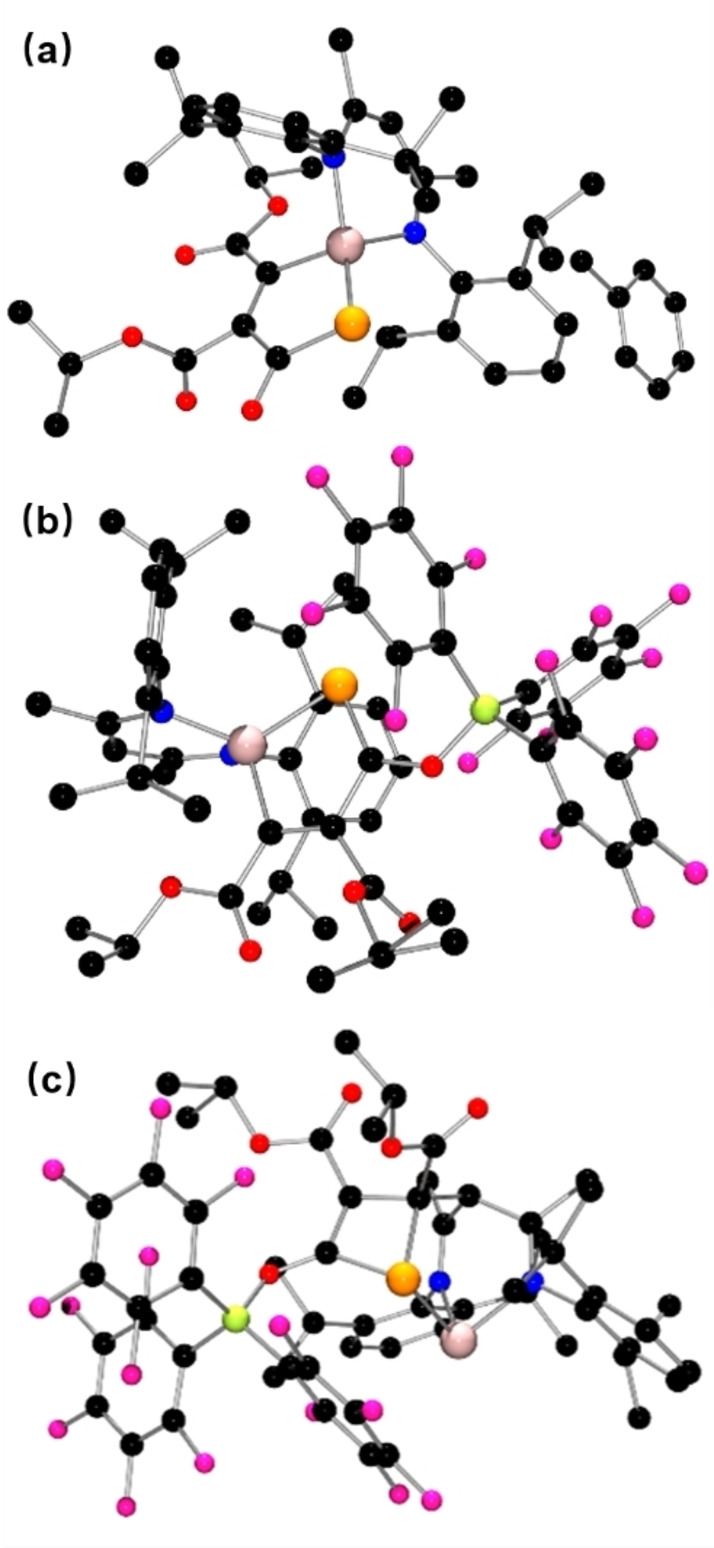
POV‐ray depiction of the molecular structure of (a) **4** (b) **5** and (c) **7**. Hydrogen atoms are omitted for clarity. C: black, P: orange, N: blue, O: red, Ge: pale pink.

Subsequent treatment of **4** with 1 equivalent of B(C_6_F_5_)_3_ in C_6_D_6_ at room temperature led to an immediate color change of the resulting solution from dark violet to bright red. With the addition of *n*‐hexane, red crystals of **5** were obtained from the solution in 71 % yield. In an analogous fashion, the adduct of **4** with Al(C_6_F_5_)_3_, **6** was also prepared, isolated and spectroscopically characterized (Scheme 2). Both products **5** (Figure 4b) and **6** (See Supporting Information) were also crystallographically characterized, revealing simple coordination of the carbonyl oxygen to B and Al, yielding the B−O and Al−O bond lengths of 1.5157(16) and 1.790(3) Å, respectively.

Interestingly, simultaneously mixing of **1**, B(C_6_F_5_)_3_ and diisopropyl‐but‐2‐ynedioate (1 : 1 : 1) in toluene proceeded at ambient temperature affording colorless crystals of **7** as the main product in 71 % yield (Scheme 2). The molecular structure of **7** was established by NMR analysis and a single‐crystal X‐ray diffraction study. The ^31^P and ^11^B NMR spectra of **7** displayed singlets at 33.4 and −1.7 ppm, respectively, (DFT ^31^P: 51.8 ^11^B: −1.8 ppm) indicating the presence of a tetracoordinated boron anionic center. In the molecular structure of **7** (Figure 4c), the *γ*‐carbon of the Nacnac ligand backbone is linked to the alkynyl carbon of the dialkyl but‐2‐ynedioate. The Ge atom is coordinated by two imine‐nitrogen atoms and one phosphorus atom and adopts a trigonal pyramidal geometry. The planar four membered C_3_P heterocycle of **7** features a C=C double bond (1.370(3) Å) and a C−C single bond (1.513(3) Å), respectively. The two C−P bond lengths of 1.847(2) Å and 1.907(2) Å in **7** are closed to that of primary acylphosphine Ph_3_PCHC(O)PH_2_ (1.858(3) Å).^[25]^ In addition to **7**, crystals of **5** were also isolated from this reaction albeit in 2 % yield.

The formation of **5** and **7** demonstrates the impact of order of reagent addition and suggests that the reaction pathway is altered by the interaction of alkyne with B(C_6_F_5_)_3_. Probing these reactions computationally revealed that in the absence of borane, nucleophilic attack of the electron‐poor alkyne *i*PrO_2_CC≡CCO_2_ *i*Pr by the Ge center of **1** is 8.3 kcal/mol endergonic over a low barrier of 11.1 kcal/mol (via **TS1**) generating the zwitterion **A** (Scheme 3). This is followed by a barrierless ring‐closing (via **TS2**) between the anionic alkynyl carbon and the electrophilic phosphaketene carbon affording five‐membered‐ring product **4**. The subsequent addition of B(C_6_F_5_)_3_ to the phosphaketene oxygen is barrierless and −19.4 kcal/mol exergonic to form the product **5**. In contrast, for the simultaneous mixture of **1**, B(C_6_F_5_)_3_ and the alkyne *i*PrO_2_CC≡CCO_2_ *i*Pr, the anion [PCOB(C_6_F_5_)_3_]^−^ may act as a nucleophile to attack the alkyne yielding a PC_3_‐ring anion which adds further via the C=P bond to the electrophilic Ge^+^ and the nucleophilic γ‐carbon centers of the FLP‐like [L^1^Ge]^+^ affording **7**.

**Scheme 3:**
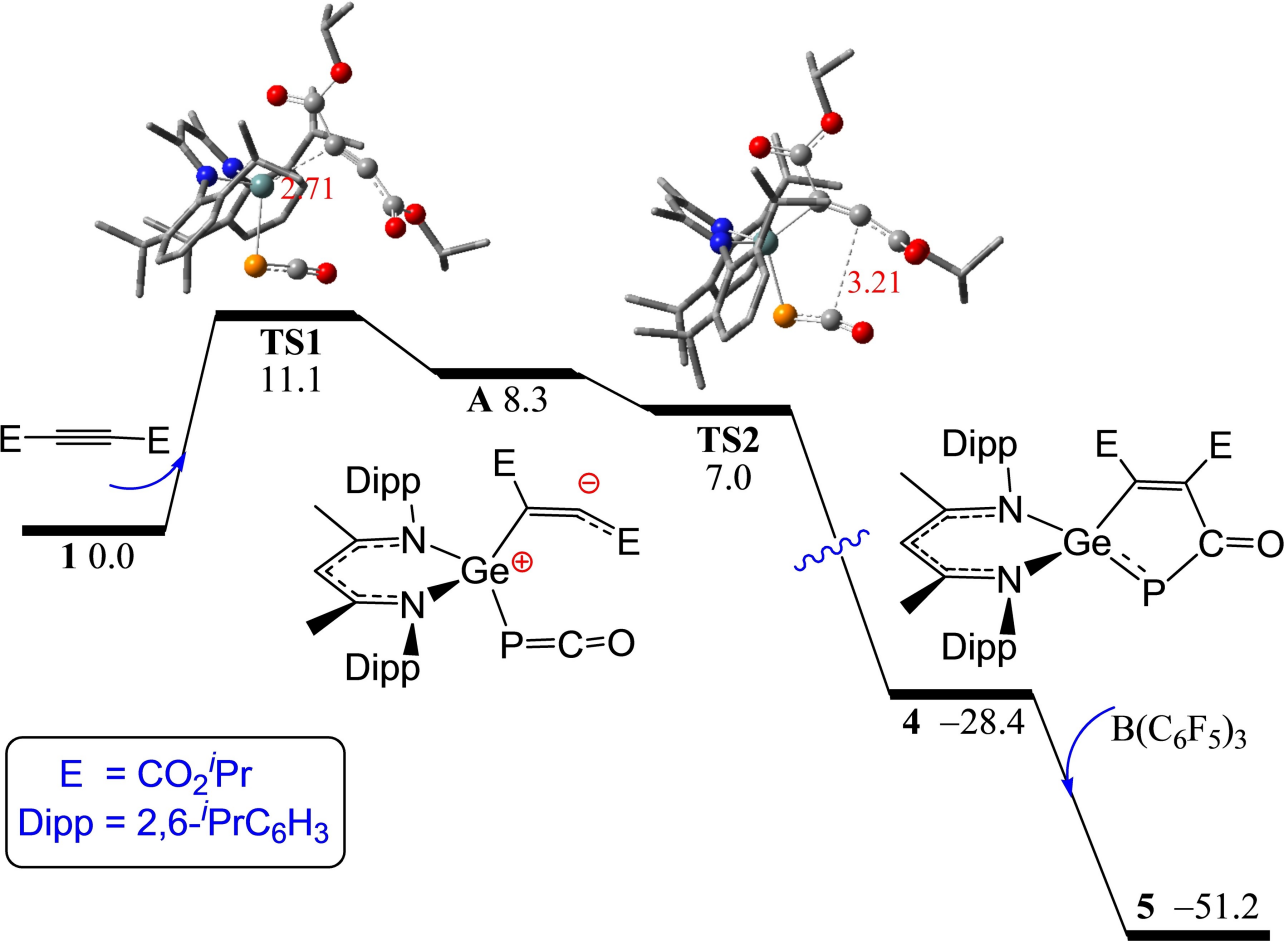
DFT‐computed free energy paths (in kcal/mol, at 298 K and 1 M) for the activation of the ^*i*^PrO_2_CC≡CCO_2_^*i*^Pr with **1**. Crucial Ge, P, C, O, N, and H atoms are highlighted in ball‐and‐stick models as teal, orange, grey, red, blue and white balls, respectively, with selected bond lengths shown in Å.

The corresponding reaction of **1**, B(C_6_F_5_)_3_ and phenylacetylene (ratio 1 : 1 : 1) in toluene, gave rise to new singlets at −304.2 ppm and −16.4 ppm in the ^31^P and ^11^B NMR spectra, respectively (DFT ^31^P: −326.7 ^11^B: −16.0 ppm). Crystallization from toluene solution afforded colorless blocks of **8** in 51 % yield. The structure of **8** was confirmed by single crystal X‐ray diffraction (Scheme 4, Figure 5a), revealing the regiospecific intermolecular *trans‐*FLP addition of germylene and B(C_6_F_5_)_3_ to the alkyne with the borane adding to the less hindered carbon. The new Ge−C and B−C bond lengths are 1.918(5) and 1.645(7) Å, respectively, yielding an olefinic linkage. At the same time, the PCO group is unchanged.

**Scheme 4:**
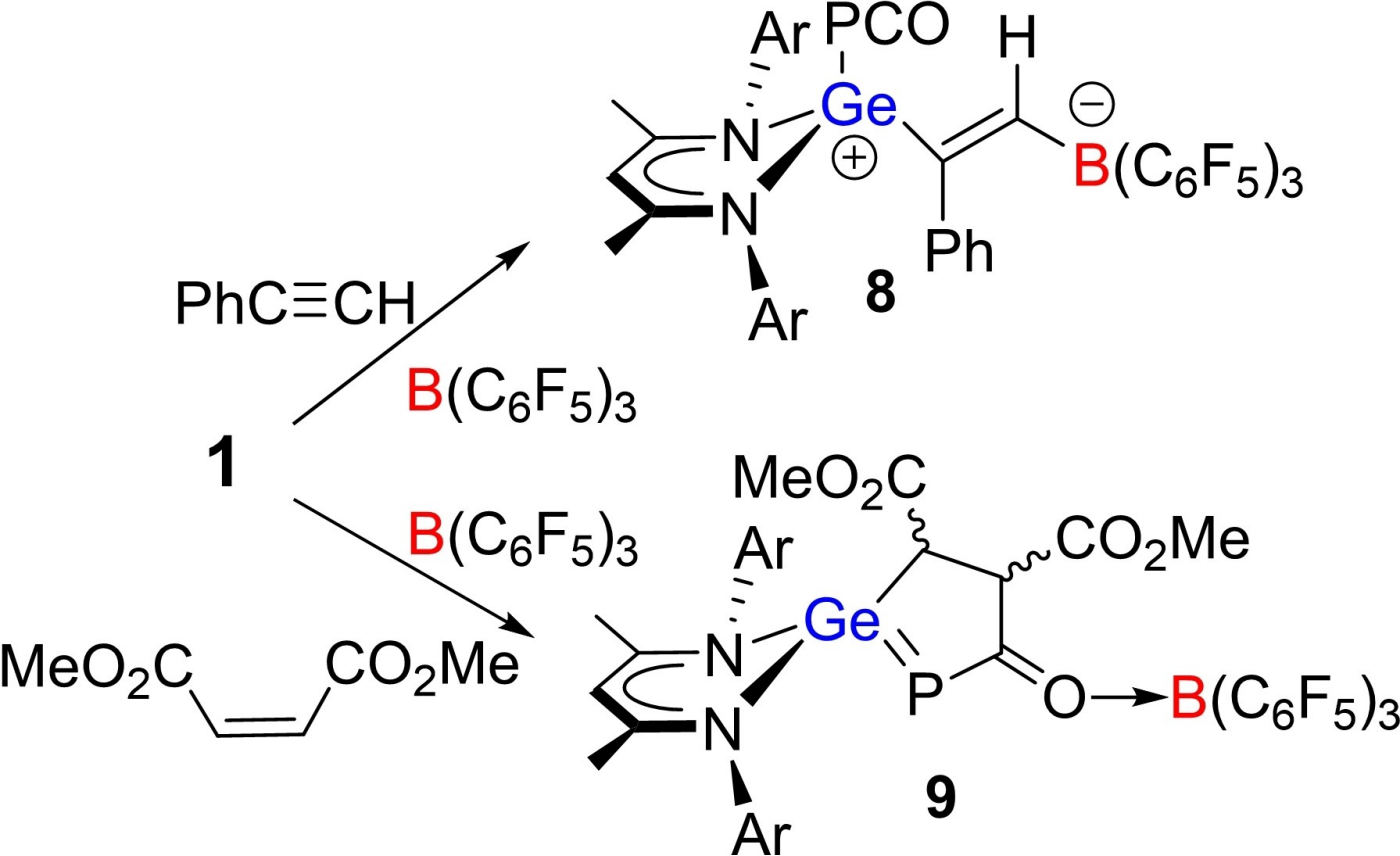
Synthesis of **8** and **9**.

**Figure 5:**
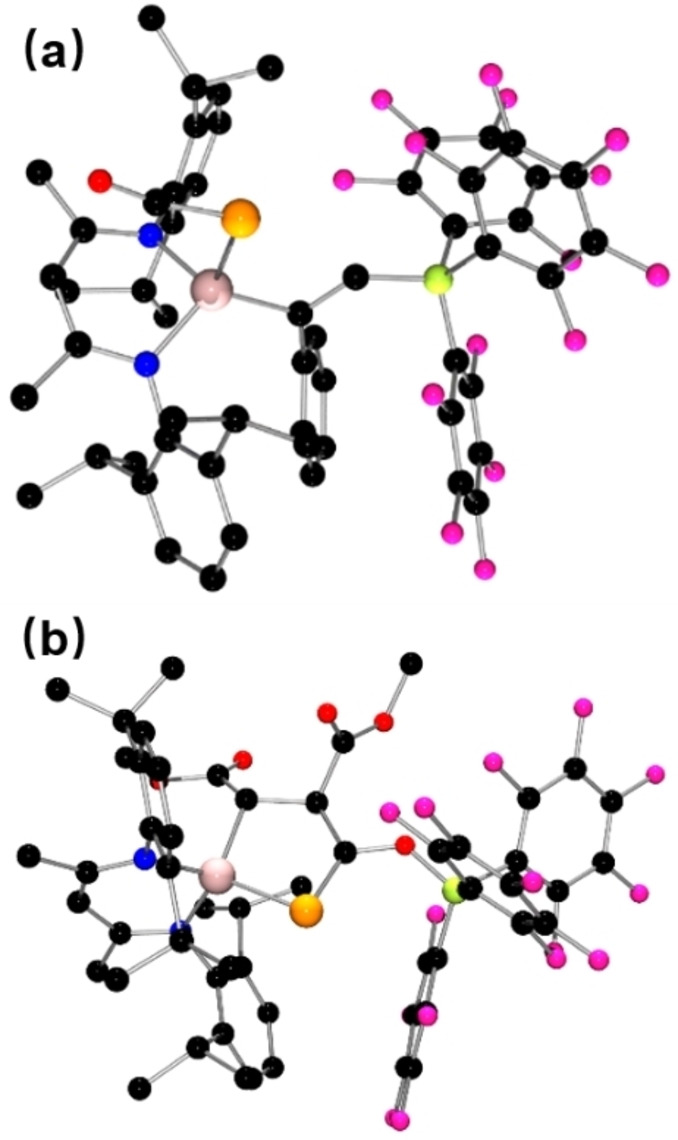
POV‐ray depiction of the molecular structure of (a) **8** and (b) **9**. Hydrogen atoms are omitted for clarity. C: black, F: hot pink, P: orange, N: blue, O: red, Ge: pale pink, B: yellow‐green.

The simultaneous mixture of **1** with B(C_6_F_5_)_3_ and dimethyl maleate (ratio 1 : 1 : 1) in toluene also gave two new species as evidenced by ^31^P resonances at −9.5 and −31.4 ppm and the broad ^11^B NMR signals at −1.7 ppm. While these two species proved challenging to separate, crystals of **9** that gives rise to the former ^31^P signal were isolated and an X‐ray crystallographic study (Figure 5b) affirmed the FLP addition of the nucleophilic Ge and the electrophilic C to the olefinic double bond. The diastereomeric, five‐membered germaphosphacycles L^1^GePC(OB(C_6_F_5_)_3_)(MeO_2_CCHCHCO_2_Me) exhibits a Ge−P double bond of 2.2518(9) Å, similar to that in **4** and to those previously reported.^[14]^ The C−C bond within the heterocycle is a typical single bond of 1.556(6) Å. Interestingly, efforts to prepare the analogous compound from more stable *trans*‐dimethyl fumarate led to no reaction even after heating at 100 °C for 24 h.

These latter two reactions were also considered computationally. For the electron‐rich alkyne PhCCH, computations revealed a transient adduct **B** with Lewis‐acidic B(C_6_F_5_)_3_ that is 8.8 kcal/mol endergonic (Scheme 5a). Rapid nucleophilic attack at the Ge center of **1** yields **8** in an overall reaction that is −14.0 kcal/mol exergonic over a low free energy barrier of only 12.0 kcal/mol (via **TS3**). An alternative reaction pathway affording a 5‐membed‐ring analogous to **5** derived from direct intramolecular FLP addition of PhCCH to **1** was found to be −20.6 kcal/mol exergonic but is prevented by a sizable barrier of 27.6 kcal/mol.

**Scheme 5:**
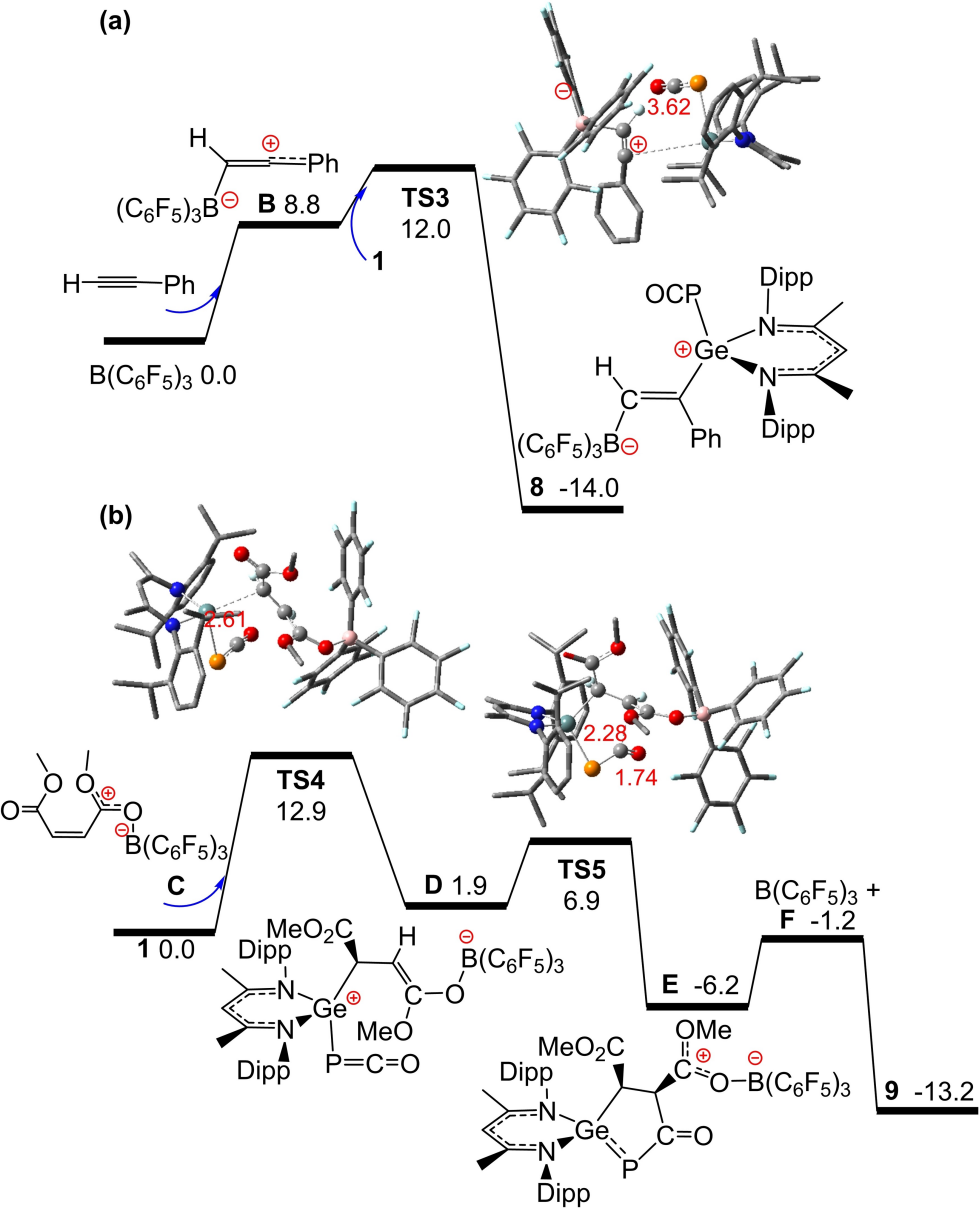
DFT‐computed free energy paths (in kcal/mol, at 298 K and 1 M) for the reaction of (a) PhCCH, B(C_6_F_5_)_3_ and **1**; (b) MeO_2_CC=CCO_2_Me, B(C_6_F_5_)_3_ and **1**. Crucial Ge, P, C, O, N, and H atoms are highlighted in ball‐and‐stick models as teal, orange, grey, red, blue and white balls, respectively, with selected bond lengths shown in Å.

Finally, binding of B(C_6_F_5_)_3_ to one ester group of the electron‐deficient olefin MeO_2_CC=CCO_2_Me to give **C** is −4.4 kcal/mol exergonic and enhances the electrophilicity of the C=C bond (Scheme 5b). Nucleophilic addition of **1** to **C** is 1.9 kcal/mol endergonic over a low barrier of 12.9 kcal/mol (via **TS4**) generating the zwitterion **D**. Subsequent rapid ring‐closing at the electrophilic phosphaketene carbon (via **TS5**) affords **E** and a subsequent shift of the B(C_6_F_5_)_3_ to the phosphaketene oxygen, affords the product **9**. For **9**, the respective DFT‐computed ^31^P and ^11^B signals at −14.9 ppm and −0.3 ppm agree well with experiment. The additional product observed experimentally is very likely the species **E**, as supported by the computed ^31^P and ^11^B signals at −27.6 ppm and −1.8 ppm, respectively. Consistent with experiment, the direct reaction of **1** and MeO_2_CC=CCO_2_Me alone encounters a sizable barrier of 23.8 kcal/mol, thus is slow even under moderate heating conditions. These results indicated that even in a molecule with multiple reactive sites, computation of reaction profiles provides insights into the subtleties of Lewis acidic/basic chemoselectivity.

In conclusion, despite the observation of direct reactivity of **1** with the Lewis acids (E(C_6_F_5_)_3_ E=B, Al), **1** participates in FLP chemistry with alkyne and olefin substrates. Specifically, **1** is shown to reacts with electron deficient alkynes or olefins, acting as an intramolecular FLP. In contrast, in the presence of B(C_6_F_5_)_3_ and an electron rich alkyne, **1** behaves as Ge‐based nucleophile to effect intermolecular FLP addition reactions. This range of reactivity demonstrates that the precise reaction pathway is controlled by the precise nature of the electrophile and nucleophile generated in solution. We are continuing to examine related systems containing Ge‐based nucleophiles and to explore the utility and generality of FLPs in inorganic and organic chemistry.

## Crystallographic details

Deposition Number(s) 2095456, 2125894, 21004686, 2095279, 2095451, 2125902, 2095286 and 2095291 contain(s) the supplementary crystallographic data for this paper. These data are provided free of charge by the joint Cambridge Crystallographic Data Centre and Fachinformationszentrum Karlsruhe Access Structures service.

## Conflict of interest

The authors declare no conflict of interest.

## Supporting information

As a service to our authors and readers, this journal provides supporting information supplied by the authors. Such materials are peer reviewed and may be re‐organized for online delivery, but are not copy‐edited or typeset. Technical support issues arising from supporting information (other than missing files) should be addressed to the authors.

Supporting Information

## Data Availability

The data that support the findings of this study are available in the supplementary material of this article.
