# Supplementary material for: The Varied Frustrated Lewis Pair Reactivity of the Germylene Phosphaketene (CH{(CMe)(2,6‐ i Pr2C6H3N)}2)GePCO
Source: Chemistry. 2022 Mar 24;28(25):e202200666. doi: 10.1002/chem.202200666 (PMC9314608; doi:10.1002/chem.202200666)
Supplement: Supplementary file 1 — Supporting Information [file CHEM-28-0-s001.pdf]

Chemistry–A European Journal

Supporting Information

The Varied Frustrated Lewis Pair Reactivity of the Germylene Phosphaketene $(CH\{(CMe)(2,6\text{-}{}^{i}Pr_2C_6H_3N)\}_2)GePCO$

Yile Wu,* Zhao Zhao, Ting Chen, Jingjie Tan, Zheng-Wang Qu,* Stefan Grimme, Yufen Zhao, and Douglas W. Stephan*

Contents

General Information 3

Experimental Section 4

- Synthesis of **2** 4
- Synthesis of **3** 5
- Synthesis of **4** 5
- Synthesis of **5** 6
- Synthesis of **6** 8
- Synthesis of **7** 9
- Synthesis of **8** 10
- Reaction of **1**, $B(C_6F_5)_3$ and dimethyl maleate (ratio 1:1:1) 11

X-ray Crystallography 12

- **Table S1**. Crystal data and structure refinement details for compounds **2**, **3**, **4** and **5** 12
- **Table S2**. Crystal data and structure refinement details for compounds **6**, **7**, **8** and **9** 13
- **Figure S1.** Thermal ellipsoid plot for **2** with the anisotropic displacement parameters depicted at the 30% probability level. Hydrogen atoms are omitted for clarity. 14
- **Figure S2.** Thermal ellipsoid plot for **3** with the anisotropic displacement parameters depicted at the 30% probability level. Hydrogen atoms are omitted for clarity. 14
- **Figure S3.** Thermal ellipsoid plot for **4** with the anisotropic displacement parameters depicted at the 30% probability level. Hydrogen atoms are omitted for clarity. 15
- **Figure S4.** Thermal ellipsoid plot for **5** with the anisotropic displacement parameters depicted at the 30% probability level. Hydrogen atoms are omitted for clarity. 15
- **Figure S5.** Thermal ellipsoid plot for **6** with the anisotropic displacement parameters depicted at the 30% probability level. Hydrogen atoms are omitted for clarity. 16
- **Figure S6.** Thermal ellipsoid plot for **7** with the anisotropic displacement parameters depicted at the 30% probability level. Disorder and hydrogen atoms are omitted for clarity. 16
- **Figure S7.** Thermal ellipsoid plot for **8** with the anisotropic displacement parameters depicted at the 30% probability level. Disorder and hydrogen atoms are omitted for clarity. 17
- **Figure S8.** Thermal ellipsoid plot for **9** with the anisotropic displacement parameters depicted at the 30% probability level. The hydrogen atoms except for those on C(1) and C(2) are omitted for clarity. 17

NMR Spectra 18

- **Figure S9.** ^{1}H NMR spectrum of **2** in C_6D_5Cl 18
- **Figure S10.** ^{13}C NMR spectrum of **2** in C_6D_5Cl 18
- **Figure S11.** ^{31}P NMR spectrum of **2** in C_6D_5Cl 19
- **Figure S12.** ^{19}F NMR spectrum of **2** in C_6D_5Cl 19
- **Figure S13.** ^{11}B NMR spectrum of **2** in C_6D_5Cl 20
- **Figure S14.** ^{1}H NMR spectrum of **3** in $CDCl_3$ 20
- **Figure S15.** ^{13}C NMR spectrum of **3** in $CDCl_3$ 21
- **Figure S16.** ^{31}P NMR spectrum of **3** in $CDCl_3$ 21
- **Figure S17.** ^{19}F NMR spectrum of **3** in $CDCl_3$ 22
- **Figure S18.** ^{1}H NMR spectrum of **4** in $CDCl_3$ 22
- **Figure S19.** ^{13}C NMR spectrum of **4** in CD_2Cl_2 23

Figure S20. ^{31}P NMR spectrum of **4** in $CDCl_3$ 23
Figure S21. ^{1}H NMR spectrum of **5** in CD_2Cl_2 24
Figure S22. ^{13}C NMR spectrum of **5** in CD_2Cl_2 24
Figure S23. ^{31}P NMR spectrum of **5** in CD_2Cl_2 25
Figure S24. ^{19}F NMR spectrum of **5** in CD_2Cl_2 25
Figure S25. ^{11}B NMR spectrum of **5** in CD_2Cl_2 26
Figure S26. ^{1}H NMR spectrum of **6** in CD_2Cl_2 26
Figure S27. ^{13}C NMR spectrum of **6** in CD_2Cl_2 27
Figure S28. ^{31}P NMR spectrum of **6** in CD_2Cl_2 27
Figure S29. ^{19}F NMR spectrum of **6** in CD_2Cl_2 28
Figure S30. ^{1}H NMR spectrum of **7** in C_6D_6 28
Figure S31. ^{13}C NMR spectrum of **7** in C_6D_6 29
Figure S32. ^{31}P NMR spectrum of **7** in C_6D_6 29
Figure S33. ^{19}F NMR spectrum of **7** in C_6D_6 30
Figure S34. ^{11}B NMR spectrum of **7** in C_6D_6 30
Figure S35. ^{1}H NMR spectrum of **8** in CD_2Cl_2 31
Figure S36. ^{13}C NMR spectrum of **8** in CD_2Cl_2 31
Figure S37. ^{31}P NMR spectrum of **8** in CD_2Cl_2 32
Figure S38. ^{19}F NMR spectrum of **8** in CD_2Cl_2 32
Figure S39. ^{11}B NMR spectrum of **8** in C_6D_6 33
Figure S40. ^{1}H NMR spectrum of **9** and side products in $CDCl_3$ 33
Figure S41. ^{31}P NMR spectrum of **9** and side products in $CDCl_3$ 34
Figure S42. ^{11}B NMR spectrum of **9** and side products in $CDCl_3$ 34
DFT Computational Details 35
Figure S43. TPSS-D3/def2-TZVP + COSMO(toluene) computed frontier molecular orbitals ... 37
Table S3. TPSS-D3/def2-TZVP + COSMO computed lowest imaginary frequency (ImF), zero-point energies (ZPE), gas-phase enthalpic (Hc) and Gibbs free-energy (Gc) corrections 38
Table S4. The TPSS-D3/def2-TZVP + COSMO optimized atomic Cartesian coordinates (in Å) in toluene solution 42
Reference 87

General Information

All manipulations were carried out on a Schlenk line or in an argon atmosphere glovebox. Solvents were dried using a MBraun solvent purification system, and stored over 3 Å sieves. Unless otherwise stated, commercial reagents were used without further purification. L^1GePCO (**1**, L^1 = CH{(CMe)-(2,6-^{i}Pr$_2$C$_6$H$_3$N)}$_2$),[S1] diisopropyl but-2-ynedioate[S2] were synthesized according to the literature methods. ^{1}H, ^{31}P, ^{19}F, ^{11}B and ^{13}C NMR spectra were recorded on a Bruker Ascend 500M or a Bruker Ascend 600M spectrometer. HRMS were recorded on a Thermo Scientific TM Q-Exactive PlusTM mass spectrometer. Single-crystal X-ray diffraction data were collected on a Bruker D8 QUEST diffractometer using Cu (60W, Diamond, μKα = 12.894 mm^{-1}) micro-focus X-ray sources. Using Olex2,[S3] the structure was solved with the XT[S4] structure solution program using Intrinsic Phasing and refined with the XL[S5] refinement package using Least Squares minimisation.

Experimental Section

Synthesis of **2**:

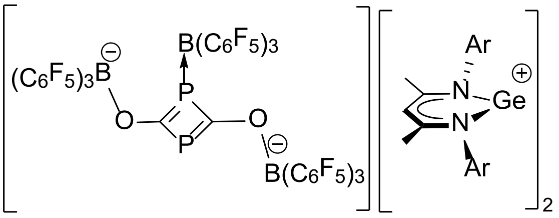

A mixture of **1** (0.1 mmol, 55 mg) and $B(C_6F_5)_3$ (0.1 mmol, 52mg) in 3 mL toluene was shook at room temperature and purple oily residues were obtained. The mixture was kept at -30℃ for additional 3 days. The residues were solidified to afford **2** as purple crystals. The solvent was decanted, and the crystals were washed with cold hexane and dried *in vacuo* to afford pure **2** (57.2mg, 63 %). ^{1}H NMR (500 MHz, C_6D_5Cl, ppm): δ 7.29 (t, *J* = 7.8 Hz, 4 H, Ar*H*), 7.14-7.13 (m, 8H, Ar*H*), 6.39 (s, 2 H, γ-*H*), 2.29 (sept, *J* = 6.7 Hz, 8 H, C*H*Me$_2$), 1.97 (s, 12 H, β-*Me*), 1.11 (d, *J* = 6.8 Hz, 24 H, CH*Me$_2$*), 0.97 (d, *J* = 6.9 Hz, 24 H, CH*Me$_2$*). ^{13}C{^{1}H} NMR (125 MHz, C_6D_5Cl, ppm): δ 250.6, 248.3 (*C*-O-B), 170.7 (*C*N), 174.5 (=*C*H), 149.7-147.8 (m, *C*$_6$F$_5$), 143.3 (*Ar*), 141.0-139.8, 139.1, 138.1-136.1 (m, *C*$_6$F$_5$), 131.1, 129.6, 125.3 (*Ar*), 119.2 (br, *C*$_6$F$_5$), 112.2 (γ-*C*H), 28.8, 25.5, 23.1, 22.6 (β-*Me*, *C*HMe$_2$ and CH*Me$_2$*). ^{31}P{^{1}H} NMR (202 MHz, C_6D_5Cl, ppm): δ 215.5 (s, 1 P), 125.6 (br, 1 P). ^{19}F{^{1}H} NMR (470 MHz, C_6D_5Cl, ppm) δ -128.2 (dd, *J* = 53.4, 23.1 Hz, 6F, P-B-*o*-C$_6$*F*$_5$), -132.0 (d, *J* = 19.6 Hz, 12F, O-B-*o*-C$_6$*F*$_5$), -158.2 (t, *J* = 20.3 Hz, 3F, P-B-*p*-C$_6$*F*$_5$), -159.4 (t, *J* = 20.3 Hz, 4F, O-B-*p*-C$_6$*F*$_5$), -161.9 (t, *J* = 20.6 Hz, 2F, O-B-*p*-C$_6$*F*$_5$), -164.2 (br, 6F, P-B-*m*-C$_6$*F*$_5$), -165.2 (t, *J* = 19.3 Hz, 8F, O-B-*m*-C$_6$*F*$_5$), -165.9 (t, 6F, *J* = 20.2 Hz, O-B-*m*-C$_6$*F*$_5$). ^{11}B{^{1}H} NMR (160 MHz, C_6D_5Cl, ppm) δ -7.5, -12.2. IR (Nujol mull, cm^{-1}): $\tilde{\nu}$ 3540.2, 1645.3, 1549.4, 1517.4, 1462.6, 1376.5, 1283.7,

1096.9, 977.6, 805.6, 773.4. HRMS (*m/z*): $[M_{cation}]^+$ Calcd. for $C_{29}H_{41}GeN_2^+$, 1205.23716; Found: 1205.23903.

Synthesis of **3**:

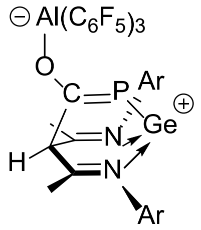

A solution of $Al(C_6F_5)_3$· 0.5toluene (0.2 mmol, 124 mg) in 3 mL toluene was added slowly into a solution of **1** (0.2 mmol, 110 mg) in 3 mL at -30°C. The mixture then store at -30°C overnight to afford yellow crystals of **3**. The yellowish crystals were washed with cold hexane (3×1 mL) and dried in vacuo for 6 hours to afford pure **3** (67.2 mg, 30 %). ^{1}H NMR (500 MHz, $CDCl_3$, ppm): δ 7.47 (t, *J* = 7.8 Hz, 2 H, Ar*H*), 7.34 (d, *J* = 7.8 Hz, 4H, Ar*H*), 6.33 (s, 1 H, γ-*H*), 2.75 (sept, *J* = 6.5 Hz, 4 H, C*H*Me$_2$), 2.14 (s, 6 H, β-*Me*), 1.21 (d, *J* = 6.7 Hz, 12 H, CH*Me$_2$*), 1.17 (d, *J* = 6.8 Hz, 12 H, CH*Me$_2$*). ^{13}C{^{1}H} NMR (150 MHz, $CDCl_3$, ppm): δ 170.2 (*C*-O-Al), 150.8-149.1 (m, *C*$_6$F$_5$), 144.0 (*Ar*), 142.2 (*C*N), 141.4-139.7(m, *C*$_6$F$_5$), 140.6 (*C*N), 137.3-135.7 (m, *C*$_6$F$_5$), 135.7, 130.4 (*Ar*), 128.8 (d, *J* = 122.7 Hz, γ-*C*H), 125.4 (*Ar*), 113.9 (m, *C*$_6$F$_5$), 29.2 (β-*Me*), 25.9 (*C*HMe$_2$), 23.8, 23.5 (CH*Me$_2$*). ^{31}P{^{1}H} NMR (202 MHz, $CDCl_3$, ppm): 169.7. ^{19}F{^{1}H} NMR (470 MHz, $CDCl_3$, ppm) δ -122.5 (dd, *J* = 27.3, 11.3 Hz, 6F, *o*-C$_6$*F*$_5$), -157.0 (t, *J* = 19.4 Hz, 3F, *p*-C$_6$*F*$_5$), -163.7 (m, 6F, *m*-C$_6$*F*$_5$). IR (Nujol mull, cm^{-1}): $\tilde{\nu}$ 1697.8, 1636.4, 1506.0, 1458.9, 1376.8, 1262.1, 1166.3, 1067.1, 955.8, 801.0, 721.0, 670.6, 637.2, 612.3, 498.2. HRMS (*m/z*): $[M + MeCN + Na]^+$ Calcd. for $C_{50}H_{44}AlF_{15}GeN_3NaO_5P^+$, 1142.19019; Found: 1142.19304.

Synthesis of **4**:

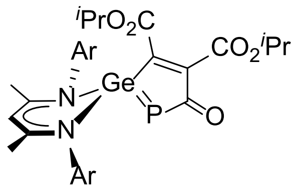

At -30 °C, a solution of diisopropyl but-2-ynedioate (99.1mg, 0.5 mmol) in toluene (2 mL) was added slowly to a solution of **1** (275 mg, 0.5 mmol) in toluene (5 mL). The mixture was stirred and slowly warmed to room temperature. After stirring for additional 24 h, the solvent was removed and the residues were extracted with ^{n}hexane/toluene (10/1, 5 mL). The residual pale dark violet powder (210.1mg, 56%) solid was proved to be pure **7**. Violet block crystals suitable for X-ray test were obtained after stored the thf/toluene solution of **4** at -30°C. ^{1}H NMR (500 MHz, $CDCl_3$, ppm): δ 7.33 (t, J = 7.7 Hz, 2 H, Ar*H*), 7.20 (d, J = 8.1 Hz, 4 H, Ar*H*), 5.62 (s, 1 H, γ-*H*), 5.22 (sept, J = 6.3 Hz, 1 H, OC*H*Me$_2$), 5.10 (sept, J = 6.4 Hz, 1 H, OC*H*Me$_2$), 3.00 (sept, J = 6.7 Hz, 2 H, ArC*H*Me$_2$), 2.91 (sept, J = 6.7 Hz, 2 H, ArC*H*Me$_2$), 2.00 (s, 6 H, β-*Me*), 1.35 (dd, J = 6.5, 3.2 Hz, 12 H, CH*Me$_2$*), 1.21 (dd, J = 6.5, 1.2 Hz, 12 H, CH*Me$_2$*), 1.13 (dd, J = 10.1, 6.8 Hz, 12 H, CH*Me$_2$*). ^{13}C{^{1}H} NMR (125 MHz, CD_2Cl_2, ppm): δ 222.0 (d, $J_{C\text{-}P}$ = 86.7 Hz, P-*C*=O), 172.9 (Ge-*C*=C), 166.5 (*C*N), 163.8, 161.2 (*C*(O)O^{i}Pr), 146.4, 144.3, 136.6, 129.6, 129.4, 128.6, 125.7, 125.2 (Ge-C=*C* and *Ar*), 101.3 (γ-*C*H), 69.4, 68.9 (O*C*HMe$_2$), 30.2, 27.9, 25.9, 25.0, 24.9, 24.1, 24.0, 22.4, 21.8 (β-*Me*, *C*HMe$_2$ and CH*Me$_2$*). ^{31}P{^{1}H} NMR (202 MHz, $CDCl_3$, ppm): δ -119.77. IR (Nujol mull, cm^{-1}): $\tilde{\nu}$ 1737.4, 1710.4, 1519.2, 1462.7, 1376.8, 1259.5, 1097.1, 801.7, 727.1. HRMS (*m/z*): [M + H]$^+$ Calcd. for $C_{40}H_{56}GeN_2O_5P^+$, 749.31331; Found: 749.31335.

Synthesis of **5**:

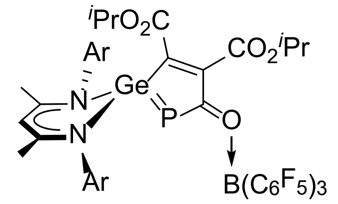

A solution of $B(C_6F_5)_3$ (0.1 mmol, 52mg) in toluene (3 mL) was added dropwise into a solution of **4** (0.1 mmol, 90 mg) in toluene (5 mL) at -30°C. The mixture was stirred at ambient temperature for another 30 min. The solvent was removed under vacuum and the residue was washed by hexane/toluene (10/1) to afford **5** as orange powders (89.9mg, 71%). Orange red crystals suitable for X-ray diffraction analysis by recrystallization from a thf/toluene solution. ^{1}H NMR (500 MHz, CD_2Cl_2, ppm): δ 7.49 (t, *J* = 7.8 Hz, 2 H, Ar*H*), 7.30 (dd, *J* = 7.8, 1.1 Hz, 2 H, Ar*H*), 7.20 (dd, *J* = 7.8, 1.1 Hz, 2 H, Ar*H*), 5.75 (s, 1 H, γ-*H*), 5.22 (sept, *J* = 6.3 Hz, 1 H, OC*H*Me$_2$), 4.92 (sept, *J* = 6.3 Hz, 1 H, OC*H*Me$_2$), 2.85 (sept, *J* = 6.7 Hz, 2 H, ArC*H*Me$_2$, overlapped), 2.77 (sept, *J* = 6.7 Hz, 2 H, ArC*H*Me$_2$, overlapped), 2.08 (s, 6 H, β-*Me*), 1.34 (d, *J* = 6.3 Hz, 6 H, CH*Me$_2$*), 1.20 (d, *J* = 6.7 Hz, 6 H, CH*Me$_2$*), 1.16 (d, *J* = 6.8 Hz, 6 H, CH*Me$_2$*), 1.12 (d, *J* = 6.7 Hz, 6 H, CH*Me$_2$*), 1.09 (d, *J* = 6.8 Hz, 6 H, CH*Me$_2$*), 1.03 (d, *J* = 5.4 Hz, 6 H, CH*Me$_2$*). ^{13}C{^{1}H} NMR (125 MHz, CD_2Cl_2, ppm): δ 219.7 (d, $J_{C\text{-}P}$ = 83.5 Hz, P*C*=O-BCF), 174.0 (Ge-*C*=C), 163.5 (*C*N), 161.5 (Ge-C=*C*), 160.5, 160.4 (*C*(O)O^{i}Pr), 149.3-147.4, 149.3-147.4 (m, *C*$_6$F$_5$), 146.4, 143.5 (*Ar*), 140.6-138.6, 137.9-135.8 (m, *C*$_6$F$_5$), 134.5, 130.7, 126.1, 125.4 (*Ar*), 101.6 (γ-*C*H), 70.2 (d, *J* = 4.3 Hz, O*C*HMe$_2$), 30.3, 28.0, 25.2, 25.1, 24.3, 23.8, 23.7, 22.4, 21.2 (β-*Me*, *C*HMe$_2$ and CH*Me$_2$*). ^{31}P{^{1}H} NMR (202 MHz, CD_2Cl_2, ppm): δ -32.9 (sept, *J* = 14.2 Hz). ^{19}F{^{1}H} NMR (470 MHz, CD_2Cl_2, ppm) δ -133.3 (br, 6F, *o*-C$_6$*F*$_5$), -160.2 (br, 3F, *p*-C$_6$*F*$_5$), -166.5 (td, 6F, *J* = 10.8, 6.3 Hz, *m*-C$_6$*F*$_5$). ^{11}B{^{1}H} NMR (160 MHz, CD_2Cl_2, ppm) δ -1.3 (br). IR (Nujol mull, cm^{-1}): $\tilde{\nu}$ 1742.0, 1718.9, 1641.0, 1531.2, 1514.1, 1465.2,

1376.3, 1338.9, 1106.0, 979.8, 802,9. HRMS (*m/z*): $[M + H]^+$ Calcd. for $C_{58}H_{55}BF_{15}GeN_2NaO_5P^+$, 1283.28061; Found: 1283.28003.

Synthesis of **6**:

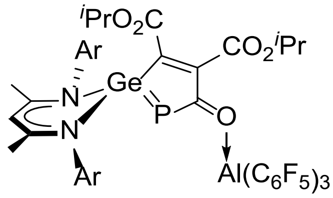

6 was prepared by the same procedure for **5**. $Al(C_6F_5)_3$·0.5 toluene (0.1 mmol, 62mg) and **4** (0.1 mmol, 90 mg) were used, and the product **6** was isolated as orange powder (87.4mg, 66%). Orange crystals of **6** suitable for X-ray diffraction analysis were obtained from a thf/benzene solution. ^1H NMR (500 MHz, CD_2Cl_2, ppm): δ 7.46 (t, *J* = 7.8 Hz, 2 H, Ar*H*), 7.30 (dd, *J* = 7.8, 1.2 Hz, 2 H, Ar*H*), 7.21(dd, *J* = 7.8 Hz, 2 H, Ar*H*), 5.77 (s, 1 H, γ-*H*), 5.22 (sept, *J* = 6.2 Hz, 1 H, OC*H*Me2), 4.97 (sept, *J* = 6.3 Hz, 1 H, OC*H*Me2), 2.85 (sept, *J* = 7.0 Hz, 2 H, ArC*H*Me2), 2.80 (sept, *J* = 6.8 Hz, 2 H, ArC*H*Me2), 2.08 (s, 6 H, β-*Me*), 1.35 (d, *J* = 6.3 Hz, 6 H, CH*Me2*), 1.21 (d, *J* = 6.7 Hz, 6 H, CH*Me2*), 1.17 (d, *J* = 6.8 Hz, 6 H, CH*Me2*), 1.13 (d, *J* = 6.8 Hz, 6 H, CH*Me2*), 1.09 (d, *J* = 6.8 Hz, 6 H, CH*Me2*), 1.03 (d, *J* = 6.3 Hz, 6 H, CH*Me2*). $^{13}C\{^1H\}$ NMR (125 MHz, CD_2Cl_2, ppm): δ 223.7 (d, $J_{C\text{-}P}$ = 77.8 Hz, P*C*=O-Al), 174.2 (Ge-*C*=C), 163.5 (*C*N), 161.4 (Ge-C=*C*), 160.4, 160.3 (*C*(O)O^{i}Pr), 151.1-149.0, (m, *C*6F5), 146.3, 143.4 (*Ar*), 142.1-140.1, 137.6-135.6 (m, *C*6F5), 136.0, 134.6, 130.7, 129.4, 128.6, 126.2, 125.4 (*Ar*), 116.1 (br, *C*6F5), 101.7 (γ-*C*H), 70.3, 70.2 (O*C*HMe2), 32.0, 30.3, 28.1, 25.3 (β-*Me*, *C*HMe2 and CH*Me2*), 25.1 (d, *J* = 4.5 Hz, CH*Me2*), 24.4, 23.6 (d, *J* = 7 Hz, CH*Me2*), 22.4, 21.3 (CH*Me2*). $^{31}P\{^1H\}$ NMR (202 MHz, CD_2Cl_2, ppm): δ -24.2 (sept, *J* = 10.8 Hz). $^{19}F\{^1H\}$ NMR (470 MHz, CD_2Cl_2, ppm) δ -122.7 (m, 6F, *o*-C6*F*5), -156.4 (t, 3F, *J* = 19.4 Hz, *p*-C6*F*5), -163.8

(m, 6F, *m*-C_6F_5). IR (Nujol mull, cm^{-1}): $\tilde{\nu}$ 1740.3, 1716.2, 1639.5, 1537.0, 1507.6, 1462.9, 1376.9, 1170.2, 1103.7, 1068.4, 957.5, 722.0. HRMS (*m/z*): $[M + MeCN + H]^+$ Calcd. for $C_{60}H_{59}AlF_{15}GeN_3O_5P^+$, 1318.29745; Found: 1318.29718.

Synthesis of **7**:

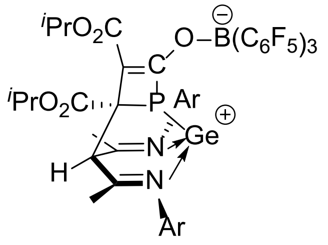

A solution of **1** (0.1 mmol, 90 mg) and $B(C_6F_5)_3$ (0.1 mmol, 52mg) in toluene (10 mL) was shaken and stored at room temperature overnight. The solvent was removed under vacuum to afford **7** as pure powders (89.9mg, 71%). Yellow crystals of **7** suitable for X-ray diffraction analysis were obtained after recrystallization from a DCM/pentane solution. Meanwhile, a trace amount of **5** was also obtained as orange crystals.

7: ^{1}H NMR (500 MHz, C_6D_6, ppm): δ 7.00 (t, *J* = 7.3 Hz, 1 H, Ar*H*), 6.93 (t, *J* = 7.6 Hz, 2 H, Ar*H*), 6.85 (t, *J* = 7.8 Hz, 2 H, Ar*H*), 6.75 (d, *J* = 7.7 Hz, 1 H, Ar*H*), 5.40 (d, *J* = 6.8 Hz, 1 H, γ-*H*), 5.22 (sept, *J* = 6.2 Hz, 1 H, OC*H*Me$_2$), 5.11 (sept, *J* = 6.3 Hz, 1 H, OC*H*Me$_2$), 2.44 (sept, *J* = 6.4 Hz, 1 H, ArC*H*Me$_2$), 2.36 (sept, *J* = 6.7 Hz, 1 H, ArC*H*Me$_2$), 2.06-1.99 (m, *2* H, ArC*H*Me$_2$, overlapped), 1.99 (s, 3 H, β-*Me*, overlapped), 1.79 (s, 3 H, β-*Me*), 1.47 (d, *J* = 6.2 Hz, 3 H, CH*Me$_2$*), 1.41 (d, *J* = 6.1 Hz, 3 H, CH*Me$_2$*), 1.22 (d, *J* = 6.3 Hz, 3 H, CH*Me$_2$*), 1.16 (d, *J* = 6.4 Hz, 3 H, CH*Me$_2$*), 1.05 (d, *J* = 6.4 Hz, 3 H, CH*Me$_2$*), 0.99 (dd, *J* = 6.3, 2.8 Hz, 6 H, CH*Me$_2$*), 0.94 (d, *J* = 6.6 Hz, 3 H, CH*Me$_2$*), 0.84 (d, *J* = 6.7 Hz, 3 H, CH*Me$_2$*), 0.76-0.73 (m, 9 H, CH*Me$_2$*). $^{13}C\{^1H\}$ NMR (125 MHz, C_6D_6, ppm): δ 191.8 (d, $J_{C\text{-}P}$ = 36.7 Hz, P*C*=O-BCF), 177.4, 176.9 (*C*N), 175.5, 162.0 (*C*(O)O^{i}Pr), 144.0, 142.3, 141.5, 141.0, 137.8, 134.7, 130.5,

129.8, 126.7, 126.3, 126.0, 125.3 (*Ar*), 111.7 (d, J_{C-P} = 15.4 Hz, P-C=*C*-C(O)O^{i}Pr), 70.5, 67.5 (O*C*HMe$_2$), 59.8 (γ-*C*H), 49.7 (d, J_{C-P} = 18.9 Hz, P- *C*-C(O)O^{i}Pr), 31.0, 29.9, 29.3, 29.2, 27.9, 27.8, 27.7, 26.9, 26.8, 26.0, 25.9, 24.4, 24.2, 24.1, 22.1, 22.0, 21.9, 21.5, 21.3 (β-*Me*, *C*HMe$_2$ and CH*Me$_2$*). ^{31}P{^{1}H} NMR (202 MHz, C_6D_6, ppm): δ 33.4. ^{19}F{^{1}H} NMR (470 MHz, C_6D_6, ppm) δ -132.0 (br, 6F, *o*-C_6F_5), -159.3 (br, 3F, *p*-C_6F_5), -166.0 (br, 6F, *m*-C_6F_5). ^{11}B{^{1}H} NMR (160 MHz, C_6D_6, ppm) δ -1.7 (br). IR (Nujol mull, cm^{-1}): ṽ 3058.4, 2728.8, 2362.1, 1950.2, 1890.2, 1733.2, 1644.4, 1514.6, 1458.7, 1377.2, 1082.9, 976.3, 828.2, 748.9, 693.6, 478.4. HRMS (*m/z*): [M + Na]$^+$ Calcd. for $C_{58}H_{55}BF_{15}GeN_2NaO_5P^+$, 1283.28061; Found: 1283.28076.

Synthesis of **8**:

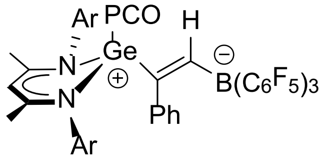

At -30 °C, a solution of B(C_6F_5)$_3$ (0.1 mmol, 52 mg) in 2 mL toluene was added dropwise into a solution of **1** (0.1 mmol, 55 mg) and phenylacetylene (0.1mmol, 10.2 mg) in 5 mL hexane. The mixture was slowly warm to room temperature and stireed overnight to afford brown powders. The mixture was decanted, washed with cold acetonitrile (5 mL) and dried in vacuo to afford a brown power of **8** (59 mg, 51 %). Crystals suitable for single-crystal XRD analysis were obtained by storage a toluene solution of **8** at -30°C overnight. ^{1}H NMR (500 MHz, CD_2Cl_2, ppm): δ 7.50-7.47 (m, 2 H, Ar*H*), 7.34-7.29 (m, 3H, =C*H*B and Ar*H*, overlapped), 7.24-7.22 (m, 2H, Ar*H*), 6.78-6.75 (m, 1H, Ar*H*), 6.50 (t, *J* = 7.8 Hz, 2 H, Ar*H*), 5.93 (d, *J* = 7.3 Hz, 2 H, Ar*H*), 5.81 (s, 1 H, γ-*H*), 3.34 (sept, *J* = 6.4 Hz, 2 H, C*H*Me$_2$), 2.87 (sept, *J* = 6.7 Hz, 2 H, C*H*Me$_2$), 2.02 (s, 6 H, β-*Me*), 1.29 (d, *J* = 6.7 Hz, 6 H, CH*Me$_2$*), 1.15 (d, *J* = 6.7 Hz, 6 H, CH*Me$_2$*), 1.09 (d, *J* = 6.8 Hz, 6 H, CH*Me$_2$*), 1.00 (d, *J* = 6.7 Hz, 6 H, CH*Me$_2$*). ^{13}C{^{1}H}

NMR (125 MHz, CD_2Cl_2, ppm): δ 192.4 (d, $J_{C\text{-}P}$ = 114.7 Hz, P=*C*=O), 174.7 (*C*N), 174.5 (=*C*H), 149.0-146.9 (m, *C*$_6$F$_5$), 146.1 (Ge-*C*=C), 143.9 (*Ar*), 139.7-137.3 (m, *C*$_6$F$_5$), 137.5 (*Ar*), 136.7-135.5 (m, *C*$_6$F$_5$), 131.0, 130.8, 129.3, 129.0, 127.8 (*Ar*), 126.8 (br, *C*$_6$F$_5$), 126.7, 125.7 (*Ar*), 105.3 (γ-*C*H), 30.1, 30.0, 28.9, 25.6, 25.1, 24.9, 23.2 (β-*Me*, *C*HMe$_2$ and CH*Me*$_2$). $^{31}P\{^1H\}$ NMR (202 MHz, CD_2Cl_2, ppm): δ -304.2. $^{19}F\{^1H\}$ NMR (470 MHz, CD_2Cl_2, ppm) δ -129.8 (d, *J* = 21.9 Hz, 6F, *o*-C$_6$*F*$_5$), -163.6 (t, *J* = 21.3 Hz, 3F, *p*-C$_6$*F*$_5$), -167.2 (t, 6F, *J* = 9.8 Hz, *m*-C$_6$*F*$_5$). $^{11}B\{^1H\}$ NMR (160 MHz, CD_2Cl_2, ppm) δ -16.4. IR (Nujol mull, cm^{-1}): $\tilde{v}$ 2357.1, 1959.9, 1639.6, 1508.4, 1462.8, 1377.1, 1083.8, 973.5, 797.3, 722.4. HRMS (*m/z*): [M + MeOH + H]$^+$ Calcd. for $C_{57}H_{52}BF_{15}GeN_2O_2P^+$, 1197.28262; Found: 1197.28202.

Reaction of **1**, $B(C_6F_5)_3$ and dimethyl maleate (ratio 1:1:1):

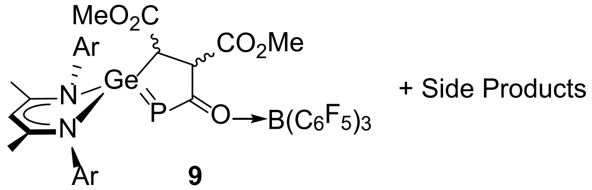

9

At -30 °C, **1** (110 mg, 0.2 mmol) and dimethyl maleate (28.8mg, 0.2 mmol) was disloved in toluene (3 mL). A solution of $B(C_6F_5)_3$ (0.2 mmol, 104mg) in toluene (1 mL) was added slowly. Pale yellow crystals suitable for single crystal XRD analysis were obtained after storage the reaction mixture at -30°C overnight. The crude product of **9** was washed with cold hexane (3 ×1 mL) and dry under vacuum. $^{31}P\{^1H\}$ NMR (202 MHz, $CDCl_3$, ppm): δ -9.5. $^{11}B\{^1H\}$ NMR (160 MHz, $CDCl_3$, ppm) δ -1.7 (br). HRMS (*m/z*): [M - H]$^-$ Calcd. for $C_{54}H_{48}BF_{15}GeN_2O_5P^-$, 1205.23716; Found: 1205.23903.

X-ray Crystallography

Table S1. Crystal data and structure refinement details for compounds **2**, **3**, **4** and **5**

	**2**	**3**	**4·toluene**	**5**
CCDC	2095291	2125894	2104686	2095279
Empirical formula	$C_{128}H_{98}B_3F_{45}Ge_2N_4O_2P_2$	$C_{48}H_{41}AlF_{15}GeN_2OP$	$C_{47}H_{63}GeN_2O_5P$	$C_{58}H_{55}BF_{15}GeN_2O_5P$
Formula weight	2818.65	1077.37	839.55	1259.41
Temperature, K	101	100.00	100.0	100
Crystal system	triclinic	triclinic	orthorhombic	monoclinic
Space group	*P*-1	*P*-1	*Pbca*	$P2_1/n$
a, Å	18.8995(5)	13.1391(14)	19.875(2)	13.5193(9)
b, Å	18.9889(5)	13.2001(13)	18.527(2)	20.4356(13)
c, Å	20.8959(6)	15.4297(18)	24.711(3)	21.4208(14)
α, deg	84.2660(10)	72.254(6)	90	90
β, deg	79.8760(10)	73.396(7)	90	104.668(2)
γ, deg	60.4850(10)	73.799(6)	90	90
V, $Å^3$	6423.6(3)	2387.9(5)	9099(2)	5725.2(6)
Z	2	2	8	4
D_{calcd}, g/cm^3	1.457	1.498	1.226	1.461
$μ/mm^{-1}$	1.854	1.498	1.6	1.865
F(000)	2848	1092.0	3568	2576
θ range, °	2.198 – 68.314	3.082 – 68.457	3.577 – 58.896	3.037 – 72.365
Index ranges	$-22 \leq h \leq 22$	$-15 \leq h \leq 15$	$-22 \leq h \leq 22$	$-16 \leq h \leq 16$
	$-22 \leq k \leq 22$	$-15 \leq k \leq 15$	$-20 \leq k \leq 20$	$-25 \leq k \leq 22$
	$-25 \leq l \leq 25$	$-18 \leq l \leq 18$	$-27 \leq l \leq 27$	$-26 \leq l \leq 26$
Reflections collected	230159	63203	172386	157648
Independent reflections	23524	8570	6502	11299
	R_{int} = 0.0553	R_{int} = 0.1076,	R_{int} = 0.1631,	R_{int} = 0.0449
	R_{sigma} = 0.0222	R_{sigma} = 0.0614	R_{sigma} = 0.0481	Rsigma = 0.0168
Data/restraints/parameters	23524/0/1697	8570/0/632	6502/0/520	11299/0/772
Goodness-of-fit on F^2	1.042	1.095	1.078	1.029
Final R indexes [I>=2σ (I)]	R_1 = 0.0342	R_1 = 0.0781	R_1 = 0.0647	R_1 = 0.0263
	wR_2 = 0.0920	wR_2 = 0.1996	wR_2 = 0.1495	wR_2 = 0.0686
Final R indexes [all data]	R_1 = 0.0385	R_1 = 0.0961	R_1 = 0.0757	R_1 = 0.0275
	wR_2 = 0.0949	wR_2 = 0.2091	wR_2 = 0.1569	wR_2 = 0.0694
Largest diff. peak/hole, $e/Å^{-3}$	1.62/-0.61	0.86/-0.92	0.51/-1.01	0.47/-0.39

Table S2. Crystal data and structure refinement details for compounds **6**, **7**, **8** and **9**

	**6·benzene**	**7**	**8·0.5(toluene)**	**9**
CCDC	2095451	2125902	2095446	2095286
Empirical formula	$C_{64}H_{61}AlF_{15}GeN_2O_5P$	$C_{58}H_{55}BF_{15}GeN_2O_5P$	$C_{59.5}H_{50.5}BF_{15}GeN_2OP$	$C_{108}H_{98}B_2F_{30}Ge_2N_4O_{10}P_2$
Formula weight	1353.68	1259.41	1208.89	2410.64
Temperature, K	227	273.00	103	150
Crystal system	orthorhombic	monoclinic	triclinic	triclinic
Space group	*Pbca*	$P2_1/n$	*P*-1	*P*-1
a, Å	19.5909(4)	15.0553(3)	12.6050(6)	13.6611(11)
b, Å	22.6999(5)	24.4759(4)	12.8881(7)	20.0829(14)
c, Å	28.5545(6)	17.6531(2)	19.1033(9)	25.1612(18)
α, deg	90	90	92.121(3)	85.459(4)
β, deg	90	90.9920(10)	106.687(3)	82.077(5)
γ, deg	90	90	112.806(3)	81.462(5)
V, $Å^3$	12698.5(5)	6504.05(18)	2701.4(2)	6749.3(9)
Z	8	4	2	2
D_{calcd}, g/cm^3	1.416	1.286	1.486	1.186
μ/mm^{-1}	1.853	1.642	1.896	1.561
F(000)	5552	2576.0	1233	2456
θ range, °	3.095 – 75.289	3.087 – 63.845	2.449 – 68.358	2.229 – 72.462
Index ranges	$-24 \le h \le 24$	$-17 \le h \le 17$	$-15 \le h \le 15$	$-16 \le h \le 16$
	$-21 \le k \le 28$	$-28 \le k \le 28$	$-15 \le k \le 15$	$-24 \le k \le 24$
	$-35 \le l \le 35$	$-18 \le l \le 20$	$-22 \le l \le 22$	$-31 \le l \le 31$
Reflections collected	181293	122683	67365	234098
Independent reflections	13096	10757	9870	26614
	R_{int} = 0.0917	R_{int} = 0.0961	R_{int} = 0.1491	R_{int} = 0.1487
	R_{sigma} = 0.0457	R_{sigma} = 0.0339	R_{sigma} = 0.0800	R_{sigma} = 0.0703
Data/restraints/parameters	13096/0/816	10757/51/782	9870/0/750	26614/2/1447
Goodness-of-fit on F^2	1.125	1.056	0.999	1.073
Final R indexes [I>=2σ (I)]	R_1 = 0.0722	R_1 = 0.0423	R_1 = 0.0732	R_1 = 0.0552
	wR_2 = 0.1926	wR_2 = 0.1150	wR_2 = 0.1960	wR_2 = 0.1508
Final R indexes [all data]	R_1 = 0.0995	R_1 = 0.0554	R_1 = 0.1046	R_1 = 0.0787
	wR_2 = 0.2362	wR_2 = 0.1232	wR_2 = 0.2250	wR_2 = 0.1660
Largest diff. peak/hole, e/Å^{-3}	0.58/-1.42	0.36/-0.44	0.76/-0.96	1.25/-1.04

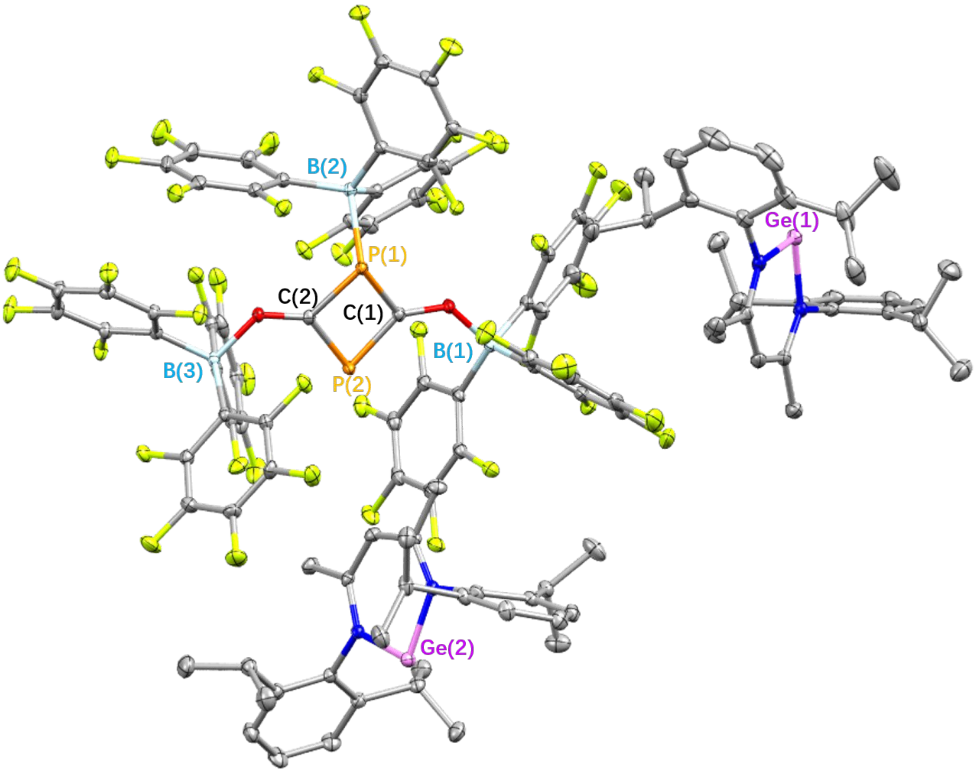

Figure S1. Thermal ellipsoid plot for **2** with the anisotropic displacement parameters depicted at the 30% probability level. Hydrogen atoms are omitted for clarity.

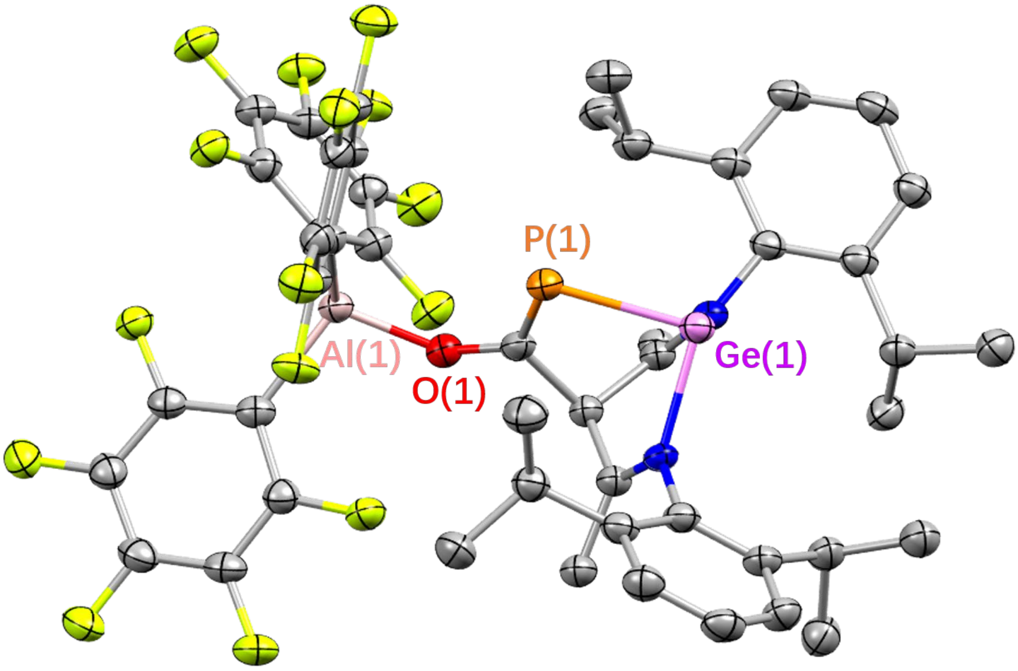

Figure S2. Thermal ellipsoid plot for **3** with the anisotropic displacement parameters depicted at the 30% probability level. Hydrogen atoms are omitted for clarity.

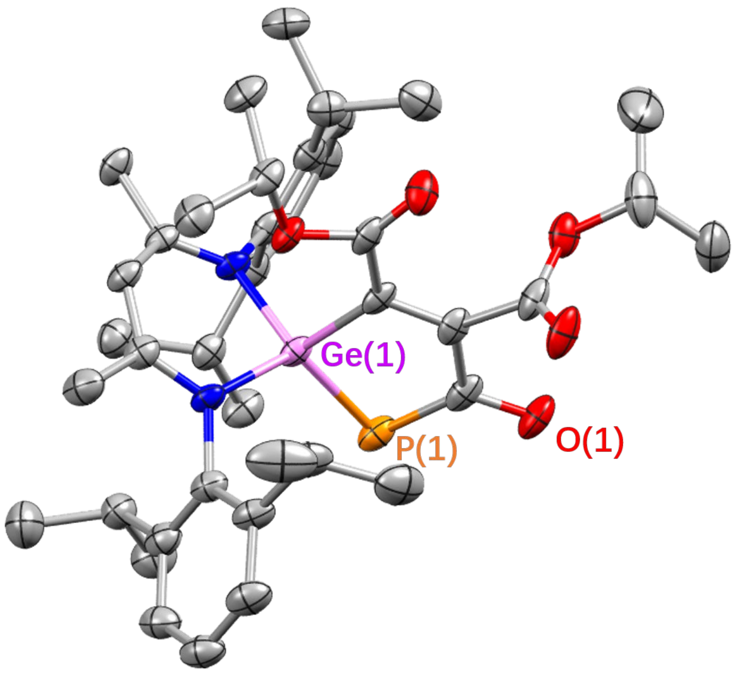

Figure S3. Thermal ellipsoid plot for **4** with the anisotropic displacement parameters depicted at the 30% probability level. Hydrogen atoms are omitted for clarity.

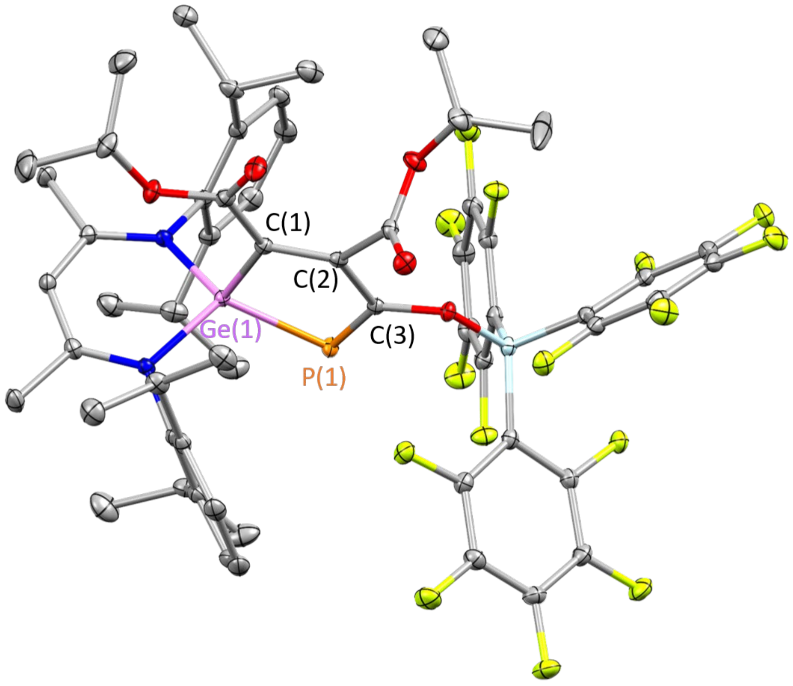

Figure S4. Thermal ellipsoid plot for **5** with the anisotropic displacement parameters depicted at the 30% probability level. Hydrogen atoms are omitted for clarity.

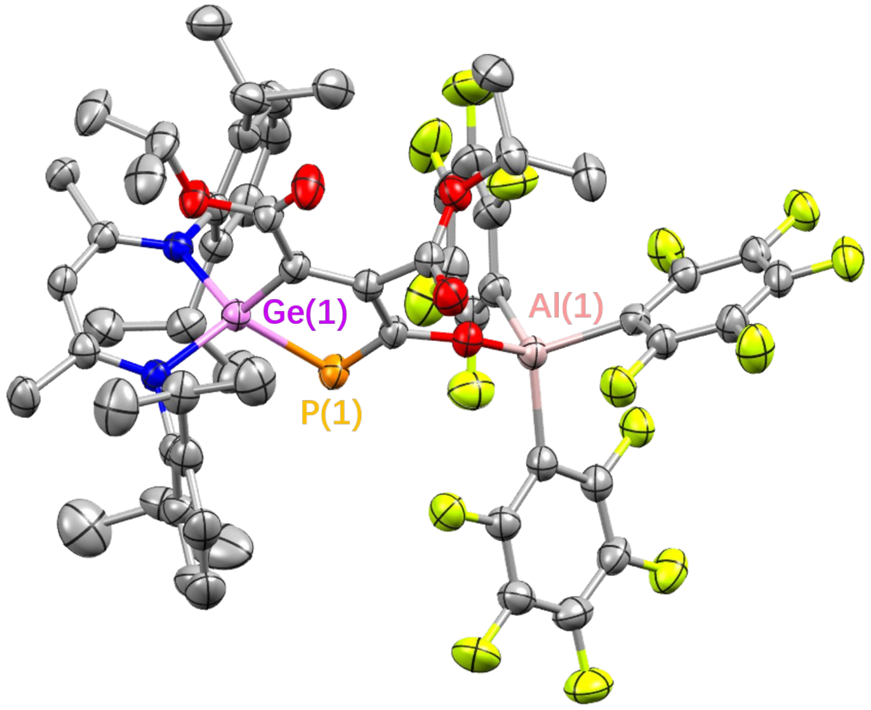

Figure S5. Thermal ellipsoid plot for **6** with the anisotropic displacement parameters depicted at the 30% probability level. Hydrogen atoms are omitted for clarity.

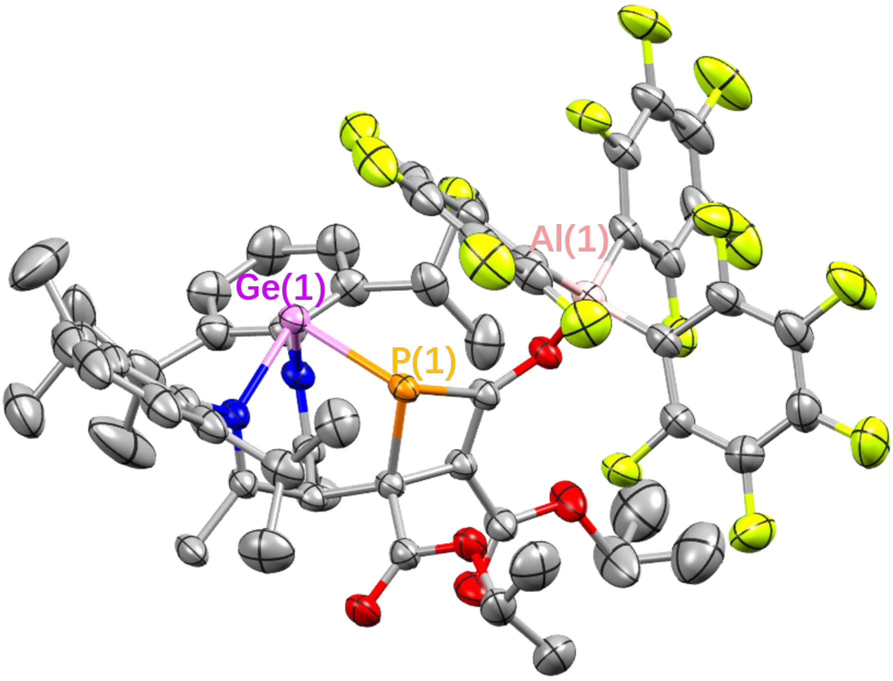

Figure S6. Thermal ellipsoid plot for **7** with the anisotropic displacement parameters depicted at the 30% probability level. Disorder and hydrogen atoms are omitted for clarity.

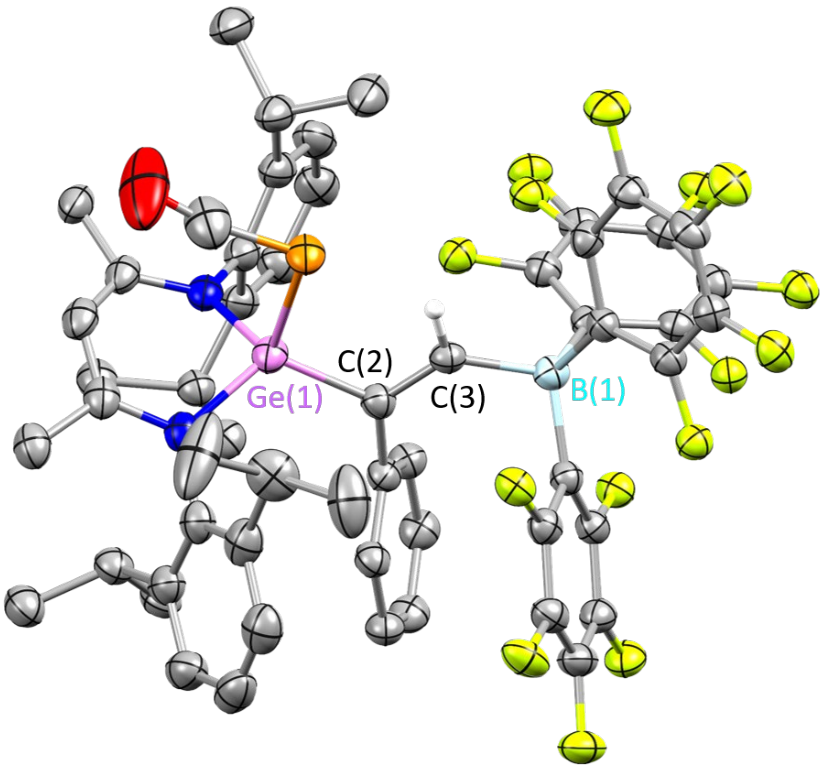

Figure S7. Thermal ellipsoid plot for **8** with the anisotropic displacement parameters depicted at the 30% probability level. Disorder and hydrogen atoms are omitted for clarity.

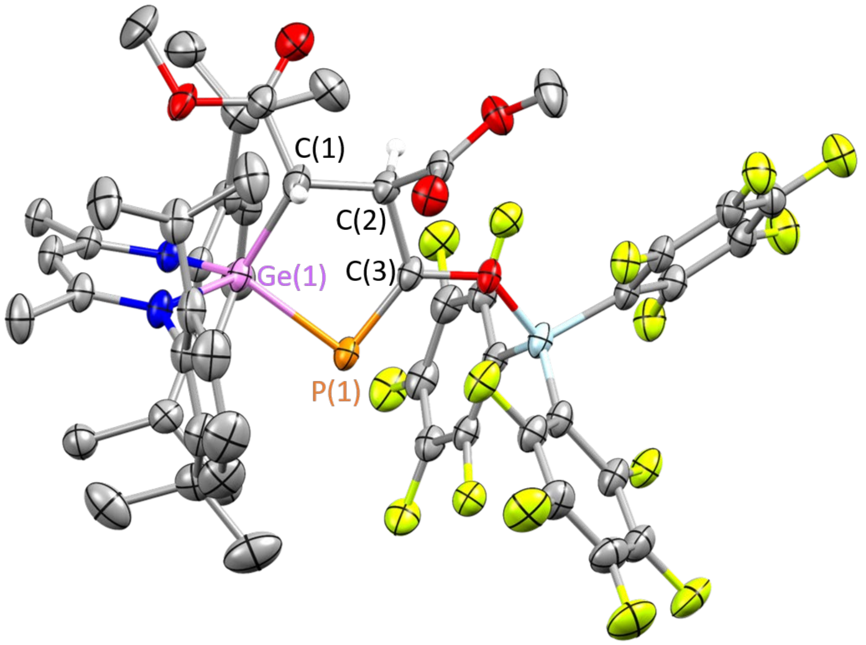

Figure S8. Thermal ellipsoid plot for **9** with the anisotropic displacement parameters depicted at the 30% probability level. The hydrogen atoms except for those on C(1) and C(2) are omitted for clarity.

NMR Spectra

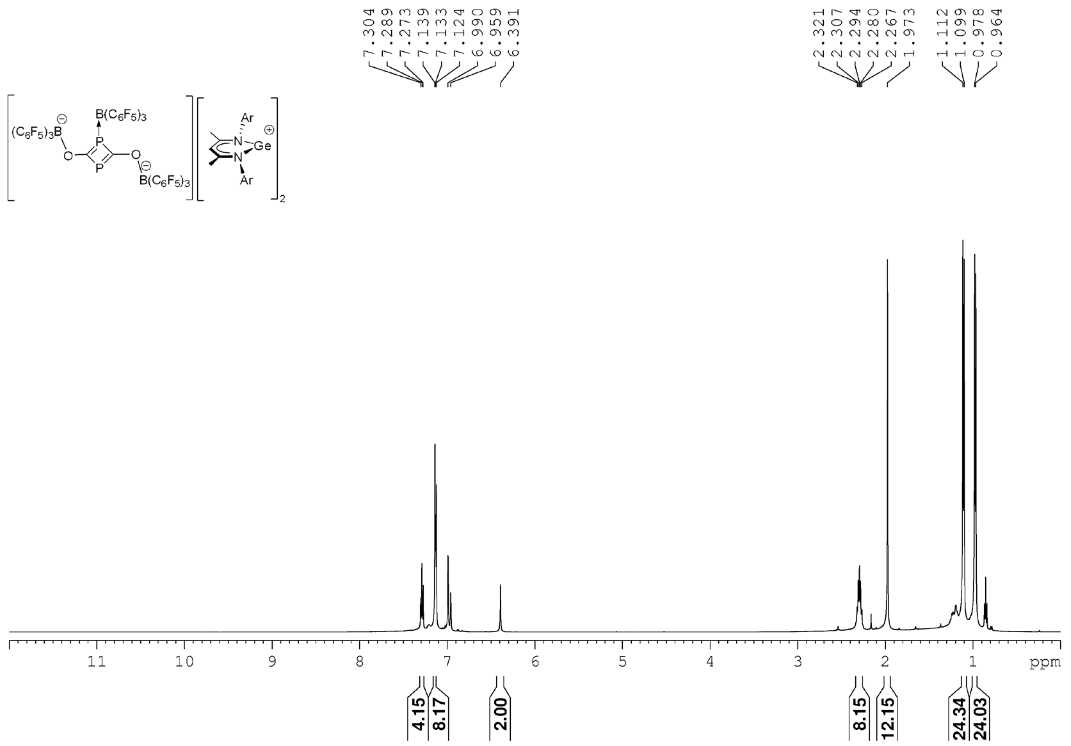

Figure S9. ^{1}H NMR spectrum of **2** in C_6D_5Cl

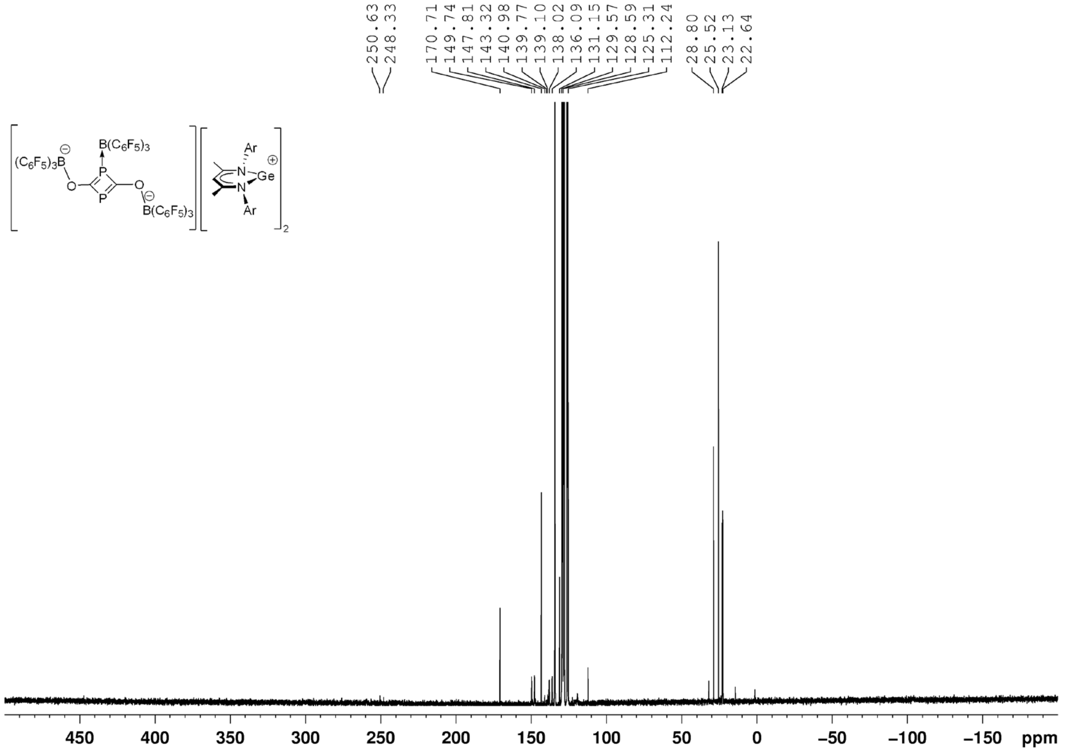

Figure S10. ^{13}C NMR spectrum of **2** in C_6D_5Cl

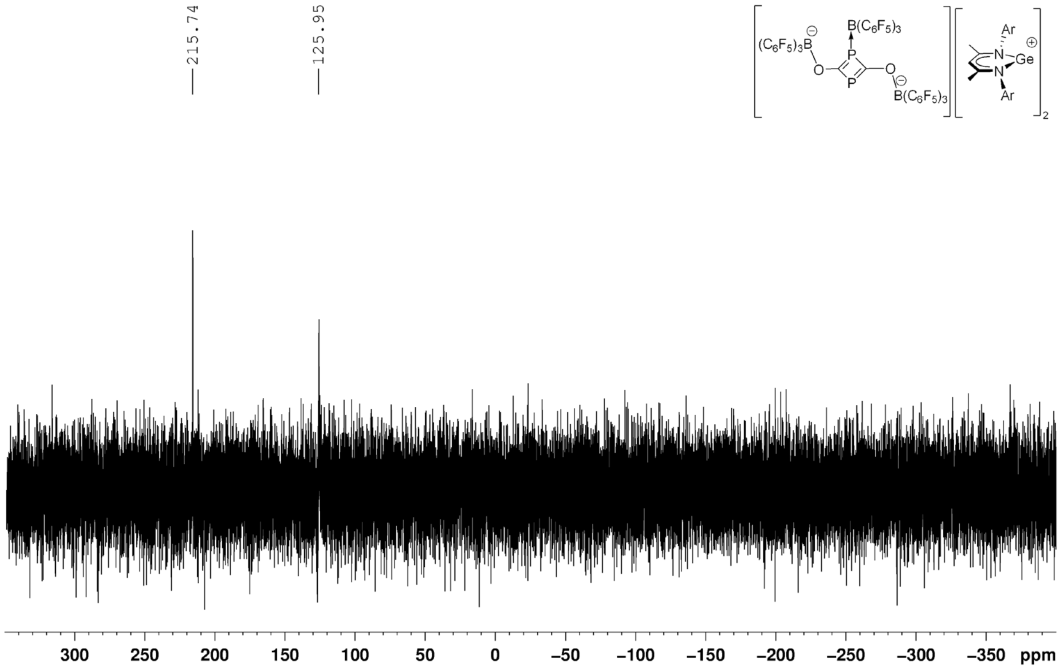

Figure S11. ^{31}P NMR spectrum of **2** in C_6D_5Cl

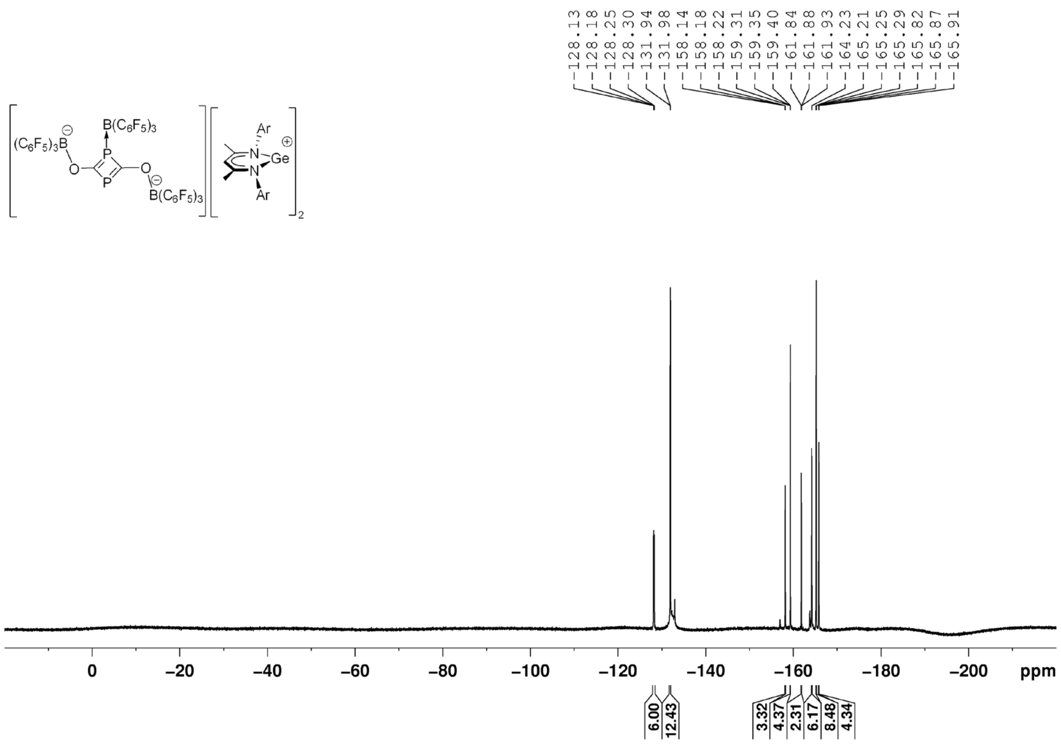

Figure S12. ^{19}F NMR spectrum of **2** in C_6D_5Cl

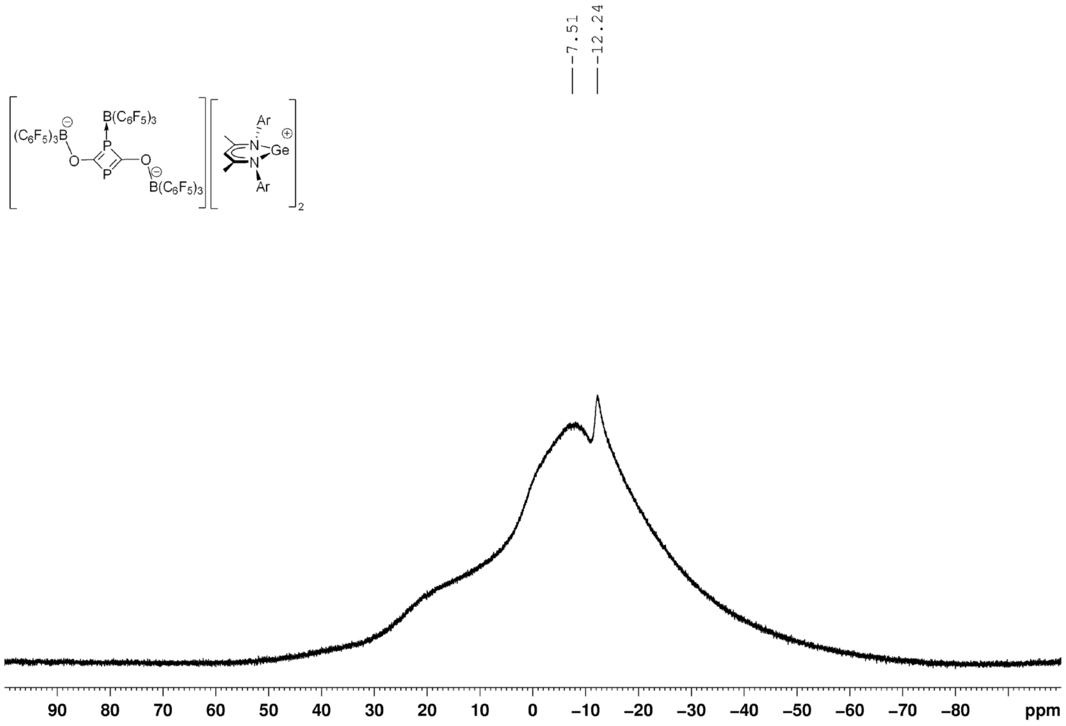

Figure S13. ^{11}B NMR spectrum of **2** in C_6D_5Cl

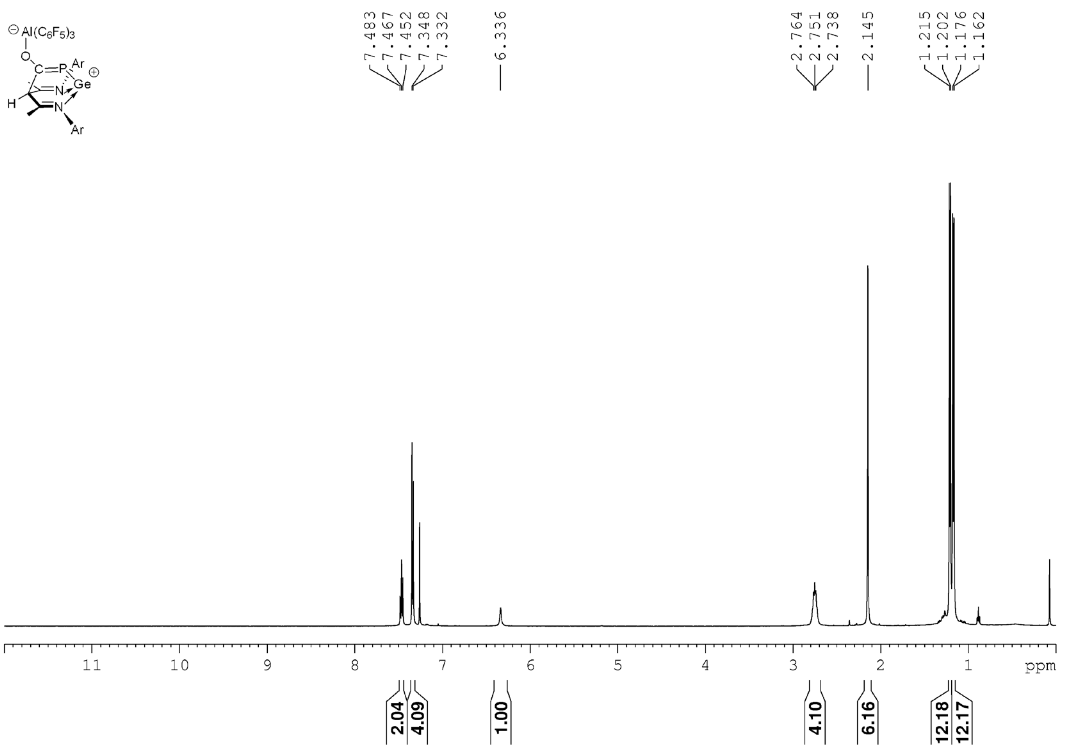

Figure S14. ^{1}H NMR spectrum of **3** in $CDCl_3$

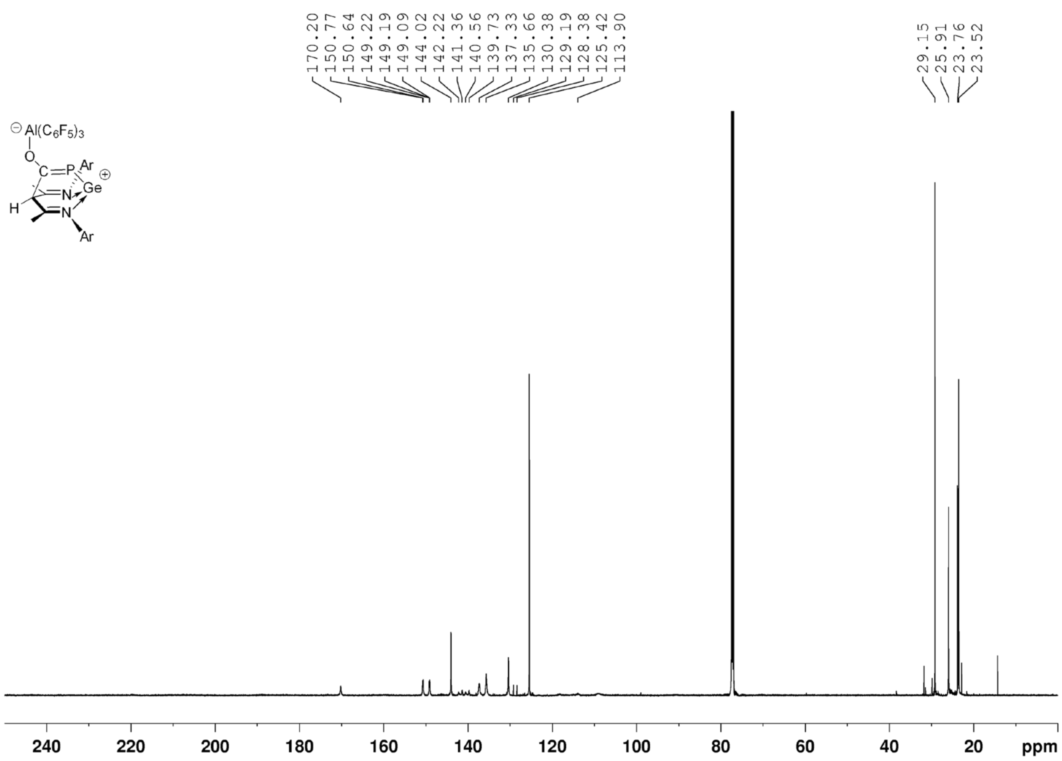

Figure S15. ^{13}C NMR spectrum of **3** in $CDCl_3$

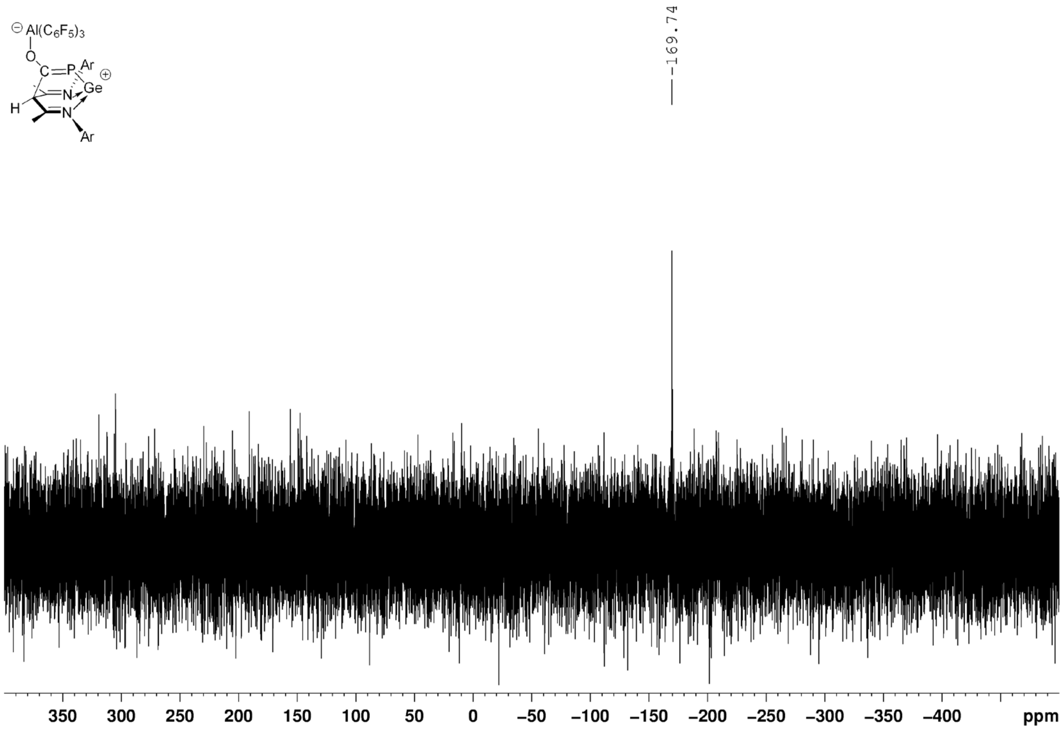

Figure S16. ^{31}P NMR spectrum of **3** in $CDCl_3$

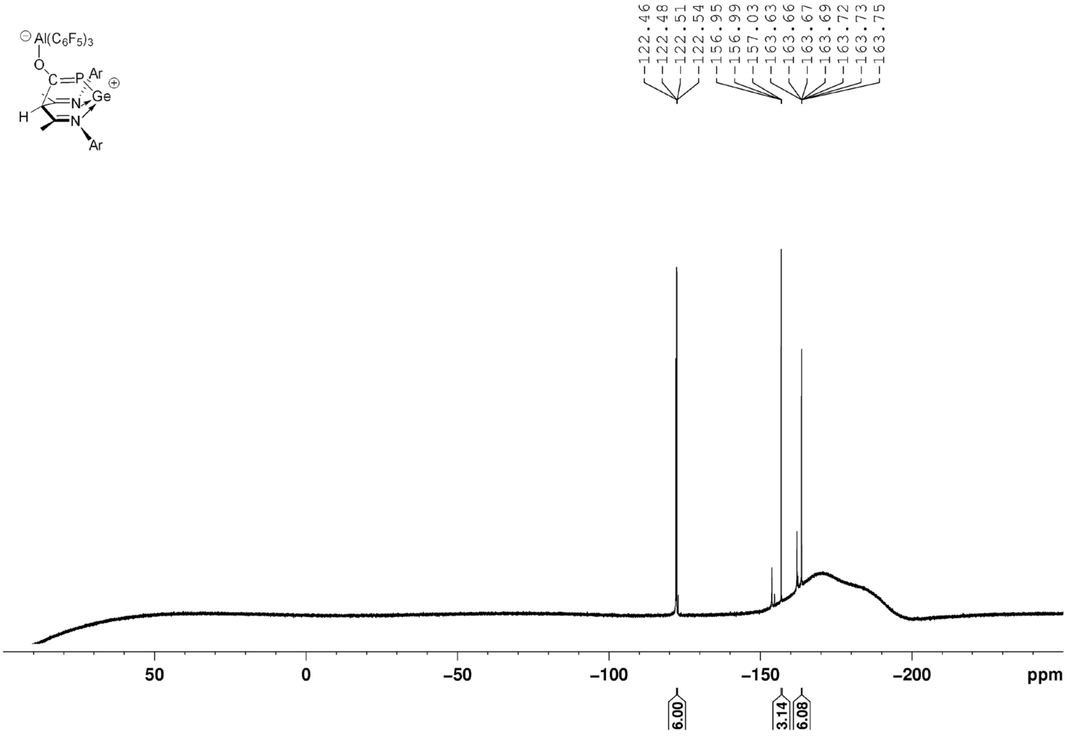

Figure S17. ^{19}F NMR spectrum of **3** in $CDCl_3$

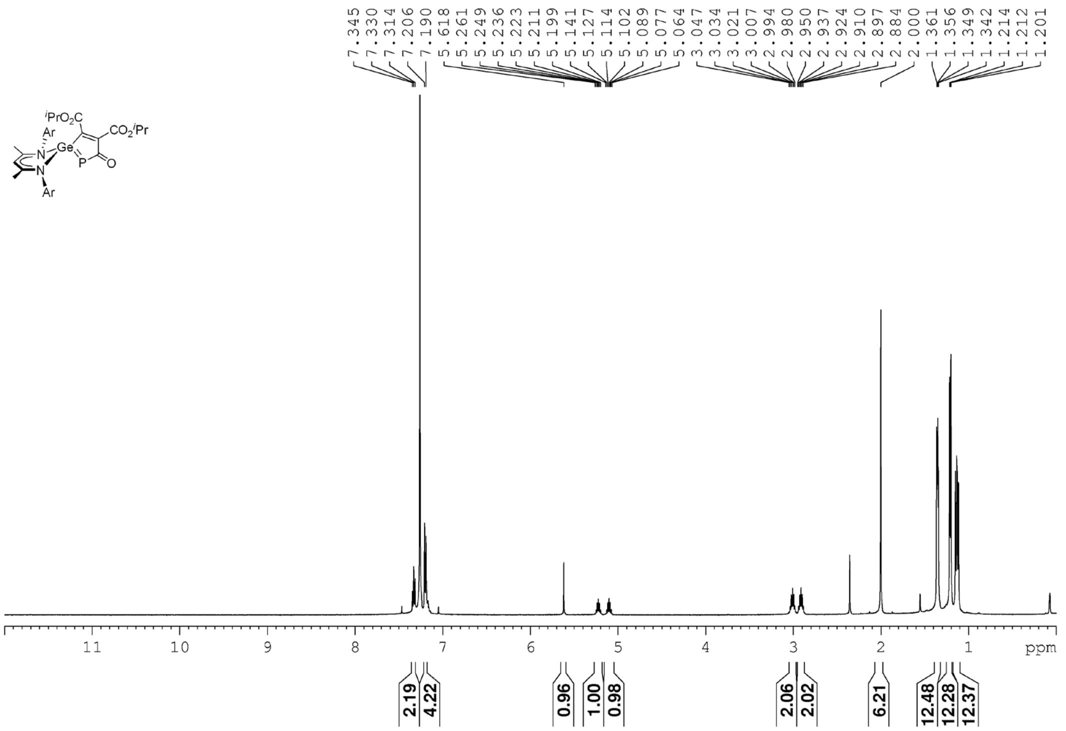

Figure S18. ^{1}H NMR spectrum of **4** in $CDCl_3$

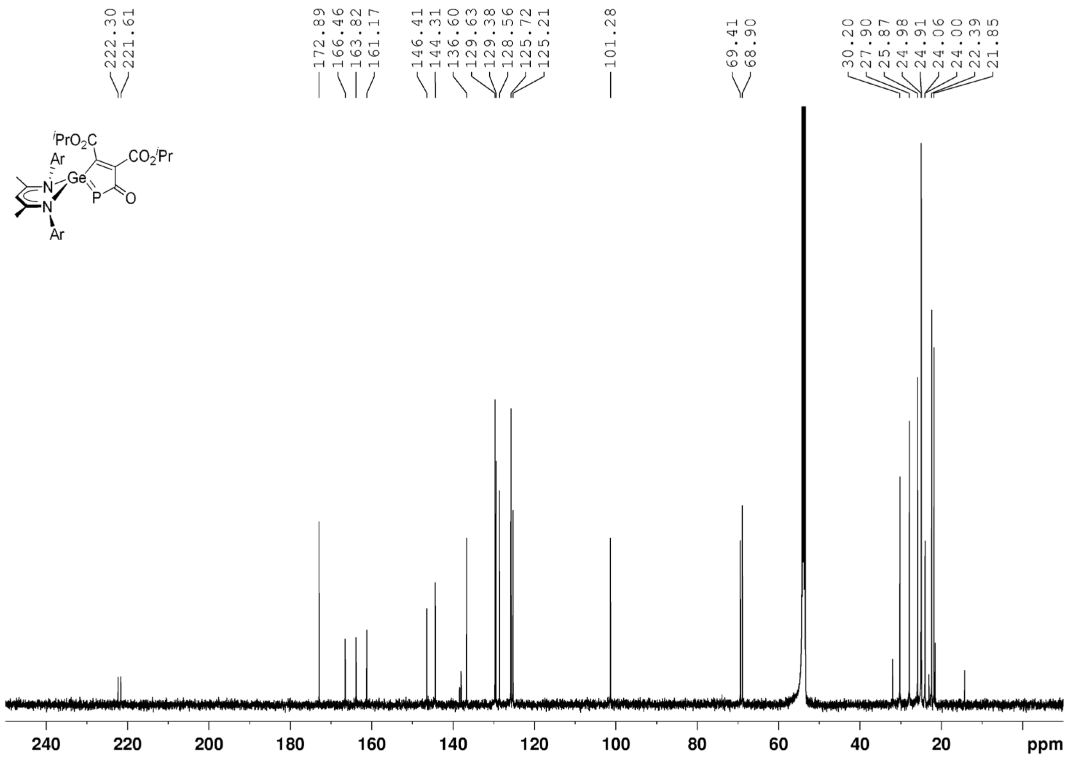

Figure S19. ^{13}C NMR spectrum of **4** in CD_2Cl_2

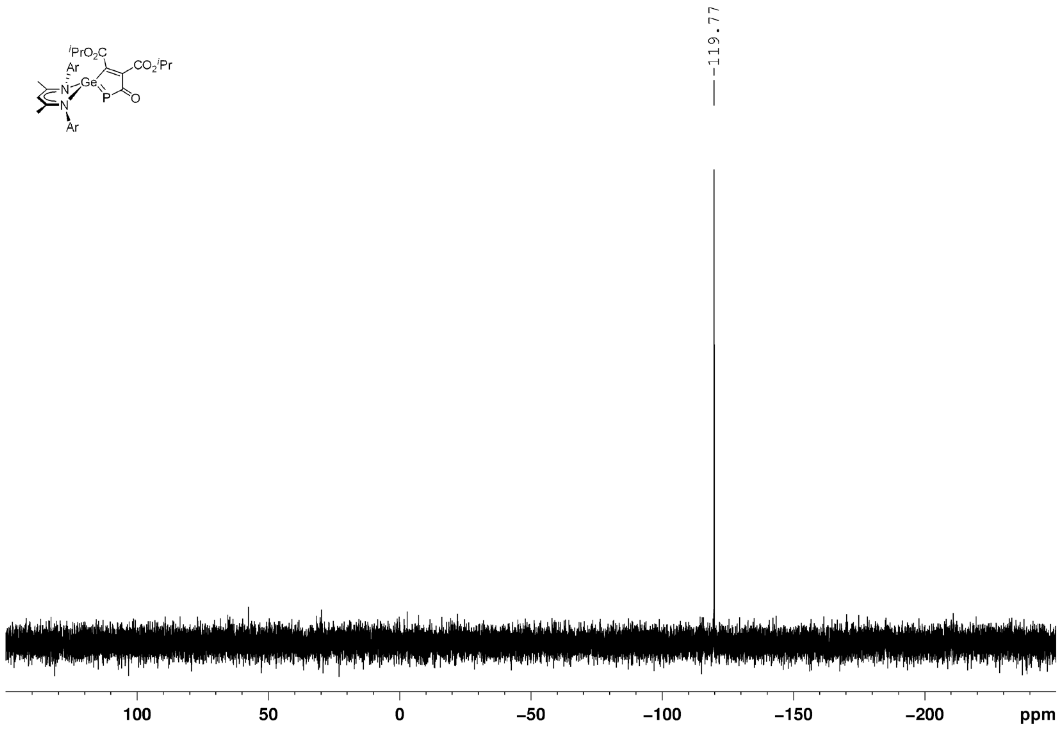

Figure S20. ^{31}P NMR spectrum of **4** in $CDCl_3$

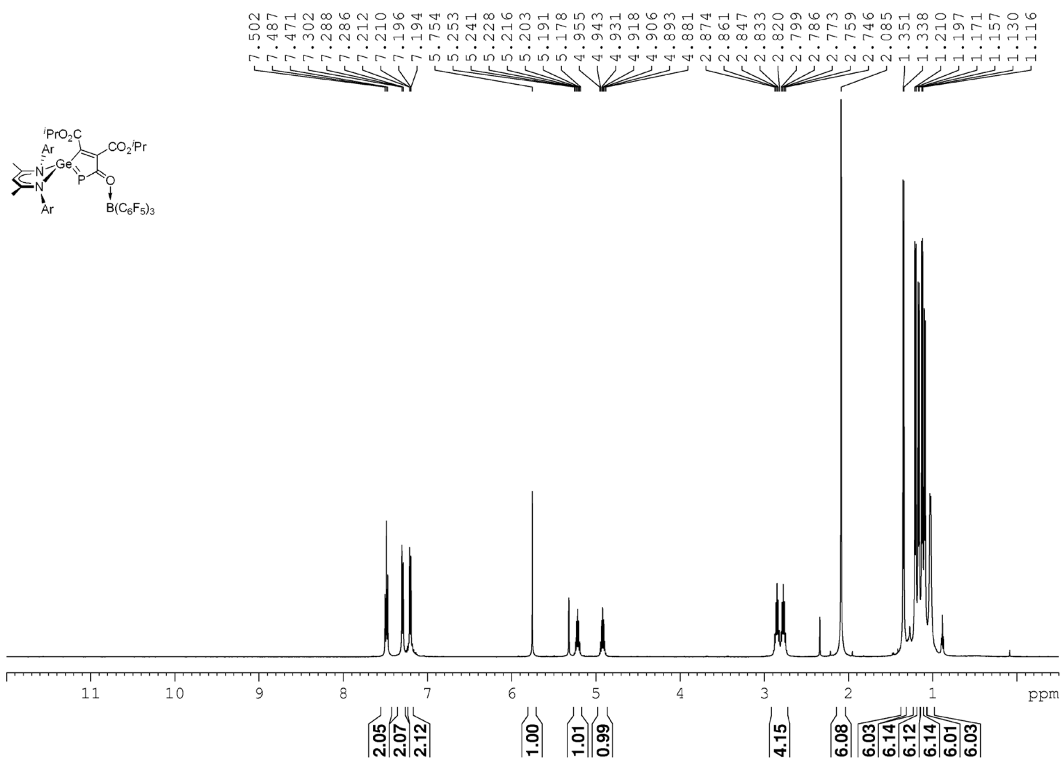

Figure S21. ^{1}H NMR spectrum of **5** in CD_2Cl_2

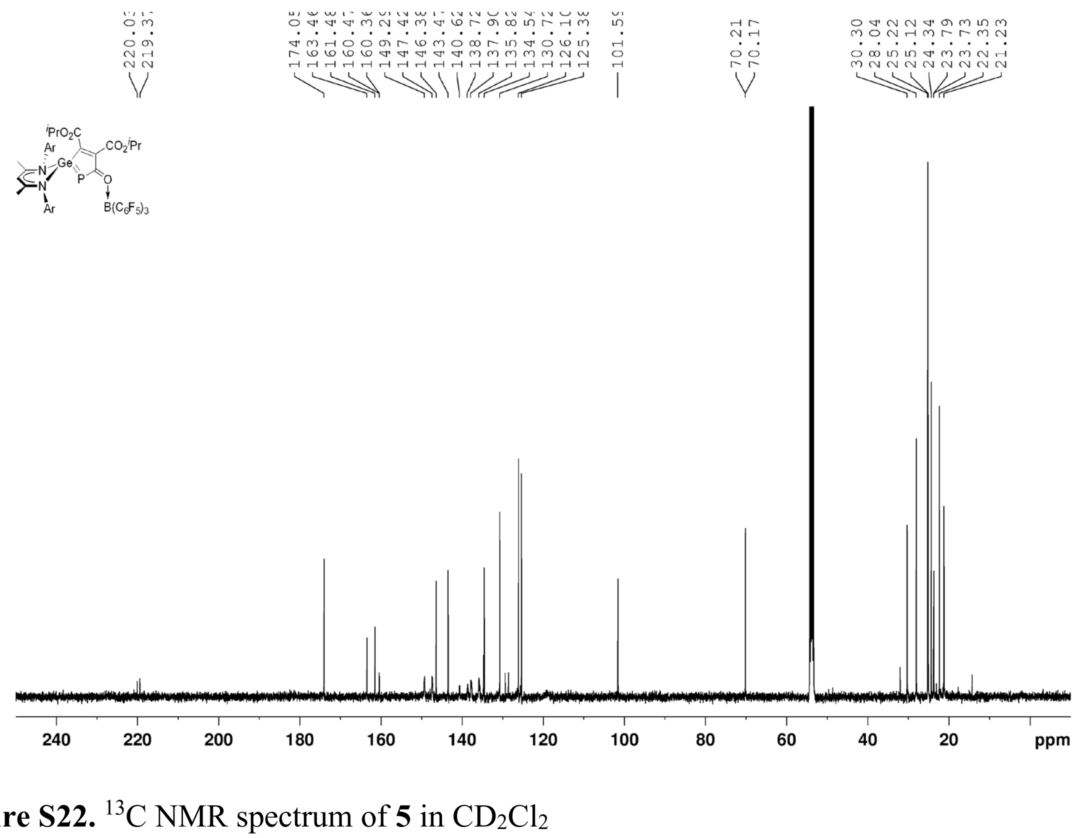

Figure S22. ^{13}C NMR spectrum of **5** in CD_2Cl_2

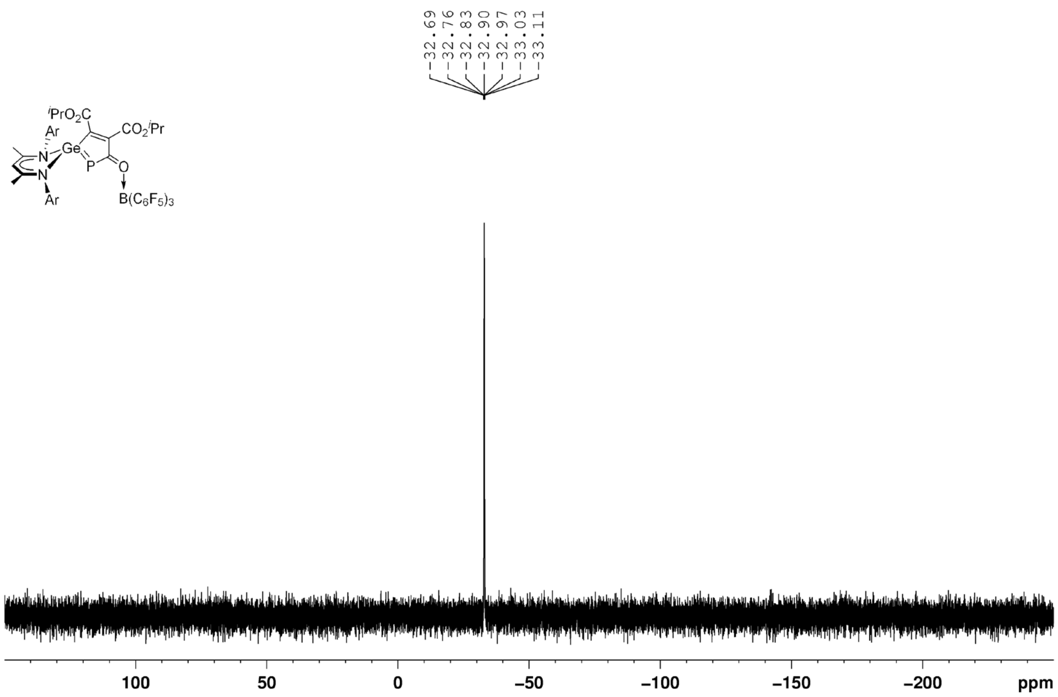

Figure S23. ^{31}P NMR spectrum of **5** in CD_2Cl_2

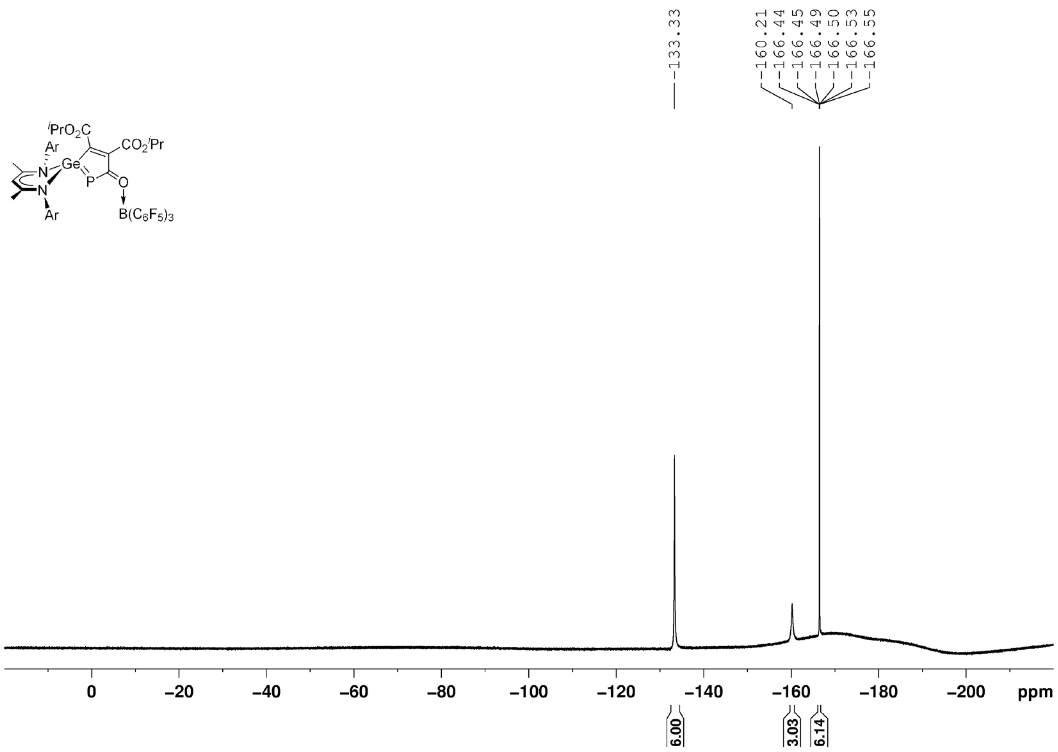

Figure S24. ^{19}F NMR spectrum of **5** in CD_2Cl_2

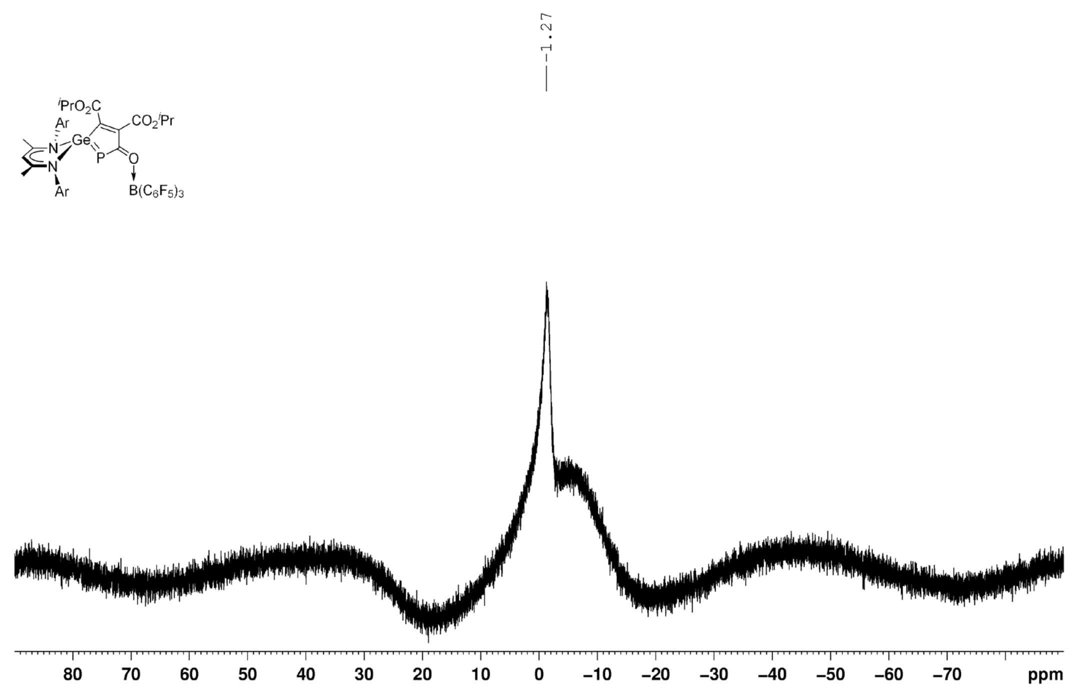

Figure S25. ^{11}B NMR spectrum of **5** in CD_2Cl_2

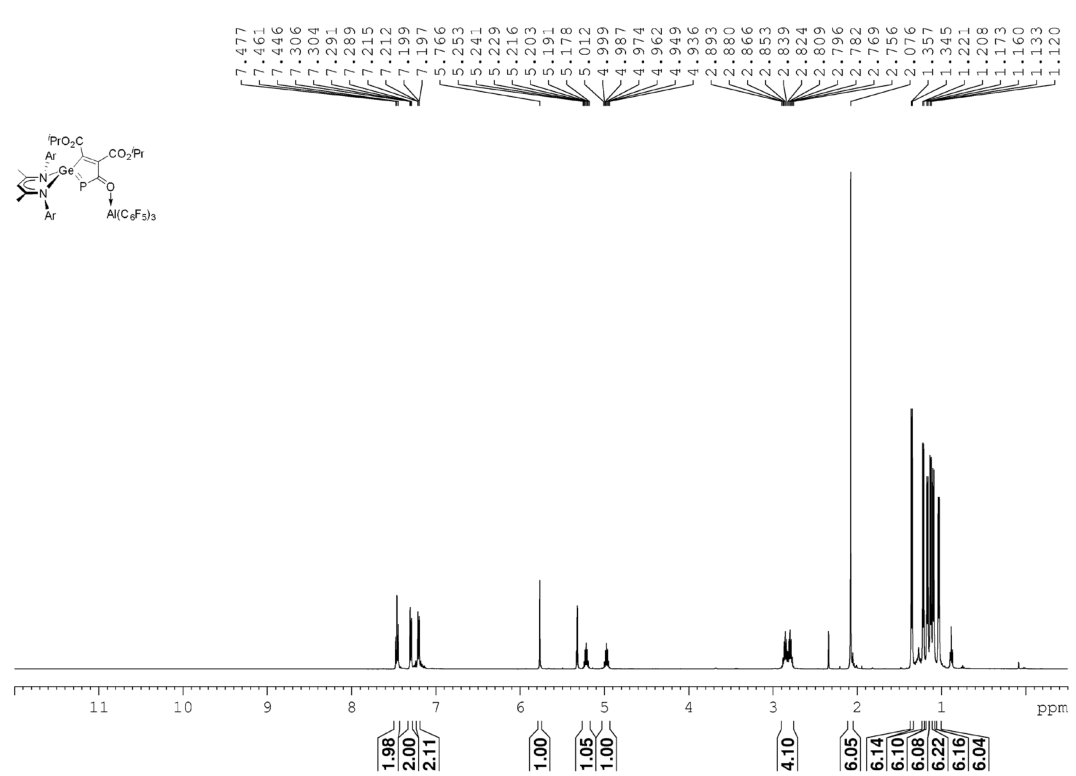

Figure S26. ^{1}H NMR spectrum of **6** in CD_2Cl_2

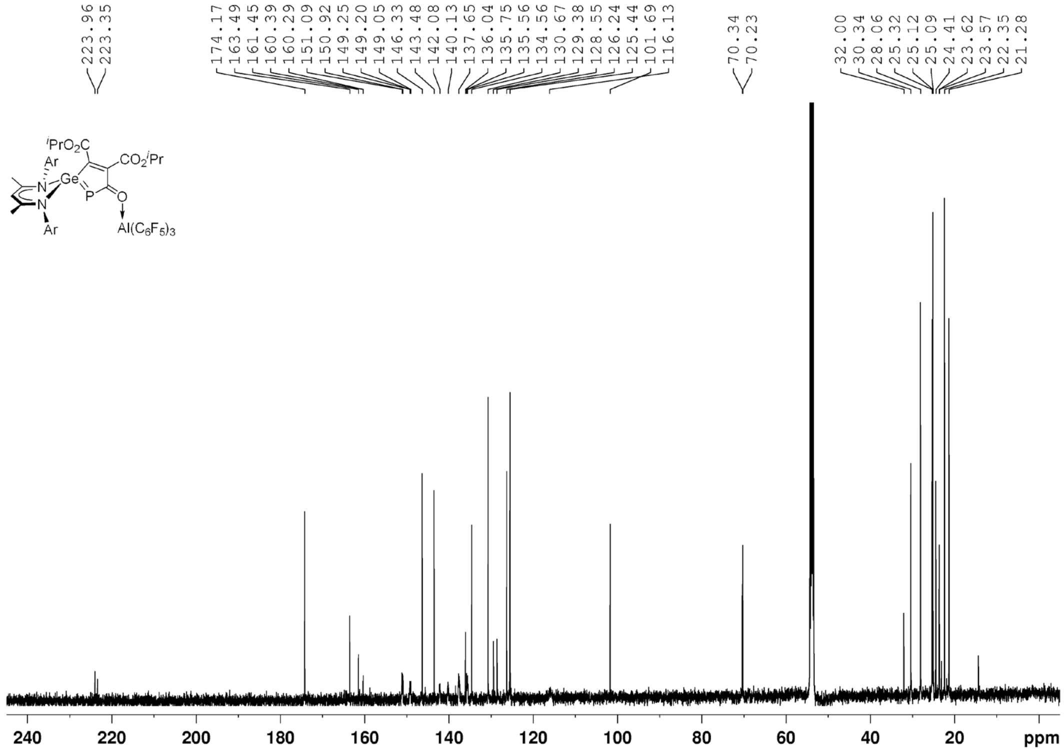

Figure S27. ^{13}C NMR spectrum of **6** in CD_2Cl_2

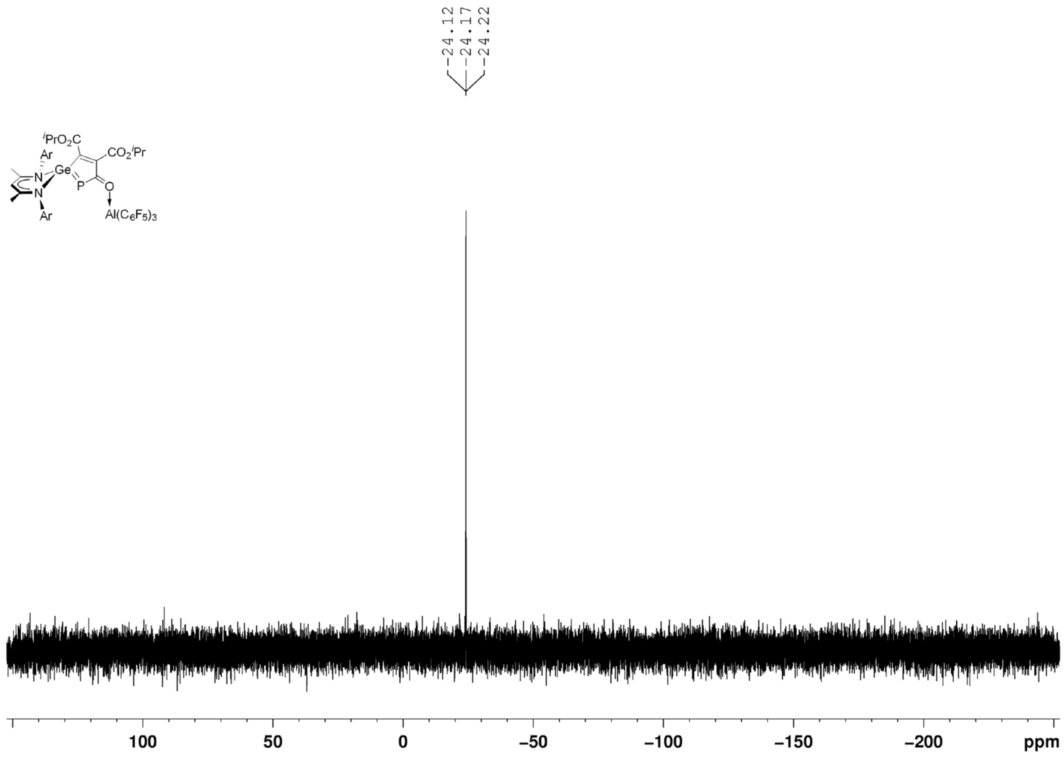

Figure S28. ^{31}P NMR spectrum of **6** in CD_2Cl_2

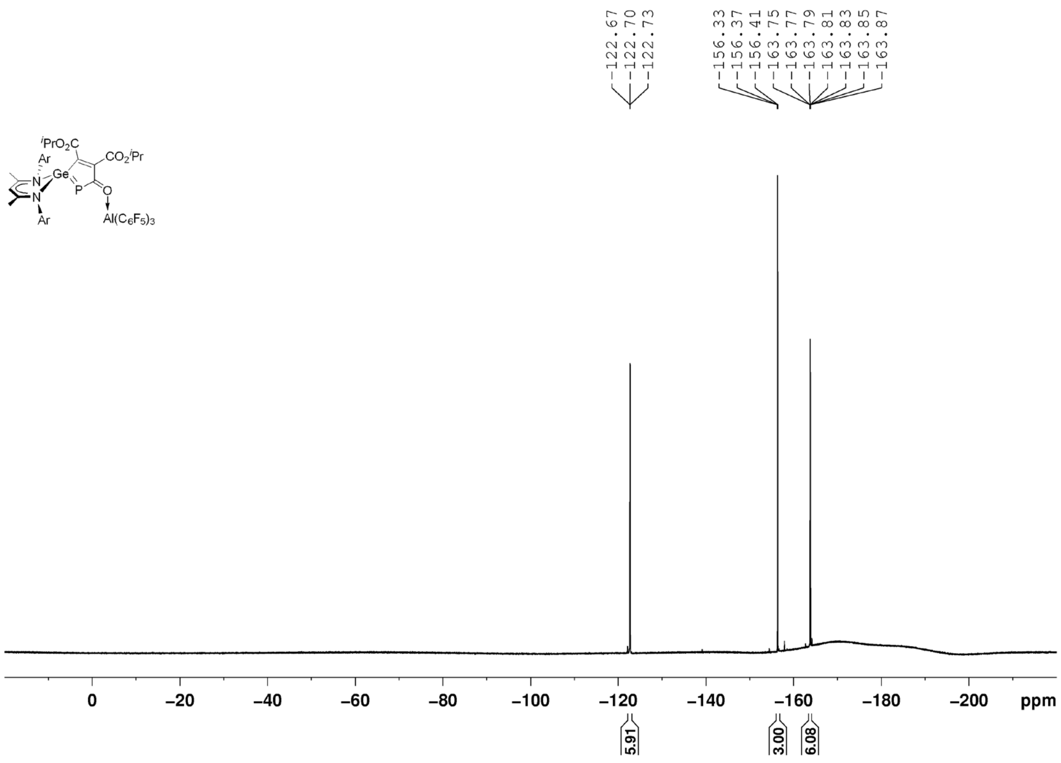

Figure S29. ^{19}F NMR spectrum of **6** in CD_2Cl_2

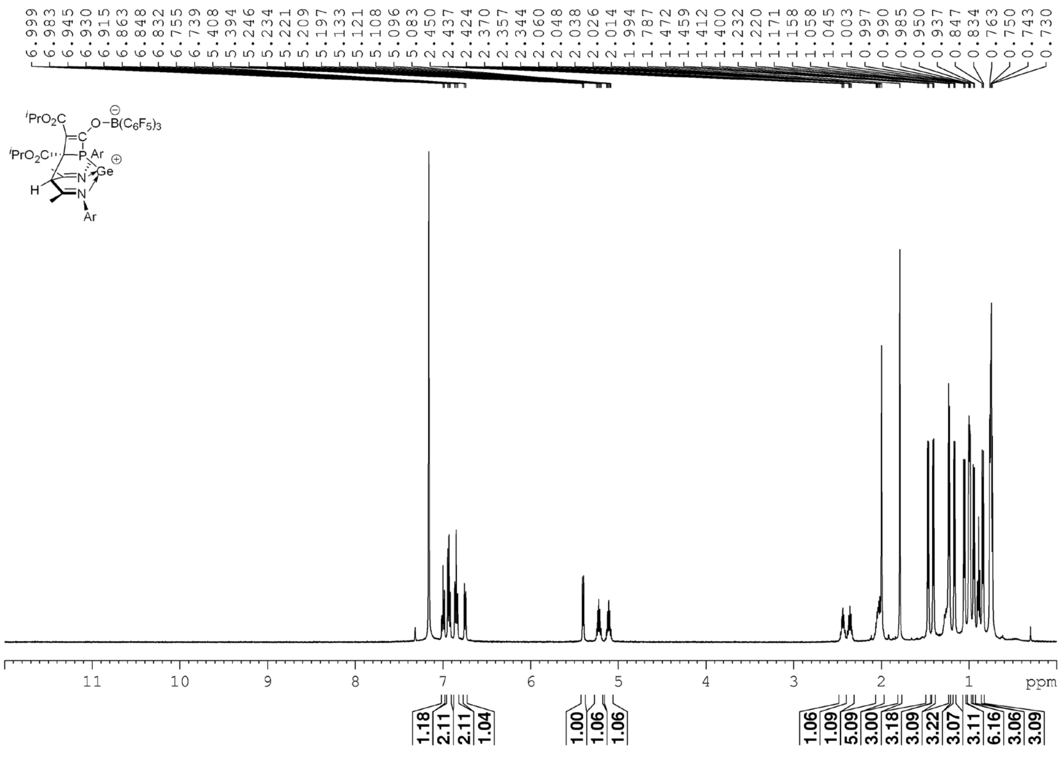

Figure S30. ^{1}H NMR spectrum of **7** in C_6D_6

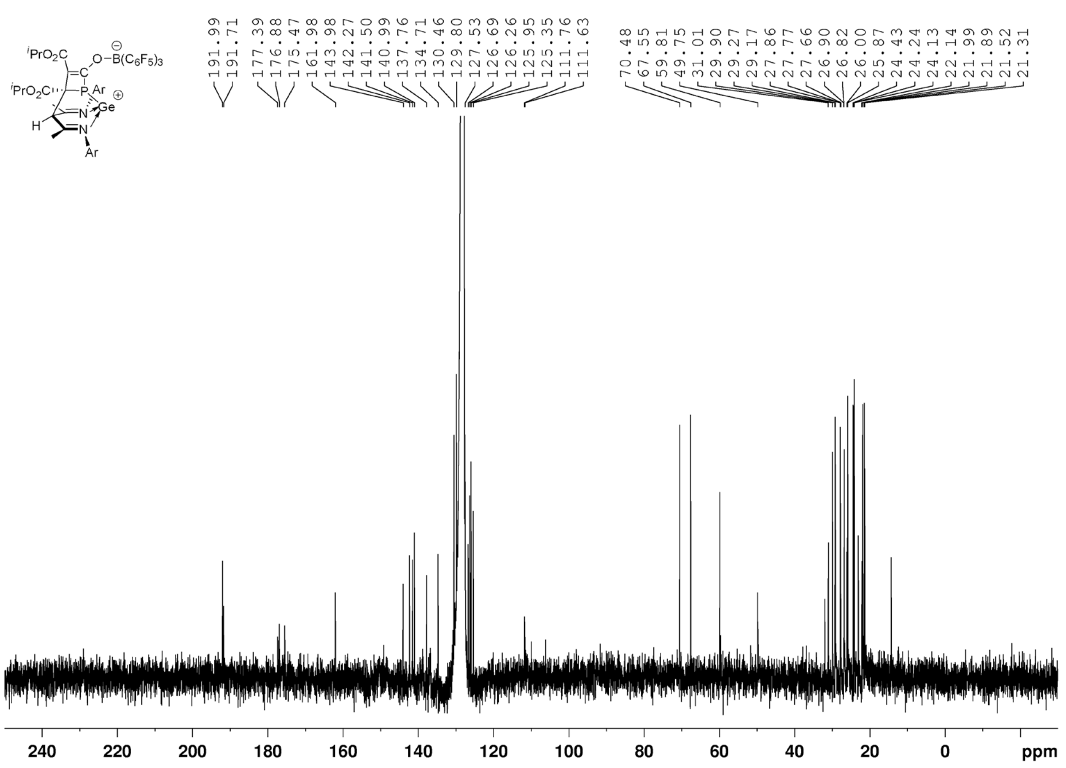

Figure S31. ^{13}C NMR spectrum of **7** in C_6D_6

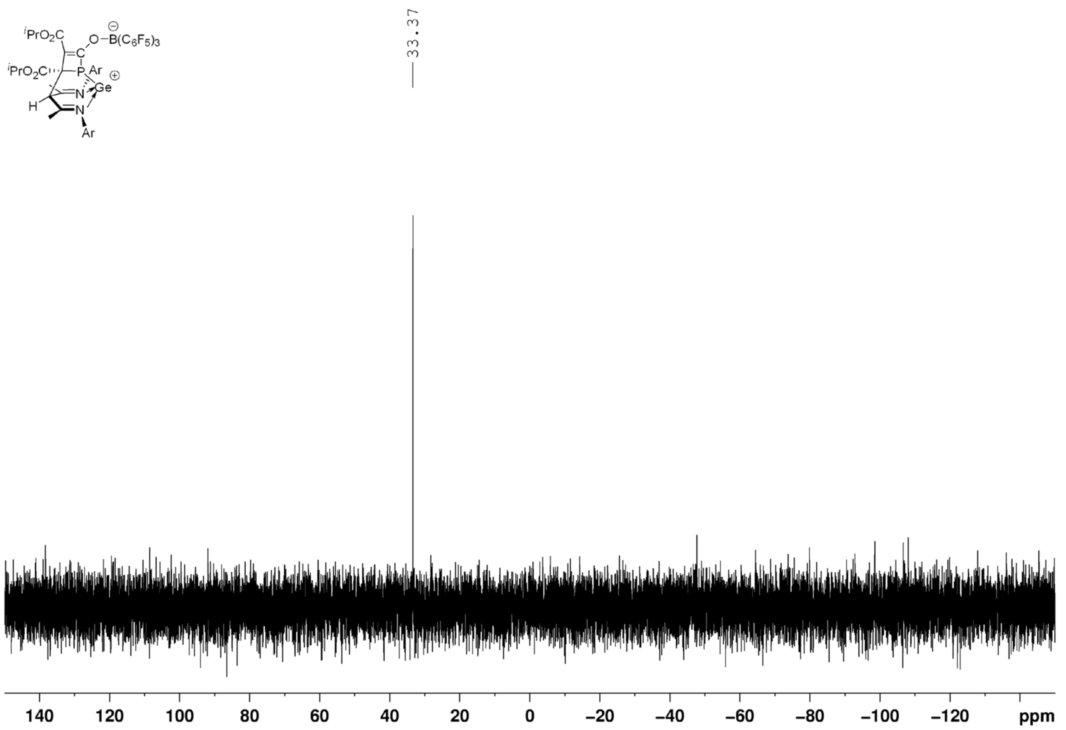

Figure S32. ^{31}P NMR spectrum of **7** in C_6D_6

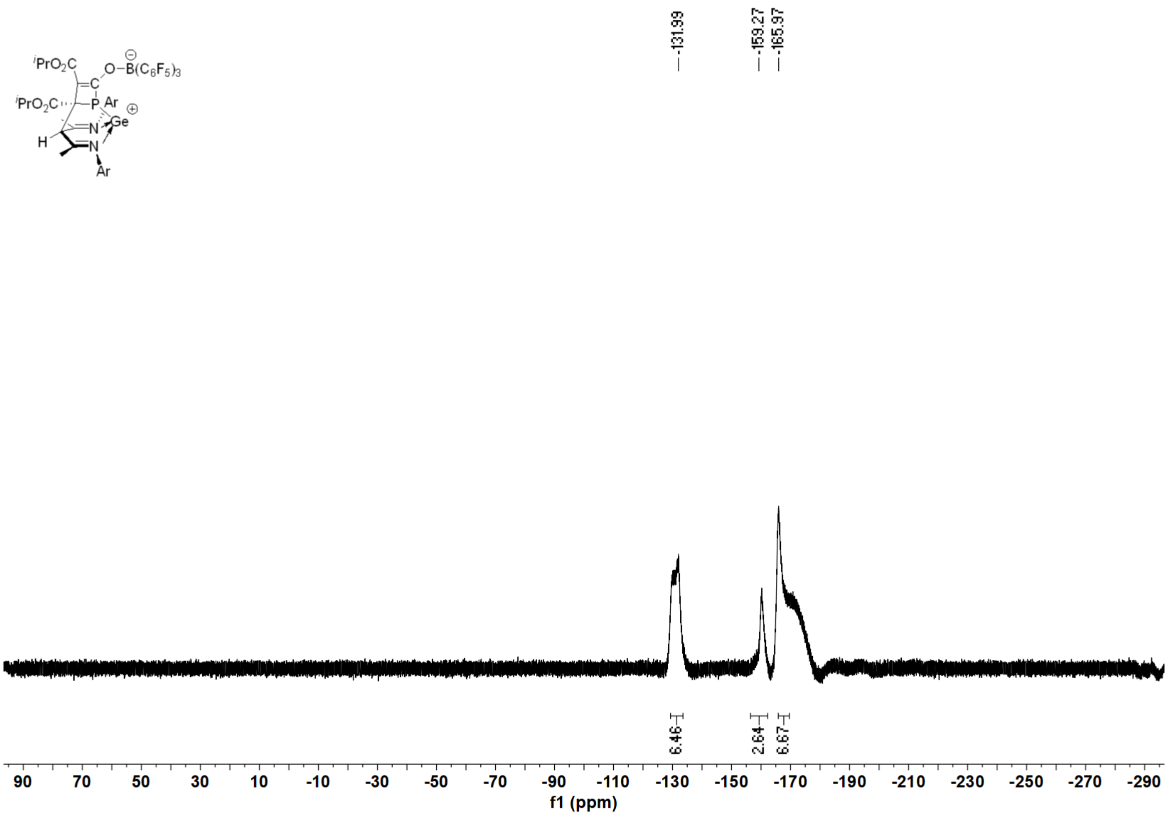

Figure S33. ^{19}F NMR spectrum of **7** in C_6D_6

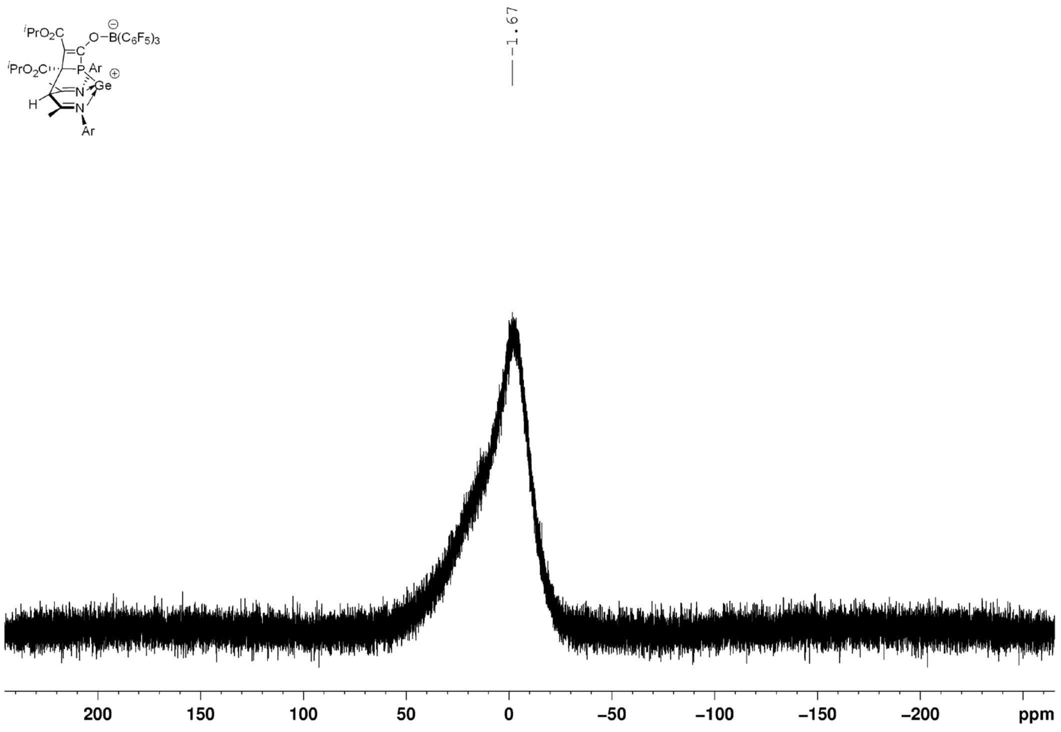

Figure S34. ^{11}B NMR spectrum of **7** in C_6D_6

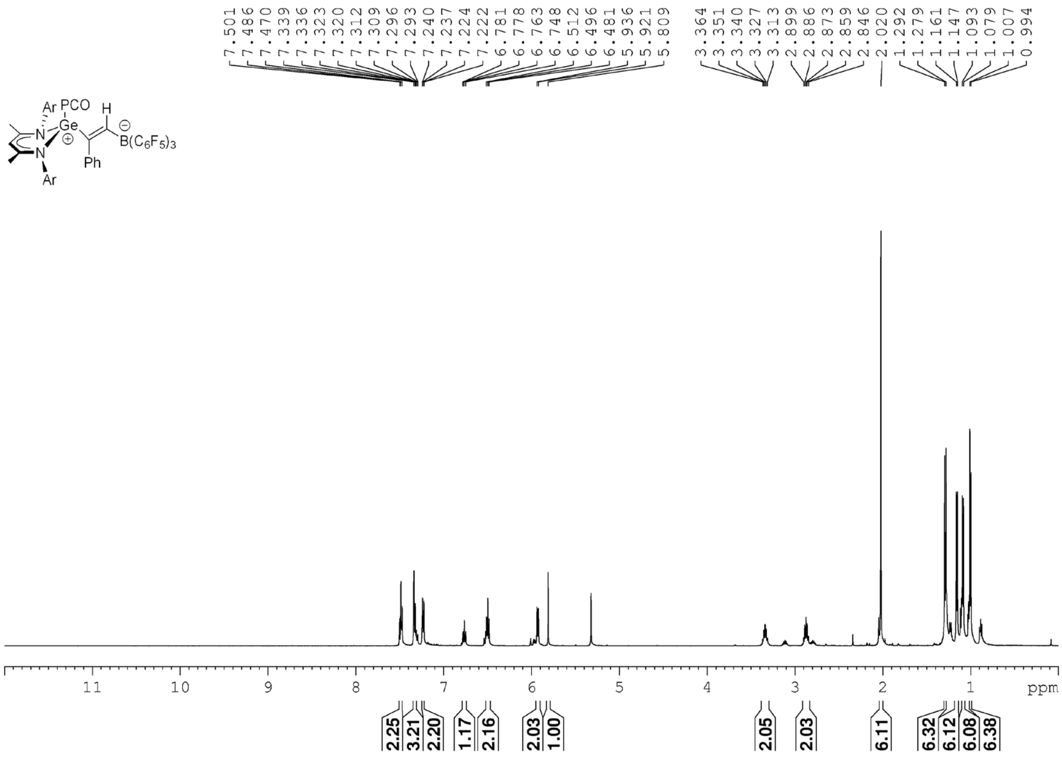

Figure S35. ^{1}H NMR spectrum of **8** in CD_2Cl_2

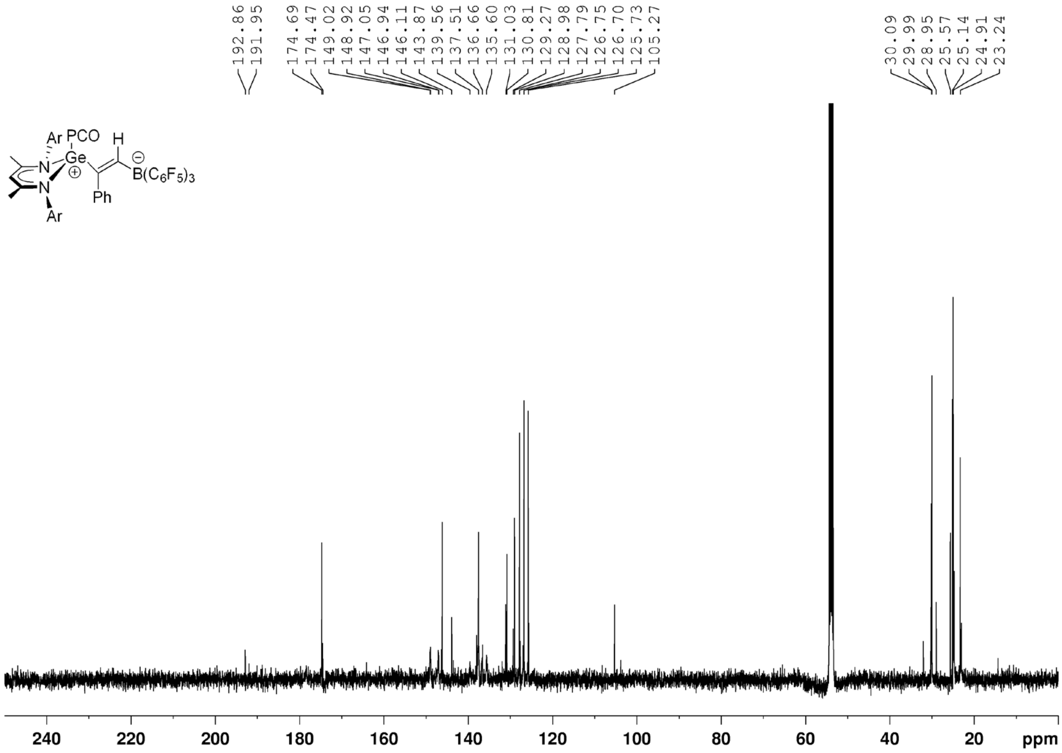

Figure S36. ^{13}C NMR spectrum of **8** in CD_2Cl_2

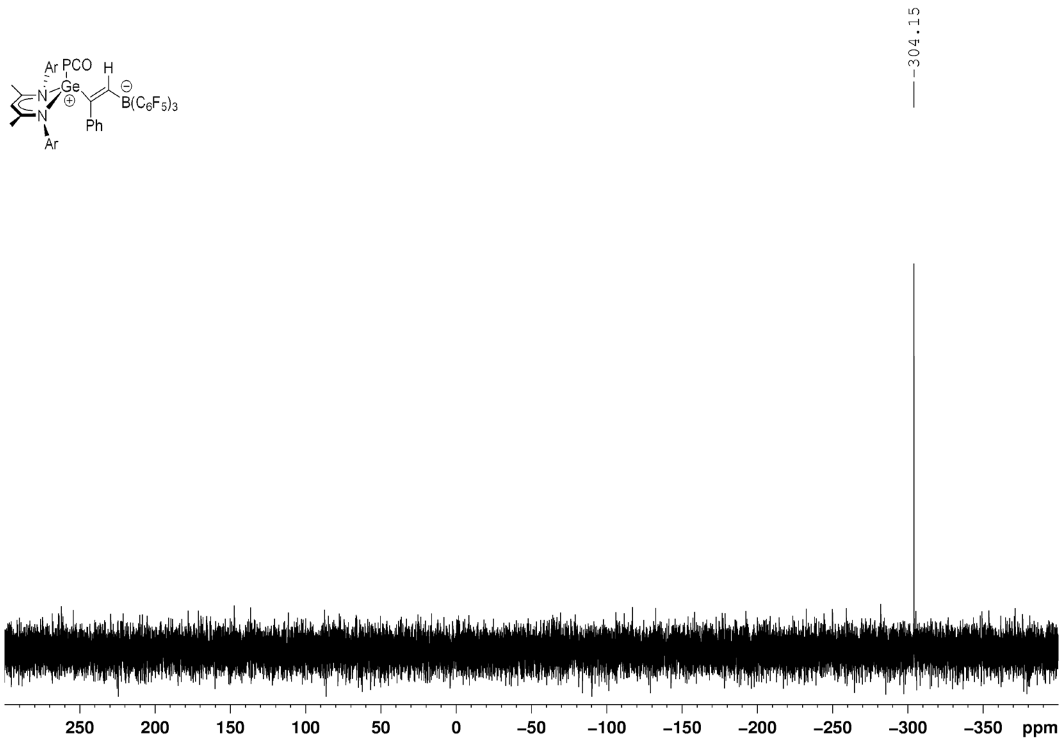

Figure S37. ^{31}P NMR spectrum of **8** in CD_2Cl_2

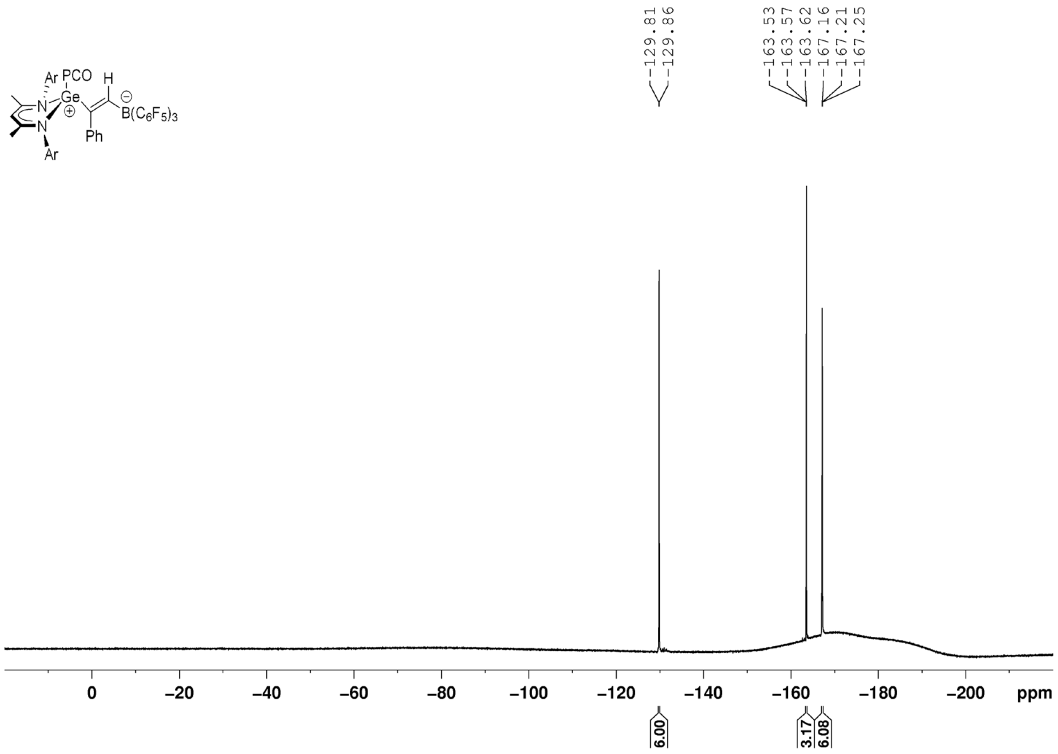

Figure S38. ^{19}F NMR spectrum of **8** in CD_2Cl_2

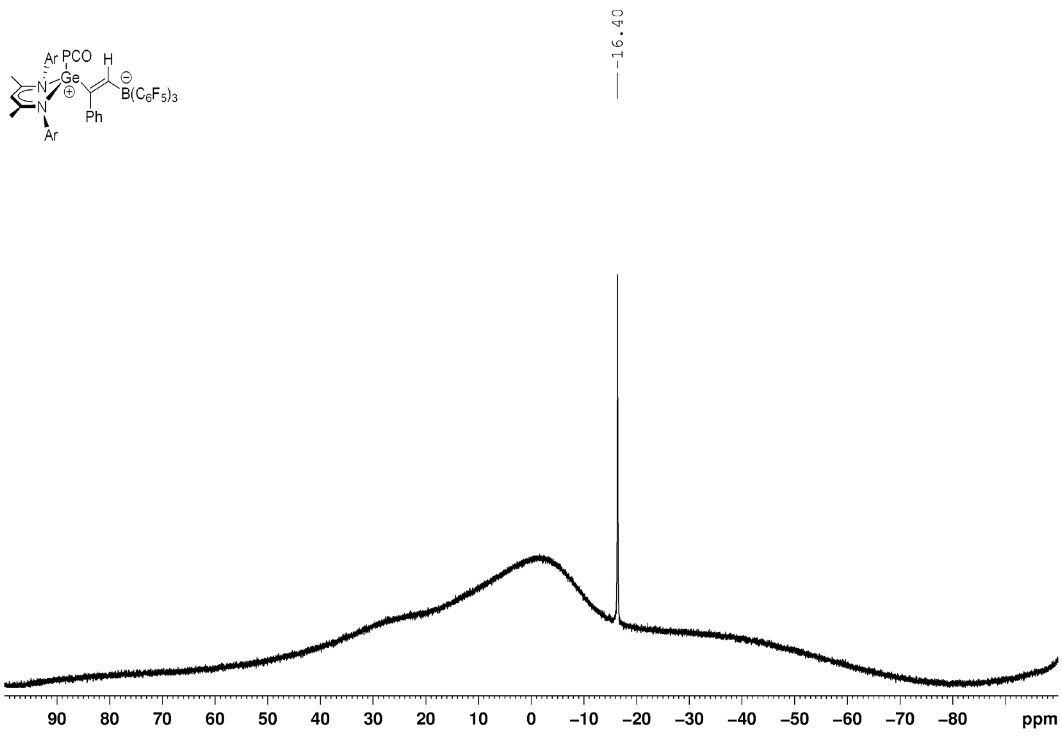

Figure S39. ^{11}B NMR spectrum of **8** in C_6D_6

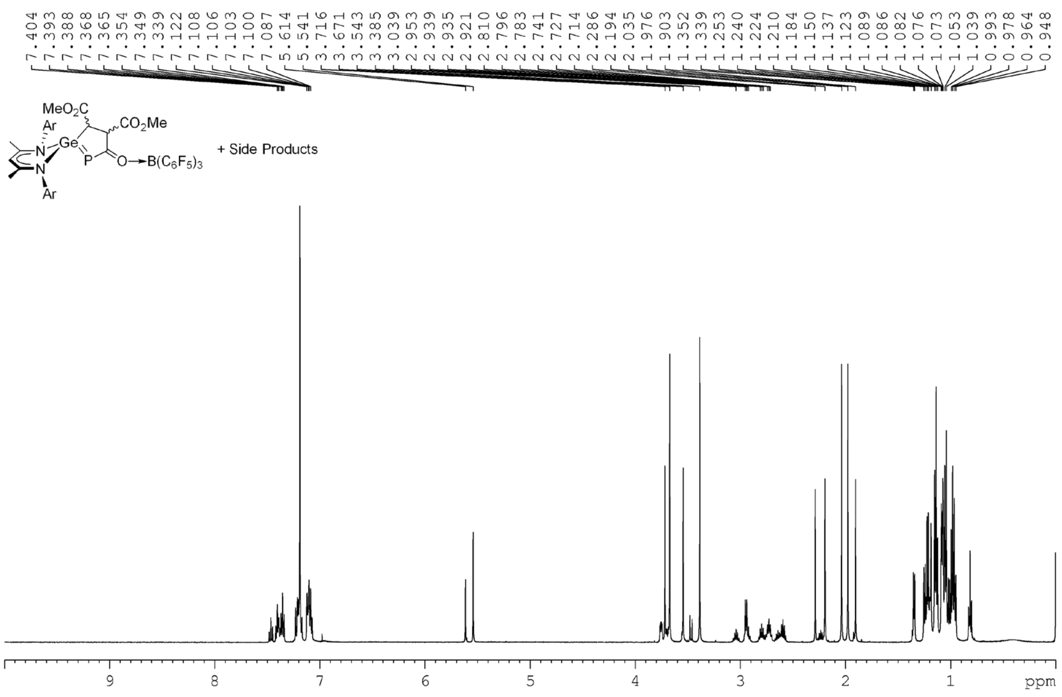

Figure S40. ^{1}H NMR spectrum of **9** and side products in $CDCl_3$

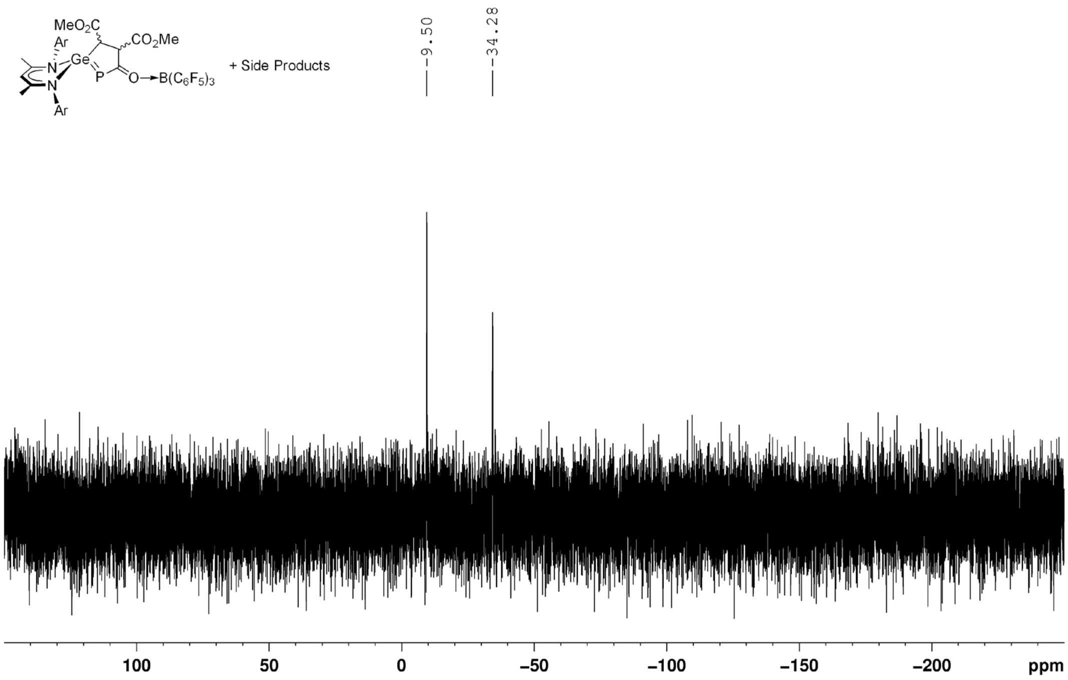

Figure S41. ^{31}P NMR spectrum of **9** and side products in $CDCl_3$

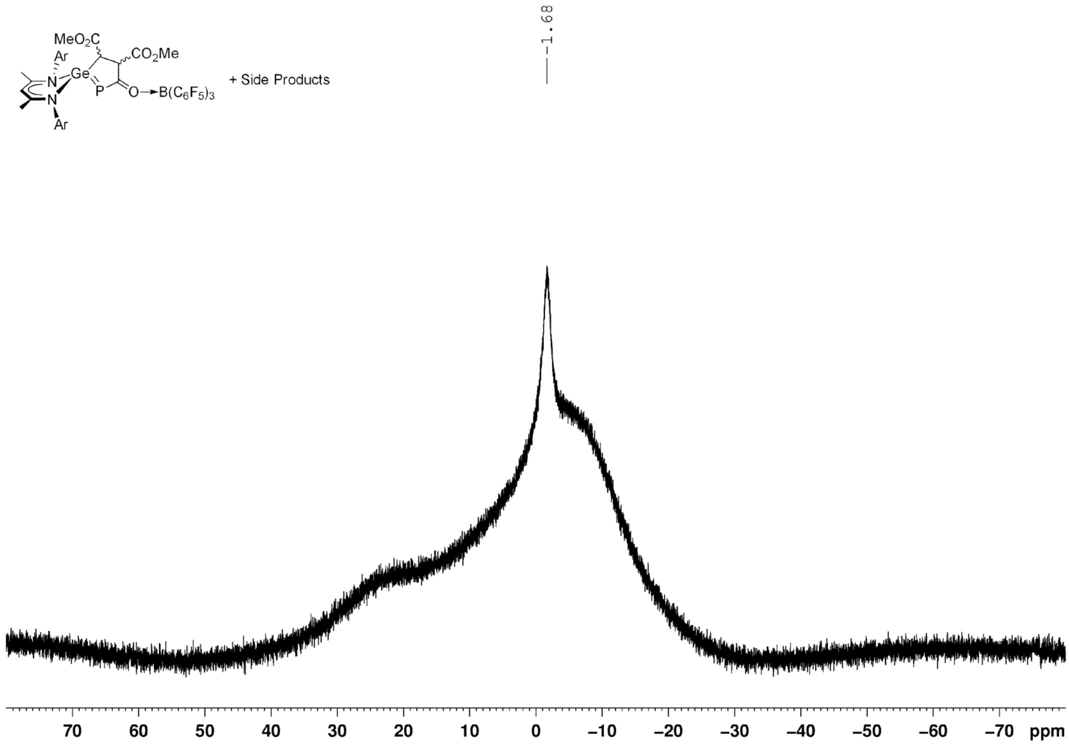

Figure S42. ^{11}B NMR spectrum of **9** and side products in $CDCl_3$

DFT Computational Details:

The quantum chemical DFT calculations have been performed with the TURBOMOLE 7.4 suite of programs[1] The structures are fully optimized at the TPSS-D3/def2-TZVP + COSMO level of theory, which combines the TPSS meta-GGA density functional[2] with the BJ-damped DFT-D3 dispersion correction[3] and the def2-TZVP basis set,[4] using the Conductor-like Screening Model (COSMO) continuum solvation model[5] for toluene solvent (dielectric constant ε = 2.38 and solvent diameter R_{solv} = 3.48 Å).. The density-fitting RI-J approach[4a, 6] is used to accelerate the geometry optimization and numerical harmonic frequency calculations[7] in solution. The optimized structures are characterized by frequency analysis to identify the nature of located stationary points (no imaginary frequency for true minima and only one imaginary frequency for transition state) and to provide thermal corrections (at 298.15 K and 1 atm) according to the modified ideal gas–rigid rotor–harmonic oscillator model.[8] This choice of dispersion-corrected meta-GGA functional makes the efficient exploration of all potential reaction paths possible.

The final solvation free energies in toluene are computed with the COSMO-RS solvation model[9] (parameter file: BP_TZVP_C30_1601.ctd) using the COSMOtherm program package[[10] on the above TPSS-D3 optimized structures, and corrected by +1.89 kcal·mol^{-1} to account for higher reference solute concentration of 1 mol·L^{-1} usually used in solution. To check the effects of the chosen DFT functional on the reaction energies and barriers, single-point calculations at the meta-GGA TPSS-D3[2] and hybrid-meta-GGA PW6B95-D3[11] levels are performed using a larger def2-QZVP basis set.[4b, 12] The final reaction Gibbs free energies (ΔG) are determined from the electronic single-point energies plus TPSS-D3 thermal corrections and COSMO-RS solvation free energies. The computed relative free energies from both DFT functionals are mostly in good mutual agreement of –0.2 ± 2.7 kcal/mol (average and standard deviations, see Table S1 below), but the barriers at PW6B95-D3 level are usually 4.3 ± 2.5 kcal/mol higher as expected. In our discussion, higher-level PW6B95-D3 Gibbs free energies (in kcal/mol, at 298.15 K and 1 mol/L concentration) will be used in our discussion unless specified otherwise. The applied DFT methods in combination with the large AO basis set provide usually accurate electronic energies leading to errors for chemical energies (including barriers) on the order of typically 1-2 kcal/mol. This has been tested thoroughly for the huge data base GMTKN55[13] which is the common standard in the field of DFT benchmarking.

References

[1] *TURBOMOLE V7.4*, **2019**, a development of University of Karlsruhe and Forschungszentrum Karlsruhe GmbH, 1989-2007, TURBOMOLE GmbH, since 2007; available from http://www.turbomole.com.

[2] J. Tao, J. P. Perdew, V. N. Staroverov, G. E. Scuseria, *Physical Review Letters* **2003**, *91*, 146401.

[3] a) S. Grimme, J. Antony, S. Ehrlich, H. Krieg, *The Journal of Chemical Physics* **2010**, *132*, 154104-154119; b) S. Grimme, S. Ehrlich, L. Goerigk, *Journal of Computational Chemistry* **2011**, *32*, 1456-1465.

[4] a) F. Weigend, M. Häser, H. Patzelt, R. Ahlrichs, *Chemical Physics Letters* **1998**, *294*, 143-152; b) F. Weigend, R. Ahlrichs, *Physical Chemistry Chemical Physics* **2005**, *7*, 3297-3305.

[5] A. Klamt, G. Schüürmann, *Journal of the Chemical Society, Perkin Transactions 2* **1993**, 799-805.

[6] a) K. Eichkorn, F. Weigend, O. Treutler, R. Ahlrichs, *Theoretical Chemistry Accounts* **1997**, *97*, 119-124; bF. Weigend, *Physical Chemistry Chemical Physics* **2006**, *8*, 1057-1065.

[7] P. Deglmann, K. May, F. Furche, R. Ahlrichs, *Chemical Physics Letters* **2004**, *384*, 103-107.

[8] S. Grimme, *Chemistry - A European Journal* **2012**, *18*, 9955-9964.

[9] F. Eckert, A. Klamt, *AIChE Journal* **2002**, *48*, 369-385.

[10] Eckert, F.; Klamt, A. *COSMOtherm, Version C3.0, Release 16.01; COSMOlogic GmbH & Co. KG, Leverkusen, Germany* **2015**.

[11] Y. Zhao, D. G. Truhlar, *The Journal of Physical Chemistry A* **2005**, *109*, 5656-5667.

[12] F. Weigend, F. Furche, R. Ahlrichs, *The Journal of Chemical Physics* **2003**, *119*, 12753-12762.

[13] L. Goerigk, A. Hansen, C. Bauer, S. Ehrlich, A. Najibi, S. Grimme, *Physical Chemistry Chemical Physics* **2017**, *19*, 32184-32215.

Figure S43. TPSS-D3/def2-TZVP + COSMO(toluene) computed frontier molecular orbitals (in eV, at 0.02 a.u. contour). For **1** L^{1}GePCO, the HOMO and LUMO are mainly located on Ge and P electron lone pair and on the L^1 ligand anti-bonding π* oribitals, respectively. For L^{1}Ge$^+$ cation, the HOMO and LUMO are mainly located on two Ar-substituents and on the empty Ge pπ orbitals, respectively.

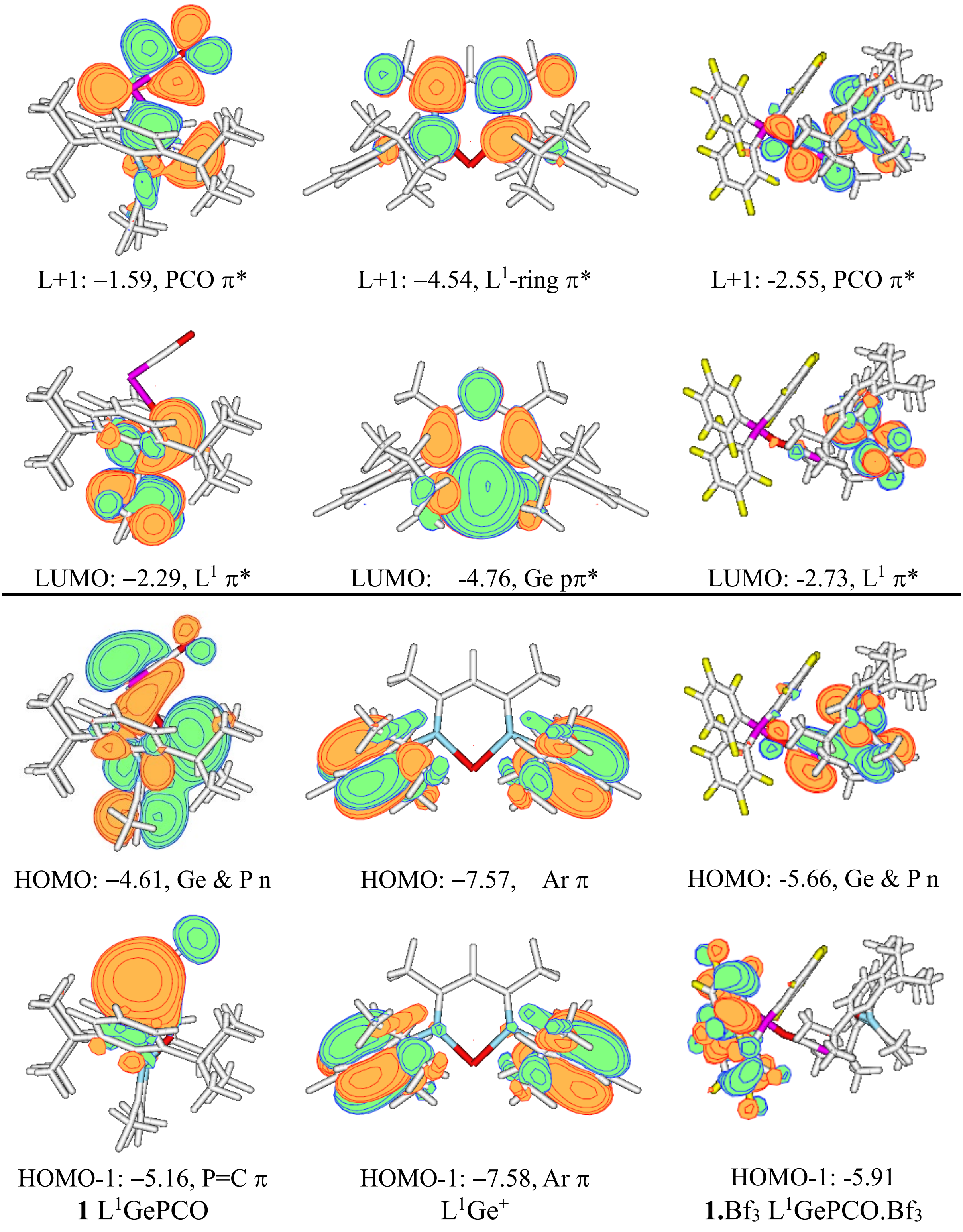

Table S3. TPSS-D3/def2-TZVP + COSMO computed lowest imaginary frequency (ImF), zero-point energies (ZPE), gas-phase enthalpic (Hc) and Gibbs free-energy (Gc) corrections; the COSMO-RS computed solvation enthalpic (Hsol) and Gibbs free-energy (Gsol) corrections in toluene solution; TPSS-D3/def2-QZVP and PW6B95-D3/def2-QZVP single-point energies (TPSS-D3 and PW6B95-D3); the PW6B95-D3 total free-energy (G_P); the relative electronic energies (ΔE_T and ΔE_P) and Gibbs free-energies (ΔG_T and ΔG_P) at the TPSS-D3 and PW6B95-D3 levels. Each structure is labeled either by its molecular formula or a specific name in bold. Transition structures (with only one imaginary frequency) are indicated by the "**TS**" prefix. $B(C_6F_5)_3$ and $Al(C_6F_5)_3$ are labeled as Bf_3 and Alf_3, respectively, for simplicity.

Reactions in toluene	ImF cm^{-1}	ZPE kcal /mol	Hc kcal /mol	Gc kcal /mol	Hsol kcal /mol	Gsol kcal /mol	TPSS-D3 E_h	PW6B95-D3 E_h	G_P E_h	ΔE_T kcal /mol	ΔE_P kcal /mol	*ΔG_P kcal /mol*	ΔG_T kcal /mol
Toluene (solvent)	0	80.00	84.16	61.71	-8.85	-1.97	-271.74572	-272.04260	-271.94741				
*The cation L^1Ge^+ is only -1.5 kcal/mol bound to toluene (Tol) in solution, but tightly bound to the anion PCO^- to form **1**.*													
L^1Ge^+ + Tol	0	477.43	503.17	423.30	-62.79	-47.50	-3588.77930	-3591.42702	-3590.82513	0.00	0.00	*0.00*	0.00
L^1GeL^+.Tol	0	475.64	502.87	434.48	-55.10	-46.64	-3588.80071	-3591.44865	-3590.82757	-13.43	-13.57	*-1.53*	-1.39
L^1Ge^+ + PCO^-	0	401.87	425.71	351.92	-115.05	-101.68	-3771.87101	-3774.60283	-3774.19802	0.00	0.00	*0.00*	0.00
**1**	0	400.95	426.10	361.22	-29.10	-20.79	-3772.04214	-3774.77300	-3774.22748	-107.39	-106.79	*-18.49*	-19.09
*..with the dimer **1d** being 10.9 kcal/mol less stable than two monomeric **1***													
2***1**	0	801.90	852.21	722.44	-58.19	-41.58	-7544.08427	-7549.54601	-7548.45497	0.00	0.00	0.00	0.00
**1d**	0	802.58	852.76	741.00	-46.34	-34.06	-7544.10284	-7549.56717	-7548.43758	-11.65	-13.28	10.91	12.54
*$B(C_6F_5)_3$ (or simply Bf_3) may be added to the C, P, O and Ge sites of **1**, and even abstract anion PCO^-*													
**1** + Bf_3	0	495.57	539.43	422.41	-45.97	-33.10	-5981.66448	-5986.64708	-5986.02065	0.00	0.00	*0.00*	0.00
**1**Bf_3c	0	496.41	540.28	440.28	-37.55	-28.40	-5981.66225	-5986.64890	-5985.98951	1.40	-1.14	*19.54*	22.08
**1**Bf_3p	0	495.71	540.11	438.94	-38.25	-29.04	-5981.68980	-5986.67721	-5986.02098	-15.89	-18.91	*-0.21*	2.81
**1**Bf_3o	0	495.17	539.74	437.70	-40.60	-30.90	-5981.68756	-5986.67462	-5986.02334	-14.48	-17.28	*-1.69*	1.11
**1**Bf_3g	0	496.55	540.57	440.55	-36.25	-27.80	-5981.68414	-5986.67309	-5986.01233	-12.34	-16.33	*5.22*	9.21
L^1Ge^+ + Bf_3OCP^-	0	497.28	540.07	424.36	-103.23	-88.45	-5981.56286	-5986.55185	-5986.01051	63.77	59.75	*6.36*	10.38
L^1Ge^+ + Bf_3PCO^-	0	496.91	539.82	423.98	-101.40	-87.20	-5981.57112	-5986.55670	-5986.01399	58.58	56.71	*4.18*	6.05
$L^1GeOCPBf_3$	0	495.79	540.13	438.63	-41.77	-31.95	-5981.67011	-5986.65635	-5986.00524	-3.53	-5.82	*9.67*	11.95
..Bf_3 is -14.3 kcal/mol bound to the P-site of PCO^-; the second Bf_3 binding to O-site is 5.6 kcal/mol endergonic thus unstable.													
Bf_3 + Bf_3PCO^-	0	194.09	234.13	123.58	-64.34	-53.99	-4874.15989	-4879.04635	-4878.92943	0.00	0.00	*0.00*	0.00
$Bf_3OCPBf_3^-$	0	194.25	234.89	139.37	-51.67	-44.33	-4874.18565	-4879.07491	-4878.92045	-16.17	-17.92	*5.64*	7.39

Bf_3 + Bf_3OCP^-	0	194.46	234.38	123.97	-66.17	-55.24	-4874.15162	-4879.04150	-4878.92595	5.19	3.04	*2.18*	4.33
PCO^- + 2*Bf_3	0	193.67	233.35	112.72	-94.87	-80.79	-4874.08212	-4878.96655	-4878.90663	48.80	50.08	*14.31*	13.04
Dimerization of Bf_3OCP^- is slightly endergonic by 1.2 kcal/mol													
2*Bf_3OCP^-	0	199.69	242.11	125.54	-98.60	-85.85	-5329.05856	-5334.33485	-5334.26557	0.00	0.00	*0.00*	0.00
**2**$^-$pp	0	200.36	242.77	144.03	-127.49	-118.34	-5329.03669	-5334.30765	-5334.26370	13.73	17.07	*1.17*	-2.17
*…while furtherBf_3 binding to form the dianion **2**— is energetically favorable*													
**2**$^-$pp + Bf_3	0	294.97	356.09	205.22	-144.37	-130.65	-7538.65903	-7546.18173	-7546.05687	0.00	0.00	*0.00*	0.00
**2**—	0	296.89	357.76	225.18	-123.00	-113.73	-7538.74342	-7546.27382	-7546.09321	-52.96	-57.79	*-22.81*	-17.97
Dimeric Alf_3d is -8.8 kcal/mol less stable than two monomeric Alf_3.Tol adducts in toluene solution													
Alf_3d + 2*Tol	0	342.68	391.06	251.42	-47.31	-26.70	-5398.01371	-5403.46859	-5403.10746	0.00	0.00	*0.00*	0.00
2*Alf_3.Tol	0	343.02	391.83	262.93	-48.88	-37.00	-5398.03310	-5403.48756	-5403.12150	-12.17	-11.90	*-8.81*	-9.07
*..Alf_3 transfer from Alf3.Tol adduct to 1 is -12.5 kcal/mol exergonic to the O-site of **1** to form the open complex **3a** in solution*													
**1** + Alf_3.Tol - Tol	0	492.47	537.86	430.98	-44.68	-37.32	-6199.31296	-6204.47419	-6203.84083	0.00	0.00	*0.00*	0.00
**3a**	0	493.10	538.26	434.79	-39.48	-30.18	-6199.34392	-6204.50855	-6203.86075	-19.43	-21.56	*-12.50*	-10.37
L^{1}Ge$^+$ + $PCOAlf_3^-$	0	493.94	537.94	418.89	-104.21	-88.94	-6199.21780	-6204.38326	-6203.85143	59.72	57.06	*-6.65*	-3.99
**3ac** (or *cis*-**3a**)	0	492.70	538.19	433.58	-41.52	-31.09	-6199.33930	-6204.50298	-6203.85857	-16.53	-18.07	*-11.14*	-9.59
**3_ts**	51i	490.79	536.73	430.22	-41.16	-30.86	-6199.33673	-6204.49870	-6203.85927	-14.91	-15.38	*-11.57*	-11.10
**3**	0	494.81	539.08	437.80	-44.33	-33.76	-6199.33771	-6204.49915	-6203.85226	-15.53	-15.66	*-7.17*	-7.04
Bf_3 binding to one ester group of diisopropyl but-2-ynedioate (or simply But) is almost neutral in free energy thus reversible													
But + Bf_3	0	235.69	264.67	176.91	-32.82	-23.02	-2900.42311	-2903.38206	-2903.13079	0.00	0.00	*0.00*	0.00
$ButBf_3$	0	236.94	266.13	192.98	-27.73	-20.66	-2900.44857	-2903.40848	-2903.13086	-15.98	-16.58	*-0.04*	0.56
***1** may formally act as intramolecular Ge/C FLP to activate But, initialized by nucleophilic attack via Ge to CC triple bond*													
**1** + But	0	542.02	577.45	476.93	-45.04	-31.49	-4462.84291	-4466.28099	-4465.56511	0.00	0.00	*0.00*	0.00
**TS1**	71i	538.96	576.08	487.28	-37.74	-27.41	-4462.85196	-4466.28329	-4465.54743	-5.68	-1.44	*11.09*	6.85
**A**	0	542.97	578.52	493.93	-40.33	-29.68	-4462.86008	-4466.29470	-4465.55186	-10.78	-8.60	*8.31*	6.14
**TS2**	51i	539.40	576.02	488.52	-40.56	-29.86	-4462.85613	-4466.28795	-4465.55401	-8.30	-4.37	*6.97*	3.04
**4**	0	546.22	580.41	499.07	-42.25	-31.35	-4462.91712	-4466.35881	-4465.61044	-46.57	-48.83	*-28.44*	-26.18
*Further Bf_3 binding to carbonyl O site of cyclic adduct **4** is still -22.8 kcal/mol exergonic to form **5***													
**4** + Bf_3	0	640.84	693.74	560.27	-59.12	-43.67	-6672.53946	-6678.23288	-6677.40360	0.00	0.00	*0.00*	0.00
**5a**	0	641.74	694.91	577.70	-48.07	-37.21	-6672.60212	-6678.29882	-6677.43448	-39.32	-41.37	*-19.38*	-17.32
**5**	0	640.55	694.13	576.17	-48.99	-38.29	-6672.60256	-6678.30007	-6677.43990	-39.59	-42.16	*-22.78*	-20.21

*..and Alf$_3$ binding to the PCO oxygen site of **4** is more exergonic to form the adduct **6***												
**4** + Alf$_3$.Tol	0	717.73	776.33	630.54	-66.69	-49.85	-7161.93367	-7168.10259	-7167.17119	0.00	0.00	*0.00*	0.00
**6** + Toluene	0	718.54	776.96	634.86	-57.78	-39.71	-7161.99657	-7168.16576	-7167.21432	-39.47	-39.64	*-27.07*	-26.90
*The formation of 7 is also -40.5 kcal/mol exergonic, likely via nucleophilic attack of **1** to the reversible ButBf$_3$ complex or Bf$_3$OCP$^-$ anion*												
**1** + Bf$_3$ + But	0	636.64	690.77	538.13	-61.92	-43.81	-6672.46525	-6678.15506	-6677.35828	0.00	0.00	*0.00*	0.00
**1** + ButBf$_3$	0	637.89	692.23	554.20	-56.83	-41.45	-6672.49071	-6678.18149	-6677.35835	-15.98	-16.58	*-0.04*	0.56
But + **1**Bf$_3$o	0	636.24	691.08	553.41	-56.54	-41.60	-6672.48833	-6678.18261	-6677.36097	-14.48	-17.28	*-1.69*	1.11
**7**	0	641.16	694.28	577.30	-47.66	-36.80	-6672.58505	-6678.28725	-6677.42289	-75.18	-82.95	*-40.54*	-32.77
*The adduct of PhCCH and Bf$_3$ (**B**) is 8.8 kcal/mol endergonic, thus is unstable in solution*												
PhCCH + Bf$_3$	0	163.46	186.45	111.80	-27.02	-18.88	-2518.22004	-2520.81393	-2520.65982	0.00	0.00	*0.00*	0.00
**B**	0	163.38	186.73	124.87	-25.49	-19.35	-2518.22668	-2520.81698	-2520.64580	-4.16	-1.91	*8.80*	6.55
*Direct addition of PhCCH to Ge/C FLP **1** is -20.5 kcal/mol exergonic but prevented by a sizable barrier of 27.6 kcal/nol (via **TS30**)*												
**1** + PhCCH	0	469.79	499.23	411.82	-39.25	-27.36	-4080.63984	-4083.71285	-4083.09414	0.00	0.00	*0.00*	0.00
**TS30**	67i	466.53	497.09	421.62	-35.46	-25.90	-4080.62408	-4083.68374	-4083.05012	9.89	18.27	*27.63*	19.24
**80**	0	472.82	501.27	430.78	-40.99	-30.51	-4080.69447	-4083.76761	-4083.12674	-34.28	-34.36	*-20.45*	-20.37
**TS3a**	54i	466.42	496.97	421.50	-34.17	-25.07	-4080.62305	-4083.68332	-4083.04856	10.54	18.53	*28.60*	20.61
**8a**	0	473.60	501.85	431.98	-38.82	-29.16	-4080.67326	-4083.74096	-4083.09603	-20.97	-17.64	*-1.18*	-4.52
*..but PhCCH can be easily activated by the intermolecular Ge/B FLP of 1 and Bf3 via trasient complex **B***												
**1** + PhCCH + BCF	0	564.40	612.55	473.02	-56.12	-39.67	-6290.26218	-6295.58693	-6294.88731	0.00	0.00	*0.00*	0.00
**TS3**	0	562.06	612.65	497.94	-44.95	-34.41	-6290.29037	-6295.60982	-6294.86814	-17.69	-14.37	*12.03*	8.71
**8**	0	566.61	615.08	506.23	-46.61	-36.64	-6290.33764	-6295.66103	-6294.90969	-47.35	-46.50	*-14.04*	-14.89
*Dimethyl maleate (Mal) is -4.4 kcal/mol bound to Bf$_3$ via ester C=O in solution, leading to a more electrophilic complex **C***												
Mal + Bf$_3$	0	180.57	206.39	125.41	-29.40	-20.52	-2744.30531	-2747.09698	-2746.92381	0.00	0.00	*0.00*	0.00
**C**	0	181.40	207.52	140.03	-26.29	-19.69	-2744.33123	-2747.12562	-2746.93084	-16.26	-17.98	*-4.41*	-2.70
*..which can be easily added to the nucleophilic Ge-center of **1**, followed by C-C ring-closing via PCO carbon to form the adduct **E***												
**1** + **C**	0	582.34	633.62	501.25	-55.38	-40.48	-6516.37337	-6521.89863	-6521.15832	0.00	0.00	*0.00*	0.00
**TS4**	55i	580.92	633.11	515.66	-44.89	-35.84	-6516.38694	-6521.90547	-6521.13782	-8.52	-4.29	*12.87*	8.64
**D**	0	585.42	635.63	524.05	-48.70	-37.98	-6516.40127	-6521.93293	-6521.15531	-17.51	-21.52	*1.89*	5.90
**TS5**	79i	581.88	633.27	518.09	-46.82	-36.21	-6516.39662	-6521.91830	-6521.14736	-14.59	-12.34	*6.88*	4.63
**E**	0	585.37	635.65	523.64	-47.35	-36.20	-6516.41694	-6521.94806	-6521.16826	-27.34	-31.02	*-6.23*	-2.56
**F** + Bf$_3$	0	584.34	634.59	505.51	-61.76	-44.16	-6516.37659	-6521.90143	-6521.16021	-2.02	-1.76	*-1.19*	-1.44

**9**	0	585.61	635.75	523.97	-47.20	-36.45	-6516.42944	-6521.95927	-6521.17934	-35.18	-38.06	*-13.19*	-10.32
*..in contrast, a sizable barrier of 23.8 kcal/mol (via **TS40**) is found for the direct addition between the Ge/C FLP **1** and Mal*													
**1** + Mal	0	486.90	519.17	425.43	-41.62	-29.00	-4306.72511	-4309.99590	-4309.35812	0.00	0.00	*0.00*	0.00
**TS40**	142i	484.89	517.95	437.79	-32.63	-23.58	-4306.71995	-4309.98325	-4309.32015	3.24	7.94	*23.83*	19.13
**D0**	0	489.06	520.51	444.41	-33.11	-24.01	-4306.74092	-4310.01060	-4309.33764	-9.92	-9.22	*12.86*	12.16
**TS50**	88i	485.88	518.26	439.77	-33.55	-24.37	-4306.73796	-4310.00210	-4309.33709	-8.07	-3.89	*13.20*	9.02
**F**	0	489.72	521.26	444.32	-44.88	-31.85	-4306.75424	-4310.02736	-4309.36704	-18.28	-19.74	*-5.60*	-4.14

Table S4. The TPSS-D3/def2-TZVP + COSMO optimized atomic Cartesian coordinates (in Å) in toluene solution. Each structure is labeled by the specific name (See also **Table S3**), followed by the number of atoms, the total energy, and the detailed atomic coordinates (in double-column text list).

1Bf3c : $B(C_6F_5)_3$ adduct at γ-carbon of **1** L^1GePCO
110
Energy = -5981.420625967

Ge	-3.2384560	-0.1204174	-0.1555820
P	-3.0593962	0.0597853	2.2537238
N	-1.7337637	-1.5088542	-0.4763698
N	-1.8701445	1.3491320	-0.6162416
C	-4.6876516	-0.2861059	2.2968338
C	-0.5135715	-1.3457306	-0.9048798
C	-2.4096718	-2.7996722	-0.5900279
C	-0.6653252	1.2218364	-1.1085968
C	-2.5711878	2.6276709	-0.6660480
O	-5.8300658	-0.5253778	2.3908138
C	0.1600723	-0.0234260	-1.0656985
C	0.2737895	-2.5424675	-1.3513020
C	-2.4768356	-3.6827922	0.5033920
C	-3.0758439	-3.0714976	-1.8070861
C	-0.0970141	2.3848086	-1.8700909
C	-2.2405836	3.6400266	0.2625523
C	-3.6203944	2.7994826	-1.5983096
H	0.6415224	-0.0676582	-2.0453524
H	1.2860665	-2.5079210	-0.9467304
H	-0.2101047	-3.4719689	-1.0571447
H	0.3679837	-2.5223490	-2.4410831
C	-3.2577469	-4.8368722	0.3615440
C	-1.6756182	-3.5063893	1.7810508
C	-3.8309245	-4.2447290	-1.8944223
C	-2.9849104	-2.1735117	-3.0322497
H	0.5372327	2.9940379	-1.2201358
H	0.5160273	2.0347018	-2.6994985
H	-0.8989610	3.0248833	-2.2381931
C	-3.0423198	4.7858127	0.2940130
C	-1.0463880	3.5829011	1.2001654
C	-4.3719207	3.9783670	-1.5310936
C	-3.9064779	1.8366211	-2.7441427
C	-3.9357676	-5.1177565	-0.8174743
H	-3.3206827	-5.5313170	1.1939182
C	-0.6053424	-4.6144225	1.8633507
C	-2.5640770	-3.5506622	3.0352766
H	-1.1784837	-2.5316509	1.7468671
H	-4.3445315	-4.4738803	-2.8233962
C	-4.3802910	-1.7858048	-3.5451878
C	-2.1781296	-2.8502649	-4.1578100
H	-2.4658017	-1.2494949	-2.7530807
C	-4.1077178	4.9556037	-0.5803709
H	-2.8129802	5.5631374	1.0169014
C	-1.4847515	3.5063460	2.6731660
C	-0.1238735	4.7987957	0.9864072
H	-0.4671928	2.6882308	0.9743691
H	-5.1718565	4.1342852	-2.2478125
C	-3.3843605	2.4165497	-4.0740114
C	-5.4025938	1.5028416	-2.8469953
H	-3.3672237	0.8994205	-2.5612593
H	-4.5359342	-6.0190405	-0.9012703
H	0.1263007	-4.5378076	1.0536188
H	-0.0726618	-4.5637203	2.8165616
H	-1.0765437	-5.6008567	1.7999929
H	-3.0345358	-4.5331651	3.1491710
H	-1.9580626	-3.3655785	3.9282403
H	-3.3473613	-2.7911304	2.9899241
H	-4.9258164	-2.6649718	-3.9025058
H	-4.9681393	-1.3145775	-2.7533916
H	-4.2916671	-1.0864322	-4.3829595
H	-2.6951172	-3.7503875	-4.5073344
H	-2.0695874	-2.1682721	-5.0079006
H	-1.1809266	-3.1474289	-3.8229859
H	-4.7151817	5.8548232	-0.5363165
H	-2.0317022	4.4118845	2.9590200
H	-2.1337459	2.6439032	2.8470840
H	-0.6074028	3.4160425	3.3218492
H	0.7506219	4.7189038	1.6387913
H	0.2227303	4.8706819	-0.0497034
H	-0.6371775	5.7336780	1.2325399
H	-3.5605126	1.7065089	-4.8894766
H	-3.9010090	3.3513673	-4.3166278
H	-2.3113149	2.6260200	-4.0276699
H	-5.7566908	1.0213491	-1.9297989
H	-5.9995852	2.4044404	-3.0129196
H	-5.5842118	0.8281026	-3.6876583
B	1.7115326	-0.0342336	-0.0992435
C	2.2960054	1.4959932	0.1790868
C	1.3805164	-0.8912007	1.2692112
C	2.9851032	-0.7014462	-0.9412946
C	2.7950726	2.2343702	-0.9070324
C	2.5700096	2.1033852	1.4127090
C	0.4916048	-0.3702585	2.2111445
C	1.9656273	-2.0960660	1.6750271
C	3.0956328	-1.0703803	-2.2833036
C	4.2043268	-0.7886550	-0.2510934
C	3.4306117	3.4673483	-0.8169396
C	3.2059399	3.3375788	1.5540536
C	0.2751489	-0.8867837	3.4819730
C	1.7863587	-2.6591718	2.9365892
C	4.2665342	-1.5312508	-2.8828522
C	5.3990270	-1.2364523	-0.8003210
C	3.6342875	4.0365048	0.4346280
C	0.9411543	-2.0494794	3.8537149
C	5.4333123	-1.6217635	-2.1365652
F	2.6813096	1.7454274	-2.1709325
F	2.2748682	1.5054953	2.5887152
F	-0.1751526	0.7696986	1.9052280
F	2.7665437	-2.8158046	0.8478067

F	2.0369702	-0.9691562	-3.1425951
F	4.2626638	-0.4256871	1.0552052
F	3.8460828	4.1088826	-1.9252317
F	3.4096710	3.8564552	2.7795983
F	-0.5398628	-0.2736303	4.3575299
F	2.4027578	-3.8111032	3.2619096
F	4.2763950	-1.8689171	-4.1870505
F	6.5165240	-1.3067751	-0.0537857
F	4.2316548	5.2309311	0.5566349
F	0.7417326	-2.5937263	5.0643087
F	6.5717154	-2.0632428	-2.6939060

1Bf3g : $B(C_6F_5)_3$ adduct at Ge of **1** L[1]GePCO

110

Energy = -5981.443793255

Ge	-0.0295853	-0.3685763	-1.0329258
P	-0.3637750	0.2601058	-3.3326951
N	1.5584590	-1.4652077	-1.4970365
N	-1.3024944	-1.9489258	-1.1350918
C	-1.2775030	1.6272407	-3.0665082
C	1.3685203	-2.3169918	-2.5386523
C	2.8481176	-1.5487044	-0.8147864
C	-1.0349813	-2.8621706	-2.0693248
C	-2.5872838	-1.9994676	-0.4446584
O	-1.9480504	2.5870263	-3.0598364
C	0.1296201	-2.8481744	-2.8678937
C	2.5421779	-2.7136939	-3.3961785
C	3.8968781	-0.6361193	-1.0639761
C	3.0483456	-2.6647705	0.0389283
C	-1.9525530	-4.0315709	-2.3186358
C	-3.7501765	-1.5671982	-1.1301046
C	-2.6660041	-2.5332967	0.8589617
H	0.1139432	-3.5242180	-3.7149317
H	2.7156447	-1.9071580	-4.1188038
H	3.4548955	-2.8427559	-2.8137453
H	2.3286690	-3.6290412	-3.9496001
C	5.0929791	-0.7953369	-0.3504554
C	3.8790976	0.4326018	-2.1454172
C	4.2764159	-2.7912506	0.6937735
C	2.0173551	-3.7741923	0.2126472
H	-2.8606804	-3.9964393	-1.7205998
H	-2.2139683	-4.0615637	-3.3805763
H	-1.4101675	-4.9566931	-2.0972911
C	-4.9677654	-1.6119437	-0.4423374
C	-3.7849718	-1.1386515	-2.5916987
C	-3.9095117	-2.5377315	1.5004216
C	-1.5089627	-3.2586376	1.5234904
C	5.2858203	-1.8506110	0.5286568
H	5.8980771	-0.0865194	-0.5187366
C	4.8130116	0.0333549	-3.3094891
C	4.3436022	1.8043765	-1.6170808
H	2.8636252	0.5160103	-2.5387781
H	4.4399008	-3.6363513	1.3535226
C	1.9460612	-4.3112467	1.6508064
C	2.3167942	-4.9568808	-0.7325601
H	1.0365624	-3.3683389	-0.0534047
C	-5.0528464	-2.0698853	0.8658470
H	-5.8655632	-1.2735000	-0.9504645
C	-4.4174446	0.2512500	-2.7910083
C	-4.5906317	-2.1483539	-3.4380462
H	-2.7595228	-1.0968572	-2.9724709
H	-3.9821376	-2.9397777	2.5058616
C	-1.6895195	-4.7746473	1.2898237
C	-1.3875375	-2.9843765	3.0289940
H	-0.5816615	-2.9435789	1.0418105
H	6.2235273	-1.9524518	1.0673039
H	4.5547403	-0.9358655	-3.7405866
H	4.7597631	0.7882379	-4.1017058
H	5.8501758	-0.0207783	-2.9606298
H	3.8387076	2.0790819	-0.6906987
H	5.4225222	1.7963985	-1.4277007
H	4.1425607	2.5797772	-2.3622506
H	1.7428336	-3.5198282	2.3720179
H	1.1546051	-5.0622034	1.7188299
H	2.8798766	-4.8055787	1.9366507
H	2.3120077	-4.6629425	-1.7832905
H	3.3003616	-5.3820452	-0.5031580
H	1.5646202	-5.7419185	-0.5956219
H	-6.0077990	-2.0749895	1.3831721
H	-3.9348256	1.0169158	-2.1877668
H	-4.3407432	0.5386296	-3.8449436
H	-5.4807462	0.2309065	-2.5274956
H	-4.2311280	-3.1733020	-3.3307011
H	-5.6454132	-2.1324234	-3.1421774
H	-4.5340830	-1.8712533	-4.4963776
H	-1.6616606	-5.0273142	0.2268522
H	-0.9032004	-5.3395030	1.7992669
H	-2.6556666	-5.1018757	1.6890030
H	-1.3047994	-1.9169329	3.2362354
H	-2.2489421	-3.3771164	3.5790384
H	-0.4943735	-3.4788414	3.4217421
B	-0.0228005	1.2076167	0.7011373
C	-1.3412672	0.8635175	1.5744375
C	1.4090447	1.0002878	1.4238857
C	-0.1634597	2.6215453	-0.0864372
C	-2.5952672	0.9632522	0.9641327
C	-1.3976973	0.6553032	2.9547440
C	1.7492212	-0.2322656	1.9854456
C	2.3646084	2.0016071	1.6363050
C	0.6687001	2.9123334	-1.1659563
C	-1.0139097	3.6762120	0.2655077
C	-3.8088047	0.9062815	1.6299692
C	-2.5932959	0.5335494	3.6626042
C	2.9015411	-0.4789677	2.7156125
C	3.5616665	1.7891018	2.3189420
C	0.6645896	4.0974449	-1.8864863
C	-1.0667125	4.8824190	-0.4300842
C	-3.8070275	0.6643668	3.0004765
C	3.8336275	0.5427532	2.8659130
C	-0.2285774	5.0969188	-1.5192256
F	-2.6418805	1.2129101	-0.3694210
F	-0.2723644	0.5929151	3.7067347
F	0.8764632	-1.2625112	1.8478675
F	2.1734638	3.2707739	1.2050350

F	1.5917111	1.9891905	-1.5222896
F	-1.8231722	3.6018363	1.3490637
F	-4.9710728	1.1117116	0.9863953
F	-2.5794222	0.2996123	4.9890753
F	3.1224707	-1.6710986	3.2993592
F	4.4503564	2.7901032	2.4643965
F	1.5029963	4.2821997	-2.9233508
F	-1.9144370	5.8541088	-0.0446621
F	-4.9646220	0.5590162	3.6722314
F	4.9746555	0.3253893	3.5377774
F	-0.2736606	6.2547632	-2.1950055

1Bf3o : $B(C_6F_5)_3$ adduct at oxygen of **1** L[1]GePCO
110
Energy = -5981.446727402

Ge	2.3405475	-0.2722863	-0.2221330
P	0.8387814	-0.0246194	-2.3760414
N	3.1978229	1.4282456	-0.8484239
N	3.6183325	-1.3613589	-1.2728214
C	-0.4494248	-0.4380949	-1.5165668
C	3.9441001	1.5313411	-1.9499582
C	2.8733664	2.5761009	-0.0285713
C	4.2311224	-0.9279900	-2.3883536
C	3.8404666	-2.7193282	-0.8181322
O	-1.4547377	-0.8240316	-0.9444297
C	4.2911304	0.4212976	-2.7436029
C	4.4828156	2.8698946	-2.3871058
C	1.9277318	3.5290786	-0.4641130
C	3.4939052	2.6780131	1.2381034
C	4.9190155	-1.9334464	-3.2737549
C	2.9902208	-3.7621198	-1.2367837
C	4.9191182	-2.9569893	0.0588128
H	4.8234597	0.6484648	-3.6597945
H	3.8942244	3.2400731	-3.2334817
H	4.4345320	3.6084367	-1.5866303
H	5.5156404	2.7618584	-2.7269631
C	1.6308404	4.5955798	0.3936208
C	1.2092159	3.4653123	-1.8021333
C	3.1652705	3.7671044	2.0486003
C	4.5432210	1.6722221	1.6881419
H	4.1968811	-2.6761021	-3.6280622
H	5.3775774	-1.4401779	-4.1314788
H	5.6871300	-2.4803646	-2.7190470
C	3.2555908	-5.0555062	-0.7731256
C	1.7927204	-3.5263905	-2.1385859
C	5.1483090	-4.2690174	0.4858955
C	5.8330381	-1.8455971	0.5488070
C	2.2406893	4.7210931	1.6339276
H	0.8987701	5.3335316	0.0789743
C	1.4608948	4.7301840	-2.6454657
C	-0.3033958	3.2724902	-1.6006720
H	1.5837388	2.5990595	-2.3582984
H	3.6306247	3.8662765	3.0226489
C	4.6682804	1.5656683	3.2129982
C	5.9095232	1.9995517	1.0547814
H	4.2364117	0.6834326	1.3174594
C	4.3277991	-5.3138806	0.0739989
H	2.6083432	-5.8708566	-1.0833581
C	0.4884190	-3.6627138	-1.3320470
C	1.7766375	-4.4635342	-3.3577041
H	1.8445580	-2.4970633	-2.5095496
H	5.9805309	-4.4718494	1.1543427
C	7.2596939	-1.9989750	-0.0098982
C	5.8563932	-1.7957255	2.0859583
H	5.4352899	-0.8936528	0.1843909
H	1.9891461	5.5535995	2.2839817
H	2.5275895	4.9342540	-2.7732862
H	1.0078123	4.6143293	-3.6357016
H	1.0075082	5.6069350	-2.1711009
H	-0.7363031	4.1286361	-1.0733172
H	-0.8052268	3.1731184	-2.5683665
H	-0.5080086	2.3725016	-1.0185041
H	5.0761440	2.4849818	3.6465455
H	3.7011675	1.3625711	3.6806371
H	5.3525173	0.7530105	3.4709287
H	6.2422265	2.9946483	1.3693151
H	6.6628437	1.2702370	1.3704809
H	5.8601685	1.9867528	-0.0376234
H	4.5195076	-6.3262294	0.4181235
H	0.3769521	-4.6858175	-0.9557555
H	0.4812018	-2.9863050	-0.4721891
H	-0.3777433	-3.4299542	-1.9595886
H	0.9407615	-4.2031803	-4.0154393
H	2.7041346	-4.3889727	-3.9353862
H	1.6513454	-5.5089074	-3.0564141
H	7.8925012	-1.1734036	0.3337888
H	7.7112642	-2.9378021	0.3291235
H	7.2633054	-1.9980479	-1.1042955
H	4.8510533	-1.6445513	2.4912406
H	6.2527859	-2.7266671	2.5039985
H	6.4983639	-0.9780302	2.4288146
B	-2.4502986	-0.1387904	0.1505238
C	-3.2158825	-1.4523137	0.7408402
C	-1.4541568	0.6130816	1.1901247
C	-3.5312534	0.7937255	-0.6322238
C	-3.7737177	-2.3837929	-0.1408591
C	-3.4323308	-1.7275352	2.0919576
C	-0.4628645	-0.1241466	1.8485697
C	-1.5016670	1.9628953	1.5473146
C	-3.5335174	1.1719915	-1.9741259
C	-4.6359598	1.2457423	0.0966660
C	-4.4589530	-3.5234532	0.2684516
C	-4.1187384	-2.8514525	2.5462463
C	0.4065466	0.4082655	2.7921252
C	-0.6593854	2.5362566	2.4990138
C	-4.5429883	1.9397107	-2.5542221
C	-5.6650960	2.0102229	-0.4377115
C	-4.6321067	-3.7606882	1.6281993
C	0.2980849	1.7533803	3.1312769
C	-5.6190579	2.3615537	-1.7839103
F	-3.6759723	-2.1936395	-1.4797301
F	-2.9846194	-0.8856860	3.0536051
F	-0.3366516	-1.4502320	1.5957241
F	-2.3993378	2.8064190	0.9887862

F	-2.5299679	0.8094236	-2.8116716
F	-4.7165692	0.9614402	1.4202778
F	-4.9648925	-4.3881564	-0.6324392
F	-4.2926055	-3.0644575	3.8649884
F	1.3350704	-0.3604443	3.3930790
F	-0.7753739	3.8385072	2.8175465
F	-4.4825455	2.2729975	-3.8588039
F	-6.6933146	2.4212592	0.3282390
F	-5.2961979	-4.8502157	2.0486601
F	1.1024030	2.2826720	4.0665801
F	-6.6002543	3.1000681	-2.3286988

1Bf3p : $B(C_6F_5)_3$ adduct at P of **1** L[1]GePCO

110

Energy = -5981.447961770

Ge	-2.1483774	-0.2586466	-1.2855857
P	0.4317034	-0.1850153	-0.7632561
N	-2.7782081	1.1397679	-0.0566871
N	-2.4811252	-1.6909283	0.0576774
C	0.6537502	-0.0350518	-2.4153664
C	-2.7951705	1.0243740	1.2868378
C	-3.2976757	2.3570752	-0.6545874
C	-2.6287304	-1.4802233	1.3693879
C	-2.5920001	-3.0180829	-0.5173610
O	0.7350771	0.0218686	-3.5745291
C	-2.6423927	-0.1972522	1.9408453
C	-3.0182310	2.2593677	2.1191158
C	-2.4287489	3.3951325	-1.0381816
C	-4.6953820	2.4680508	-0.8230680
C	-2.8226003	-2.6370228	2.3165947
C	-1.5357062	-3.9443396	-0.4052505
C	-3.7764886	-3.3338059	-1.2229273
H	-2.6999215	-0.1664697	3.0215394
H	-2.1484023	2.9179053	2.0262689
H	-3.8863295	2.8238686	1.7725236
H	-3.1469160	1.9963432	3.1686270
C	-2.9891541	4.5657838	-1.5633055
C	-0.9201674	3.2837052	-0.9338316
C	-5.2058951	3.6620305	-1.3416148
C	-5.6593198	1.3481003	-0.4625387
H	-3.0665216	-3.5614143	1.7941413
H	-1.9000855	-2.7946514	2.8860098
H	-3.6121860	-2.4011155	3.0342600
C	-1.7019920	-5.2037794	-0.9959343
C	-0.2216105	-3.6565956	0.3011021
C	-3.8861072	-4.6025732	-1.7975377
C	-4.9424672	-2.3606507	-1.3036071
C	-4.3643920	4.7092470	-1.7035860
H	-2.3309219	5.3760208	-1.8637577
C	-0.3090935	4.4122308	-0.0877408
C	-0.3051576	3.2645403	-2.3445917
H	-0.6764952	2.3335503	-0.4496263
H	-6.2798987	3.7708701	-1.4638802
C	-6.5427641	0.9836084	-1.6678585
C	-6.5342231	1.7132815	0.7510832
H	-5.0712811	0.4658257	-0.1933445
C	-2.8602672	-5.5362867	-1.6839688
H	-0.8983355	-5.9299158	-0.9168058
C	0.9477022	-3.7558965	-0.6950158
C	0.0170615	-4.6280703	1.4729247
H	-0.2500040	-2.6319562	0.6889344
H	-4.7881227	-4.8664211	-2.3385824
C	-5.8020955	-2.4481770	-0.0276037
C	-5.8072493	-2.5588078	-2.5552513
H	-4.5292361	-1.3429423	-1.3540950
H	-4.7799299	5.6300382	-2.1028646
H	-0.7379756	4.4355336	0.9186304
H	0.7698375	4.2610306	0.0066706
H	-0.4741816	5.3911180	-0.5504974
H	-0.7370053	2.4636380	-2.9524855
H	-0.4880438	4.2137560	-2.8600634
H	0.7753697	3.1093337	-2.2914214
H	-5.9343989	0.6973160	-2.5312640
H	-7.2050931	0.1499673	-1.4138379
H	-7.1734079	1.8279282	-1.9641680
H	-5.9280908	1.9146841	1.6387861
H	-7.1351347	2.6046814	0.5405619
H	-7.2174513	0.8897840	0.9859762
H	-2.9643596	-6.5179569	-2.1370283
H	0.7968941	-3.0920778	-1.5482020
H	1.8846189	-3.4796445	-0.2038152
H	1.0484935	-4.7813750	-1.0665491
H	-0.7898688	-4.5937256	2.2095053
H	0.0905056	-5.6573800	1.1063543
H	0.9572759	-4.3876768	1.9766590
H	-5.2193433	-2.2091409	0.8661864
H	-6.6440615	-1.7505350	-0.0819078
H	-6.2023946	-3.4611200	0.0880094
H	-5.1987619	-2.5464753	-3.4648268
H	-6.3547462	-3.5064574	-2.5194733
H	-6.5477345	-1.7579496	-2.6249811
B	2.2532059	0.1642090	0.3812220
C	3.5475090	-0.7079743	-0.0959778
C	2.6836661	1.7283657	0.2458423
C	1.5935584	-0.2895663	1.7991870
C	3.7752463	-1.4310722	-1.2662810
C	4.6571629	-0.6495733	0.7591517
C	3.0108233	2.2522106	-1.0100436
C	2.9319828	2.6074095	1.3077206
C	0.6422805	0.5112363	2.4423238
C	1.8828417	-1.4801983	2.4764984
C	4.9811126	-2.0702296	-1.5575131
C	5.8761039	-1.2662366	0.5112585
C	3.4749329	3.5442256	-1.2285387
C	3.4001997	3.9090432	1.1371774
C	0.0808988	0.2189118	3.6780522
C	1.3208884	-1.8287263	3.7033128
C	6.0415134	-1.9910305	-0.6651630
C	3.6666287	4.3886820	-0.1399358
C	0.4156131	-0.9704343	4.3150919
F	2.8231620	-1.5567771	-2.2198494
F	4.5586502	0.0363017	1.9258084
F	2.8958885	1.4704081	-2.1118593
F	2.7595056	2.2253907	2.5935275

F	0.2256587	1.6595080	1.8551930
F	2.7504467	-2.3844922	1.9662295
F	5.1250245	-2.7575334	-2.7069666
F	6.8895889	-1.1756898	1.3931003
F	3.7409705	3.9804729	-2.4739073
F	3.6038400	4.7060347	2.2030611
F	-0.7913541	1.0648187	4.2617357
F	1.6480370	-2.9893609	4.3020394
F	7.2084062	-2.5989151	-0.9337023
F	4.1103246	5.6425410	-0.3192373
F	-0.1411364	-1.2903196	5.4947084

1d : cyclic dimer of **1** L^1GePCO
152
Energy = -7543.828812494

Ge	2.8461302	-0.5520631	-0.6092256
P	1.0095665	-0.2164815	1.0028120
C	0.2311607	1.1780133	0.0096479
O	0.4652371	2.3665996	0.0241400
N	3.6543891	-2.1660842	0.3061861
C	4.9799712	-2.2590771	0.4265641
C	2.7706941	-3.2790040	0.5285598
C	5.8323356	-1.1418755	0.3671164
C	5.6193840	-3.6167031	0.5983057
C	2.2674778	-3.5006456	1.8296380
C	2.3534933	-4.0620678	-0.5651354
C	5.4582124	0.2111963	0.4561633
H	6.8946237	-1.3482244	0.4349229
H	6.4891862	-3.5593083	1.2565133
H	4.9119080	-4.3522672	0.9826919
H	5.9645415	-3.9645182	-0.3835451
C	1.3732116	-4.5581488	2.0155773
C	2.6888275	-2.6255597	3.0003692
C	1.4529190	-5.1050550	-0.3261554
C	2.8320138	-3.7999927	-1.9844940
C	6.5581763	1.2267854	0.6574921
N	4.1946302	0.6229788	0.3390535
C	0.9701446	-5.3588620	0.9508800
H	0.9720827	-4.7472628	3.0065159
C	3.8905564	-3.2226112	3.7571742
C	1.5350620	-2.3531206	3.9759181
H	3.0025296	-1.6643104	2.5787827
H	1.1141041	-5.7138524	-1.1602185
C	1.6603682	-3.3725040	-2.8877733
C	3.5579995	-5.0240471	-2.5713857
H	3.5470105	-2.9703203	-1.9562114
H	6.1726834	2.1709808	1.0437749
H	7.3302486	0.8400957	1.3264579
H	7.0274729	1.4270703	-0.3140648
C	3.7898563	1.9801542	0.5908720
H	0.2605142	-6.1651748	1.1151562
H	4.7582914	-3.3461004	3.1046823
H	4.1794989	-2.5650093	4.5852540
H	3.6346550	-4.2043674	4.1724842
H	0.6729998	-1.9289211	3.4536575
H	1.2178489	-3.2662437	4.4918001
H	1.8590900	-1.6397502	4.7406327
H	1.1770719	-2.4711363	-2.5012428
H	2.0200971	-3.1667093	-3.9023995
H	0.9011976	-4.1604956	-2.9425642
H	4.4002076	-5.3262607	-1.9403383
H	2.8782964	-5.8791470	-2.6572451
H	3.9400631	-4.7951909	-3.5725940
C	3.4020080	2.3446921	1.8991661
C	3.6983274	2.8865460	-0.4831643
C	2.9664146	3.6550836	2.1129744
C	3.4614264	1.3496348	3.0482325
C	3.2514513	4.1846741	-0.2166437
C	4.0474514	2.4964246	-1.9108249
C	2.8946792	4.5719181	1.0683050
H	2.6615107	3.9579262	3.1099970
C	2.2816932	1.5017655	4.0190290
C	4.7934382	1.4431486	3.8166039
H	3.4004671	0.3502241	2.6037280
H	3.1670185	4.8941349	-1.0354964
C	5.1802516	3.3727778	-2.4752268
C	2.8026924	2.5587522	-2.8153936
H	4.4000903	1.4589841	-1.9052131
H	2.5370166	5.5809821	1.2541250
H	1.3275695	1.4378316	3.4885457
H	2.3137154	0.7026666	4.7667056
H	2.3208205	2.4561390	4.5559184
H	5.6500480	1.2549702	3.1647210
H	4.9141068	2.4395899	4.2574936
H	4.8147166	0.7051058	4.6268609
H	6.0730174	3.3226972	-1.8433546
H	5.4506657	3.0409803	-3.4839495
H	4.8704867	4.4220151	-2.5371965
H	2.0163601	1.8959171	-2.4443863
H	2.3942818	3.5748218	-2.8488771
H	3.0605343	2.2550037	-3.8364100
Ge	-2.8423677	0.5485026	0.6076133
P	-1.0135889	0.2068166	-1.0123164
C	-0.2324502	-1.1885185	-0.0218195
O	-0.4638110	-2.3775739	-0.0392130
N	-4.1980568	-0.6217814	-0.3357208
C	-5.4608489	-0.2060169	-0.4480381
C	-3.7982826	-1.9800572	-0.5892174
C	-5.8300959	1.1481841	-0.3577431
C	-6.5646505	-1.2183642	-0.6445713
C	-3.4171056	-2.3457881	-1.8991636
C	-3.7053260	-2.8868103	0.4843955
C	-4.9742230	2.2625624	-0.4208219
H	-6.8919178	1.3581096	-0.4215730
H	-7.3404672	-0.8280601	-1.3070795
H	-6.1840391	-2.1624143	-1.0360538
H	-7.0274856	-1.4205096	0.3297103
C	-2.9861997	-3.6574538	-2.1147004
C	-3.4776064	-1.3503435	-3.0478808
C	-3.2629916	-4.1861399	0.2161644
C	-4.0482231	-2.4957469	1.9132972
C	-5.6104063	3.6218957	-0.5913441
N	-3.6485755	2.1655048	-0.3047690
C	-2.9126797	-4.5744230	-1.0702558

H	-2.6863838	-3.9612563	-3.1129625
C	-4.8134206	-1.4379417	-3.8102992
C	-2.3029288	-1.5072893	-4.0240802
H	-3.4103154	-0.3512894	-2.6034814
H	-3.1772453	-4.8958323	1.0346787
C	-2.8000074	-2.5596572	2.8129537
C	-5.1800885	-3.3701710	2.4825323
H	-4.3992956	-1.4577759	1.9087280
H	-4.8995062	4.3578552	-0.9684962
H	-6.4761116	3.5682698	-1.2553085
H	-5.9617804	3.9661090	0.3895628
C	-2.7620014	3.2755937	-0.5301494
H	-2.5587681	-5.5845448	-1.2574992
H	-5.6662840	-1.2452378	-3.1548566
H	-4.8347001	-0.7002202	-4.6208483
H	-4.9407227	-2.4340181	-4.2501606
H	-1.3460741	-1.4484485	-3.4978968
H	-2.3491837	-2.4609497	-4.5616806
H	-2.3343650	-0.7073284	-4.7708437
H	-2.0143006	-1.8978429	2.4386974
H	-3.0535527	-2.2555241	3.8349363
H	-2.3927507	-3.5762238	2.8450033
H	-6.0752053	-3.3191030	1.8540711
H	-4.8716442	-4.4198222	2.5439170
H	-5.4461368	-3.0374078	3.4920942
C	-2.2631473	3.4963785	-1.8329933
C	-2.3381788	4.0568844	0.5623005
C	-1.3651164	4.5502321	-2.0216168
C	-2.6921213	2.6238356	-3.0028134
C	-1.4342263	5.0962925	0.3205851
C	-2.8124171	3.7960751	1.9833364
C	-0.9548168	5.3486400	-0.9579967
H	-0.9669526	4.7382609	-3.0139618
C	-1.5420431	2.3446485	-3.9808582
C	-3.8916959	3.2285695	-3.7569190
H	-3.0107265	1.6644037	-2.5807604
H	-1.0901829	5.7034555	1.1536958
C	-3.5327975	5.0223029	2.5726059
C	-1.6388814	3.3650368	2.8823910
H	-3.5299854	2.9685518	1.9575397
H	-0.2424045	6.1520509	-1.1244313
H	-0.6816034	1.9147195	-3.4606394
H	-1.8721231	1.6337840	-4.7453206
H	-1.2201793	3.2560979	-4.4968102
H	-4.7566558	3.3590424	-3.1020910
H	-3.6299152	4.2080870	-4.1738503
H	-4.1875817	2.5722961	-4.5836090
H	-4.3765336	5.3266329	1.9446169
H	-3.9117203	4.7948305	3.5753250
H	-2.8503944	5.8755384	2.6555900
H	-1.1599560	2.4621176	2.4940785
H	-0.8769133	4.1505030	2.9343349
H	-1.9953719	3.1601655	3.8983533

1 : complex L[1]GePCO of cation L[1]Ge$^+$ and PCO$^-$
76
Energy = -3771.907287290

Ge	-0.0034174	-1.0560590	0.2132732
P	-0.0202067	0.3989490	2.2103534
C	0.0954319	-0.9833662	3.1147986
O	0.1779868	-1.9449686	3.7854694
N	1.4105269	-0.0173902	-0.8182000
C	1.2527060	0.1930699	-2.1279190
C	2.6879583	0.1616296	-0.1854765
C	-0.0023528	0.1699404	-2.7592968
C	2.4688170	0.4410530	-2.9869435
C	2.9958268	1.4224089	0.3706820
C	3.5735899	-0.9282012	-0.0798935
C	-1.2570961	0.1914407	-2.1282594
H	-0.0024313	0.3154774	-3.8327744
H	2.1892770	0.9168322	-3.9283624
H	3.2124293	1.0510547	-2.4708036
H	2.9439916	-0.5214492	-3.2130327
C	4.2361064	1.5804370	0.9946515
C	2.0089768	2.5770367	0.2929297
C	4.7992298	-0.7223372	0.5648130
C	3.2322734	-2.3087571	-0.6187907
C	-2.4738153	0.4339096	-2.9880783
N	-1.4164160	-0.0185064	-0.8179366
C	5.1357139	0.5205379	1.0891647
H	4.4991357	2.5424681	1.4238938
C	2.2483162	3.4629526	-0.9447574
C	2.0089988	3.4365041	1.5652418
H	1.0138715	2.1305063	0.1868431
H	5.4973520	-1.5504695	0.6550608
C	3.0300139	-3.3071584	0.5366555
C	4.3019996	-2.8258034	-1.5974504
H	2.2846256	-2.2369902	-1.1641015
H	-3.2137706	1.0536657	-2.4781792
H	-2.1936806	0.8971602	-3.9354960
H	-2.9543432	-0.5288294	-3.2013961
C	-2.6956706	0.1626661	-0.1893655
H	6.0928764	0.6625639	1.5834840
H	2.1219272	2.9025922	-1.8743308
H	1.5353984	4.2953154	-0.9538709
H	3.2616809	3.8803375	-0.9302813
H	1.8559949	2.8194119	2.4554159
H	2.9490042	3.9875521	1.6795144
H	1.2017313	4.1741623	1.5131918
H	2.2398855	-2.9682378	1.2141297
H	2.7483040	-4.2910182	0.1451083
H	3.9526217	-3.4191935	1.1173333
H	4.4642343	-2.1230746	-2.4211978
H	5.2611966	-2.9749258	-1.0897584
H	3.9931146	-3.7881717	-2.0200502
C	-3.0025430	1.4237601	0.3665096
C	-3.5846263	-0.9248497	-0.0887370
C	-4.2448509	1.5845304	0.9857531
C	-2.0127870	2.5762364	0.2923750
C	-4.8125649	-0.7162617	0.5507055
C	-3.2435259	-2.3057564	-0.6267562
C	-5.1473498	0.5267045	1.0760982
H	-4.5072241	2.5469225	1.4145261
C	-2.0219242	3.4415551	1.5608019

C	-2.2396059	3.4576145	-0.9508733
H	-1.0172982	2.1280054	0.1955399
H	-5.5134757	-1.5424456	0.6369371
C	-4.3152745	-2.8264668	-1.6011483
C	-3.0363854	-3.3012503	0.5303626
H	-2.2975297	-2.2337894	-1.1749189
H	-6.1058069	0.6705547	1.5673625
H	-1.8806721	2.8288884	2.4560098
H	-1.2113427	4.1757830	1.5132517
H	-2.9608992	3.9967297	1.6628839
H	-2.1048205	2.8936909	-1.8770856
H	-3.2525982	3.8760930	-0.9473501
H	-1.5256935	4.2891191	-0.9562350
H	-4.4819684	-2.1251244	-2.4252001
H	-4.0053336	-3.7884993	-2.0236966
H	-5.2724935	-2.9776576	-1.0903531
H	-2.2437162	-2.9596894	1.2036243
H	-3.9567793	-3.4120904	1.1147913
H	-2.7554406	-4.2859020	0.1403050

2^{-}oo : C_2P_2-ring dimer of Bf_3OCP^-
74
Energy = -5328.832826060

P	-0.3859289	-0.6091488	-1.2537318
C	-1.0586902	0.1474527	0.2238109
O	-2.2597776	0.4017827	0.6223156
B	-3.5545693	0.1357553	-0.1431403
C	-3.3921592	-1.2985728	-0.9160722
C	-4.7052359	0.1375702	1.0485605
C	-3.9290972	1.4085624	-1.1193920
C	-3.0512132	-2.4243783	-0.1580050
C	-3.4451171	-1.5245507	-2.2912861
C	-5.7104947	-0.8115874	1.2364833
C	-4.7439685	1.2054915	1.9521862
C	-3.1513618	2.5443543	-1.3538283
C	-5.1862064	1.4339400	-1.7291352
C	-2.7924272	-3.6773977	-0.6963240
C	-3.1936934	-2.7647476	-2.8757640
C	-6.6609724	-0.7398973	2.2542945
C	-5.6722784	1.3156720	2.9848013
C	-3.5822543	3.6148929	-2.1406131
C	-5.6550115	2.4760700	-2.5190723
C	-2.8612372	-3.8478901	-2.0743204
C	-6.6394486	0.3293718	3.1405561
C	-4.8396144	3.5834330	-2.7281557
F	-2.9641857	-2.3230802	1.1899975
F	-3.7221057	-0.5247103	-3.1630400
F	-5.8313254	-1.8794739	0.4114563
F	-3.8667613	2.2295609	1.8398851
F	-1.9185256	2.6833004	-0.8241590
F	-6.0244840	0.3763569	-1.5747478
F	-2.4642894	-4.7247272	0.0920991
F	-3.2549857	-2.9221625	-4.2177135
F	-7.6076327	-1.6974303	2.3887538
F	-5.6550626	2.3723866	3.8294001
F	-2.7868174	4.6916435	-2.3341307
F	-6.8811579	2.4281050	-3.0898039
F	-2.6093102	-5.0537156	-2.6260470
F	-7.5518497	0.4150149	4.1331999
F	-5.2674993	4.6138062	-3.4904407
P	0.4285252	0.6080559	1.2308453
C	1.0858417	-0.1452188	-0.2493424
O	2.3020663	-0.3902677	-0.6528360
B	3.5678595	-0.1367846	0.1292974
C	4.7413742	-0.0994380	-1.0455097
C	3.3971333	1.2802732	0.9376240
C	3.9460930	-1.4273918	1.0856319
C	5.7369164	0.8640057	-1.2031506
C	4.7971985	-1.1422252	-1.9768474
C	3.0167573	2.4090156	0.2042399
C	3.4341702	1.4795704	2.3158075
C	5.1954364	-1.4681330	1.7075748
C	3.1623338	-2.5667905	1.2884754
C	6.6943970	0.8302047	-2.2159992
C	5.7322462	-1.2137496	-3.0066341
C	2.7119219	3.6409471	0.7663086
C	3.1281608	2.6941422	2.9253164
C	5.6535999	-2.5240287	2.4848878
C	3.5834236	-3.6502387	2.0635354
C	6.6899125	-0.2142650	-3.1308109
C	2.7603322	3.7819649	2.1469691
C	4.8334861	-3.6319773	2.6667607
F	5.8422613	1.9135802	-0.3494160
F	3.9328243	-2.1796100	-1.8973588
F	2.9384890	2.3345312	-1.1473510
F	3.7394668	0.4701342	3.1718671
F	6.0415440	-0.4090489	1.5820424
F	1.9415523	-2.6968780	0.7417123
F	7.6330858	1.8021379	-2.3185424
F	5.7312306	-2.2482884	-3.8809381
F	2.3387329	4.6892651	-0.0022371
F	3.1665562	2.8209297	4.2744955
F	6.8758544	-2.4887667	3.0712660
F	2.7834488	-4.7292981	2.2299890
F	7.6098557	-0.2637839	-4.1202697
F	2.4514934	4.9632185	2.7259176
F	5.2511684	-4.6768459	3.4178018

2^{-}opo : $B(C_6F_5)_3$ adduct at one P of dianion **2**$^{-}$oo
108
Energy = -7538.449932290

P	0.1119217	-1.1120904	-1.1258303
C	-1.1481077	0.0732793	-0.4999620
O	-2.4157685	-0.1019369	-0.5383472
B	-3.4538670	0.9873812	-0.1564451
C	-3.0496407	1.4584563	1.3585018
C	-3.5080711	2.1874542	-1.2795621
C	-4.9062643	0.2202610	-0.2808997
C	-2.5586520	2.7058188	1.7502199
C	-3.0701074	0.5018557	2.3783049
C	-4.4203932	3.2261401	-1.0735489
C	-2.8415881	2.2437078	-2.5055049
C	-5.2054183	-0.4820396	-1.4533360
C	-5.9465667	0.2574441	0.6488760

C	-2.1190664	2.9871638	3.0422636
C	-2.6465566	0.7380860	3.6794549
C	-4.6434557	4.2635901	-1.9684280
C	-3.0382007	3.2641692	-3.4381835
C	-6.4146642	-1.1312354	-1.6843628
C	-7.1699376	-0.3832207	0.4617251
C	-2.1590174	1.9965388	4.0142637
C	-3.9391268	4.2849455	-3.1677674
C	-7.4060650	-1.0879472	-0.7117330
F	-2.4523441	3.7315181	0.8707482
F	-3.5171043	-0.7483525	2.1136363
F	-5.1545384	3.2500342	0.0689483
F	-1.9630353	1.2874096	-2.8801270
F	-4.3019326	-0.5391116	-2.4588389
F	-5.8268659	0.9428601	1.8121517
F	-1.6359519	4.2095302	3.3536325
F	-2.6937044	-0.2326422	4.6137588
F	-5.5345857	5.2432957	-1.6947670
F	-2.3757386	3.2588666	-4.6131982
F	-6.6412337	-1.7915695	-2.8395442
F	-8.1345627	-0.3211862	1.4075305
F	-1.7313815	2.2511337	5.2655348
F	-4.1422953	5.2751086	-4.0611920
F	-8.5863039	-1.7103583	-0.9098869
B	0.2401430	-2.8354052	0.0377905
C	1.1270994	-2.4394183	1.3436490
C	-1.3105872	-3.2619110	0.3498049
C	0.9485124	-3.9405258	-0.9502243
C	0.6197552	-1.5489128	2.2981217
C	2.4232559	-2.8820576	1.6248468
C	-1.8058129	-3.6754856	1.5893009
C	-2.2646069	-3.2828395	-0.6715761
C	1.9832721	-3.5993068	-1.8277376
C	0.5832333	-5.2875944	-1.0071395
C	1.2985608	-1.1376431	3.4370577
C	3.1571483	-2.4714683	2.7362278
C	-3.1379295	-4.0061246	1.8215455
C	-3.5985092	-3.6295268	-0.4946539
C	2.5843488	-4.4825826	-2.7163756
C	1.1535828	-6.2108514	-1.8825891
C	2.5927420	-1.5984980	3.6560481
C	-4.0484097	-3.9751520	0.7734169
C	2.1591825	-5.8063638	-2.7500818
F	-0.6413828	-1.0785913	2.1585018
F	3.0525085	-3.7819787	0.8264918
F	-0.9802269	-3.8224685	2.6569850
F	-1.8969536	-2.9670448	-1.9413487
F	2.4936623	-2.3445944	-1.8086224
F	-0.3541955	-5.7962747	-0.1652469
F	0.7149171	-0.3168961	4.3328095
F	4.4125612	-2.9306369	2.9389414
F	-3.5524443	-4.3761418	3.0542216
F	-4.4583242	-3.6402428	-1.5366388
F	3.5816367	-4.0788434	-3.5326769
F	0.7470495	-7.5020474	-1.8877945
F	3.2871294	-1.2107488	4.7465993
F	-5.3446560	-4.2893888	0.9786523

F	2.7239513	-6.6869355	-3.6031864
P	-0.1567174	1.4975964	-0.0433404
C	1.1002315	0.2659220	-0.3926611
O	2.3734479	0.2625431	-0.2929986
B	3.4127717	1.4416322	-0.2685782
C	4.5381459	0.8480598	-1.3092011
C	2.6445992	2.7786461	-0.8138461
C	4.0122731	1.5734562	1.2532090
C	5.0847914	1.5160055	-2.4025186
C	5.0034871	-0.4601914	-1.1374669
C	1.9665620	2.7261257	-2.0390226
C	2.5132172	3.9941238	-0.1410968
C	5.3714832	1.6308669	1.5678621
C	3.1685685	1.6819068	2.3615601
C	5.9752517	0.9285632	-3.2989165
C	5.8838982	-1.0901526	-2.0106602
C	1.1953759	3.7617268	-2.5508065
C	1.7390633	5.0547427	-0.6083361
C	5.8608113	1.7459833	2.8681696
C	3.6079087	1.8192812	3.6735272
C	6.3709901	-0.3890229	-3.1083708
C	1.0668634	4.9357842	-1.8176536
C	4.9739886	1.8382035	3.9316190
F	4.7877572	2.8195145	-2.6488030
F	4.6356472	-1.1734107	-0.0505838
F	2.0443285	1.6097440	-2.8013604
F	3.1538896	4.2237893	1.0328450
F	6.3248070	1.6084077	0.6003778
F	1.8283721	1.7144063	2.1790778
F	6.4627022	1.6280619	-4.3496936
F	6.2899540	-2.3587155	-1.7951576
F	0.5601992	3.6373247	-3.7318272
F	1.6453609	6.2025749	0.0974361
F	7.1921165	1.7830434	3.1028986
F	2.7360427	1.9555191	4.6908195
F	7.2318402	-0.9739056	-3.9669573
F	0.3127005	5.9498091	-2.2814486
F	5.4301267	1.9594229	5.1936805

2¯ppo : B(C_6F_5)$_3$ adduct at one O of dianion **2**¯pp
108
Energy = -7538.436253959

P	0.9861514	1.3625990	-0.1610318
C	0.0505559	-0.1420653	-0.1677349
O	0.4175874	-1.2824590	0.2804149
B	0.0103045	-2.7483331	-0.0424051
C	-0.8786977	-2.7712753	-1.4126604
C	1.4846962	-3.4939891	-0.1757700
C	-0.7112207	-3.4654551	1.2449186
C	-2.1743617	-3.2620260	-1.5689303
C	-0.3349193	-2.2254192	-2.5790735
C	1.9066692	-4.3429751	-1.1994533
C	2.4017115	-3.3606621	0.8738858
C	-0.9434704	-4.8426501	1.1967834
C	-1.0629610	-2.8646586	2.4523937
C	-2.8804603	-3.2082601	-2.7704220
C	-0.9935367	-2.1448262	-3.7964036

C	3.1424283	-4.9881692	-1.2087317
C	3.6452669	-3.9852454	0.9055296
C	-1.5066597	-5.5770602	2.2319439
C	-1.6278757	-3.5630868	3.5214921
C	-2.2905553	-2.6401771	-3.8910878
C	4.0215494	-4.8088548	-0.1490842
C	-1.8578389	-4.9273388	3.4110747
F	-2.8449764	-3.8241553	-0.5360805
F	0.9226262	-1.7287258	-2.5416102
F	1.1094669	-4.6201758	-2.2610496
F	2.0881423	-2.6213973	1.9612731
F	-0.6318216	-5.5352960	0.0703668
F	-0.8664211	-1.5492593	2.6570455
F	-4.1351479	-3.6956342	-2.8516432
F	-0.4051334	-1.5902571	-4.8736261
F	3.4911871	-5.7957895	-2.2350860
F	4.4818879	-3.8128067	1.9507407
F	-1.7228805	-6.9066703	2.1081619
F	-1.9484138	-2.9245944	4.6672917
F	-2.9621332	-2.5733991	-5.0550748
F	5.2170786	-5.4299949	-0.1407649
F	-2.4059114	-5.6163016	4.4331879
B	3.0278326	1.6788887	-0.3863199
C	3.6040158	1.9752112	1.1138658
C	3.6291777	0.3626606	-1.1190097
C	3.0477597	3.0499935	-1.2884367
C	3.1424303	1.2914617	2.2421317
C	4.5775397	2.9386971	1.3873165
C	4.5958424	-0.4918398	-0.5801554
C	3.2042069	0.0066076	-2.4057465
C	2.3004832	4.1664227	-0.9016757
C	3.8110744	3.2563183	-2.4414156
C	3.5559340	1.5612530	3.5417752
C	5.0230164	3.2441232	2.6711937
C	5.1101352	-1.5967194	-1.2554850
C	3.6933736	-1.0813810	-3.1164466
C	2.2433508	5.3624721	-1.6067991
C	3.7878521	4.4352235	-3.1818068
C	4.5030584	2.5556283	3.7599898
C	4.6537283	-1.8995875	-2.5309841
C	2.9909799	5.4963796	-2.7698608
F	2.2592805	0.2793009	2.0950968
F	5.1779913	3.6287133	0.3818236
F	5.1270487	-0.2716904	0.6493113
F	2.2782398	0.7663018	-3.0335320
F	1.5784010	4.1241518	0.2463399
F	4.6797350	2.3140894	-2.8869007
F	3.0607565	0.8696166	4.5873363
F	5.9662567	4.1937858	2.8695030
F	6.0620887	-2.3693770	-0.6854826
F	3.2548151	-1.3553947	-4.3608259
F	1.4807446	6.3868214	-1.1746251
F	4.5527257	4.5681459	-4.2892595
F	4.9232924	2.8383344	5.0103762
F	5.1384834	-2.9681287	-3.1946462
F	2.9603667	6.6438304	-3.4776871
P	-1.4739934	0.4226505	-1.0442474
C	-0.3440928	1.9019884	-1.3229044
O	-0.4660431	2.8840419	-2.0199491
B	-2.9224124	1.2668880	0.2570648
C	-2.1380693	2.4926727	0.9749476
C	-4.2048254	1.7672650	-0.6537997
C	-3.5299600	0.0948867	1.2278846
C	-1.1681903	2.2728806	1.9645268
C	-2.2725989	3.8410332	0.6156130
C	-5.3323086	2.2377723	0.0296945
C	-4.3548341	1.7468799	-2.0404983
C	-3.7046677	0.1671890	2.6145905
C	-4.0993005	-1.0460058	0.6473557
C	-0.4496292	3.2817792	2.5981804
C	-1.5649354	4.8786482	1.2147080
C	-6.5177037	2.6266687	-0.5829854
C	-5.5248072	2.1232672	-2.6996707
C	-4.3015809	-0.8384373	3.3712398
C	-4.7283607	-2.0625317	1.3595600
C	-0.6508702	4.6021395	2.2235842
C	-6.6190402	2.5635265	-1.9679332
C	-4.8083776	-1.9682654	2.7438088
F	-0.9181066	1.0161021	2.3850322
F	-3.1321273	4.2275774	-0.3620175
F	-5.2972079	2.3650317	1.3810543
F	-3.3397174	1.3647641	-2.8518452
F	-3.3553479	1.2709088	3.3160922
F	-4.0989935	-1.1770970	-0.7022136
F	0.4608465	2.9861199	3.5502010
F	-1.7551085	6.1582676	0.8205690
F	-7.5658601	3.0735019	0.1463165
F	-5.6040599	2.0674954	-4.0475134
F	-4.4228953	-0.7103159	4.7107125
F	-5.2842302	-3.1190794	0.7331310
F	0.0564141	5.5955199	2.7981206
F	-7.7591673	2.9316523	-2.5897116
F	-5.3939725	-2.9479052	3.4629945

2¯pp : C_2P_2-ring dimer of Bf_3PCO^-
74
Energy = -5328.859070297

P	-1.1586804	0.3705496	-0.7348235
C	0.1208564	-0.9875368	-0.6679498
O	0.2706096	-1.9706775	-1.3725803
B	2.9681440	0.2027908	-0.0840241
C	2.4851202	1.2570654	-1.2331404
C	3.9972828	0.8055526	1.0482067
C	3.7284728	-1.1469255	-0.6486880
C	2.0948676	2.5620583	-0.9029208
C	2.2313650	0.9155412	-2.5651264
C	4.8507953	1.8994190	0.8589731
C	4.1714313	0.1631285	2.2809881
C	3.4495843	-2.4732241	-0.3101580
C	4.8425252	-0.9950457	-1.4812269
C	1.5166529	3.4558660	-1.7954962
C	1.6567649	1.7807916	-3.4939068
C	5.7493153	2.3592001	1.8197515
C	5.0548531	0.5897591	3.2689990

C	4.1865741	-3.5605663	-0.7817792
C	5.6046350	-2.0470739	-1.9737660
C	1.2962478	3.0645160	-3.1109814
C	5.8504208	1.7054131	3.0405273
C	5.2714787	-3.3497790	-1.6207805
F	2.2616366	3.0165204	0.3632634
F	2.5304395	-0.3220313	-3.0380823
F	4.8658864	2.5864661	-0.3086392
F	3.4755942	-0.9615084	2.5683719
F	2.4333540	-2.7899327	0.5201210
F	5.2342676	0.2477900	-1.8610388
F	1.1689073	4.7017735	-1.4025600
F	1.4450445	1.3798162	-4.7693111
F	6.5371842	3.4328443	1.5728229
F	5.1577937	-0.0752517	4.4430796
F	3.8612739	-4.8222166	-0.4205336
F	6.6645503	-1.8232859	-2.7875996
F	0.7311652	3.9151010	-3.9961768
F	6.7177618	2.1371632	3.9826090
F	5.9992991	-4.3916071	-2.0805049
P	1.1479637	-0.3250615	0.7283849
C	-0.1337956	1.0306574	0.6655097
O	-0.2911196	2.0050062	1.3804776
B	-2.9726106	-0.1957766	0.0704454
C	-4.0461970	-0.7012828	-1.0692070
C	-2.4739192	-1.3408582	1.1220743
C	-3.6956833	1.1205495	0.7507325
C	-4.9332257	-1.7735647	-0.9116612
C	-4.2385595	0.0160427	-2.2570861
C	-2.1364084	-2.6294204	0.6859872
C	-2.1492617	-1.0989611	2.4606950
C	-4.7524652	0.9313171	1.6478904
C	-3.4327826	2.4611654	0.4583400
C	-5.8877394	-2.1391528	-1.8587228
C	-5.1770925	-0.3161460	-3.2305425
C	-1.5431225	-3.5967981	1.4877125
C	-1.5553421	-2.0389593	3.2995888
C	-5.4678624	1.9609894	2.2467017
C	-4.1274161	3.5265065	1.0325048
C	-6.0106448	-1.4095060	-3.0335418
C	-1.2494496	-3.3021921	2.8138680
C	-5.1501190	3.2784103	1.9370365
F	-4.9303024	-2.5297040	0.2126431
F	-3.5036223	1.1226849	-2.5140691
F	-2.3702137	-2.9915772	-0.5997137
F	-2.3901312	0.1114050	3.0279539
F	-5.1310221	-0.3265932	1.9890844
F	-2.4771423	2.8135980	-0.4279936
F	-6.7074387	-3.1954949	-1.6427349
F	-5.2978306	0.4210967	-4.3589103
F	-1.2474295	-4.8201919	0.9938693
F	-1.2696921	-1.7309657	4.5861539
F	-6.4685878	1.7007144	3.1222574
F	-3.8211989	4.8032551	0.7093766
F	-6.9324625	-1.7485493	-3.9616453
F	-0.6649702	-4.2240064	3.6108084
F	-5.8345782	4.2989002	2.4998133

3a : the open $Al(C_6F_5)_3$ adduct at oxygen of **1**

110

Energy = -6199.096340426

Ge	-3.3877640	0.4845582	-1.7803589
P	-0.8462787	1.0156089	-2.5390102
N	-2.7097006	-1.0225271	-0.6740941
N	-3.2157244	1.7640150	-0.2727276
C	-1.6494299	1.1023688	-3.9231169
C	-2.1448681	-0.9059691	0.5353235
C	-2.9382663	-2.3435865	-1.2273411
C	-2.4826976	1.5639251	0.8351259
C	-3.9823865	2.9877460	-0.4088173
O	-2.2492884	1.1681498	-4.9841283
C	-1.9224302	0.3261542	1.1646158
C	-1.7937158	-2.1518362	1.3047139
C	-4.2559771	-2.8476530	-1.2376617
C	-1.8590405	-3.0964740	-1.7425735
C	-2.2983801	2.6940810	1.8137666
C	-5.3584658	2.9277543	-0.0898782
C	-3.3723264	4.1852426	-0.8296699
H	-1.3909229	0.2852611	2.1071658
H	-1.4464934	-1.8941659	2.3057742
H	-2.6728522	-2.8002319	1.3812257
H	-1.0199070	-2.7296204	0.7962885
C	-4.4702508	-4.1374270	-1.7335943
C	-5.4449722	-2.0560954	-0.7199435
C	-2.1301824	-4.3825739	-2.2202265
C	-0.4331517	-2.5730331	-1.8438210
H	-3.2337828	3.2351470	1.9757318
H	-1.9243993	2.3149865	2.7656545
H	-1.5737205	3.4120186	1.4170610
C	-6.1011501	4.1090590	-0.1537467
C	-6.0076289	1.6247975	0.3522231
C	-4.1657523	5.3378144	-0.8882780
C	-1.9110896	4.2785042	-1.2359831
C	-3.4168245	-4.9082147	-2.2088853
H	-5.4794392	-4.5393771	-1.7445091
C	-6.1173128	-2.7575064	0.4734821
C	-6.4546042	-1.7797924	-1.8483272
H	-5.0754947	-1.0916565	-0.3548714
H	-1.3142282	-4.9769714	-2.6210605
C	-0.0363629	-2.3854019	-3.3208760
C	0.5916280	-3.5056218	-1.1690423
H	-0.3876135	-1.5934324	-1.3551844
C	-5.5101240	5.3090035	-0.5399918
H	-7.1575670	4.0894687	0.0899610
C	-7.4983445	1.5346437	0.0025240
C	-5.7965460	1.3724051	1.8573787
H	-5.5012464	0.8147700	-0.1892715
H	-3.7164204	6.2717845	-1.2134350
C	-1.1594344	5.3853645	-0.4738389
C	-1.7939770	4.5223718	-2.7500139
H	-1.4322948	3.3176036	-1.0167762
H	-3.6009530	-5.9075577	-2.5915306
H	-5.4052920	-2.9196801	1.2893088
H	-6.9445412	-2.1483724	0.8536499

H	-6.5229676	-3.7309973	0.1785594
H	-5.9879914	-1.2148458	-2.6606816
H	-6.8419918	-2.7170314	-2.2616538
H	-7.3023078	-1.1995900	-1.4709810
H	-0.7254415	-1.7167923	-3.8373191
H	0.9724088	-1.9636260	-3.3876982
H	-0.0406207	-3.3502412	-3.8397690
H	0.3470399	-3.7195555	-0.1239364
H	0.6506275	-4.4632515	-1.6968299
H	1.5854396	-3.0467375	-1.1987586
H	-6.1052997	6.2155716	-0.5904462
H	-7.6722686	1.7155190	-1.0609572
H	-7.8707164	0.5355118	0.2506454
H	-8.0908989	2.2533134	0.5785146
H	-4.7359778	1.3223868	2.1167390
H	-6.2558505	2.1754570	2.4441655
H	-6.2605935	0.4242584	2.1502925
H	-1.2634917	5.2851768	0.6110400
H	-0.0937559	5.3509522	-0.7232059
H	-1.5350135	6.3754221	-0.7529683
H	-2.3070834	3.7406076	-3.3117092
H	-2.2382295	5.4865797	-3.0198333
H	-0.7426019	4.5298153	-3.0554929
Al	-3.9577199	1.2622033	-5.7540387
C	-3.5582171	1.6058023	-7.6783110
C	-4.8431448	-0.5187215	-5.6030482
C	-4.8404195	2.7784585	-4.8030998
C	-2.5891808	0.8531961	-8.3349967
C	-4.2297964	2.5292309	-8.4734301
C	-4.2511984	-1.7322902	-5.2749296
C	-6.1744492	-0.5948288	-6.0005944
C	-5.9873196	2.6950873	-4.0182557
C	-4.3898287	4.0716616	-5.0542963
C	-2.2723993	1.0027876	-9.6811438
F	-1.9030354	-0.0974774	-7.6418567
C	-3.9553879	2.7222182	-9.8245706
F	-5.2171592	3.2945488	-7.9386459
C	-4.9220401	-2.9510545	-5.3351913
F	-2.9496693	-1.7690044	-4.8890203
C	-6.9058101	-1.7760834	-6.0425202
F	-6.8073021	0.5450157	-6.3956509
C	-6.6791349	3.8104137	-3.5551864
F	-6.4960208	1.4838820	-3.6748246
C	-5.0226292	5.2183201	-4.5913307
F	-3.2646661	4.2472016	-5.7999956
C	-2.9648135	1.9520903	-10.4295146
F	-1.3205376	0.2515721	-10.2703398
F	-4.6293128	3.6325623	-10.5549466
C	-6.2645724	-2.9659391	-5.7044977
F	-4.3012626	-4.1127426	-5.0580612
F	-8.2000592	-1.7960805	-6.4161877
C	-6.1896848	5.0804467	-3.8452797
F	-7.8124796	3.6849779	-2.8357922
F	-4.5325555	6.4485384	-4.8444754
F	-2.6826932	2.1196432	-11.7323549
F	-6.9364265	-4.1296283	-5.7461720
F	-6.8349945	6.1701730	-3.3973841

3_ts : ring-closing transition state from **3a** to **3**
110
Energy = -6199.086966939

Ge	-3.6051156	0.0561367	-1.0172901
P	-1.2561660	0.4593717	-2.1963498
N	-3.1008782	-1.4442149	0.1447203
N	-3.3120083	1.3794420	0.4217303
C	-0.2208568	0.1397958	-1.0173057
C	-2.2522278	-1.3180318	1.1823690
C	-3.6828796	-2.7276597	-0.1981835
C	-2.5504396	1.1570329	1.4999005
C	-3.9703237	2.6519242	0.2012143
O	0.6584843	-0.1104236	-0.2003164
C	-1.9448727	-0.0836038	1.7541647
C	-1.5877441	-2.5417427	1.7616205
C	-4.8944863	-3.0893971	0.4309269
C	-3.0731102	-3.5592383	-1.1557167
C	-2.3211866	2.2502268	2.5110217
C	-5.3670236	2.7217210	0.4080370
C	-3.2349223	3.7677862	-0.2478739
H	-1.2528369	-0.1055404	2.5870374
H	-1.5563232	-2.4902392	2.8520363
H	-2.0815829	-3.4628889	1.4526661
H	-0.5517998	-2.5692508	1.4002977
C	-5.4792718	-4.3104785	0.0813705
C	-5.5376420	-2.2160573	1.4961592
C	-3.7036505	-4.7673534	-1.4768813
C	-1.7733979	-3.1930461	-1.8460601
H	-3.0119926	3.0822513	2.3774415
H	-2.4211029	1.8479852	3.5222350
H	-1.2974936	2.6238800	2.4096954
C	-6.0092395	3.9406207	0.1756296
C	-6.1508860	1.5302678	0.9351841
C	-3.9279780	4.9674250	-0.4552106
C	-1.7455916	3.7333990	-0.5464480
C	-4.8940241	-5.1444038	-0.8669722
H	-6.4064161	-4.6129057	0.5579857
C	-5.1418433	-2.6938861	2.9070543
C	-7.0663698	-2.1551990	1.3606899
H	-5.1459600	-1.2009912	1.3748303
H	-3.2487498	-5.4199528	-2.2164728
C	-2.0237024	-2.8691499	-3.3299768
C	-0.7171191	-4.3010855	-1.7042699
H	-1.3789134	-2.2882876	-1.3747747
C	-5.2973552	5.0602355	-0.2465875
H	-7.0798108	4.0177799	0.3317428
C	-7.6289793	1.5442916	0.5260501
C	-6.0103904	1.4333318	2.4668067
H	-5.7057783	0.6223506	0.5035483
H	-3.3765771	5.8388482	-0.7963560
C	-0.9670366	4.7690020	0.2863829
C	-1.5041158	3.9836770	-2.0473587
H	-1.3609138	2.7370792	-0.3097176
H	-5.3665775	-6.0869277	-1.1286163
H	-4.0567044	-2.6924150	3.0404824
H	-5.5813001	-2.0394232	3.6677833

H	-5.5024925	-3.7141181	3.0795517
H	-7.3640620	-1.8295997	0.3591876
H	-7.5259451	-3.1300324	1.5526640
H	-7.4788876	-1.4531320	2.0921008
H	-2.7573876	-2.0630694	-3.4399368
H	-1.0924068	-2.5531989	-3.8106182
H	-2.4072246	-3.7494661	-3.8582873
H	-0.5055450	-4.5187282	-0.6523366
H	-1.0458912	-5.2295467	-2.1828321
H	0.2109366	-3.9896773	-2.1892830
H	-5.8136098	6.0004235	-0.4182653
H	-7.7414473	1.6588835	-0.5564464
H	-8.1036504	0.6054990	0.8243012
H	-8.1744094	2.3567620	1.0173292
H	-4.9654705	1.3196356	2.7680470
H	-6.4037651	2.3403977	2.9387801
H	-6.5712891	0.5756098	2.8516110
H	-1.1366449	4.6393589	1.3590376
H	0.1046267	4.6716508	0.0906509
H	-1.2673296	5.7882574	0.0206274
H	-2.0430776	3.2563365	-2.6617092
H	-1.8450503	4.9860316	-2.3293519
H	-0.4379556	3.9066456	-2.2761845
Al	2.5314095	-0.0762559	-0.0839496
C	3.0764455	1.0385417	-1.6468836
C	3.0207613	0.8647033	1.6027811
C	2.9811561	-2.0126151	-0.1695718
C	2.5247899	2.3084335	-1.7741996
C	3.9870252	0.6851187	-2.6372503
C	2.1839306	1.4432120	2.5457241
C	4.3812735	1.0360737	1.8363353
C	3.6107953	-2.7467601	0.8309906
C	2.6963572	-2.7180176	-1.3350066
C	2.8197245	3.1847483	-2.8117253
F	1.6399922	2.7406234	-0.8289654
C	4.3222310	1.5209740	-3.6997123
F	4.6071543	-0.5207558	-2.5916383
C	2.6445847	2.1485878	3.6535801
F	0.8325715	1.3378702	2.4116257
C	4.9032268	1.7273159	2.9235123
F	5.2655566	0.4998422	0.9491640
C	3.9535227	-4.0903870	0.6940145
F	3.9189784	-2.1582206	2.0149185
C	3.0242357	-4.0541326	-1.5321845
F	2.0567358	-2.0782931	-2.3537921
C	3.7288110	2.7787168	-3.7864266
F	2.2511643	4.4051215	-2.8911335
F	5.2083338	1.1398493	-4.6401096
C	4.0174114	2.2897444	3.8415028
F	1.7882842	2.6946665	4.5414265
F	6.2300995	1.8648238	3.1050460
C	3.6593800	-4.7445294	-0.5008630
F	4.5597958	-4.7641573	1.6887781
F	2.7364428	-4.6902265	-2.6839658
F	4.0374649	3.6002740	-4.8030912
F	4.4857098	2.9652973	4.9032502
F	3.9793676	-6.0382621	-0.6561640

3 : ring-closed Al(C_6F_5)$_3$ adduct at oxygen of **1**
110
Energy = -6199.091731190

Ge	-3.0130935	0.7655311	-1.2299329
P	-0.6021382	0.5805595	-1.4111067
N	-3.0888665	-1.1262887	-0.3505884
N	-2.7865231	1.5779256	0.6764036
C	-0.2970493	-0.0325617	0.1641367
C	-2.3526204	-1.4122452	0.6726101
C	-3.8436611	-2.1490522	-1.0509147
C	-2.1282669	0.9424343	1.5918925
C	-3.1794016	2.9602678	0.8527043
O	0.8384002	-0.3204689	0.7281826
C	-1.4565827	-0.3468142	1.2237323
C	-2.2545147	-2.7671842	1.2927535
C	-5.2049745	-2.3242674	-0.7446292
C	-3.1874842	-2.9121704	-2.0371374
C	-1.9048629	1.4447399	2.9805711
C	-4.5469127	3.2797605	0.9677668
C	-2.1731267	3.9504994	0.8476066
H	-0.9436364	-0.7378549	2.1019087
H	-2.4720580	-2.7012543	2.3645003
H	-2.9250043	-3.4836624	0.8192838
H	-1.2187318	-3.1164762	1.1988284
C	-5.9113042	-3.3025858	-1.4513317
C	-5.8923134	-1.5350838	0.3551556
C	-3.9470679	-3.8670252	-2.7235260
C	-1.7221866	-2.7274141	-2.3928777
H	-2.4268052	2.3829511	3.1664380
H	-2.2345740	0.6839323	3.6975367
H	-0.8289944	1.5823054	3.1426986
C	-4.8928370	4.6253668	1.1139442
C	-5.6090166	2.1953172	0.9938764
C	-2.5802602	5.2822433	0.9948521
C	-0.6973832	3.6616464	0.6239541
C	-5.2928902	-4.0657548	-2.4370810
H	-6.9595058	-3.4701209	-1.2229289
C	-6.0636748	-2.3992290	1.6198303
C	-7.2476970	-0.9764871	-0.1029892
H	-5.2476247	-0.6886338	0.6139300
H	-3.4667954	-4.4647918	-3.4925671
C	-1.5894953	-2.0467205	-3.7677769
C	-0.9432249	-4.0532025	-2.3595225
H	-1.2634338	-2.0566547	-1.6607325
C	-3.9197250	5.6212852	1.1335235
H	-5.9375023	4.8989700	1.2126319
C	-6.9734561	2.6839393	0.4903123
C	-5.7339730	1.5982516	2.4094728
H	-5.2726082	1.3976085	0.3168782
H	-1.8251872	6.0628220	0.9907946
C	0.1952301	4.2694551	1.7195973
C	-0.2716990	4.1705215	-0.7663609
H	-0.5399490	2.5800671	0.6236667
H	-5.8601309	-4.8167309	-2.9793137
H	-5.1028558	-2.7767000	1.9820871
H	-6.5280185	-1.8151689	2.4218039

H	-6.7051546	-3.2616974	1.4088475
H	-7.1333660	-0.3306379	-0.9787421
H	-7.9430279	-1.7815306	-0.3604967
H	-7.7061991	-0.3958343	0.7033159
H	-2.1011024	-1.0780048	-3.7801148
H	-0.5351066	-1.8783379	-4.0058249
H	-2.0278797	-2.6715752	-4.5540046
H	-1.0395283	-4.5471714	-1.3876739
H	-1.2962426	-4.7452564	-3.1315447
H	0.1182723	-3.8591872	-2.5385963
H	-4.2095248	6.6618061	1.2479852
H	-6.8881400	3.1516419	-0.4954407
H	-7.6652862	1.8417171	0.4120760
H	-7.4192534	3.4091105	1.1793386
H	-4.7878281	1.1668934	2.7502890
H	-6.0274123	2.3752587	3.1233756
H	-6.4940811	0.8107894	2.4296826
H	-0.0913379	3.9221797	2.7169828
H	1.2367523	3.9840453	1.5425478
H	0.1437516	5.3632344	1.7153167
H	-0.8755846	3.7086877	-1.5538449
H	-0.3931935	5.2575561	-0.8331385
H	0.7769410	3.9237163	-0.9531997
Al	2.5988270	-0.3349882	0.3147712
C	2.6995649	0.0789390	-1.6461041
C	3.4045327	1.0758069	1.4979084
C	3.1852025	-2.1884614	0.8258700
C	2.7382158	1.3823840	-2.1338022
C	2.5361507	-0.9039147	-2.6175099
C	2.8172994	1.5658123	2.6586467
C	4.6140108	1.6756298	1.1582853
C	4.4683351	-2.4638888	1.2885503
C	2.3371705	-3.2904216	0.7734717
C	2.6169809	1.7103724	-3.4793425
F	2.8530621	2.4260308	-1.2681481
C	2.4060382	-0.6355590	-3.9760890
F	2.4564113	-2.2126611	-2.2511783
C	3.3588919	2.5922028	3.4277051
F	1.6382388	1.0439431	3.1101026
C	5.2063457	2.7005071	1.8888008
F	5.2738614	1.2576288	0.0459949
C	4.8969439	-3.7282819	1.6848730
F	5.3849460	-1.4622431	1.3643398
C	2.7072009	-4.5751824	1.1567063
F	1.0594807	-3.1490584	0.3162881
C	2.4447658	0.6872613	-4.4082833
F	2.6428474	2.9924135	-3.8968889
F	2.2303438	-1.6264890	-4.8757555
C	4.5654529	3.1645979	3.0356745
F	2.7353777	3.0402499	4.5389313
F	6.3776490	3.2505675	1.5119729
C	4.0028242	-4.7934197	1.6183809
F	6.1529083	-3.9390892	2.1269305
F	1.8387146	-5.6069315	1.0877159
F	2.3184187	0.9746302	-5.7150134
F	5.1108313	4.1558285	3.7618843
F	4.3877855	-6.0253564	1.9937926

4 : $GePC_3$-ring adduct of **1** and the alkyne But

104

Energy = -4462.748463444

Ge	-0.7296213	0.0842295	-0.1661355
P	-0.9541622	0.3758372	-2.3602008
C	0.8136969	-0.0418157	-2.5399540
O	1.3816682	-0.0599455	-3.6426911
N	-1.7390684	-1.2731361	0.7732604
C	-2.1480070	-1.1271142	2.0411631
C	-2.0302347	-2.4701426	0.0075844
C	-2.0104360	0.0621610	2.7671037
C	-2.7950436	-2.2923777	2.7406397
C	-3.1174645	-2.4279024	-0.8922065
C	-1.2128667	-3.6087766	0.1352016
C	-1.5968527	1.3101668	2.2986490
H	-2.3534007	0.0303783	3.7937055
H	-3.4845709	-1.9377119	3.5086724
H	-3.3196762	-2.9455989	2.0415971
H	-2.0169944	-2.8866162	3.2328473
C	-3.3809213	-3.5680699	-1.6548007
C	-4.0058755	-1.1994176	-1.0039336
C	-1.5208376	-4.7241654	-0.6540838
C	-0.0227627	-3.6803883	1.0773048
C	-1.6940597	2.4834207	3.2364029
N	-1.1264038	1.5188988	1.0566801
C	-2.5917968	-4.7097653	-1.5383482
H	-4.2092835	-3.5611401	-2.3554385
C	-5.1433531	-1.2395130	0.0345838
C	-4.5672498	-0.9902479	-2.4157478
H	-3.3838792	-0.3268172	-0.7734628
H	-0.9020100	-5.6134982	-0.5734268
C	1.2878387	-3.8884390	0.2955109
C	-0.1867172	-4.8136365	2.1091665
H	0.0483223	-2.7324479	1.6175137
H	-2.0809372	3.3710290	2.7309351
H	-2.3277117	2.2378199	4.0894934
H	-0.6934876	2.7328143	3.6059234
C	-0.9401199	2.8652879	0.5514017
H	-2.8097066	-5.5846009	-2.1445533
H	-4.7559201	-1.2809178	1.0563829
H	-5.7683312	-0.3440388	-0.0543809
H	-5.7776572	-2.1184511	-0.1256009
H	-3.7609333	-0.9703917	-3.1541777
H	-5.2801136	-1.7758359	-2.6888799
H	-5.1001806	-0.0352927	-2.4614068
H	1.4439252	-3.1179109	-0.4616282
H	2.1409647	-3.8735160	0.9825565
H	1.2832879	-4.8603032	-0.2104042
H	-1.1287117	-4.7359739	2.6600486
H	-0.1694577	-5.7917585	1.6159744
H	0.6380532	-4.7860837	2.8296267
C	-1.9938851	3.4415161	-0.1898140
C	0.2684189	3.5472012	0.7789729
C	-1.8290183	4.7484527	-0.6551477
C	-3.2823991	2.6818983	-0.4627115
C	0.3832727	4.8545748	0.2903800

C	1.4551436	2.9144561	1.4854686
C	-0.6549078	5.4563786	-0.4099011
H	-2.6257824	5.2163266	-1.2245582
C	-3.8495279	2.9630249	-1.8607588
C	-4.3413888	2.9583469	0.6215937
H	-3.0442983	1.6130368	-0.4202765
H	1.3070530	5.4015298	0.4573730
C	1.8941283	3.7189468	2.7228104
C	2.6384723	2.7767024	0.5080836
H	1.1607718	1.9145023	1.8179237
H	-0.5455258	6.4717799	-0.7805005
H	-3.0978681	2.7666753	-2.6304730
H	-4.7112811	2.3137696	-2.0453803
H	-4.1932206	3.9984220	-1.9577906
H	-3.9929937	2.6556211	1.6130724
H	-4.5851624	4.0260504	0.6560136
H	-5.2600306	2.4031168	0.4025073
H	1.0760074	3.8530438	3.4372952
H	2.7141913	3.2032524	3.2339499
H	2.2518359	4.7133787	2.4343208
H	2.3523684	2.2380170	-0.3969638
H	3.0053916	3.7659833	0.2124880
H	3.4636867	2.2354094	0.9802043
C	1.1802037	-0.3627773	-0.0711839
C	1.6843810	-0.3801108	-1.3199736
C	1.8741851	-0.6468640	1.1897307
C	3.0872947	-0.7680762	-1.6851059
O	1.2956610	-0.6776278	2.2729575
O	3.1952613	-0.8748268	1.0393442
O	3.4053729	-1.9154041	-1.9312075
O	3.8882496	0.3038967	-1.7340707
C	3.9511128	-1.2636826	2.2399722
C	4.3543418	-0.0135216	3.0095124
C	5.1289860	-2.0763230	1.7291331
H	3.2798615	-1.8773964	2.8479532
H	3.4705191	0.5450138	3.3268579
H	4.9227291	-0.2983988	3.9011995
H	4.9852642	0.6308595	2.3887953
H	4.7839430	-2.9276687	1.1365237
H	5.7814123	-1.4586609	1.1036250
H	5.7134641	-2.4491576	2.5763655
C	5.2869001	0.0686456	-2.1405289
C	6.1037365	1.1295725	-1.4226625
C	5.3659844	0.1442375	-3.6582422
H	5.5507071	-0.9344474	-1.7944543
H	5.9857808	1.0428018	-0.3385507
H	7.1634859	1.0075390	-1.6692522
H	5.7880142	2.1317869	-1.7296031
H	4.7177828	-0.6082228	-4.1135832
H	5.0552428	1.1340636	-4.0070246
H	6.3967346	-0.0357415	-3.9827384

5a : higher $B(C_6F_5)_3$ adduct at PCO oxygen of **4**
138
Energy = -6672.325034446

Ge	2.3188654	-0.4788624	0.0753463
P	0.1675759	-1.0643344	0.4377615
C	-0.2653062	0.5143481	-0.2051186
O	-1.4946746	0.9395189	-0.2978258
N	3.3595372	-1.6213599	-1.0231118
C	4.6831785	-1.7758041	-0.8241205
C	2.6336927	-2.3989410	-2.0130760
C	5.3467498	-1.3001169	0.3077522
C	5.4805753	-2.4948975	-1.8760271
C	2.1537793	-3.6691184	-1.6285128
C	2.3817380	-1.8594693	-3.2863328
C	4.7904092	-0.7332557	1.4636023
H	6.4145145	-1.4786115	0.3411824
H	6.4330720	-2.8377537	-1.4705987
H	4.9287955	-3.3383842	-2.2949422
H	5.6829254	-1.7984897	-2.6983815
C	1.4565915	-4.4172780	-2.5809668
C	2.3856504	-4.2384206	-0.2373057
C	1.6766231	-2.6495747	-4.2017012
C	2.8141578	-0.4610404	-3.6880969
C	5.7049014	-0.5127741	2.6371044
N	3.4984778	-0.3860675	1.5697691
C	1.2274566	-3.9193303	-3.8605554
H	1.0778147	-5.3990738	-2.3143226
C	3.5062718	-5.2961997	-0.2469361
C	1.0999821	-4.8216470	0.3687909
H	2.7166511	-3.4195049	0.4111409
H	1.4679496	-2.2539257	-5.1913242
C	1.5884161	0.3911617	-4.0666754
C	3.8264178	-0.4849155	-4.8478810
H	3.3089767	-0.0018562	-2.8273110
H	5.1517473	-0.4174144	3.5718738
H	6.4257657	-1.3295935	2.7116250
H	6.2671800	0.4140689	2.4765080
C	2.8998857	0.0972253	2.8036904
H	0.6762189	-4.5133995	-4.5825482
H	4.4499062	-4.8828627	-0.6146751
H	3.6728203	-5.6793369	0.7656231
H	3.2310138	-6.1381792	-0.8914235
H	0.3130805	-4.0648663	0.4282574
H	0.7217545	-5.6618975	-0.2211844
H	1.3018915	-5.1927720	1.3785734
H	0.8181925	0.3581644	-3.2934297
H	1.8773521	1.4364410	-4.2108059
H	1.1413114	0.0314512	-4.9994832
H	4.7158594	-1.0712878	-4.5974491
H	3.3765429	-0.9213286	-5.7461151
H	4.1445086	0.5349775	-5.0905901
C	2.1460417	-0.8126247	3.5783088
C	3.0201339	1.4552633	3.1520123
C	1.5297964	-0.3271058	4.7336088
C	2.0254755	-2.2812748	3.2016796
C	2.3875899	1.8838725	4.3256233
C	3.7822500	2.4710396	2.3174307
C	1.6507301	1.0074757	5.1097271
H	0.9346551	-0.9982823	5.3425648
C	0.6986172	-2.9065384	3.6526984
C	3.2200794	-3.0937882	3.7375044
H	2.0578022	-2.3465830	2.1069556

H	2.4667315	2.9277859	4.6149501
C	4.9731322	3.0620142	3.0968549
C	2.8483944	3.6079211	1.8618638
H	4.1714256	1.9700682	1.4262842
H	1.1527190	1.3642436	6.0061047
H	-0.1539349	-2.3278389	3.2855998
H	0.6167585	-3.9233033	3.2587113
H	0.6352848	-2.9722756	4.7437871
H	4.1716176	-2.7128833	3.3559301
H	3.2508464	-3.0515487	4.8315375
H	3.1290313	-4.1430385	3.4361654
H	5.6491729	2.2849976	3.4645425
H	5.5439801	3.7398649	2.4529836
H	4.6211243	3.6349784	3.9616515
H	1.9692442	3.2285350	1.3384680
H	2.4999753	4.1882776	2.7231728
H	3.3821999	4.2903561	1.1932670
C	2.0276160	1.2857621	-0.7148080
C	0.7029007	1.5443857	-0.7157191
C	3.1488457	2.0727411	-1.2330711
C	0.0576400	2.8054008	-1.2394791
O	4.3112952	1.6945940	-1.0957907
O	2.7903273	3.1857206	-1.8968395
O	-0.3831536	2.9071833	-2.3638247
O	0.0212237	3.7296552	-0.2742006
C	3.8770833	3.9323458	-2.5660736
C	4.5007436	4.9051358	-1.5765582
C	3.2407117	4.6016346	-3.7714640
H	4.6155318	3.1869830	-2.8737746
H	4.9500580	4.3678362	-0.7378118
H	5.2858756	5.4809994	-2.0775495
H	3.7478499	5.6029950	-1.1974577
H	2.7661125	3.8627321	-4.4226005
H	2.4865715	5.3302544	-3.4616816
H	4.0133343	5.1253654	-4.3434211
C	-0.7050692	4.9878277	-0.5664537
C	0.1766831	5.8962158	-1.4091933
C	-1.0576503	5.5683842	0.7911417
H	-1.6022820	4.7005955	-1.1216795
H	0.4021022	5.4322920	-2.3711377
H	-0.3459883	6.8411600	-1.5908006
H	1.1117268	6.1130229	-0.8824388
H	-1.6408145	4.8554500	1.3767899
H	-0.1489022	5.8275154	1.3444477
H	-1.6519509	6.4772195	0.6529061
B	-2.7525157	0.1043723	-0.0138747
C	-2.9789440	-0.0983106	1.6014754
C	-3.9785372	1.0694231	-0.5360471
C	-2.5756571	-1.2727362	-0.8988087
C	-2.2245478	0.4532973	2.6398460
C	-4.1158838	-0.7942353	2.0220000
C	-4.0208522	2.4002032	-0.1115993
C	-5.0454602	0.6917718	-1.3518541
C	-2.3554461	-1.1463857	-2.2758147
C	-2.5454096	-2.5896697	-0.4342586
C	-2.5375743	0.2775568	3.9883419
C	-4.4664285	-0.9932641	3.3511811
C	-5.0005769	3.3102462	-0.4935984
C	-6.0567844	1.5651087	-1.7503928
C	-2.1445694	-2.2181285	-3.1351132
C	-2.3516037	-3.6944182	-1.2608337
C	-3.6600928	-0.4556893	4.3499360
C	-6.0322403	2.8881451	-1.3253298
C	-2.1549585	-3.5106919	-2.6227462
F	-1.1348092	1.2204048	2.3995399
F	-4.9513448	-1.3208251	1.0923345
F	-3.0747907	2.8694555	0.7400056
F	-5.1602917	-0.5776049	-1.8114554
F	-2.3424187	0.0821170	-2.8456807
F	-2.6545194	-2.8747559	0.8854244
F	-1.7663986	0.8279953	4.9507275
F	-5.5708632	-1.6918898	3.6837032
F	-4.9657487	4.5890499	-0.0601686
F	-7.0583032	1.1402320	-2.5474126
F	-1.9430594	-2.0220686	-4.4542213
F	-2.3253674	-4.9425493	-0.7469717
F	-3.9731135	-0.6291022	5.6477210
F	-6.9967962	3.7457854	-1.7049723
F	-1.9738178	-4.5695716	-3.4328742

5 : stable $B(C_6F_5)_3$ adduct at PCO oxygen of **4**
138
Energy = -6672.326677797

Ge	2.2425359	0.0826902	-0.3855020
P	0.4287322	0.7422437	0.7937471
C	-0.5044379	-0.3462646	-0.2259750
O	-1.8008599	-0.4704352	-0.1450515
N	3.6972795	-0.6505422	0.5969776
C	4.9739047	-0.4602426	0.2232806
C	3.3228786	-1.3209986	1.8318753
C	5.3495465	0.4246189	-0.7918896
C	6.0556606	-1.2391934	0.9204995
C	3.1898831	-0.5272677	2.9921534
C	3.0302466	-2.6955715	1.8266097
C	4.5497840	1.3509242	-1.4751167
H	6.4109767	0.4769115	-1.0029105
H	6.9734160	-0.6518498	0.9842521
H	5.7477203	-1.5592027	1.9163748
H	6.2716344	-2.1383464	0.3304971
C	2.7821590	-1.1597688	4.1691822
C	3.5133897	0.9587524	2.9923760
C	2.6218139	-3.2778013	3.0327276
C	3.1494748	-3.5635058	0.5866658
C	5.2437849	2.3370348	-2.3746289
N	3.2121886	1.3987105	-1.3676578
C	2.5023921	-2.5235993	4.1929150
H	2.6697653	-0.5761765	5.0771245
C	4.9486912	1.2073253	3.4963215
C	2.5091366	1.7863904	3.8063358
H	3.4680850	1.3114242	1.9553024
H	2.3866471	-4.3377209	3.0549120
C	1.8136846	-4.2534039	0.2577551
C	4.2603415	-4.6171257	0.7607236
H	3.4223862	-2.9181009	-0.2540841

H	4.6378341	3.2271329	-2.5459101
H	6.2103097	2.6186320	-1.9523052
H	5.4291779	1.8619374	-3.3448792
C	2.4054650	2.4450639	-1.9779535
H	2.1750924	-2.9931039	5.1150255
H	5.6886772	0.6768894	2.8906226
H	5.1814796	2.2770913	3.4624117
H	5.0516822	0.8653172	4.5319043
H	1.4898152	1.6460425	3.4378100
H	2.5313597	1.5200708	4.8672438
H	2.7614433	2.8487133	3.7306178
H	0.9921865	-3.5389330	0.1772022
H	1.8887762	-4.7906153	-0.6930537
H	1.5518234	-4.9798106	1.0343113
H	5.2301492	-4.1549167	0.9694164
H	4.0231764	-5.2888995	1.5925918
H	4.3530721	-5.2239622	-0.1457379
C	1.9927263	3.5245597	-1.1659194
C	2.0086423	2.3283936	-3.3229751
C	1.1916455	4.5080446	-1.7500120
C	2.4194675	3.6480235	0.2885061
C	1.2078479	3.3454296	-3.8565215
C	2.3929232	1.1566268	-4.2093946
C	0.8043834	4.4255573	-3.0841016
H	0.8514630	5.3438206	-1.1490147
C	1.3625633	4.3385044	1.1616879
C	3.7798547	4.3606539	0.4135244
H	2.5503359	2.6324544	0.6819397
H	0.8878227	3.2749581	-4.8921137
C	3.2338515	1.6131594	-5.4168341
C	1.1411854	0.4074083	-4.7042763
H	2.9930755	0.4619833	-3.6152809
H	0.1703946	5.1958978	-3.5122548
H	0.3881464	3.8529351	1.0575530
H	1.6598680	4.2859698	2.2126938
H	1.2572869	5.3973468	0.9035575
H	4.5665849	3.8284000	-0.1288860
H	3.7169787	5.3772102	0.0106586
H	4.0772530	4.4254880	1.4658731
H	4.1337809	2.1543759	-5.1098718
H	3.5372012	0.7468944	-6.0151657
H	2.6503754	2.2769511	-6.0635614
H	0.4950096	0.1035757	-3.8787331
H	0.5525705	1.0434605	-5.3739996
H	1.4324312	-0.4893326	-5.2610751
C	1.3899437	-1.2219816	-1.5623114
C	0.0665724	-1.2375383	-1.2852729
C	2.0380463	-2.0688882	-2.5697807
C	-0.8900393	-2.2061283	-1.9379137
O	1.4961215	-2.9020365	-3.2752574
O	3.3730693	-1.7836919	-2.6184872
O	-1.1462675	-3.2789232	-1.4310219
O	-1.3627935	-1.7257638	-3.0870147
C	4.1656662	-2.5279713	-3.6117814
C	4.5878186	-3.8624929	-3.0145762
C	5.3353572	-1.6282914	-3.9728570
H	3.5115047	-2.6918100	-4.4724874
H	3.7133092	-4.4529207	-2.7313684
H	5.1622954	-4.4300848	-3.7537564
H	5.2182875	-3.7054479	-2.1336161
H	4.9856315	-0.6788546	-4.3868694
H	5.9500254	-1.4222342	-3.0912117
H	5.9582620	-2.1230427	-4.7245142
C	-2.1872643	-2.6537455	-3.8996168
C	-1.9577093	-2.2296061	-5.3392845
C	-3.6354077	-2.5646283	-3.4472385
H	-1.7872540	-3.6540041	-3.7145974
H	-0.8966106	-2.2944393	-5.5948987
H	-2.5206860	-2.8891753	-6.0076111
H	-2.2992310	-1.2015344	-5.4969190
H	-3.7280304	-2.8356646	-2.3928769
H	-4.0228392	-1.5528950	-3.5913577
H	-4.2417585	-3.2594934	-4.0381073
B	-2.6783664	0.1684873	0.9449146
C	-2.8434008	1.7907287	0.7295999
C	-4.1703630	-0.4668932	0.6701062
C	-2.0028854	-0.3028539	2.3707391
C	-2.3817519	2.5599476	-0.3410638
C	-3.6556129	2.4859659	1.6305261
C	-4.7189032	-0.3664986	-0.6121490
C	-5.0134061	-1.0553363	1.6132054
C	-1.8331289	-1.6747492	2.5952406
C	-1.5053080	0.5091286	3.3926725
C	-2.6549299	3.9216500	-0.4766638
C	-3.9508001	3.8399062	1.5369148
C	-5.9790012	-0.8426022	-0.9589264
C	-6.2840968	-1.5438360	1.3119944
C	-1.2501047	-2.2130446	3.7361879
C	-0.9291194	0.0125821	4.5601964
C	-3.4362196	4.5705980	0.4703715
C	-6.7704541	-1.4434890	0.0140986
C	-0.8051706	-1.3582387	4.7383141
F	-1.6457255	2.0186086	-1.3401599
F	-4.2102938	1.8194979	2.6736492
F	-4.0203489	0.2448196	-1.6002119
F	-4.6366300	-1.1806856	2.9088055
F	-2.2608409	-2.5654660	1.6705808
F	-1.5144018	1.8601405	3.2900791
F	-2.1794253	4.6178842	-1.5318071
F	-4.7290591	4.4485982	2.4547040
F	-6.4454083	-0.7192354	-2.2198059
F	-7.0474462	-2.1120493	2.2677580
F	-1.1219664	-3.5468325	3.8890688
F	-0.4635372	0.8518803	5.5100415
F	-3.7061109	5.8839249	0.3482906
F	-7.9936269	-1.9107147	-0.2952478
F	-0.2526176	-1.8508910	5.8625118

6 : $Al(C_6F_5)_3$ adduct at PCO oxygen of **4**
138
Energy = -6889.966491191

Ge	2.4247005	-0.5656890	-0.1692425
P	0.2999344	-1.3273545	0.0005823
C	-0.2250532	0.2845338	-0.4825119

O	-1.4762169	0.6283300	-0.5631489
N	3.6248553	-1.4657225	-1.3402269
C	4.9338869	-1.5804564	-1.0614380
C	3.0021678	-2.0609817	-2.5106064
C	5.4918258	-1.2070694	0.1653929
C	5.8558261	-2.1307307	-2.1146756
C	2.4324071	-3.3449268	-2.3688634
C	2.9147023	-1.3296570	-3.7087216
C	4.8293130	-0.8178744	1.3341800
H	6.5647720	-1.3284759	0.2508111
H	6.6422749	-2.7338400	-1.6567824
H	5.3191298	-2.7212778	-2.8577742
H	6.3355541	-1.2897767	-2.6292416
C	1.7762983	-3.8917653	-3.4744225
C	2.5586373	-4.1385039	-1.0780190
C	2.2477441	-1.9260059	-4.7859715
C	3.5230270	0.0515188	-3.8812415
C	5.6483630	-0.6552510	2.5846499
N	3.5056275	-0.5853885	1.3978399
C	1.6824297	-3.1904260	-4.6736003
H	1.3250599	-4.8752024	-3.3939515
C	3.7807842	-5.0765344	-1.1289381
C	1.2882344	-4.9268116	-0.7341292
H	2.7331787	-3.4245032	-0.2645135
H	2.1684948	-1.3834329	-5.7236481
C	2.4608796	1.0853849	-4.2971047
C	4.6613052	0.0279836	-4.9203960
H	3.9498713	0.3601172	-2.9225771
H	5.0756639	-0.9083279	3.4783585
H	6.5488815	-1.2688705	2.5315892
H	5.9543449	0.3932817	2.6736905
C	2.8189595	-0.3558732	2.6596596
H	1.1608214	-3.6296939	-5.5189596
H	4.7076748	-4.5242253	-1.3069221
H	3.8814978	-5.6180641	-0.1821911
H	3.6636306	-5.8096331	-1.9344132
H	0.4225675	-4.2625970	-0.6755953
H	1.0790732	-5.7039900	-1.4754279
H	1.4115911	-5.4231324	0.2331845
H	1.6308317	1.1311924	-3.5886992
H	2.9122591	2.0807072	-4.3601667
H	2.0499119	0.8438819	-5.2830976
H	5.4301976	-0.7068179	-4.6648435
H	4.2721638	-0.2247461	-5.9127332
H	5.1335548	1.0144191	-4.9830786
C	2.1682393	-1.4521567	3.2666180
C	2.7801867	0.9360083	3.2152342
C	1.4997088	-1.2270046	4.4725788
C	2.2023441	-2.8445799	2.6561766
C	2.1013582	1.1027310	4.4279724
C	3.4019146	2.1522333	2.5502320
C	1.4710725	0.0367646	5.0550283
H	0.9860148	-2.0496594	4.9580683
C	0.9273401	-3.6473679	2.9466588
C	3.4522967	-3.6274667	3.1009063
H	2.2674935	-2.7240357	1.5677611
H	2.0509429	2.0923080	4.8719672
C	4.4717859	2.8104591	3.4417027
C	2.3103986	3.1833861	2.2053253
H	3.8826364	1.8325672	1.6207155
H	0.9389433	0.1924394	5.9887984
H	0.0367508	-3.0742217	2.6770141
H	0.9305031	-4.5740134	2.3657445
H	0.8566718	-3.9245816	4.0033970
H	4.3749308	-3.1211214	2.8033672
H	3.4626041	-3.7436927	4.1900060
H	3.4530724	-4.6253260	2.6489256
H	5.2531663	2.1037221	3.7366043
H	4.9439823	3.6426924	2.9083754
H	4.0211057	3.2100875	4.3565015
H	1.5169750	2.7468192	1.5973267
H	1.8504779	3.5740159	3.1194617
H	2.7433424	4.0262857	1.6580786
C	2.0414509	1.2569937	-0.7682603
C	0.7038876	1.4220652	-0.8314191
C	3.1286262	2.1954265	-1.0612816
C	0.0004074	2.6944617	-1.2366231
O	4.3057569	1.8731177	-0.9066995
O	2.7282911	3.3885920	-1.5317665
O	-0.2810687	2.9557419	-2.3868168
O	-0.2758521	3.4219028	-0.1502010
C	3.7965773	4.3182963	-1.9580163
C	4.2740205	5.1156181	-0.7534659
C	3.1951824	5.1696402	-3.0622841
H	4.6090849	3.6945943	-2.3406726
H	4.6941659	4.4528164	0.0069532
H	5.0530515	5.8182447	-1.0672115
H	3.4473401	5.6866640	-0.3195882
H	2.7959317	4.5432099	-3.8640595
H	2.3888132	5.8000762	-2.6775042
H	3.9710550	5.8198743	-3.4789310
C	-1.0465786	4.6745873	-0.3352027
C	-0.0665173	5.8048849	-0.6040424
C	-1.8526452	4.8446165	0.9400336
H	-1.6966962	4.5144349	-1.1975648
H	0.4933999	5.6142597	-1.5219266
H	-0.6154901	6.7450441	-0.7206468
H	0.6348973	5.9116693	0.2298210
H	-2.5226112	3.9954314	1.0886287
H	-1.1892383	4.9315193	1.8067293
H	-2.4550694	5.7559159	0.8698671
Al	-3.0424696	0.0522315	0.1661230
C	-3.1772675	1.0800210	1.8895974
C	-4.4465337	0.6269763	-1.1414311
C	-2.8845843	-1.9366438	0.3601203
C	-2.0695233	1.4204666	2.6609197
C	-4.3894160	1.5862807	2.3484290
C	-4.4777346	1.9449281	-1.5827094
C	-5.4397541	-0.1913250	-1.6690664
C	-2.6669748	-2.6977014	-0.7853191
C	-2.8551637	-2.6425224	1.5565602
C	-2.1345168	2.2290414	3.7913912
C	-4.5151527	2.3964166	3.4738952
C	-5.3995232	2.4399861	-2.4984285

C	-6.3893358	0.2468161	-2.5888699
C	-2.4219825	-4.0650250	-0.7711959
C	-2.6102668	-4.0114355	1.6316611
C	-3.3713512	2.7227344	4.1976105
C	-6.3644111	1.5745974	-3.0077794
C	-2.3874407	-4.7235819	0.4562993
F	-0.8383763	0.9579902	2.3197756
F	-5.5397711	1.2901477	1.6849132
F	-3.5683722	2.8323910	-1.0911018
F	-5.5303011	-1.4915555	-1.2828416
F	-2.6512425	-2.0802739	-1.9971498
F	-3.0433506	-1.9948043	2.7368715
F	-1.0278377	2.5503435	4.4959396
F	-5.7144779	2.8643045	3.8760342
F	-5.3804798	3.7300696	-2.8941343
F	-7.3320853	-0.5862385	-3.0762367
F	-2.1949436	-4.7581791	-1.9064361
F	-2.5729948	-4.6569059	2.8163691
F	-3.4608179	3.5061606	5.2876282
F	-7.2721191	2.0208899	-3.8941773
F	-2.1331308	-6.0436197	0.5057052

7 : polycyclic adduct of **1**, Bf_3 and the alkyne But
138
Energy = -6672.308028289

Ge	-2.4617646	0.4174849	-0.8603031
P	-0.7779511	-1.0546932	0.1122465
C	0.4979062	0.0659772	0.8405295
O	1.6100240	0.5514150	0.4000261
N	-3.6722631	-0.9636579	0.1167330
C	-3.7474676	-1.0050881	1.4101637
C	-4.3453262	-1.8922872	-0.7684234
C	-2.7039924	-0.2503283	2.2199818
C	-4.8025109	-1.7521050	2.1509762
C	-5.2664733	-1.3965017	-1.7121358
C	-3.9487215	-3.2516264	-0.7621410
C	-2.7066728	1.2379397	1.9934140
H	-2.9150299	-0.4268608	3.2763504
H	-4.3304494	-2.4708625	2.8268992
H	-5.3480650	-1.0358453	2.7798951
H	-5.4977341	-2.2517929	1.4758802
C	-5.7627908	-2.2771359	-2.6772497
C	-5.8136569	0.0189540	-1.6725476
C	-4.4844539	-4.0851463	-1.7501112
C	-2.9844245	-3.8678578	0.2366543
C	-2.7378860	2.0857489	3.2170115
N	-2.7052560	1.6910102	0.7764579
C	-5.3707327	-3.6098922	-2.7083008
H	-6.4754309	-1.9090827	-3.4092484
C	-7.3186325	-0.0277515	-1.3388053
C	-5.5649249	0.7704940	-2.9907624
H	-5.3042311	0.5645421	-0.8690092
H	-4.1872648	-5.1294403	-1.7654538
C	-1.7576137	-4.4766141	-0.4624188
C	-3.6867655	-4.9351581	1.0979175
H	-2.6239088	-3.0869550	0.9094967
H	-1.9516350	1.7247654	3.8923762
H	-2.6094164	3.1444834	2.9946166
H	-3.6864490	1.9315417	3.7445531
C	-2.8999648	3.1078063	0.5085114
H	-5.7614156	-4.2776997	-3.4702863
H	-7.7456951	0.9785385	-1.3382517
H	-7.8556897	-0.6221736	-2.0847143
H	-7.4917601	-0.4818233	-0.3573908
H	-5.9395670	1.7973213	-2.9187851
H	-4.4965064	0.8073605	-3.2230899
H	-6.0829102	0.2832140	-3.8231692
H	-1.0671962	-4.8825746	0.2837708
H	-2.0489846	-5.2914342	-1.1328907
H	-1.2258943	-3.7188998	-1.0404273
H	-4.5892847	-4.5450628	1.5764515
H	-3.9757971	-5.7949887	0.4839591
H	-3.0071828	-5.2854854	1.8809176
C	-4.1733032	3.6786541	0.7253582
C	-1.8254901	3.8662556	0.0037667
C	-4.3370677	5.0370731	0.4295450
C	-5.3873359	2.9186999	1.2384256
C	-2.0503801	5.2164326	-0.2814080
C	-0.4268765	3.2967215	-0.1205879
C	-3.2927158	5.8034375	-0.0706071
H	-5.3082813	5.4953682	0.5916787
C	-6.5369200	2.9916076	0.2177494
C	-5.8822935	3.4734292	2.5890571
H	-5.1190288	1.8646056	1.3715404
H	-1.2318175	5.8170582	-0.6628689
C	0.3401007	3.5606235	1.1888822
C	0.3488016	3.8558826	-1.3196611
H	-0.5018328	2.2081730	-0.2436809
H	-3.4453665	6.8551822	-0.2944999
H	-7.3742645	2.3674890	0.5443907
H	-6.9005085	4.0197761	0.1244749
H	-6.2130555	2.6603886	-0.7711673
H	-5.0910769	3.5045414	3.3411771
H	-6.2599491	4.4934148	2.4621404
H	-6.7029445	2.8571557	2.9719051
H	1.3217124	3.0841498	1.1629701
H	0.4668008	4.6400148	1.3324777
H	-0.2011263	3.1641899	2.0512724
H	1.2970999	3.3246042	-1.4169602
H	-0.2103597	3.7364243	-2.2520991
H	0.5771037	4.9182129	-1.1841325
C	-1.2720661	-0.8102323	1.9410146
C	-0.0893333	0.1395744	2.0900005
C	-1.1019437	-2.1059282	2.7233465
C	0.3671385	0.5847443	3.3888432
O	-2.0050108	-2.6651616	3.3297304
O	0.1499564	-2.5594504	2.6221429
O	-0.2499175	0.3187944	4.4280748
O	1.5022246	1.3090121	3.3454575
C	0.4961848	-3.8034649	3.3354947
C	0.9637642	-3.4288951	4.7341072
C	1.5626893	-4.4763079	2.4881754
H	-0.4158178	-4.4051849	3.3821143
H	0.1753416	-2.9013651	5.2770937

H	1.2258755	-4.3356041	5.2895098
H	1.8468030	-2.7851768	4.6757980
H	1.2006002	-4.6542559	1.4717840
H	2.4593617	-3.8534196	2.4346954
H	1.8318722	-5.4377762	2.9369619
C	2.0709032	1.7508834	4.6244446
C	1.5473294	3.1485640	4.9299446
C	3.5801091	1.6875380	4.4504596
H	1.7312608	1.0462439	5.3880855
H	1.9695989	3.5060484	5.8751883
H	1.8323715	3.8454819	4.1352770
H	0.4571142	3.1409018	5.0216168
H	3.9008481	2.3429451	3.6351369
H	4.0712310	2.0096357	5.3748113
H	3.8972467	0.6670360	4.2206957
B	2.4898088	0.0131880	-0.7319493
C	3.1788611	1.3669121	-1.3745418
C	3.7275768	-0.8638125	-0.0892757
C	1.5563552	-0.8572207	-1.7726037
C	3.7396022	2.3133922	-0.5086033
C	3.3092386	1.6811723	-2.7277842
C	3.8598370	-1.2728253	1.2373750
C	4.8046338	-1.2136355	-0.9071048
C	1.5990664	-2.2465485	-1.9289366
C	0.5666820	-0.2320671	-2.5396658
C	4.3166399	3.5076090	-0.9313205
C	3.8803346	2.8627334	-3.1964995
C	4.9758274	-1.9536505	1.7233623
C	5.9357743	-1.8922029	-0.4690479
C	0.7549226	-2.9550899	-2.7829417
C	-0.2986458	-0.8950049	-3.4038552
C	4.3806216	3.7908367	-2.2909909
C	6.0254649	-2.2646610	0.8686626
C	-0.2073531	-2.2782353	-3.5219515
F	3.7560500	2.0945158	0.8276799
F	2.8815560	0.8255061	-3.6885301
F	2.8826753	-1.0508804	2.1460063
F	4.7604687	-0.9141645	-2.2315955
F	2.4585268	-3.0099490	-1.2168637
F	0.3970845	1.1106112	-2.4505758
F	4.8187609	4.3869481	-0.0395183
F	3.9568872	3.1134764	-4.5195637
F	5.0397752	-2.3221576	3.0226388
F	6.9352567	-2.2017487	-1.3203649
F	0.8443994	-4.2957683	-2.8782510
F	-1.2176275	-0.2204352	-4.1226608
F	4.9326320	4.9386331	-2.7243374
F	7.1069053	-2.9242082	1.3236010
F	-1.0410386	-2.9486756	-4.3331164

80 : GePC_3-ring adduct of **1** and PhCCH
90
Energy = -4080.548099770

Ge	-0.1074103	-0.0156429	-0.0732503
P	-0.0050445	0.3638483	-2.2452259
N	0.5506434	-1.6497677	0.7639019
N	-1.8121079	-0.0441368	0.8473005
C	1.0766878	1.8058441	-1.8533620
C	0.0823902	-2.0905219	1.9373806
C	1.6479476	-2.3221394	0.1006253
C	-1.9736267	-0.6525311	2.0337300
C	-2.9427255	0.6080016	0.2198629
O	1.4569870	2.5479029	-2.7678214
C	-1.0343827	-1.5326796	2.5804847
C	0.7609813	-3.2582254	2.6032517
C	1.3449121	-3.1825858	-0.9754842
C	2.9741283	-2.0834146	0.5110131
C	-3.2548977	-0.4213318	2.7905213
C	-3.8658578	-0.1863133	-0.4886416
C	-3.0935151	2.0027423	0.3231329
H	-1.2774611	-1.9453017	3.5517255
H	0.1098161	-3.6936873	3.3624022
H	1.0396991	-4.0204952	1.8720839
H	1.6839756	-2.9240749	3.0876996
C	2.4022779	-3.8525301	-1.5962334
C	-0.0869485	-3.3933089	-1.4419652
C	3.9959014	-2.7820173	-0.1439538
C	3.3515356	-1.0692236	1.5796822
H	-4.0963721	-0.8921616	2.2729123
H	-3.1814871	-0.8404633	3.7949971
H	-3.4801305	0.6471564	2.8502478
C	-4.9804143	0.4432166	-1.0509371
C	-3.6623332	-1.6762522	-0.7122367
C	-4.2265775	2.5855314	-0.2555838
C	-2.0705517	2.8911546	1.0090210
C	3.7174995	-3.6663343	-1.1783517
H	2.1962501	-4.5232379	-2.4236210
C	-0.7832213	-4.5048549	-0.6337673
C	-0.1795458	-3.6736171	-2.9476211
H	-0.6296807	-2.4589242	-1.2555451
H	5.0262025	-2.6158049	0.1580643
C	4.1527152	0.0865519	0.9479237
C	4.1713384	-1.6942649	2.7238644
H	2.4304509	-0.6535793	2.0011723
C	-5.1704202	1.8160885	-0.9269490
H	-5.7036703	-0.1514884	-1.6023233
C	-3.4328761	-1.9490786	-2.2102505
C	-4.8399028	-2.5149717	-0.1855576
H	-2.7626699	-1.9867419	-0.1717884
H	-4.3643179	3.6607020	-0.1816786
C	-2.6625267	3.6128958	2.2326686
C	-1.4865639	3.9010244	0.0055230
H	-1.2494542	2.2573996	1.3600380
H	4.5254579	-4.1970433	-1.6741432
H	-0.8254125	-4.2625449	0.4322682
H	-1.8102120	-4.6450586	-0.9883262
H	-0.2491592	-5.4547359	-0.7476814
H	0.3315982	-2.8934762	-3.5191097
H	0.2568089	-4.6443953	-3.2068181
H	-1.2285151	-3.6940452	-3.2563168
H	3.6230257	0.5150726	0.0960811
H	4.3224519	0.8814255	1.6822741
H	5.1283166	-0.2725070	0.6020511
H	3.6554538	-2.5375124	3.1931989

H	5.1354868	-2.0596151	2.3544139
H	4.3719726	-0.9424112	3.4946587
H	-6.0441375	2.2874655	-1.3680934
H	-2.5696737	-1.3892014	-2.5831349
H	-3.2629673	-3.0172597	-2.3800274
H	-4.3096096	-1.6496642	-2.7945730
H	-5.0128092	-2.3448645	0.8820495
H	-5.7650552	-2.2722695	-0.7193007
H	-4.6371752	-3.5815186	-0.3318642
H	-3.0481072	2.9025887	2.9716534
H	-1.8948665	4.2264429	2.7170723
H	-3.4855632	4.2729404	1.9374519
H	-1.0707446	3.3867890	-0.8649582
H	-2.2606740	4.5931639	-0.3434529
H	-0.6914830	4.4898302	0.4734469
C	1.5098829	2.1457842	-0.4078495
C	1.0106345	1.4152396	0.6087136
H	1.2962121	1.5937241	1.6423309
C	2.4584091	3.2561179	-0.1892744
C	2.3141048	4.0880803	0.9344370
C	3.5505204	3.4736953	-1.0469761
C	3.2299162	5.1023856	1.1992915
H	1.4634955	3.9411370	1.5945094
C	4.4721510	4.4833216	-0.7746728
H	3.6767330	2.8435903	-1.9184409
C	4.3159212	5.3033654	0.3439516
H	3.0937629	5.7405287	2.0683106
H	5.3159855	4.6314431	-1.4430734
H	5.0317597	6.0952785	0.5463573

8a : higher $GePC_3$-ring adduct of **1** and PhCCH
90
Energy = -4080.527513821

Ge	0.0179473	0.0006401	0.5278926
P	-0.2235587	-0.9937986	2.4943435
N	-1.4856825	-0.0680451	-0.7701582
N	1.4324891	-0.6016095	-0.6953966
C	-0.0986354	0.6749633	3.2505115
C	-1.2569649	-0.2286712	-2.0791763
C	-2.8555516	-0.1211940	-0.2577277
C	1.2446323	-0.6066616	-2.0279597
C	2.5577475	-1.3516634	-0.1359971
O	-0.1962042	0.8318635	4.4804878
C	0.0258881	-0.3144873	-2.6476266
C	-2.4034146	-0.3756636	-3.0514755
C	-3.3284214	-1.3596501	0.2374767
C	-3.7044405	1.0148480	-0.2940181
C	2.3908272	-0.9502688	-2.9494149
C	2.3786497	-2.7487618	-0.0090668
C	3.7829586	-0.7450927	0.2267858
H	0.0431614	-0.3434865	-3.7314481
H	-3.3671598	-0.1355674	-2.6076033
H	-2.2253952	0.2690898	-3.9160041
H	-2.4340290	-1.4074686	-3.4167360
C	-4.6508164	-1.4489077	0.6831500
C	-2.4865280	-2.6260131	0.2646218
C	-5.0210029	0.8533924	0.1654982
C	-3.3886844	2.4540421	-0.7158189
H	3.2279319	-1.4025260	-2.4202710
H	2.0524817	-1.6209319	-3.7423872
H	2.7392338	-0.0262173	-3.4244361
C	3.4313177	-3.5278638	0.4788814
C	1.0995367	-3.4620669	-0.4167903
C	4.7996967	-1.5807552	0.7138370
C	4.1530644	0.7358179	0.1946161
C	-5.5009032	-0.3539458	0.6485887
H	-5.0179796	-2.3974291	1.0585700
C	-2.6947519	-3.4577866	-1.0152699
C	-2.7722689	-3.4807364	1.5096287
H	-1.4343753	-2.3310423	0.3125062
H	-5.6785020	1.7187092	0.1365575
C	-2.7932089	3.2299274	0.4731251
C	-2.6092599	2.6806463	-2.0159229
H	-4.3767660	2.8949378	-0.8974464
C	4.6406944	-2.9531929	0.8423685
H	3.2976522	-4.6015091	0.5686416
C	0.6055172	-4.4027014	0.6927411
C	1.2960064	-4.2423062	-1.7311490
H	0.3229514	-2.7118814	-0.5912651
H	5.7456966	-1.1218313	0.9902548
C	3.7750907	1.5127913	-1.0698999
C	3.6786548	1.4283005	1.4846154
H	5.2498426	0.7415788	0.2302932
H	-6.5271388	-0.4399217	0.9942377
H	-2.3715727	-2.9160736	-1.9077949
H	-2.1226873	-4.3899322	-0.9594959
H	-3.7528528	-3.7144609	-1.1370679
H	-2.0560998	-4.3038582	1.5665408
H	-2.6758947	-2.8797803	2.4175901
H	-3.7736966	-3.9224707	1.4758829
H	-3.4850173	3.2106053	1.3214147
H	-2.6105714	4.2731441	0.1923337
H	-1.8518129	2.7939189	0.8031635
H	-3.1379916	2.2476408	-2.8697457
H	-1.5993223	2.2762727	-1.9842458
H	-2.5200348	3.7583663	-2.1894637
H	5.4535892	-3.5679870	1.2180985
H	0.4201607	-3.8496869	1.6179691
H	-0.3221963	-4.8910780	0.3803699
H	1.3350019	-5.1918100	0.8997427
H	1.6154695	-3.5877690	-2.5464229
H	2.0587036	-5.0186133	-1.6038027
H	0.3595740	-4.7275763	-2.0267329
H	4.2303359	1.0579669	-1.9551465
H	2.6984262	1.5706746	-1.2178368
H	4.1473572	2.5396616	-0.9884612
H	2.5953491	1.3753366	1.5912559
H	3.9713983	2.4842367	1.4789744
H	4.1222540	0.9449543	2.3607279
C	0.3160018	1.8816506	1.0791829
C	0.1551610	1.9001293	2.4194745
H	0.2305935	2.8112234	3.0148966
C	0.6589747	3.0296715	0.2402212
C	0.9975180	4.2816409	0.7937027

C	0.6738712	2.9113168	-1.1578942
C	1.3180333	5.3603924	-0.0210186
H	1.0157988	4.4052097	1.8719491
C	0.9901483	3.9909946	-1.9782353
H	0.4381466	1.9521388	-1.6126048
C	1.3126789	5.2237029	-1.4139179
H	1.5774065	6.3145298	0.4294888
H	0.9877959	3.8664216	-3.0575892
H	1.5639475	6.0693616	-2.0476135

8 : adduct of PhCCH and Ge/B FLP of **1** and Bf_3
124
Energy = -6290.082770733

Ge	-1.8859726	0.3082741	-0.1444818
P	-1.9372669	0.9455323	2.0953274
C	-1.9526300	-0.6624672	2.5692612
O	-1.9752375	-1.7241830	3.0419427
N	-2.3742657	1.9462058	-1.0938926
C	-3.1968515	1.8892669	-2.1517589
C	-1.6897192	3.1761106	-0.7191014
C	-3.8739984	0.7264634	-2.5508474
C	-3.4418306	3.1197968	-2.9839588
C	-2.1731345	3.9263241	0.3770868
C	-0.5512314	3.5799044	-1.4469364
C	-4.0737219	-0.4412102	-1.8179668
H	-4.4295541	0.8058092	-3.4775574
H	-4.5155944	3.2473250	-3.1442521
H	-3.0287608	4.0180582	-2.5278689
H	-2.9800608	2.9804865	-3.9673404
C	-1.4292292	5.0370322	0.7865016
C	-3.5124548	3.6327019	1.0367221
C	0.1485583	4.7045975	-0.9944973
C	-0.0777162	2.9085848	-2.7245998
C	-5.0323882	-1.4604619	-2.3695755
N	-3.4513128	-0.7012462	-0.6471345
C	-0.2670600	5.4148788	0.1225543
H	-1.7698177	5.6199721	1.6348838
C	-4.6588546	4.2932780	0.2449907
C	-3.5715400	4.0669565	2.5073741
H	-3.6796169	2.5505259	1.0008426
H	1.0361785	5.0219464	-1.5335354
C	1.3610139	2.3989054	-2.5896452
C	-0.1667542	3.8811798	-3.9190693
H	-0.7216025	2.0477300	-2.9301417
H	-5.5780306	-1.9796763	-1.5807482
H	-5.7349590	-0.9960492	-3.0626201
H	-4.4482286	-2.2086484	-2.9193013
C	-4.0727689	-1.6039506	0.3127689
H	0.3047020	6.2700178	0.4689470
H	-4.7124650	3.9159282	-0.7790935
H	-5.6188542	4.0909352	0.7321110
H	-4.5175196	5.3785040	0.2017266
H	-2.7303973	3.6649628	3.0803107
H	-3.5617572	5.1577029	2.6025339
H	-4.4984203	3.7074496	2.9623763
H	1.4545390	1.7055140	-1.7556145
H	1.6671942	1.8824621	-3.5034246
H	2.0510741	3.2328022	-2.4248065
H	-1.1609649	4.3225828	-4.0280681
H	0.5494713	4.7000151	-3.7941124
H	0.0846386	3.3546720	-4.8459040
C	-5.0639570	-1.0532432	1.1592424
C	-3.7154059	-2.9623626	0.3778009
C	-5.6770759	-1.8976076	2.0878729
C	-5.5186511	0.3926710	1.0420581
C	-4.3688344	-3.7642062	1.3224988
C	-2.6890365	-3.6072046	-0.5343739
C	-5.3343224	-3.2437326	2.1728908
H	-6.4389479	-1.4974103	2.7489515
C	-5.7741963	1.0471911	2.4062585
C	-6.7792904	0.5030942	0.1610439
H	-4.7193258	0.9536565	0.5466891
H	-4.1105453	-4.8168786	1.3855767
C	-3.3432275	-4.6440384	-1.4682004
C	-1.5788048	-4.2831142	0.2879115
H	-2.2378605	-2.8285336	-1.1560159
H	-5.8232708	-3.8849059	2.9006695
H	-4.8858374	0.9933091	3.0418621
H	-6.0386436	2.0996515	2.2658633
H	-6.6081829	0.5713656	2.9315705
H	-6.6095763	0.1086206	-0.8439387
H	-7.6069201	-0.0574061	0.6093990
H	-7.0833402	1.5512853	0.0683145
H	-4.1623753	-4.2134573	-2.0508855
H	-2.5945585	-5.0363272	-2.1639303
H	-3.7469599	-5.4832240	-0.8907799
H	-1.0605316	-3.5674311	0.9274636
H	-1.9950455	-5.0694482	0.9263745
H	-0.8481186	-4.7469823	-0.3800423
C	-0.1753795	-0.4985905	-0.6682617
C	0.8290556	-0.2045357	0.1916755
C	-0.2120019	-1.3878956	-1.8429313
C	-0.8965044	-1.0132661	-3.0096265
C	0.3587357	-2.6696379	-1.7919769
C	-0.9939106	-1.8755752	-4.0980229
H	-1.3530967	-0.0283863	-3.0681591
C	0.2644721	-3.5334729	-2.8808029
H	0.8565427	-2.9860506	-0.8838500
C	-0.4085512	-3.1419760	-4.0384994
H	-1.5207387	-1.5580545	-4.9936252
H	0.7142978	-4.5206150	-2.8207343
H	-0.4821881	-3.8192920	-4.8841849
H	0.4910697	0.4236576	1.0183422
B	2.4494518	-0.4605336	0.3963325
C	2.5977151	-0.8964044	1.9882685
C	3.1032440	-1.5864690	-0.6105613
C	3.1098711	1.0640260	0.2131962
C	1.7059090	-1.8006977	2.5656158
C	3.5649328	-0.4147778	2.8720738
C	3.0286649	-1.4338146	-1.9979710
C	3.7173162	-2.7788880	-0.2171298
C	4.1935243	1.3918572	-0.6079948
C	2.6036458	2.1633889	0.9201239
C	1.7134119	-2.1727645	3.9048644

C	3.6153761	-0.7523592	4.2236348
C	3.4876979	-2.3574460	-2.9261797
C	4.1817103	-3.7470224	-1.1097310
C	4.6811332	2.6863203	-0.7827342
C	3.0667840	3.4682235	0.7924209
C	2.6772107	-1.6323893	4.7496614
C	4.0642640	-3.5399709	-2.4774447
C	4.1078091	3.7409347	-0.0857501
F	0.7831021	-2.4223045	1.7819669
F	4.5541502	0.4106627	2.4449090
F	2.4675397	-0.3146727	-2.5134194
F	3.9119045	-3.0837920	1.0912397
F	4.8840195	0.4391591	-1.2813074
F	1.6206201	2.0018459	1.8499298
F	0.8182935	-3.0595129	4.3881192
F	4.5712664	-0.2404131	5.0267269
F	3.3531226	-2.1364460	-4.2502764
F	4.7537777	-4.8814455	-0.6536579
F	5.7159495	2.9226274	-1.6157784
F	2.5309514	4.4678742	1.5240840
F	2.7115127	-1.9711584	6.0520232
F	4.4993693	-4.4662320	-3.3523887
F	4.5620321	4.9990947	-0.2417253

9 : GePC$_3$-ring adduct of **1**Bf$_3$o and alkene Mal
128
Energy = -6516.162527368

Ge	2.5947750	0.1940100	-0.4684857
P	0.5186010	-0.1935064	-1.2681056
N	3.3497034	1.9358650	-0.8957326
N	4.0709750	-0.8034204	-1.1536822
C	-0.0802712	-0.0192008	0.3879384
C	4.2162119	2.0927196	-1.9004299
C	2.6842208	3.0306209	-0.2178889
C	4.7986721	-0.3293483	-2.1908743
C	4.3990885	-2.0847853	-0.5550761
O	-1.2772478	-0.2525470	0.8036173
C	4.8002355	1.0086664	-2.5777209
C	4.6269235	3.4739291	-2.3308317
C	1.5794327	3.6646768	-0.8288639
C	3.0936265	3.3375971	1.0997692
C	5.6499170	-1.2959928	-2.9694415
C	3.7420319	-3.2552787	-0.9730594
C	5.3939363	-2.0897575	0.4480814
H	5.4301774	1.2623891	-3.4216347
H	4.2715662	3.6583942	-3.3499139
H	4.2238771	4.2420265	-1.6715135
H	5.7183537	3.5428428	-2.3486494
C	0.8381679	4.5584822	-0.0457956
C	1.2198029	3.5040581	-2.3005595
C	2.3175589	4.2412631	1.8303679
C	4.3808109	2.7686749	1.6809706
H	6.1249374	-2.0345982	-2.3218488
H	5.0071710	-1.8415195	-3.6701624
H	6.4098976	-0.7628177	-3.5416607
C	4.1144156	-4.4585257	-0.3600566
C	2.6852017	-3.2728622	-2.0653183

C	5.7339602	-3.3190128	1.0168361
C	6.0911422	-0.8096889	0.8910559
C	1.1833746	4.8273255	1.2723717
H	-0.0326132	5.0398004	-0.4792237
C	1.5221161	4.8213008	-3.0503941
C	-0.2506983	3.1292721	-2.5402312
H	1.8402449	2.7075790	-2.7258399
H	2.5996473	4.4859895	2.8488867
C	4.3525363	2.6134255	3.2081367
C	5.5860911	3.6356397	1.2639064
H	4.5391210	1.7779278	1.2398591
C	5.1018379	-4.4940868	0.6176588
H	3.6228725	-5.3790620	-0.6617371
C	1.3367775	-3.7851544	-1.5332945
C	3.1363842	-4.1303203	-3.2635308
H	2.5363646	-2.2453749	-2.4165818
H	6.4895834	-3.3559279	1.7944074
C	7.3683415	-0.5437581	0.0691884
C	6.4297506	-0.7928357	2.3891414
H	5.3999421	0.0195489	0.6970890
H	0.5740858	5.5033662	1.8645039
H	2.5397136	5.1796924	-2.8764041
H	1.3813744	4.6795458	-4.1272561
H	0.8317284	5.6046406	-2.7197216
H	-0.4952027	2.1741488	-2.0744208
H	-0.9251756	3.8923005	-2.1402610
H	-0.4360312	3.0487835	-3.6166493
H	3.4784994	2.0501903	3.5512999
H	5.2489316	2.0831513	3.5421142
H	4.3410067	3.5882326	3.7065658
H	5.6811061	3.6938653	0.1767327
H	5.4753026	4.6542648	1.6506812
H	6.5125225	3.2119578	1.6665087
H	5.3753607	-5.4390711	1.0784691
H	0.9700242	-3.1520715	-0.7236064
H	0.5911763	-3.7778931	-2.3329655
H	1.4321202	-4.8127628	-1.1645088
H	4.0990844	-3.8003791	-3.6659476
H	3.2403307	-5.1802654	-2.9692976
H	2.3896781	-4.0767046	-4.0626986
H	7.1591896	-0.4308730	-0.9969452
H	7.8506129	0.3765212	0.4169834
H	8.0773292	-1.3697679	0.1924896
H	5.5456597	-1.0035511	2.9935681
H	7.2080050	-1.5252256	2.6296086
H	6.8213642	0.1938041	2.6599697
H	0.8060116	1.6488039	1.3211864
C	0.8028261	0.5693025	1.5211254
C	0.1743816	0.4035862	2.9036553
C	2.2545682	0.0635977	1.4889081
O	0.5174332	-0.4107412	3.7365367
O	-0.7865040	1.3242169	3.0944407
H	2.9114784	0.7246343	2.0476069
C	2.4193235	-1.3271280	2.0598004
C	-1.5331236	1.1901817	4.3331174
O	3.2961287	-1.6488379	2.8365838
O	1.4808793	-2.1680123	1.5818451

H	-2.2785368	1.9830651	4.2986531
H	-2.0046264	0.2064579	4.3676249
H	-0.8644735	1.3145813	5.1873715
C	1.4489964	-3.4768082	2.2062086
H	1.2519481	-3.3576238	3.2731102
H	2.4009195	-3.9856194	2.0463337
H	0.6321816	-4.0028222	1.7153417
B	-2.6973446	-0.3393557	0.1983286
C	-3.4203422	1.0017982	0.8369715
C	-2.6138474	-0.3976703	-1.4378504
C	-3.3627221	-1.7293416	0.7670384
C	-2.7524678	2.2306185	0.8318066
C	-4.6487047	1.0338740	1.5016195
C	-2.9720387	0.6202838	-2.3233078
C	-2.1337360	-1.5577392	-2.0597244
C	-2.9001206	-2.4654707	1.8586476
C	-4.4733769	-2.2887317	0.1287286
C	-3.2265042	3.3892725	1.4321267
C	-5.1692827	2.1738134	2.1171583
C	-2.8285187	0.5247421	-3.7061671
C	-1.9868785	-1.7024321	-3.4332556
C	-3.4586583	-3.6786463	2.2621712
C	-5.0655407	-3.4901964	0.4979465
C	-4.4528255	3.3627340	2.0877773
C	-2.3257755	-0.6427420	-4.2673407
C	-4.5474505	-4.2004050	1.5763686
F	-1.5654714	2.3514420	0.1903606
F	-5.4351819	-0.0655822	1.5907412
F	-3.4962021	1.7868329	-1.8784549
F	-1.7439746	-2.6165406	-1.3086354
F	-1.8599445	-2.0357460	2.6144797
F	-5.0569095	-1.6356889	-0.9093650
F	-2.5171306	4.5378246	1.3814709
F	-6.3633087	2.1303761	2.7423799
F	-3.1717076	1.5545932	-4.5060800
F	-1.4875170	-2.8377730	-3.9633491
F	-2.9501228	-4.3487110	3.3180772
F	-6.1365658	-3.9690432	-0.1673004
F	-4.9377402	4.4707788	2.6763584
F	-2.1744097	-0.7483181	-5.5989350
F	-5.1002802	-5.3671886	1.9549765

Alf3d : F..Al bonded dimer of $Al(C_6F_5)_3$

68

Energy = -4854.293992656

Al	1.3294053	1.6446047	-0.2466210
C	2.6983879	2.6441211	-1.2396951
C	-0.5550071	2.1564988	-0.6408395
C	1.6948079	1.0230063	1.5828488
C	2.5890166	2.8037605	-2.6188359
C	3.8108056	3.2371848	-0.6474233
C	-1.5901054	1.3971778	-1.1320736
C	-0.8856641	3.4963396	-0.4437895
C	0.9282545	1.4143236	2.6785791
C	2.8227599	0.2634273	1.8816879
C	3.5157940	3.4979134	-3.3872376
F	1.5247008	2.2524503	-3.2637877
C	4.7685285	3.9433858	-1.3687443
F	3.9894154	3.1420719	0.6932350
C	-2.8598424	1.8447927	-1.4579275
F	-1.3703576	0.0069702	-1.3755751
C	-2.1384020	4.0315467	-0.7277964
F	0.0632977	4.3288342	0.0514462
C	1.2479957	1.0804151	3.9903027
F	-0.1833804	2.1697601	2.4885403
C	3.1771007	-0.1182779	3.1693997
F	3.6396999	-0.1262428	0.8665619
C	4.6152552	4.0714290	-2.7487074
F	3.3732311	3.6255602	-4.7172320
F	5.8292724	4.5022198	-0.7628143
C	-3.1317958	3.1965181	-1.2433395
F	-3.7993749	1.0304877	-1.9490361
F	-2.4058828	5.3260362	-0.5200183
C	2.3769233	0.2991210	4.2331471
F	0.4847270	1.4879335	5.0179960
F	4.2670144	-0.8656878	3.4049777
F	5.5243989	4.7473583	-3.4622009
F	-4.3408370	3.6861717	-1.5268768
F	2.6935044	-0.0477395	5.4845916
Al	-1.3295198	-1.6440048	-0.2465881
C	0.5549360	-2.1557017	-0.6408766
C	-2.6981919	-2.6439671	-1.2396590
C	-1.6951577	-1.0228536	1.5829493
C	1.5898425	-1.3963525	-1.1324609
C	0.8858091	-3.4954556	-0.4435849
C	-3.8103147	-3.2375566	-0.6473579
C	-2.5887682	-2.8035726	-2.6187958
C	-2.8245199	-0.2655243	1.8821842
C	-0.9273917	-1.4125912	2.6783859
C	2.8596159	-1.8438443	-1.4583498
F	1.3696881	-0.0062130	-1.3759072
C	2.1385977	-4.0305357	-0.7276171
F	-0.0629787	-4.3279955	0.0518930
C	-4.7676804	-3.9442769	-1.3686484
F	-3.9889927	-3.1424582	0.6932866
C	-3.5152071	-3.4982064	-3.3871759
F	-1.5247271	-2.2517625	-3.2637732
C	-3.1789970	0.1157418	3.1699844
F	-3.6429752	0.1218632	0.8674129
C	-1.2472121	-1.0791388	3.9902014
F	0.1856225	-2.1659240	2.4879368
C	3.1318141	-3.1954742	-1.2434618
F	3.7989773	-1.0294883	-1.9496994
F	2.4063029	-5.3249374	-0.5195621
C	-4.6143672	-4.0722700	-2.7486160
F	-5.8281213	-4.5036493	-0.7626879
F	-3.3725904	-3.6258177	-4.7171656
C	-2.3775728	-0.3000362	4.2334281
F	-4.2701944	0.8610943	3.4059471
F	-0.4827121	-1.4850434	5.0176270
F	4.3409148	-3.6849916	-1.5269591
F	-5.5231669	-4.7487002	-3.4620729
F	-2.6944085	0.0461714	5.4849982

AlF_3OCP^- : $Al(C_6F_5)_3$ adduct at oxygen of PCO^-
37
Energy = -2882.074265420

P	-0.6029961	2.5709417	3.9911213
C	-0.3247961	1.3006952	3.0990749
O	-0.0995821	0.2785240	2.4074764
Al	-0.0144592	-0.0615774	0.6246426
C	0.2487008	1.7069690	-0.2868844
C	1.5865957	-1.2187050	0.2781038
C	-1.7531659	-0.9660868	0.1888715
C	1.2991662	2.5198754	0.1264107
C	-0.5269308	2.2223145	-1.3184858
C	2.4295375	-1.7988485	1.2187516
C	1.9289846	-1.4556978	-1.0484262
C	-1.9095573	-2.2496602	-0.3211602
C	-2.9381803	-0.2705938	0.4113637
C	1.5720096	3.7708087	-0.4175799
F	2.1304138	2.0839656	1.1113378
C	-0.2989563	3.4651731	-1.9032110
F	-1.5661490	1.5019104	-1.8196091
C	3.5460008	-2.5603214	0.8776826
F	2.1853011	-1.6461506	2.5451776
C	3.0290132	-2.2055460	-1.4479857
F	1.1426925	-0.9375076	-2.0354578
C	-3.1495116	-2.8175985	-0.6055783
F	-0.8216351	-3.0278840	-0.5701290
C	-4.2022023	-0.7856106	0.1453101
F	-2.8804456	0.9913581	0.9133669
C	0.7592828	4.2443388	-1.4439183
F	2.6045213	4.5248092	0.0174568
F	-1.0779045	3.9273166	-2.9065068
C	3.8454402	-2.7623061	-0.4665648
F	4.3407862	-3.1078442	1.8234311
F	3.3184988	-2.4064903	-2.7521876
C	-4.3030460	-2.0759225	-0.3693564
F	-3.2544058	-4.0716475	-1.0989113
F	-5.3239776	-0.0685519	0.3708368
F	0.9999482	5.4496911	-1.9958769
F	4.9192435	-3.4965325	-0.8177438
F	-5.5130404	-2.6053533	-0.6351224

AlF_3PCO^- : $Al(C_6F_5)_3$ adduct at P of PCO^-
37
Energy = -2882.080407617

P	0.6701807	0.8314480	2.8407235
C	-0.3730653	-0.3355247	3.3702201
O	-1.1085414	-1.1462555	3.8057127
Al	0.2089164	0.1639241	0.5666936
C	0.2151109	1.8993029	-0.4721268
C	1.7301376	-1.0307383	-0.0205953
C	-1.5790841	-0.7568260	0.4124909
C	1.2418065	2.8159623	-0.2691693
C	-0.7483479	2.2965642	-1.3927084
C	1.9884397	-2.1997921	0.6906676
C	2.6027702	-0.7833430	-1.0748790
C	-1.8018051	-2.0305260	-0.0991986
C	-2.7199505	-0.1012258	0.8673241
C	1.3245138	4.0492659	-0.9062117
F	2.2518657	2.5072599	0.5923848
C	-0.7188330	3.5191247	-2.0605934
F	-1.7873835	1.4740288	-1.7015306
C	3.0347176	-3.0699977	0.4035819
F	1.1773850	-2.5451539	1.7257094
C	3.6680798	-1.6179230	-1.4075214
F	2.4408180	0.3166495	-1.8605597
C	-3.0577238	-2.6316597	-0.1510412
F	-0.7706767	-2.7624303	-0.6010076
C	-3.9966685	-0.6511380	0.8408364
F	-2.6119004	1.1607707	1.3639869
C	0.3266450	4.4026596	-1.8109195
F	2.3462810	4.9027430	-0.6728376
F	-1.6794787	3.8606650	-2.9487211
C	3.8840749	-2.7705284	-0.6587603
F	3.2408223	-4.1946323	1.1244800
F	4.4899120	-1.3340307	-2.4437139
C	-4.1628461	-1.9342999	0.3264579
F	-3.2243582	-3.8726983	-0.6615407
F	-5.0719769	0.0297999	1.2953619
F	0.3766696	5.5907194	-2.4457765
F	4.9066627	-3.5949632	-0.9625882
F	-5.3879741	-2.4949460	0.2850890

AlF_3.Tol : $Al(C_6F_5)_3$ adduct at para-site of toluene
49
Energy = -2698.889013875

Al	0.1638869	0.0895513	1.3715241
C	0.5918800	1.8993269	0.6711531
C	1.5596122	-1.3126690	1.1127818
C	-1.6360512	-0.6501979	0.9242997
C	1.8977764	2.3659226	0.5492177
C	-0.3988353	2.8144769	0.3275510
C	1.8692720	-2.3215081	2.0179616
C	2.2610621	-1.3649115	-0.0903738
C	-1.9792300	-0.6165071	-0.4253213
C	-2.5786670	-1.2452951	1.7542858
C	2.2194444	3.6475264	0.1135390
F	2.9378776	1.5644174	0.8971759
C	-0.1350123	4.1072628	-0.1139660
F	-1.7069810	2.4678952	0.4503745
C	2.8208136	-3.3085114	1.7824539
F	1.2222873	-2.3750344	3.2185019
C	3.2209345	-2.3285290	-0.3825117
F	2.0042448	-0.4426517	-1.0532157
C	-3.1718716	-1.1116799	-0.9390146
F	-1.0987933	-0.0694185	-1.3080467
C	-3.7903496	-1.7577681	1.2989864
F	-2.3440541	-1.3535295	3.0932269
C	1.1900367	4.5235528	-0.2231861
F	3.4991398	4.0589814	0.0291291
F	-1.1297071	4.9605909	-0.4226777
C	3.5032125	-3.3073306	0.5679925
F	3.0884647	-4.2572062	2.6990400
F	3.8729873	-2.3341374	-1.5590086
C	-4.0872698	-1.6879485	-0.0601523

F	-3.4537577	-1.0486724	-2.2525822
F	-4.6727584	-2.3197692	2.1460050
F	1.4733933	5.7677446	-0.6375672
F	4.4227480	-4.2493979	0.3119526
F	-5.2471582	-2.1782769	-0.5215549
C	0.0619237	0.4518369	3.6794574
C	1.4148491	0.8398909	3.7896460
C	1.7528312	2.1877290	3.7933535
C	0.7642119	3.1801762	3.7012643
C	-0.5808939	2.7897213	3.6162459
C	-0.9370605	1.4463907	3.6149011
H	-0.2172577	-0.5891307	3.8214804
H	2.1863676	0.0813805	3.8692136
H	2.7974008	2.4780506	3.8576781
H	-1.3533398	3.5497587	3.5423528
H	-1.9806217	1.1559801	3.5610036
C	1.1429403	4.6359857	3.6952237
H	1.3194788	4.9850919	4.7202191
H	2.0654749	4.8000926	3.1312380
H	0.3491065	5.2506320	3.2636693

Alf3 : strong Lewis acid $Al(C_6F_5)_3$
34
Energy = -2427.124256787

Al	0.1168062	-0.1763259	-0.4341892
C	0.3020790	1.7115787	-0.9138418
C	1.6976271	-1.3198903	-0.3044544
C	-1.6601155	-0.9121380	-0.0769698
C	1.3796317	2.4833228	-0.4827669
C	-0.6409285	2.3648085	-1.7054383
C	1.7772503	-2.3658588	0.6140655
C	2.8088677	-1.1430129	-1.1272746
C	-2.0225417	-2.1976600	-0.4740745
C	-2.6375755	-0.1789353	0.5928719
C	1.5307481	3.8263532	-0.8090331
F	2.3264665	1.9175449	0.3060940
C	-0.5377782	3.7050586	-2.0605482
F	-1.7088701	1.6713582	-2.1728574
C	2.8849332	-3.1982952	0.7231190
F	0.7401900	-2.5880949	1.4593322
C	3.9389576	-1.9496090	-1.0613352
F	2.7988537	-0.1508038	-2.0507505
C	-3.2783532	-2.7419481	-0.2297893
F	-1.1241639	-2.9617923	-1.1437172
C	-3.9068047	-0.6764381	0.8656848
F	-2.3478515	1.0758205	1.0198126
C	0.5601866	4.4362132	-1.6051597
F	2.5793699	4.5412762	-0.3726444
F	-1.4628362	4.3010828	-2.8288646
C	3.9710919	-2.9838877	-0.1256949
F	2.9303328	-4.1913523	1.6255946
F	4.9878965	-1.7562065	-1.8768518
C	-4.2232338	-1.9697319	0.4474799
F	-3.5973199	-3.9815172	-0.6334574
F	-4.8216307	0.0542771	1.5216595
F	0.6825476	5.7273255	-1.9325803
F	5.0481799	-3.7726394	-0.0412769
F	-5.4376760	-2.4718150	0.6971558

A : Ge-to-C adduct of **1** and the alkyne But
104
Energy = -4462.688210531

Ge	-0.2002298	0.0048778	0.0955654
P	-0.3921564	0.7520685	-2.1290118
C	1.0328177	1.6110453	-2.0241197
O	2.0015449	2.2623339	-2.0737225
N	-2.0213453	-0.6029723	0.4228924
C	-2.6005390	-0.3785753	1.6147252
C	-2.6883007	-1.3562458	-0.6173785
C	-2.1181509	0.5459156	2.5460039
C	-3.8144005	-1.1833777	2.0008963
C	-3.5736885	-0.6667659	-1.4776209
C	-2.4208806	-2.7302905	-0.7729171
C	-1.1016095	1.4913295	2.3769700
H	-2.6492470	0.5867425	3.4893834
H	-4.4617829	-0.6104428	2.6671684
H	-4.3807114	-1.5214029	1.1327220
H	-3.4721079	-2.0723583	2.5447890
C	-4.2150816	-1.4011028	-2.4793095
C	-3.8501522	0.8221988	-1.3239769
C	-3.0860707	-3.4155665	-1.7964320
C	-1.4648520	-3.4886629	0.1299573
C	-0.8797019	2.4819873	3.4903785
N	-0.3056830	1.5381731	1.2962879
C	-3.9795885	-2.7644229	-2.6379788
H	-4.9038747	-0.8975123	-3.1502341
C	-5.0985557	1.0855639	-0.4598146
C	-3.9919302	1.5363576	-2.6763440
H	-2.9898659	1.2598600	-0.8055351
H	-2.8941733	-4.4761869	-1.9312265
C	-0.3595377	-4.1874072	-0.6821268
C	-2.2144486	-4.5164522	0.9996335
H	-0.9975820	-2.7661612	0.8038696
H	-0.4572285	3.4204275	3.1300337
H	-1.8102621	2.6748892	4.0269453
H	-0.1648493	2.0437033	4.1976879
C	0.5434475	2.6776601	1.0286540
H	-4.4859223	-3.3146173	-3.4261164
H	-4.9889489	0.6800499	0.5485246
H	-5.2743618	2.1636299	-0.3737823
H	-5.9835903	0.6288127	-0.9168160
H	-3.1194383	1.3604256	-3.3120073
H	-4.8863812	1.2055552	-3.2146085
H	-4.0878569	2.6144738	-2.5150809
H	0.1468614	-3.4977863	-1.3614958
H	0.3916117	-4.6054959	-0.0035668
H	-0.7782850	-5.0109944	-1.2725249
H	-2.9981882	-4.0467708	1.6014425
H	-2.6838814	-5.2833062	0.3733172
H	-1.5126266	-5.0157136	1.6770022
C	-0.0121714	3.7721859	0.3281232
C	1.8934344	2.6598741	1.4272711
C	0.8109124	4.8731775	0.0781484
C	-1.4585962	3.7720214	-0.1419683

C	2.6754728	3.7857718	1.1445392
C	2.5234998	1.4807524	2.1467582
C	2.1421365	4.8866327	0.4862635
H	0.4081350	5.7286250	-0.4547980
C	-1.6229818	4.4155704	-1.5262820
C	-2.3922815	4.4566446	0.8744470
H	-1.7655089	2.7246039	-0.2278433
H	3.7191803	3.7920382	1.4459536
C	2.9528163	1.8641360	3.5758495
C	3.7273091	0.9288753	1.3606709
H	1.7699201	0.6926417	2.2259754
H	2.7657919	5.7510271	0.2761489
H	-0.9692072	3.9382588	-2.2614713
H	-2.6580042	4.3046312	-1.8640332
H	-1.3995144	5.4873302	-1.5032706
H	-2.3737548	3.9551898	1.8451164
H	-2.0967891	5.5011534	1.0250915
H	-3.4241509	4.4421687	0.5060038
H	2.1081808	2.2299241	4.1677267
H	3.3817434	0.9955365	4.0865964
H	3.7140198	2.6518638	3.5526639
H	3.4671295	0.7103214	0.3229034
H	4.5533906	1.6492842	1.3646229
H	4.0815263	0.0009517	1.8214110
C	1.2958490	-1.3220607	0.3418822
C	2.1561384	-1.7138357	-0.5718105
C	1.1674008	-1.7838224	1.7296726
C	2.3885570	-1.5658469	-1.9524041
O	0.2839906	-1.3336048	2.4716142
O	2.0474194	-2.7191828	2.1100872
O	1.8332561	-2.2545560	-2.8209133
O	3.4066233	-0.6802867	-2.2337508
C	1.9164350	-3.2228507	3.4861061
C	2.6356823	-2.2729575	4.4344911
C	2.5027492	-4.6243538	3.4601671
H	0.8476313	-3.2453971	3.7176623
H	2.1746215	-1.2826995	4.4052775
H	2.5733874	-2.6542176	5.4594774
H	3.6918069	-2.1869782	4.1587071
H	1.9654701	-5.2546583	2.7456351
H	3.5586367	-4.5943768	3.1732622
H	2.4231941	-5.0761758	4.4543332
C	3.9013111	-0.6943780	-3.6087993
C	4.8168437	-1.8979540	-3.8080048
C	4.6150880	0.6341730	-3.8075264
H	3.0339892	-0.7734563	-4.2718001
H	4.2668137	-2.8264097	-3.6374250
H	5.2083057	-1.9061463	-4.8318703
H	5.6616904	-1.8486849	-3.1121980
H	3.9359002	1.4714200	-3.6306577
H	5.4607315	0.7205186	-3.1164500
H	4.9971660	0.6987770	-4.8321769

$Bf_3OCPBf_3^-$: $B(C_6F_5)_3$ adduct at O and P of PCO^-
71
Energy = -4873.969845562

P	1.0563672	0.8847998	1.5565319
C	-0.1976718	-0.0210417	1.1101697
O	-1.1862410	-0.7055959	0.9219451
B	-2.7085232	-0.3350540	0.2708281
C	-2.9159212	-1.5028322	-0.8330526
C	-3.6535553	-0.5138984	1.5781522
C	-2.5513063	1.1741334	-0.2817663
C	-1.9143588	-1.8226016	-1.7545207
C	-4.0868991	-2.2521693	-0.9667783
C	-3.5254821	-1.6258501	2.4169533
C	-4.6751990	0.3685352	1.9333246
C	-2.1892242	2.2056946	0.5927508
C	-2.7053573	1.5712006	-1.6117811
C	-2.0348596	-2.8172467	-2.7181412
C	-4.2508359	-3.2601577	-1.9152411
C	-4.3087293	-1.8327716	3.5482762
C	-5.4850339	0.1975050	3.0528445
C	-1.9559786	3.5156888	0.1952722
C	-2.4935804	2.8753106	-2.0527402
C	-3.2153140	-3.5502863	-2.7946193
C	-5.2965806	-0.9091160	3.8723664
C	-2.1067808	3.8537929	-1.1455201
F	-0.7668703	-1.1103178	-1.7656925
F	-5.1646295	-2.0148532	-0.1787433
F	-2.6220754	-2.5929628	2.1333652
F	-4.9563904	1.4505137	1.1667513
F	-2.0860416	1.9549041	1.9219648
F	-3.0964152	0.6916671	-2.5649293
F	-1.0394935	-3.0634022	-3.5911737
F	-5.4063367	-3.9513619	-1.9913812
F	-4.1338614	-2.9230047	4.3217611
F	-6.4528971	1.0895653	3.3466782
F	-1.6001771	4.4591634	1.0888950
F	-2.6530369	3.1954032	-3.3515110
F	-3.3578915	-4.5151015	-3.7196777
F	-6.0683294	-1.0905512	4.9589313
F	-1.8864756	5.1136909	-1.5561939
B	2.7253316	0.3448307	0.1892518
C	3.9075226	0.1985560	1.2997565
C	2.3422647	-1.0270124	-0.5745176
C	2.8173244	1.6764306	-0.7325029
C	4.1420118	1.1942146	2.2579556
C	4.7848050	-0.8881024	1.3899096
C	1.9379969	-2.1719186	0.1242779
C	2.4382806	-1.2088584	-1.9571458
C	1.7244226	2.1158846	-1.4931260
C	3.9547997	2.4817701	-0.8548229
C	5.1091335	1.1183964	3.2513197
C	5.7716851	-1.0062525	2.3677161
C	1.6058200	-3.3768148	-0.4803933
C	2.1283407	-2.4033609	-2.6045912
C	1.7271374	3.2692084	-2.2682870
C	4.0015233	3.6456089	-1.6200088
C	5.9335354	-0.0006115	3.3117400
C	1.7031474	-3.4968755	-1.8631866
C	2.8772829	4.0498851	-2.3278636
F	3.4133332	2.3392662	2.2276852
F	4.7487796	-1.8986994	0.4889332

F	1.8912268	-2.1555456	1.4785338
F	2.8777370	-0.2117895	-2.7652123
F	0.5905746	1.3841756	-1.5235556
F	5.1194191	2.1496937	-0.2438674
F	5.2716829	2.1194818	4.1393749
F	6.5810460	-2.0846505	2.3975846
F	1.2156547	-4.4367508	0.2565882
F	2.2369597	-2.5051661	-3.9444648
F	0.6401483	3.6289782	-2.9795954
F	5.1339304	4.3758430	-1.6909263
F	6.8831989	-0.0978764	4.2588950
F	1.3944852	-4.6574350	-2.4681159
F	2.9059302	5.1669620	-3.0760229

Bf_3OCP^- : $B(C_6F_5)_3$ adduct at O of PCO^-
37
Energy = -2664.426466684

P	0.3102129	0.2722720	-1.0258429
C	-0.2503077	-0.7910695	-0.0080828
O	-0.6680901	-1.6577107	0.8026125
B	-1.7366479	-1.3808608	1.9064511
C	-1.3656153	-2.4501399	3.0989551
C	-1.5549121	0.1946659	2.2963215
C	-3.2284815	-1.7967055	1.3623908
C	-1.1700491	-3.7966444	2.7755636
C	-1.2869441	-2.1673406	4.4628949
C	-0.3229962	0.6322288	2.7929617
C	-2.4718741	1.2155957	2.0525022
C	-3.5616606	-2.2399120	0.0821866
C	-4.2925306	-1.7910991	2.2672754
C	-0.8815688	-4.7860405	3.7112713
C	-1.0048904	-3.1259872	5.4342043
C	-0.0178545	1.9602253	3.0601066
C	-2.2114259	2.5616327	2.3068657
C	-4.8477893	-2.6497362	-0.2735782
C	-5.5866629	-2.1892377	1.9581878
C	-0.7946376	-4.4462850	5.0564231
C	-0.9759226	2.9382569	2.8152388
C	-5.8679143	-2.6271053	0.6680076
F	-1.2814280	-4.2117280	1.4904485
F	-1.5002883	-0.9133980	4.9306690
F	0.6584777	-0.2685303	3.0423477
F	-3.6964508	0.9544094	1.5304403
F	-2.6429138	-2.2949234	-0.9083284
F	-4.0848897	-1.3492049	3.5354199
F	-0.6965023	-6.0695175	3.3321876
F	-0.9351930	-2.7873200	6.7404023
F	1.1914798	2.3148457	3.5452402
F	-3.1450894	3.5051010	2.0537497
F	-5.1124951	-3.0710072	-1.5299210
F	-6.5703901	-2.1505075	2.8838785
F	-0.5192600	-5.3864179	5.9823210
F	-0.7061784	4.2348514	3.0642097
F	-7.1142966	-3.0212842	0.3371179

Bf_3PCO^- : $B(C_6F_5)_3$ adduct at P of PCO^-
37
Energy = -2664.434774954

P	-0.7010648	-0.1285448	-0.6032474
C	-0.8561339	-1.2877493	-1.7731241
O	-0.9165887	-2.0859120	-2.6371124
B	-2.4431804	-0.6409553	0.5464431
C	-1.8390455	-1.5156326	1.7887695
C	-2.9925495	0.8368482	0.9955928
C	-3.4851125	-1.4377326	-0.4207246
C	-1.1052616	-2.6898529	1.5640199
C	-1.9519473	-1.1798288	3.1417174
C	-2.1321510	1.8144427	1.5097948
C	-4.3264334	1.2489475	0.9241052
C	-3.9145158	-0.8808097	-1.6340299
C	-4.0392124	-2.6928206	-0.1538692
C	-0.4899603	-3.4335578	2.5629115
C	-1.3498562	-1.8944971	4.1771568
C	-2.5229152	3.0974259	1.8709903
C	-4.7662945	2.5258164	1.2712185
C	-4.7593793	-1.5188976	-2.5329044
C	-4.8889889	-3.3711688	-1.0270424
C	-0.6044368	-3.0283392	3.8887096
C	-3.8583376	3.4636764	1.7429530
C	-5.2500748	-2.7848426	-2.2315242
F	-0.9924750	-3.1889428	0.3118863
F	-2.7001532	-0.1200823	3.5471049
F	-0.8243219	1.5160444	1.7223251
F	-5.3072337	0.3962859	0.5310962
F	-3.5297960	0.3716283	-1.9782093
F	-3.8042201	-3.3308465	1.0220402
F	0.2033332	-4.5548356	2.2656430
F	-1.4983963	-1.5008819	5.4623135
F	-1.6299236	3.9841127	2.3624789
F	-6.0727968	2.8558903	1.1671149
F	-5.1205194	-0.9207285	-3.6895538
F	-5.3776957	-4.5896633	-0.7036104
F	-0.0158520	-3.7345624	4.8753787
F	-4.2652569	4.7021684	2.0863522
F	-6.0739739	-3.4228896	-3.0876840

Bf_3 : strong Lewis acid $B(C_6F_5)_3$
34
Energy = -2209.514301676

B	-0.0002623	0.0009772	-0.0003911
C	-0.0002005	1.5633955	-0.0000504
C	1.3521554	-0.7823976	-0.0040996
C	-1.3524614	-0.7827720	0.0031168
C	-0.9408109	2.3134390	0.7232962
C	0.9407638	2.3135995	-0.7227741
C	2.4657018	-0.3605317	0.7379076
C	1.5346272	-1.9555655	-0.7518184
C	-1.5352297	-1.9550128	0.7522876
C	-2.4654637	-0.3624049	-0.7406366
C	-0.9487704	3.7020745	0.7463010
C	0.9497317	3.7022736	-0.7440958
C	3.6714360	-1.0495558	0.7545567
C	2.7326424	-2.6578513	-0.7811151
C	-2.7333768	-2.6570739	0.7823059

C	-3.6712271	-1.0514055	-0.7567385
C	0.0006033	4.4000233	0.0013408
C	3.8059774	-2.2031260	-0.0166507
C	-3.8063615	-2.2033867	0.0167430
F	-1.8733686	1.6881192	1.4712489
F	1.8723662	1.6885225	-1.4720890
F	2.3832577	0.7426157	1.5106567
F	0.5314917	-2.4301051	-1.5194584
F	-0.5323124	-2.4289322	1.5205094
F	-2.3823276	0.7387008	-1.5161573
F	-1.8536077	4.3768070	1.4716726
F	1.8555259	4.3772149	-1.4680795
F	4.7033050	-0.6210940	1.4973440
F	2.8689953	-3.7622349	-1.5307172
F	-2.8702471	-3.7600938	1.5337747
F	-4.7024753	-0.6245452	-1.5012625
F	0.0007255	5.7357901	0.0016762
F	4.9624769	-2.8718041	-0.0228182
F	-4.9632113	-2.8714497	0.0241873

ButBf$_3$: B(C_6F_5)$_3$ adduct at one ester C=O of But
62
Energy = -2900.304191974

C	1.9056088	-1.7890504	1.1326028
C	1.1639471	-0.8754155	1.9265733
C	2.5404319	-2.5818053	0.4716920
O	0.2234807	-0.1552209	1.4991589
O	1.5250714	-0.8255644	3.1767567
C	3.1922110	-3.5874817	-0.3452292
O	2.7302012	-4.7066927	-0.4599771
O	4.2956070	-3.0962933	-0.9118234
B	-0.3968952	-0.0597289	0.0283698
C	-1.2878769	1.3060902	0.1386470
C	0.8852959	0.0259612	-0.9698311
C	-1.4166203	-1.3112407	-0.1977629
C	-2.1732412	1.4745929	1.2077534
C	-1.3051537	2.3417762	-0.7973006
C	1.8382956	1.0296930	-0.7679450
C	1.1946614	-0.8655296	-1.9981932
C	-1.6597472	-2.3879440	0.6526234
C	-2.2211178	-1.2850492	-1.3411453
C	-2.9891956	2.5887255	1.3731881
C	-2.1122096	3.4715135	-0.6779127
C	3.0046831	1.1624700	-1.5116876
C	2.3467137	-0.7674316	-2.7749950
C	-2.6143611	-3.3714565	0.3974633
C	-3.1839984	-2.2400755	-1.6393952
C	-2.9576130	3.6003666	0.4185040
C	3.2607790	0.2510984	-2.5314670
C	-3.3836067	-3.2989788	-0.7573468
F	-2.2843731	0.5083369	2.1578344
F	-0.5255825	2.2925672	-1.9024940
F	1.6463960	1.9459364	0.2162940
F	0.4017739	-1.9267799	-2.2701950
F	-0.9622483	-2.5454763	1.8128798
F	-2.0547400	-0.2846072	-2.2405787
F	-3.8105947	2.6936151	2.4356660
F	-2.0819860	4.4385443	-1.6135168
F	3.8798026	2.1527148	-1.2603191
F	2.6000648	-1.6729616	-3.7396185
F	-2.7984582	-4.3871002	1.2618776
F	-3.9167279	-2.1574380	-2.7645700
F	-3.7381859	4.6842734	0.5521409
F	4.3819050	0.3459646	-3.2622751
F	-4.3068045	-4.2372180	-1.0200495
C	0.7508706	0.0871179	4.1064071
C	1.2970236	1.4947218	3.9486070
C	0.9449335	-0.5207529	5.4794166
H	-0.2877267	0.0237211	3.7770172
H	1.1644900	1.8634486	2.9292149
H	0.7483109	2.1561069	4.6266063
H	2.3575612	1.5289608	4.2142925
H	0.5726748	-1.5478785	5.5099800
H	2.0007225	-0.5105368	5.7652872
H	0.3817968	0.0732609	6.2057226
C	5.0656976	-4.0307475	-1.7868272
C	5.9564042	-4.8912400	-0.9043614
C	5.8229873	-3.1408585	-2.7539415
H	4.3227875	-4.6436535	-2.3033470
H	5.3624416	-5.4855056	-0.2055678
H	6.5298784	-5.5768170	-1.5365533
H	6.6585205	-4.2661902	-0.3437635
H	5.1378253	-2.5359406	-3.3519130
H	6.5073073	-2.4784518	-2.2148849
H	6.4101016	-3.7697182	-3.4304477

But : the alkyne diisopropyl but-2-ynedioate
28
Energy = -690.7648247102

C	-0.0792620	0.5979981	-0.0859235
C	0.1052798	-0.6002438	-0.0898363
C	-0.3871539	2.0074530	-0.0150766
C	0.4082995	-2.0110954	-0.0257636
O	-1.2332125	2.4582831	0.7377511
O	0.3744823	2.7110490	-0.8680148
O	1.2575199	-2.4682250	0.7196009
O	-0.3627693	-2.7084093	-0.8754487
C	0.1486448	4.1783335	-0.8929551
C	-1.0118621	4.4751218	-1.8312822
C	1.4702664	4.7824726	-1.3324357
H	-0.1013081	4.4713698	0.1298410
H	-1.9287261	3.9945929	-1.4805808
H	-1.1820129	5.5562305	-1.8675814
H	-0.7849616	4.1245229	-2.8431816
H	2.2720799	4.5103273	-0.6405229
H	1.7378042	4.4405232	-2.3372159
H	1.3807786	5.8733880	-1.3492761
C	-0.1487603	-4.1773464	-0.9025794
C	1.0001592	-4.4834518	-1.8520714
C	-1.4792707	-4.7709263	-1.3295622
H	0.1087992	-4.4721900	0.1177870
H	1.9247083	-4.0111548	-1.5105254
H	1.1600212	-5.5661584	-1.8896043
H	0.7663779	-4.1312555	-2.8618199

H	-2.2724721	-4.4919194	-0.6304996
H	-1.7529404	-4.4267429	-2.3319462
H	-1.3991852	-5.8625377	-1.3470718

B : unstable $B(C_6F_5)_3$ adduct at CH of PhCCH
48
Energy = -2518.105278742

C	-0.1191266	-0.1146715	-0.1846607
C	0.7174114	0.5135132	0.5047213
C	-0.8830256	-0.9293782	-1.0145132
C	-1.1871709	-0.5115652	-2.3361331
C	-1.3243729	-2.1964989	-0.5532859
C	-1.8981986	-1.3537391	-3.1745587
H	-0.8423840	0.4584689	-2.6745228
C	-2.0270216	-3.0289671	-1.4093362
H	-1.0901657	-2.4990188	0.4600268
C	-2.3098410	-2.6109530	-2.7153963
H	-2.1266395	-1.0437855	-4.1888591
H	-2.3569530	-4.0047073	-1.0680978
H	-2.8572107	-3.2709612	-3.3818414
H	0.6122283	1.3316138	1.2024837
B	2.4516342	-0.0539331	0.4366764
C	2.6558296	-0.5504384	1.9738538
C	2.5360390	-1.2231421	-0.6931793
C	3.2469898	1.3141380	0.0569179
C	1.8061649	-1.4953354	2.5626779
C	3.6679923	-0.0894534	2.8241281
C	2.1394338	-0.9683490	-2.0111281
C	2.9599860	-2.5354199	-0.4724097
C	4.2277946	1.4030855	-0.9393129
C	3.0183137	2.5224814	0.7291687
C	1.9047203	-1.9258468	3.8793802
C	3.8108711	-0.4991426	4.1487582
C	2.0979105	-1.9237051	-3.0173693
C	2.9373057	-3.5298050	-1.4515252
C	4.8866237	2.5834800	-1.2755201
C	3.6429538	3.7248143	0.4243178
C	2.9181405	-1.4183343	4.6866429
C	2.4939244	-3.2271748	-2.7325399
C	4.5874400	3.7589189	-0.5966121
F	0.8328252	-2.0852253	1.8231685
F	4.6101452	0.7785447	2.3864215
F	1.7755668	0.2907315	-2.3709300
F	3.4465770	-2.9233879	0.7300956
F	4.6295189	0.3075694	-1.6251005
F	2.1608035	2.5637444	1.7866005
F	1.0474302	-2.8392203	4.3734741
F	4.8113040	-0.0181841	4.9093585
F	1.6836397	-1.6030348	-4.2590771
F	3.3455378	-4.7803428	-1.1647354
F	5.8178430	2.5939831	-2.2464352
F	3.3628161	4.8441934	1.1167937
F	3.0409009	-1.8237993	5.9593404
F	2.4588237	-4.1727718	-3.6855258
F	5.2143468	4.9032751	-0.9052378

C : $B(C_6F_5)_3$ adduct at one C=O of dimethyl maleate
52
Energy = -2744.198580145

H	1.2430877	-2.4807410	0.6254954
C	1.8870824	-1.7946036	1.1681854
C	1.1688483	-0.7920017	1.9716473
C	3.2191461	-1.9120924	1.0867896
O	0.2087920	-0.0918756	1.5622259
O	1.5350187	-0.6975184	3.2207355
H	3.6452717	-2.7360837	0.5234426
C	4.2241820	-1.0078574	1.7023656
C	0.8343140	0.2990463	4.0443740
O	5.3141305	-1.3883145	2.0842515
O	3.7974723	0.2715985	1.7450914
H	1.2781841	0.1885018	5.0300361
H	1.0156511	1.2887732	3.6240205
H	-0.2310800	0.0704471	4.0486581
C	4.7308193	1.2233425	2.3281759
H	5.6958833	1.1455956	1.8250928
H	4.8480409	1.0137635	3.3935027
H	4.2756368	2.1976315	2.1624389
B	-0.3849120	-0.0261141	0.0795571
C	-1.2975422	1.3261651	0.1312636
C	0.9286132	0.0457528	-0.8797542
C	-1.3911593	-1.2906184	-0.1481854
C	-2.2127845	1.5070500	1.1727439
C	-1.3113570	2.3322396	-0.8361724
C	1.8530238	1.0773671	-0.6793029
C	1.3069937	-0.8916318	-1.8428370
C	-1.6949904	-2.3188945	0.7415061
C	-2.1315591	-1.3159589	-1.3343511
C	-3.0546600	2.6084673	1.2836379
C	-2.1430668	3.4483595	-0.7707887
C	3.0594532	1.1826752	-1.3568995
C	2.5022999	-0.8218731	-2.5546080
C	-2.6486200	-3.3037022	0.4872101
C	-3.0897784	-2.2748952	-1.6344499
C	-3.0180542	3.5921455	0.3003379
C	3.3888597	0.2211247	-2.3083832
C	-3.3528419	-3.2831884	-0.7101643
F	-2.3274642	0.5665610	2.1468247
F	-0.5001437	2.2658446	-1.9175356
F	1.5971238	2.0335909	0.2468778
F	0.5401704	-1.9780465	-2.0997275
F	-1.0566380	-2.4249321	1.9427628
F	-1.9070179	-0.3619649	-2.2693425
F	-3.9054696	2.7276672	2.3210661
F	-2.1074245	4.3885346	-1.7332748
F	3.9159931	2.1875518	-1.0959407
F	2.8191194	-1.7665725	-3.4585082
F	-2.8915846	-4.2707663	1.3916457
F	-3.7593100	-2.2440201	-2.8001381
F	-3.8220455	4.6639856	0.3827084
F	4.5490106	0.2981377	-2.9757314
F	-4.2733320	-4.2230157	-0.9743046

D0 : Ge..C adduct of **1** and the alkene Mal

94

Energy = -4306.577931822

Ge	0.0797096	0.3464592	-0.2422585
P	-0.7182767	-0.5356305	-2.3043554
N	1.1440175	-0.8185635	0.8938233
N	-1.6510510	-0.0855853	0.8534060
C	-1.4751997	0.9037661	-2.6457399
C	0.8042332	-1.1350187	2.1676642
C	2.4235409	-1.3207495	0.4116466
C	-1.5767825	-0.3557780	2.1537792
C	-2.9422900	-0.0741069	0.1985724
O	-2.0679931	1.8529900	-2.9963014
C	-0.3898963	-0.7775409	2.7780047
C	1.7485532	-1.9903951	2.9764313
C	2.4573091	-2.5229737	-0.3151546
C	3.6071097	-0.6180802	0.7249469
C	-2.7978796	-0.2568421	3.0316376
C	-3.3711375	-1.2967588	-0.3765517
C	-3.7483174	1.0806692	0.1227785
H	-0.4684441	-1.0076212	3.8331343
H	1.4274533	-2.0107728	4.0188142
H	1.7341506	-3.0145362	2.5877616
H	2.7822170	-1.6508077	2.9139365
C	3.7050606	-3.0523133	-0.6689352
C	1.1974353	-3.2581472	-0.7389618
C	4.8272317	-1.1893830	0.3546395
C	3.6096849	0.7396793	1.4108229
H	-3.7178641	-0.4536141	2.4798892
H	-2.7218664	-0.9403801	3.8795232
H	-2.8457346	0.7661636	3.4229152
C	-4.5820538	-1.3240676	-1.0685711
C	-2.5655001	-2.5743991	-0.2044810
C	-4.9512883	1.0004100	-0.5934481
C	-3.4329102	2.3856395	0.8360014
C	4.8842234	-2.4035058	-0.3246638
H	3.7469916	-3.9869536	-1.2213313
C	0.9954656	-4.5569182	0.0622689
C	1.2054522	-3.5522300	-2.2491825
H	0.3447141	-2.6047732	-0.5289441
H	5.7488359	-0.6662960	0.5948842
C	4.3848248	1.7739365	0.5741138
C	4.2093691	0.6892699	2.8304380
H	2.5758749	1.0812398	1.4842080
C	-5.3646003	-0.1788283	-1.1952675
H	-4.9169077	-2.2522304	-1.5204668
C	-2.6642270	-3.5181988	-1.4100204
C	-2.9760613	-3.3194795	1.0812574
H	-1.5170042	-2.2795938	-0.0896916
H	-5.5756354	1.8866385	-0.6660646
C	-4.4926425	2.6885962	1.9159522
C	-3.3703183	3.5796498	-0.1373421
H	-2.4669932	2.2852892	1.3385527
H	5.8444773	-2.8316302	-0.5990638
H	0.8870707	-4.3563933	1.1322979
H	0.0948818	-5.0797044	-0.2773349
H	1.8501141	-5.2289381	-0.0733128
H	1.3823693	-2.6390363	-2.8249146
H	1.9868113	-4.2754750	-2.5062368
H	0.2433266	-3.9736336	-2.5568504
H	4.0207897	1.8040140	-0.4533765
H	4.2612995	2.7702681	1.0092779
H	5.4556638	1.5413861	0.5588018
H	3.6062107	0.0906016	3.5181174
H	5.2207042	0.2678204	2.8080863
H	4.2776305	1.7023529	3.2415187
H	-6.2987278	-0.2135472	-1.7491419
H	-2.4146557	-2.9969017	-2.3390132
H	-1.9706405	-4.3554877	-1.2826370
H	-3.6692821	-3.9423016	-1.5085121
H	-2.8340334	-2.7002607	1.9700480
H	-4.0316915	-3.6095150	1.0328544
H	-2.3739630	-4.2272475	1.2000415
H	-4.6088942	1.8598115	2.6189810
H	-4.2037394	3.5820259	2.4804081
H	-5.4695311	2.8783894	1.4569994
H	-2.7000127	3.3961506	-0.9809844
H	-4.3626531	3.7924154	-0.5505556
H	-3.0282946	4.4755503	0.3922878
H	0.6976605	3.8333826	-1.5464011
C	0.8052368	2.8360202	-1.1376167
C	1.6922303	1.9425623	-1.6447311
C	-0.0411012	2.3464127	-0.0095263
O	1.7779249	0.7059511	-1.1780993
O	2.5461640	2.2689735	-2.6557347
H	-1.1067580	2.5406487	-0.1495067
C	0.2654563	2.7836835	1.3999588
C	3.1891312	1.1525194	-3.3112516
O	-0.4844635	2.6364985	2.3554639
O	1.4782408	3.3750779	1.5180263
H	3.8023447	1.6028472	-4.0925247
H	3.8106225	0.5918822	-2.6080337
H	2.4409863	0.4838193	-3.7472953
C	1.8095485	3.8027606	2.8611180
H	1.8159619	2.9451450	3.5378993
H	1.0804934	4.5365926	3.2118543
H	2.8013415	4.2463797	2.7810329

D : Ge..C adduct of **1** and **C**

128

Energy = -6516.134710910

Ge	-2.9831343	0.0467286	-0.1701197
P	-1.8523388	0.0981999	1.8641465
C	-1.2785965	1.6121703	1.4267436
O	-0.8552744	2.6814441	1.2485364
N	-4.4846788	1.2847468	-0.0164319
C	-5.6958417	0.9842074	-0.5029646
C	-4.2562705	2.5394561	0.6792674
C	-6.0542846	-0.3021306	-0.9162182
C	-6.7384088	2.0610780	-0.6289811
C	-4.4567702	2.5520009	2.0800348
C	-3.8404508	3.6880745	-0.0221160
C	-5.3222232	-1.4786368	-0.8054028
H	-7.0643813	-0.4114431	-1.2899596
H	-7.7385921	1.6410083	-0.5095578

H	-6.5876646	2.8727889	0.0826023	H	-3.8584342	-3.8204529	-4.5937853
H	-6.6703338	2.4783507	-1.6409655	H	-3.2254273	-5.1743651	-3.6491332
C	-4.1925573	3.7389432	2.7670301	H	-0.9675693	-2.3206526	-2.7868681
C	-5.0015322	1.3421433	2.8251077	H	-0.9982667	-4.0534503	-3.1452843
C	-3.5947388	4.8514833	0.7180325	H	-1.5812812	-2.8978540	-4.3462959
C	-3.6851964	3.7448817	-1.5339829	H	0.2256262	1.3269554	-1.8800007
C	-6.0438871	-2.7593018	-1.1278833	C	-0.4285809	0.5256620	-1.5645925
N	-4.0175613	-1.5519546	-0.4407292	C	0.1278152	-0.6033112	-1.0622555
C	-3.7577367	4.8792409	2.0962132	C	-1.8912237	0.7267637	-1.6896051
H	-4.3295837	3.7730052	3.8427730	O	1.3926913	-0.9202816	-0.9503697
C	-6.5420248	1.3725406	2.8837771	O	-0.7215884	-1.5727808	-0.5805538
C	-4.4309476	1.2001715	4.2425053	H	-2.1127850	1.7964976	-1.5769513
H	-4.7103179	0.4503665	2.2591446	C	-2.6285473	0.3686737	-2.9654169
H	-3.2678018	5.7470767	0.1979752	C	-0.0905636	-2.6091106	0.2166530
C	-2.2642791	4.1916124	-1.9397508	O	-3.8520282	0.3522426	-3.0350771
C	-4.7117768	4.7083848	-2.1629119	O	-1.8237583	0.1362167	-4.0085359
H	-3.8789693	2.7451754	-1.9383220	H	-0.9132954	-3.1943164	0.6191674
H	-5.8274995	-3.5448620	-0.4013418	H	0.5489899	-3.2230499	-0.4192524
H	-7.1186768	-2.5808474	-1.1675428	H	0.5011956	-2.1586805	1.0149208
H	-5.7162521	-3.1245026	-2.1057356	C	-2.5116354	-0.1203536	-5.2638647
C	-3.4517607	-2.8688051	-0.1811084	H	-3.1387030	-1.0088018	-5.1680538
H	-3.5512245	5.7899080	2.6508392	H	-1.7218793	-0.2750365	-5.9950892
H	-6.9892789	1.3690787	1.8873916	H	-3.1302202	0.7394200	-5.5275158
H	-6.9132364	0.4950095	3.4242152	B	2.5285198	-0.2405828	-1.6870055
H	-6.8866244	2.2703838	3.4083212	C	3.8468034	-1.2016213	-1.4203449
H	-3.3375355	1.2061229	4.2316494	C	2.0594465	-0.2040826	-3.2675616
H	-4.7786407	2.0044146	4.8992038	C	2.9328842	1.2090919	-1.0177336
H	-4.7679252	0.2551116	4.6806951	C	4.1230432	-1.6679754	-0.1309160
H	-1.4834690	3.6378602	-1.4092947	C	4.8037705	-1.5567189	-2.3730772
H	-2.1164198	4.0593430	-3.0166379	C	1.8349897	-1.4185321	-3.9221681
H	-2.1174957	5.2521047	-1.7104925	C	1.7329570	0.9182200	-4.0253901
H	-5.7382107	4.4416575	-1.8982991	C	2.4815472	1.6891269	0.2127253
H	-4.5328189	5.7338089	-1.8223816	C	3.9183213	1.9992243	-1.6148366
H	-4.6204862	4.6926406	-3.2541513	C	5.2263859	-2.4519119	0.1943508
C	-3.4689929	-3.3399927	1.1494312	C	5.9273869	-2.3321910	-2.0913628
C	-2.9622042	-3.6559143	-1.2441886	C	1.3686627	-1.5282768	-5.2264987
C	-2.9801718	-4.6271376	1.3992693	C	1.2661695	0.8616408	-5.3365253
C	-4.0394932	-2.5177500	2.2956411	C	2.9246898	2.8830080	0.7829783
C	-2.4748430	-4.9310667	-0.9333704	C	4.3912555	3.1938296	-1.0863018
C	-2.9654233	-3.2229737	-2.7039866	C	6.1404371	-2.7899911	-0.7970394
C	-2.4845909	-5.4187385	0.3680585	C	1.0903652	-0.3724069	-5.9468588
H	-2.9859516	-5.0111990	2.4141108	C	3.8828045	3.6458900	0.1280767
C	-3.2245742	-2.6648708	3.5897858	F	3.3092568	-1.3451107	0.9053600
C	-5.5199987	-2.8590747	2.5527301	F	4.6870997	-1.1600904	-3.6647375
H	-4.0064423	-1.4620118	2.0025940	F	2.0717211	-2.5909846	-3.2797357
H	-2.0832703	-5.5520754	-1.7336206	F	1.8049891	2.1677115	-3.4946100
C	-3.7563160	-4.2192449	-3.5791459	F	1.5790117	0.9987443	0.9508720
C	-1.5377037	-3.1093138	-3.2733866	F	4.4736195	1.5977048	-2.7858867
H	-3.4502776	-2.2442073	-2.7715497	F	5.4259296	-2.8752111	1.4613556
H	-2.1019549	-6.4127367	0.5802915	F	6.8086833	-2.6475055	-3.0637694
H	-2.1583814	-2.4923502	3.4139603	F	1.1746355	-2.7363391	-5.7989170
H	-3.5719336	-1.9389949	4.3308811	F	0.9427514	1.9895294	-6.0056650
H	-3.3414164	-3.6626912	4.0248675	F	2.4413231	3.2973662	1.9739457
H	-6.1435641	-2.6363798	1.6827670	F	5.3341184	3.9150894	-1.7280231
H	-5.6315676	-3.9219892	2.7926328	F	7.2179875	-3.5428543	-0.5056616
H	-5.8983941	-2.2745844	3.3982469	F	0.6202503	-0.4510622	-7.2097173
H	-4.7568917	-4.4262259	-3.1886083	F	4.3243034	4.7989842	0.6659909

E : $GePC_3$-ring adduct of **1** and **C**
128
Energy = -6516.149084554

Ge	3.1104826	-0.0830396	0.7438827
P	2.7438677	-0.2840181	2.9251450
N	4.0018627	-1.5543617	-0.1766311
N	4.3234006	1.2634890	0.1109190
C	0.9917867	-0.6272804	2.5765613
C	5.3303738	-1.5365783	-0.3293172
C	3.1759665	-2.7088484	-0.4609783
C	5.6297549	0.9398184	-0.0149756
C	3.8848299	2.6407632	-0.0513766
O	0.1369095	-0.7317657	3.4552270
C	6.0918616	-0.3740205	-0.1281775
C	6.0621746	-2.7847365	-0.7432587
C	3.0119146	-3.7258217	0.5045649
C	2.4754481	-2.7288172	-1.6897895
C	6.6558425	2.0427614	-0.0307949
C	3.8224584	3.5049323	1.0578266
C	3.5405883	3.0665773	-1.3544138
H	7.1655845	-0.4917155	-0.2136537
H	6.6238721	-3.1713493	0.1144736
H	5.3799405	-3.5608041	-1.0905432
H	6.7847585	-2.5528311	-1.5297043
C	2.1124121	-4.7606186	0.2095318
C	3.7827628	-3.7837828	1.8151477
C	1.5831196	-3.7776989	-1.9236401
C	2.7139281	-1.6697953	-2.7546052
H	6.2602430	2.9638901	-0.4594966
H	6.9535951	2.2566356	1.0024977
H	7.5438821	1.7302367	-0.5822734
C	3.4204977	4.8280144	0.8293816
C	4.1885257	3.0904520	2.4734778
C	3.1536377	4.3974946	-1.5244698
C	3.5943360	2.1207988	-2.5456703
C	1.3960743	-4.7847616	-0.9809358
H	1.9753826	-5.5590595	0.9331404
C	4.7831590	-4.9582469	1.8039276
C	2.8459011	-3.9291963	3.0262215
H	4.3346791	-2.8456367	1.9342982
H	1.0189849	-3.8023591	-2.8491860
C	1.4665658	-1.3548707	-3.5925483
C	3.8838628	-2.0786230	-3.6713594
H	3.0173251	-0.7514174	-2.2402926
C	3.0969522	5.2751224	-0.4451151
H	3.3663065	5.5138657	1.6699204
C	2.9880954	3.2499715	3.4225261
C	5.3890176	3.8992648	3.0016528
H	4.4648663	2.0308829	2.4649583
H	2.8764411	4.7482250	-2.5124005
C	5.0082881	2.0444774	-3.1555508
C	2.5815748	2.4728918	-3.6444873
H	3.3426471	1.1233877	-2.1683686
H	0.6919675	-5.5878679	-1.1783781
H	5.4603963	-4.9163277	0.9472319
H	5.3823213	-4.9480397	2.7205218
H	4.2480315	-5.9132946	1.7598938
H	2.1338238	-3.1027593	3.0731124
H	2.2960004	-4.8760638	2.9884568
H	3.4309453	-3.9168062	3.9514251
H	0.6045583	-1.1039116	-2.9688610
H	1.6694308	-0.5020999	-4.2460638
H	1.1899811	-2.2005973	-4.2306614
H	4.8075061	-2.2246967	-3.1053920
H	3.6512414	-3.0139712	-4.1916090
H	4.0624809	-1.3019711	-4.4225282
H	2.7885967	6.3054376	-0.5987114
H	2.1445366	2.6421186	3.0877735
H	3.2620360	2.9215364	4.4303309
H	2.6733885	4.2982257	3.4771582
H	6.2564000	3.8241314	2.3389381
H	5.1318334	4.9600272	3.0947702
H	5.6781569	3.5342719	3.9928602
H	5.7469968	1.6655469	-2.4458807
H	5.0037113	1.3750525	-4.0227979
H	5.3325945	3.0356650	-3.4912183
H	1.5703421	2.5468698	-3.2412709
H	2.8424777	3.4146489	-4.1400596
H	2.5970682	1.6925034	-4.4123736
H	0.9666643	-1.9791043	0.9166180
C	0.5734954	-0.9634156	1.0658316
C	-0.9047797	-1.0702845	0.9056809
C	1.2454989	-0.0531514	0.0260262
O	-1.5282301	-0.1883351	0.2563059
O	-1.5944340	-2.0628516	1.3941564
H	1.2006558	-0.4915837	-0.9689014
C	0.7229524	1.3578895	-0.0734161
C	-0.9582898	-3.2072586	2.0451207
O	0.6262328	1.9692169	-1.1185268
O	0.4233289	1.8774102	1.1335502
H	-0.2672356	-3.6782770	1.3426861
H	-0.4611254	-2.8688624	2.9520877
H	-1.7883167	-3.8728266	2.2715093
C	-0.0808694	3.2397854	1.0988320
H	-0.9403857	3.2946010	0.4294459
H	0.7097117	3.9085062	0.7538103
H	-0.3684687	3.4589480	2.1249702
B	-3.0868538	-0.1820876	-0.0400953
C	-3.7731381	-0.1849843	1.4397048
C	-3.2623473	1.1859887	-0.9204246
C	-3.4900790	-1.4203628	-1.0314630
C	-3.5116696	0.8673270	2.3219243
C	-4.5281595	-1.2211169	1.9914442
C	-4.1974820	2.1989125	-0.7047627
C	-2.4445541	1.3765512	-2.0394590
C	-2.6475878	-2.3468863	-1.6418080
C	-4.8258153	-1.5093587	-1.4341379
C	-3.9792975	0.9263829	3.6293029
C	-5.0237109	-1.2047022	3.2936231
C	-4.2809761	3.3442313	-1.4944921
C	-2.4855009	2.5065668	-2.8485396
C	-3.0832728	-3.3050270	-2.5552054
C	-5.3092036	-2.4455414	-2.3398129

C	-4.7517154	-0.1210836	4.1203608
C	-3.4117716	3.5056184	-2.5679116
C	-4.4255356	-3.3595865	-2.9079457
F	-2.7500908	1.9114978	1.9146083
F	-4.7975311	-2.3461166	1.2842632
F	-5.0975482	2.1145689	0.3026742
F	-1.5628937	0.4138307	-2.4011922
F	-1.3085172	-2.3673062	-1.3882785
F	-5.7279883	-0.6399816	-0.9178178
F	-3.6950573	1.9791872	4.4197312
F	-5.7475491	-2.2402467	3.7652201
F	-5.1977221	4.2966154	-1.2302992
F	-1.6565323	2.6368296	-3.9022499
F	-2.2078829	-4.1744585	-3.1045118
F	-6.6135455	-2.4800938	-2.6722934
F	-5.2191257	-0.0908777	5.3796074
F	-3.4786027	4.6067175	-3.3371225
F	-4.8639518	-4.2766981	-3.7876023

F : $GePC_3$-ring adduct of **1** and Mal
94
Energy = -4306.594640209

Ge	-0.3254604	-0.1098776	-0.2221185
P	-0.8181427	-0.2636150	-2.3916551
N	1.2166840	-0.9180854	0.6643631
N	-1.6655247	-0.9246403	0.9690806
C	-1.0517203	1.5379758	-2.4776905
C	1.1262531	-1.5622081	1.8427860
C	2.4689843	-0.8921848	-0.0721953
C	-1.3665335	-1.5041655	2.1367088
C	-3.0232375	-0.9189707	0.4612082
O	-1.5540820	2.0888257	-3.4601951
C	-0.0563713	-1.7355804	2.5616097
C	2.3568555	-2.1871646	2.4528702
C	2.5909225	-1.7417655	-1.1928633
C	3.5140569	-0.0322151	0.3224445
C	-2.4665066	-1.9710484	3.0558720
C	-3.3766215	-1.9360956	-0.4545442
C	-3.9393561	0.0805492	0.8455421
H	0.0472596	-2.2344527	3.5180617
H	2.1053268	-3.1742645	2.8481719
H	3.1738933	-2.2773144	1.7384299
H	2.6945539	-1.5724440	3.2933674
C	3.7646536	-1.6713084	-1.9481630
C	1.5342377	-2.7841229	-1.5263763
C	4.6714344	-0.0114376	-0.4633536
C	3.4801103	0.8360367	1.5730121
H	-3.4163206	-2.0920157	2.5353264
H	-2.1842521	-2.9131083	3.5312666
H	-2.5982969	-1.2277863	3.8499088
C	-4.6678523	-1.9261387	-0.9850052
C	-2.4065860	-3.0515735	-0.8116899
C	-5.2220727	0.0431810	0.2818692
C	-3.6264856	1.1694578	1.8625403
C	4.7956604	-0.8062363	-1.5954135
H	3.8753742	-2.3080502	-2.8194651
C	1.7691473	-4.0700982	-0.7097197
C	1.4427380	-3.1033930	-3.0231757
H	0.5589245	-2.3850957	-1.2263751
H	5.4872060	0.6475434	-0.1791466
C	3.6624938	2.3303456	1.2514822
C	4.5932810	0.4173926	2.5585022
H	2.5087729	0.7109242	2.0613497
C	-5.5858934	-0.9424785	-0.6257689
H	-4.9584126	-2.6941451	-1.6942373
C	-2.5700724	-3.5582855	-2.2500162
C	-2.5203961	-4.2216221	0.1851179
H	-1.3934388	-2.6438373	-0.7233554
H	-5.9439774	0.8041273	0.5650602
C	-4.4631685	0.9915486	3.1463488
C	-3.9041361	2.5781595	1.2963937
H	-2.5675842	1.1013340	2.1335809
H	5.6993589	-0.7620591	-2.1971741
H	1.7383403	-3.8744317	0.3658069
H	0.9975594	-4.8124430	-0.9424938
H	2.7469445	-4.5026326	-0.9490839
H	1.3095627	-2.1887130	-3.6069159
H	2.3358131	-3.6287764	-3.3794705
H	0.5828306	-3.7546449	-3.2075901
H	2.8801293	2.6937244	0.5869331
H	3.6536754	2.9101049	2.1811915
H	4.6287565	2.5011448	0.7639640
H	4.5861621	-0.6541980	2.7705402
H	5.5766097	0.6665208	2.1445218
H	4.4809019	0.9596640	3.5039872
H	-6.5839897	-0.9469647	-1.0547408
H	-2.5088041	-2.7300905	-2.9609803
H	-1.7722943	-4.2724181	-2.4793549
H	-3.5236273	-4.0792924	-2.3893372
H	-2.3133278	-3.9024042	1.2097142
H	-3.5288576	-4.6492221	0.1592102
H	-1.8054428	-5.0098385	-0.0757091
H	-4.3281970	0.0038614	3.5923396
H	-4.1795597	1.7472950	3.8869856
H	-5.5290624	1.1160070	2.9252532
H	-3.4524762	2.7318945	0.3118656
H	-4.9812523	2.7439229	1.1880284
H	-3.5151131	3.3405290	1.9796059
H	-1.0667372	3.4183391	-1.4188503
C	-0.5174127	2.4799143	-1.3176695
C	0.9411463	2.6948105	-1.6221215
C	-0.6976830	1.8281497	0.0472432
O	1.8196088	1.9319967	-1.2579325
O	1.1415483	3.7828714	-2.3938942
H	-1.7719574	1.7418156	0.2406287
C	-0.1238466	2.4755530	1.2635166
C	2.4978159	4.0119708	-2.8591707
O	-0.1497383	1.9785286	2.3794339
O	0.3861817	3.7057984	1.0126899
H	2.4537255	4.0700293	-3.9480092
H	2.8312962	4.9645810	-2.4422852
H	3.1432373	3.1959139	-2.5325500
C	0.8817810	4.4211122	2.1729437
H	1.5093915	3.7677758	2.7791917

H	0.0364379	4.7766791	2.7673686
H	1.4515161	5.2579773	1.7714951

L[1]GeOCPBf$_3$: B(C_6F_5)$_3$ adduct at P of L[1]GeOCP
110
Energy = -5981.429074943

Ge	-2.5478119	0.2893879	-0.0239696
P	1.5512341	0.0658101	2.0112065
N	-3.5556146	1.1423009	1.3984590
N	-3.5034455	-1.4195322	0.2313848
C	0.0590834	-0.3724953	1.5801395
C	-4.1554881	0.4891665	2.4213830
C	-3.6780494	2.5844502	1.3212038
C	-4.2613586	-1.7381771	1.2815262
C	-3.2548944	-2.3270673	-0.8676456
O	-1.1055160	-0.6638640	1.4049389
C	-4.4419043	-0.8701040	2.3749926
C	-4.5714483	1.2796145	3.6330386
C	-2.5752291	3.4140044	1.5989797
C	-4.9221800	3.1204329	0.9191270
C	-4.9951755	-3.0529232	1.3260034
C	-2.4041438	-3.4396552	-0.6894069
C	-3.7976852	-1.9992997	-2.1293072
H	-4.9683727	-1.2827790	3.2278628
H	-3.7362572	1.8927809	3.9862928
H	-5.3892433	1.9660396	3.3948276
H	-4.8955701	0.6090233	4.4301904
C	-2.7588885	4.8002874	1.5240313
C	-1.1983117	2.8681071	1.9269476
C	-5.0546793	4.5105933	0.8649229
C	-6.0862217	2.2474050	0.4710840
H	-4.8882902	-3.6117191	0.3966398
H	-4.6148659	-3.6621195	2.1520262
H	-6.0562973	-2.8683205	1.5194495
C	-2.1045524	-4.2130793	-1.8166793
C	-1.8007559	-3.8376292	0.6494825
C	-3.4657672	-2.8084757	-3.2193766
C	-4.7889412	-0.8585026	-2.3041888
C	-3.9877168	5.3488620	1.1768131
H	-1.9197858	5.4551888	1.7408158
C	-0.6711930	3.3711673	3.2811031
C	-0.2133535	3.2110610	0.7940469
H	-1.2696124	1.7792168	1.9977283
H	-6.0042077	4.9417269	0.5597737
C	-6.3039237	2.4203874	-1.0446207
C	-7.3855456	2.5505530	1.2368628
H	-5.8282411	1.2000382	0.6531959
C	-2.6214324	-3.9041879	-3.0692862
H	-1.4454217	-5.0693964	-1.7057716
C	-0.2628447	-3.8156422	0.6020621
C	-2.2740529	-5.2419132	1.0759994
H	-2.1187876	-3.1152684	1.4060855
H	-3.8744561	-2.5782088	-4.1979956
C	-6.2293322	-1.3974920	-2.2013493
C	-4.5808459	-0.0681713	-3.6028935
H	-4.6542827	-0.1550083	-1.4710779
H	-4.1103456	6.4272144	1.1309396
H	-1.3575871	3.1151413	4.0951490
H	0.3003949	2.9118254	3.4895184
H	-0.5397970	4.4585759	3.2758050
H	-0.5603827	2.8052609	-0.1620083
H	-0.1130403	4.2962740	0.6834521
H	0.7711831	2.7894256	1.0087375
H	-5.3857642	2.2085733	-1.6010719
H	-7.0912305	1.7464422	-1.3987829
H	-6.6045403	3.4477686	-1.2760136
H	-7.2570361	2.4245995	2.3164555
H	-7.7209161	3.5770946	1.0552059
H	-8.1820092	1.8747748	0.9073139
H	-2.3663852	-4.5161903	-3.9295205
H	0.1195750	-2.8511119	0.2679901
H	0.1440048	-4.0238383	1.5969877
H	0.1099998	-4.5856810	-0.0814587
H	-3.3640690	-5.3251614	1.0904217
H	-1.8903733	-6.0006139	0.3854099
H	-1.8948190	-5.4767105	2.0762657
H	-6.3958708	-1.9022021	-1.2442569
H	-6.9528007	-0.5806851	-2.2928767
H	-6.4213348	-2.1199169	-3.0021583
H	-3.5593670	0.3172215	-3.6690568
H	-4.7730670	-0.6862088	-4.4856144
H	-5.2751583	0.7774011	-3.6386306
B	2.8633211	-0.1428255	0.2206754
C	2.9530325	-1.7390866	-0.0512513
C	2.1304278	0.7358401	-0.9269375
C	4.2431136	0.4702563	0.8338195
C	3.1802207	-2.6507478	0.9864423
C	2.8783614	-2.3282830	-1.3174439
C	0.9304773	0.3115213	-1.5154015
C	2.6105967	1.9426313	-1.4504001
C	4.2962056	1.6572472	1.5763237
C	5.4906496	-0.1430594	0.6634235
C	3.2482637	-4.0279674	0.8139811
C	2.9389595	-3.7017829	-1.5378940
C	0.2538101	0.9965195	-2.5139726
C	1.9490976	2.6817910	-2.4324619
C	5.4513322	2.1756735	2.1475086
C	6.6762725	0.3438321	1.2120764
C	3.1164767	-4.5632014	-0.4625794
C	0.7600962	2.2100858	-2.9705317
C	6.6589548	1.5087122	1.9681458
F	3.3700748	-2.2055269	2.2532440
F	2.7725337	-1.5662365	-2.4339104
F	0.3684818	-0.8620222	-1.1281591
F	3.7896098	2.4688737	-1.0462990
F	3.1737685	2.4005730	1.7483777
F	5.6268683	-1.2584843	-0.0931578
F	3.4375241	-4.8473488	1.8674661
F	2.8325996	-4.2030926	-2.7849318
F	-0.8729124	0.4951867	-3.0587877
F	2.4669237	3.8435046	-2.8755259
F	5.4180851	3.3235843	2.8515073
F	7.8406526	-0.3008265	1.0053381
F	3.1610219	-5.8936870	-0.6529429

F	0.1168875	2.8996199	-3.9270433
F	7.7910509	1.9935558	2.5030269

L^1Ge^+.Tol : toluene adduct of cation L^1Ge^+

88

Energy = -3588.687157190

Ge	2.6475490	0.5706534	0.1943914
N	3.1543198	2.0006730	-1.0186650
N	3.8410643	-0.6569399	-0.7279537
C	3.9735980	1.9602093	-2.0902765
C	2.5350425	3.2558643	-0.6205646
C	4.5904053	-0.4483103	-1.8302992
C	3.8754925	-1.9488988	-0.0650523
C	4.6300453	0.7906129	-2.4774803
C	4.1864970	3.2199123	-2.8794396
C	1.2496521	3.5583256	-1.1053570
C	3.2111534	4.0692119	0.3078315
C	5.4049266	-1.5825146	-2.3886305
C	3.0830787	-2.9943385	-0.5718655
C	4.6402889	-2.0700678	1.1138629
H	5.2603134	0.8568039	-3.3556265
H	3.2294717	3.6141967	-3.2366140
H	4.6298712	3.9956324	-2.2459019
H	4.8418833	3.0353117	-3.7308288
C	0.6220187	4.7012320	-0.5987607
C	0.5525127	2.6705575	-2.1254629
C	2.5392308	5.2004490	0.7828893
C	4.6142732	3.7351594	0.7947961
H	5.9282000	-2.1193352	-1.5934425
H	4.7489077	-2.3064883	-2.8852998
H	6.1242208	-1.2107012	-3.1191109
C	3.1097974	-4.2144452	0.1115454
C	2.1737200	-2.7869055	-1.7745369
C	4.6368006	-3.3139801	1.7532697
C	5.4226072	-0.8910923	1.6805599
C	1.2538807	5.5075473	0.3444279
H	-0.3713584	4.9645286	-0.9486346
C	-0.1218013	3.4805457	-3.2445854
H	1.3125684	2.0348141	-2.5937825
C	-0.4622151	1.7440236	-1.4319368
H	3.0287284	5.8492659	1.5027848
C	4.5687027	3.0931234	2.1931195
C	5.5407508	4.9623113	0.7875249
H	5.0445869	2.9970201	0.1089628
C	3.8869917	-4.3767507	1.2545841
H	2.5141134	-5.0450401	-0.2529150
C	0.7774523	-2.3379358	-1.3027149
C	2.0728955	-4.0237325	-2.6788846
H	2.5855439	-1.9708163	-2.3779412
H	5.2176843	-3.4516106	2.6586824
C	6.7295907	-0.6279102	0.9089256
C	5.7209640	-1.0224645	3.1791863
H	4.7891997	0.0044177	1.5610942
H	0.7459091	6.3856414	0.7320317
H	0.5892436	4.1571893	-3.7293580
H	-0.5246679	2.8021032	-4.0032714
H	-0.9532525	4.0801321	-2.8609773
H	-1.2433913	2.3310883	-0.9376864
H	-0.9362125	1.0754251	-2.1580029
H	0.0208257	1.1298768	-0.6616499
H	3.9425256	2.1925145	2.1979579
H	5.5754830	2.8160489	2.5229254
H	4.1440144	3.7894158	2.9240664
H	5.5661527	5.4356167	-0.1992905
H	5.2173168	5.7131971	1.5151708
H	6.5594488	4.6610192	1.0517018
H	3.8999413	-5.3333872	1.7683199
H	0.8381356	-1.4300281	-0.6890513
H	0.1282931	-2.1308476	-2.1597802
H	0.3100675	-3.1167446	-0.6912264
H	3.0629766	-4.3727348	-2.9896613
H	1.5634411	-4.8510344	-2.1748307
H	1.4957104	-3.7799242	-3.5761963
H	6.5474661	-0.3830925	-0.1400479
H	7.2675656	0.2120216	1.3606413
H	7.3771876	-1.5100285	0.9478654
H	4.8209002	-1.2525385	3.7558939
H	6.4603486	-1.8072701	3.3703909
H	6.1373241	-0.0817441	3.5519777
C	0.6719722	-1.2168443	2.3447771
C	1.8388413	-0.9623873	3.0642241
C	2.1387796	0.3385201	3.4786793
C	1.2654519	1.3806351	3.1580543
C	0.0975681	1.1197293	2.4399801
C	-0.2252306	-0.1825888	2.0328668
H	0.4455102	-2.2340490	2.0355268
H	2.5094793	-1.7822061	3.3015889
H	3.0404303	0.5370352	4.0502097
H	1.4902632	2.3964193	3.4694353
H	-0.5795601	1.9356255	2.2002555
C	-1.5203126	-0.4760586	1.3186239
H	-2.0350490	0.4455005	1.0362717
H	-1.3543250	-1.0719590	0.4151464
H	-2.1910364	-1.0524919	1.9665183

L^1Ge^+ : Lewis-acidic cation

73

Energy = -3316.936822720

Ge	2.9153957	0.4233788	0.2544118
N	3.2299064	1.9244843	-0.8999701
N	4.1107968	-0.6867948	-0.7569212
C	4.0094482	1.9892727	-2.0017497
C	2.4836503	3.1014196	-0.4747634
C	4.8527564	-0.3575178	-1.8374091
C	4.1849350	-2.0382049	-0.2168001
C	4.7840975	0.9072882	-2.4252836
C	4.0389907	3.2669532	-2.7886377
C	1.1331289	3.2187713	-0.8443364
C	3.1389612	4.0406758	0.3462212
C	5.7750478	-1.3891280	-2.4186056
C	3.3480079	-3.0230001	-0.7782041
C	5.0342970	-2.2872018	0.8745087
H	5.3967160	1.0658873	-3.3039553
H	3.0270560	3.5419527	-3.1034310

H	4.4107821	4.0919910	-2.1721126
H	4.6756609	3.1608254	-3.6669927
C	0.4295828	4.3299482	-0.3621417
C	0.4291846	2.1936959	-1.7205784
C	2.3916348	5.1317299	0.7962564
C	4.5903772	3.8466123	0.7623011
H	6.4741117	-1.7461720	-1.6554529
H	5.2105132	-2.2629783	-2.7604915
H	6.3344429	-0.9737192	-3.2568800
C	3.3860208	-4.2982681	-0.2090489
C	2.4073492	-2.6948881	-1.9293590
C	5.0303360	-3.5819571	1.4089900
C	5.9237356	-1.2142489	1.4841038
C	1.0494648	5.2752632	0.4469868
H	-0.6164566	4.4526039	-0.6268143
C	-0.1974230	2.8433561	-2.9671556
H	1.1736695	1.4691018	-2.0704342
C	-0.6322523	1.4258314	-0.9102148
H	2.8613331	5.8775899	1.4288369
C	4.6664490	3.0117853	2.0544951
C	5.3549409	5.1680132	0.9298967
H	5.0961067	3.2761949	-0.0258056
C	4.2178405	-4.5756847	0.8749687
H	2.7579021	-5.0842711	-0.6148684
C	1.0568972	-2.1911531	-1.3863228
C	2.1944943	-3.8720534	-2.8924549
H	2.8524636	-1.8772888	-2.5088142
H	5.6737921	-3.8104415	2.2533670
C	7.3999673	-1.6475885	1.5195688
C	5.4289789	-0.8385187	2.8939212
H	5.8622822	-0.3173655	0.8566436
H	0.4864275	6.1301447	0.8092161
H	0.5517204	3.3835173	-3.5549130
H	-0.6468849	2.0752708	-3.6046480
H	-0.9839835	3.5519484	-2.6892601
H	-1.4074712	2.1074992	-0.5455725
H	-1.1114884	0.6637113	-1.5330128
H	-0.1891570	0.9318617	-0.0376956
H	4.1680524	2.0377789	1.9378239
H	5.7077725	2.8241564	2.3352162
H	4.1668027	3.5293655	2.8793985
H	5.2760491	5.7903002	0.0328782
H	4.9804950	5.7455546	1.7808990
H	6.4136088	4.9606130	1.1136205
H	4.2305359	-5.5732935	1.3035187
H	1.1839178	-1.3142914	-0.7340555
H	0.3903596	-1.9054635	-2.2063975
H	0.5674010	-2.9661248	-0.7881355
H	3.1486056	-4.2665403	-3.2558384
H	1.6457375	-4.6897561	-2.4148466
H	1.6071625	-3.5416300	-3.7547366
H	7.7629207	-1.9100506	0.5206863
H	8.0202428	-0.8337310	1.9085053
H	7.5405345	-2.5169294	2.1694837
H	4.3875903	-0.4964428	2.8744612
H	5.4799143	-1.7028509	3.5639507
H	6.0474790	-0.0401492	3.3163793

Mal : dimethyl maleate *cis*-$MeO_2CCH{=}CHCO_2Me$
18
Energy = -534.6581984465

C	-0.2211376	0.6318734	1.7429099
C	0.2211285	-0.6318790	1.7429120
H	-0.3654133	1.1485818	2.6879418
C	-0.4928528	1.4740936	0.5497386
H	0.3653927	-1.1485900	2.6879442
C	0.4928602	-1.4740975	0.5497453
O	-0.1487664	2.6400722	0.4678448
O	-1.1932634	0.8221272	-0.4020983
O	0.1488510	-2.6400992	0.4678711
O	1.1932057	-0.8220977	-0.4021242
C	-1.4415591	1.5897645	-1.6099105
C	1.4415470	-1.5897500	-1.6099075
H	-1.9851488	2.5044027	-1.3646865
H	-0.4919571	1.8414253	-2.0874953
H	-2.0386653	0.9346085	-2.2422344
H	1.9851709	-2.5043625	-1.3646666
H	0.4919667	-1.8414770	-2.0875033
H	2.0386409	-0.9345962	-2.2422469

PCO^- : anion
3
Energy = -454.8647129713

P	0.3158143	-0.3169794	2.1226406
C	-1.0562610	0.2410196	2.7912981
O	-2.0747774	0.6559622	3.2876147

PhCCH : alkyne
14
Energy = -308.5830568203

C	-3.2287774	-0.0000116	-0.0002048
C	-2.0177889	-0.0000190	0.0001321
H	-4.2953769	-0.0000370	-0.0002943
C	-0.5922429	0.0000008	0.0001146
C	0.1212098	1.2140222	0.0000499
C	0.1212274	-1.2140061	0.0001736
C	1.5131175	1.2086071	0.0001559
H	-0.4267604	2.1509229	-0.0000860
C	1.5131329	-1.2085729	-0.0000580
H	-0.4267192	-2.1509171	0.0003727
C	2.2133811	0.0000189	-0.0000294
H	2.0533259	2.1509165	0.0002578
H	2.0533441	-2.1508765	-0.0003501
H	3.2994611	0.0000138	-0.0000969

Tol : toluene C_6H_5Me
15
Energy = -271.7307458168

C	4.3371219	4.0185956	-1.6212370
C	5.7381113	4.0403000	-1.6171243
C	6.4384308	5.2471828	-1.6040139
C	5.7465829	6.4596067	-1.5970446
C	4.3507534	6.4527552	-1.6055843
C	3.6556191	5.2427931	-1.6185183

H	6.2857803	3.1003320	-1.6272814
H	7.5251732	5.2403563	-1.6033853
H	6.2898477	7.4003280	-1.5898558
H	3.8015147	7.3905235	-1.6063155
H	2.5677686	5.2471726	-1.6296624
C	3.5825012	2.7116105	-1.6043059
H	4.1474995	1.9204097	-2.1069057
H	3.3992662	2.3807694	-0.5737825
H	2.6093060	2.8087183	-2.0954467

TS1 : transition state (TS) for nucleophilic Ge attack
104
Energy = -4462.678912610

Ge	-0.2203380	-0.0870490	-0.0275595
P	-1.1056202	0.7163113	-2.1976350
C	0.1570392	1.7790151	-2.3258565
O	1.0311263	2.5537583	-2.4664693
N	-1.8301732	-1.2005287	0.4061549
C	-2.3033262	-1.2454870	1.6619959
C	-2.3730922	-2.0548347	-0.6185947
C	-2.0422175	-0.2606022	2.6187619
C	-3.1264445	-2.4334888	2.0993186
C	-3.5766364	-1.6662769	-1.2509768
C	-1.6893906	-3.2267740	-0.9950531
C	-1.4512626	0.9947323	2.4095428
H	-2.4482061	-0.4365619	3.6080132
H	-3.8427019	-2.1483141	2.8721844
H	-3.6495657	-2.9047978	1.2670015
H	-2.4444599	-3.1776037	2.5297499
C	-4.1073135	-2.5120618	-2.2291416
C	-4.2925390	-0.3737189	-0.8861687
C	-2.2596332	-4.0361215	-1.9842387
C	-0.3745343	-3.6393503	-0.3565966
C	-1.5053861	1.9768972	3.5553383
N	-0.8357875	1.3337076	1.2725000
C	-3.4628783	-3.6926665	-2.5900620
H	-5.0359058	-2.2390853	-2.7215766
C	-5.4486745	-0.6171268	0.1029542
C	-4.8175108	0.3712872	-2.1233014
H	-3.5588605	0.2711481	-0.3914042
H	-1.7499989	-4.9491460	-2.2799536
C	0.7242990	-3.8254039	-1.4165248
C	-0.5336074	-4.9174111	0.4876555
H	-0.0669886	-2.8354846	0.3191087
H	-1.4054631	3.0090879	3.2190272
H	-2.4324167	1.8595945	4.1203881
H	-0.6709572	1.7618293	4.2339954
C	-0.4056631	2.6869027	1.0262981
H	-3.8925763	-4.3357278	-3.3531187
H	-5.0986919	-1.0616415	1.0371644
H	-5.9431992	0.3307923	0.3427663
H	-6.1934363	-1.2902539	-0.3370194
H	-4.0152292	0.5624284	-2.8415560
H	-5.6085723	-0.1942662	-2.6271373
H	-5.2444030	1.3326597	-1.8202946
H	0.8573982	-2.9222736	-2.0174073
H	1.6808954	-4.0530152	-0.9351172
H	0.4798780	-4.6537164	-2.0908860
H	-1.2988464	-4.7953795	1.2602073
H	-0.8219007	-5.7657924	-0.1432486
H	0.4147175	-5.1665329	0.9774313
C	-1.3145899	3.5893725	0.4299625
C	0.9138439	3.0691574	1.3380123
C	-0.8894359	4.9034289	0.2156076
C	-2.7177913	3.1585207	0.0327446
C	1.2904058	4.3962040	1.1007078
C	1.9320934	2.0999858	1.9153709
C	0.3975201	5.3115369	0.5570847
H	-1.5728967	5.6169168	-0.2341565
C	-3.1749058	3.8043054	-1.2831785
C	-3.7449558	3.4382344	1.1460797
H	-2.6786508	2.0761048	-0.1275373
H	2.3016274	4.7103851	1.3442015
C	2.3119297	2.4713000	3.3609871
C	3.1926440	2.0392825	1.0346536
H	1.4781274	1.1041688	1.9333386
H	0.7071579	6.3381665	0.3814536
H	-2.4522307	3.6150307	-2.0817214
H	-4.1383503	3.3831351	-1.5863108
H	-3.3083953	4.8867005	-1.1786561
H	-3.5009537	2.9022526	2.0665087
H	-3.7840134	4.5094930	1.3749701
H	-4.7434045	3.1204612	0.8248822
H	1.4361409	2.4946006	4.0159093
H	3.0246656	1.7432361	3.7641782
H	2.7839018	3.4597958	3.3938467
H	2.9441352	1.8119698	-0.0039858
H	3.7284875	2.9950215	1.0558461
H	3.8732604	1.2628586	1.3996711
C	2.2404365	-1.1940845	0.1991598
C	2.9885445	-1.0910420	-0.7804391
C	1.9953296	-1.6423198	1.5610669
C	3.4321496	-0.8068171	-2.1045368
O	0.9860358	-1.4040816	2.2137480
O	3.0327735	-2.3688769	2.0144908
O	3.2482266	-1.5592107	-3.0551562
O	4.0712663	0.3854401	-2.1604847
C	2.9105794	-2.8793279	3.3975937
C	3.3091331	-1.7754714	4.3669129
C	3.8063193	-4.1040322	3.4536288
H	1.8603074	-3.1478945	3.5404719
H	2.6395295	-0.9174218	4.2698681
H	3.2448501	-2.1492209	5.3942659
H	4.3383404	-1.4543071	4.1771854
H	3.4924958	-4.8487657	2.7165617
H	4.8477835	-3.8310590	3.2566133
H	3.7468216	-4.5536773	4.4499581
C	4.5426079	0.8137714	-3.4911336
C	5.8981949	0.1761402	-3.7650870
C	4.5753349	2.3318614	-3.4384915
H	3.8044627	0.4611178	-4.2167387
H	5.8197744	-0.9140092	-3.7692503
H	6.2647964	0.5006757	-4.7450358

H	6.6236941	0.4819811	-3.0039471
H	3.5845879	2.7286357	-3.2042752
H	5.2841794	2.6748629	-2.6774542
H	4.8929294	2.7240077	-4.4103523

TS2 : TS for C..C ring-closing at PCO

104

Energy = -4462.684760573

Ge	-0.2669354	0.0763676	0.0759691
P	-0.3899554	0.9996179	-2.0498076
C	0.5600526	-0.1416533	-2.8331666
O	1.1054354	-0.8120000	-3.6201539
N	-1.8178798	-1.0696406	0.4156986
C	-2.5336590	-0.9379512	1.5426421
C	-2.1133482	-2.1130459	-0.5451027
C	-2.4127417	0.1508508	2.4160787
C	-3.5077629	-2.0235084	1.9194065
C	-3.0399717	-1.8309780	-1.5733755
C	-1.4646915	-3.3604273	-0.4536536
C	-1.7225123	1.3461332	2.2071043
H	-2.9914898	0.0896315	3.3295091
H	-4.2754320	-1.6331015	2.5892755
H	-3.9739392	-2.4776667	1.0438685
H	-2.9627260	-2.8142818	2.4475001
C	-3.3391608	-2.8489111	-2.4830966
C	-3.7150421	-0.4738151	-1.6969732
C	-1.7970115	-4.3421770	-1.3950501
C	-0.4298123	-3.6817683	0.6107276
C	-1.8425869	2.4251872	3.2510969
N	-0.9375223	1.5666609	1.1372643
C	-2.7306285	-4.0980752	-2.3942849
H	-4.0528507	-2.6585895	-3.2784028
C	-5.0833658	-0.4452784	-0.9897438
C	-3.8604787	-0.0279672	-3.1591298
H	-3.0667639	0.2527483	-1.1963616
H	-1.3089131	-5.3111212	-1.3397144
C	0.9108956	-4.0923903	-0.0267906
C	-0.9145096	-4.7976443	1.5567135
H	-0.2722233	-2.7813591	1.2102107
H	-1.8403371	3.4248486	2.8137197
H	-2.7425663	2.2856217	3.8518703
H	-0.9711031	2.3555950	3.9138449
C	-0.5228442	2.9041954	0.7794285
H	-2.9752953	-4.8745051	-3.1137265
H	-4.9904869	-0.6416753	0.0815852
H	-5.5498352	0.5384842	-1.1132351
H	-5.7543609	-1.1982836	-1.4181637
H	-2.8951646	-0.0557192	-3.6721573
H	-4.5669265	-0.6611032	-3.7064565
H	-4.2410193	0.9972342	-3.1977396
H	1.2669825	-3.3569082	-0.7506313
H	1.6762641	-4.1998476	0.7492603
H	0.8122361	-5.0565263	-0.5385163
H	-1.8661674	-4.5474956	2.0348524
H	-1.0514495	-5.7361075	1.0080834
H	-0.1706736	-4.9712825	2.3422060
C	-1.4160542	3.6943094	0.0204714
C	0.7512720	3.3729420	1.1475877
C	-1.0192825	4.9915959	-0.3146330
C	-2.7706593	3.1659879	-0.4299482
C	1.1033149	4.6767847	0.7788056
C	1.7464991	2.5196294	1.9126455
C	0.2263387	5.4853744	0.0659549
H	-1.6896078	5.6215435	-0.8911376
C	-3.1196085	3.5998267	-1.8618738
C	-3.9009794	3.5786321	0.5326800
H	-2.7121592	2.0718409	-0.4172437
H	2.0819920	5.0582025	1.0567294
C	2.0770946	3.1340242	3.2853932
C	3.0328683	2.3096725	1.0924463
H	1.2854577	1.5437081	2.0897645
H	0.5164748	6.4960107	-0.2075354
H	-2.3239977	3.3318711	-2.5630398
H	-4.0429660	3.1065999	-2.1813868
H	-3.2876914	4.6801035	-1.9244286
H	-3.7433404	3.1843886	1.5391446
H	-3.9664448	4.6704939	0.5992690
H	-4.8616918	3.1989664	0.1677402
H	1.1777300	3.2647411	3.8954777
H	2.7726309	2.4871964	3.8304030
H	2.5508989	4.1149934	3.1685247
H	2.8157420	1.9214703	0.0948242
H	3.5780935	3.2542414	0.9830674
H	3.6892617	1.5927962	1.5967594
C	1.5195735	-0.7593535	0.5071657
C	2.4582469	-0.9341301	-0.3706241
C	1.5085542	-1.0691003	1.9475210
C	3.1881582	-1.0737822	-1.5333456
O	0.5261563	-0.8199985	2.6524814
O	2.6277659	-1.6498472	2.4020183
O	3.2193009	-2.1188837	-2.2060448
O	3.9115799	0.0607795	-1.8556236
C	2.6509469	-2.0035148	3.8306813
C	3.0601003	-0.7787101	4.6371991
C	3.6219197	-3.1664689	3.9442562
H	1.6352841	-2.3078711	4.0991673
H	2.3290955	0.0237309	4.5113558
H	3.1126514	-1.0355400	5.7006248
H	4.0443290	-0.4227032	4.3157723
H	3.2942033	-4.0061381	3.3245844
H	4.6235144	-2.8649958	3.6219070
H	3.6759227	-3.5007932	4.9854684
C	4.7834582	-0.0561762	-3.0191956
C	6.1225369	-0.6483082	-2.5929839
C	4.8960417	1.3496704	-3.5902035
H	4.2923994	-0.7247368	-3.7322628
H	5.9754947	-1.6458069	-2.1705187
H	6.7923031	-0.7313745	-3.4567665
H	6.5995155	-0.0094408	-1.8414589
H	3.9079510	1.7326798	-3.8621027
H	5.3428970	2.0289288	-2.8559300
H	5.5275023	1.3410446	-4.4853321

TS30 : TS for **1** as Ge/C FLP addition of PhCCH

90

Energy = -4080.473841900

Ge	-0.2123642	0.4807811	0.5606382
P	-0.4037111	1.0754902	-1.7027480
N	-0.0353516	-1.5150364	0.8311078
N	-2.2046856	0.3673220	0.9609701
C	1.1444564	0.6432877	-2.1599946
C	-0.8528695	-2.1696419	1.6694921
C	1.0459404	-2.1898205	0.1495029
C	-2.6679247	-0.4436957	1.9154091
C	-2.9904397	1.4350510	0.4179369
O	2.1256150	0.3363630	-2.7317273
C	-1.9828983	-1.6085460	2.2856219
C	-0.5881791	-3.6201841	2.0048124
C	0.8258379	-2.6215895	-1.1793268
C	2.2914742	-2.3831531	0.7894350
C	-3.9527360	-0.1059004	2.6300114
C	-3.7915791	1.1551088	-0.7130611
C	-2.8886097	2.7358511	0.9484768
H	-2.4665051	-2.2313093	3.0298578
H	-1.5061470	-4.2019138	1.8871727
H	0.1955725	-4.0534732	1.3846818
H	-0.2895881	-3.6941234	3.0558788
C	1.8887369	-3.2131718	-1.8669887
C	-0.5442795	-2.5158418	-1.8332183
C	3.3168873	-2.9915852	0.0549383
C	2.5578329	-2.0370206	2.2488886
H	-4.6605206	0.4022035	1.9722273
H	-4.4143193	-1.0038759	3.0451647
H	-3.7294684	0.5768772	3.4596115
C	-4.5314674	2.2011173	-1.2688977
C	-3.8469965	-0.2445971	-1.3069116
C	-3.6380951	3.7532247	0.3447726
C	-1.9948225	3.0649108	2.1337006
C	3.1286204	-3.3929130	-1.2618236
H	1.7428859	-3.5384721	-2.8917189
C	-1.4140280	-3.7424319	-1.4950858
C	-0.4766679	-2.3288379	-3.3547991
H	-1.0370669	-1.6318604	-1.4163497
H	4.2808394	-3.1505479	0.5302954
C	3.8264213	-1.1844106	2.4377042
C	2.7160548	-3.3207699	3.0931305
H	1.7001187	-1.4714818	2.6279675
C	-4.4602251	3.4912651	-0.7452992
H	-5.1613607	2.0094772	-2.1324492
C	-3.8504540	-0.2316248	-2.8425545
C	-5.0571735	-1.0380231	-0.7778204
H	-2.9398665	-0.7636779	-0.9796484
H	-3.5736834	4.7645327	0.7375766
C	-2.7934869	3.6829583	3.2955169
C	-0.8421818	3.9932750	1.7084019
H	-1.5520760	2.1287616	2.4936115
H	3.9441011	-3.8506001	-1.8148236
H	-1.5759850	-3.8386380	-0.4191730
H	-2.3937962	-3.6520256	-1.9778722
H	-0.9389405	-4.6630157	-1.8526423
H	0.1860853	-1.5037723	-3.6271941
H	-0.1248364	-3.2370593	-3.8572781
H	-1.4761564	-2.1066434	-3.7404445
H	3.7944996	-0.2709816	1.8432743
H	3.9319228	-0.9109120	3.4936763
H	4.7194218	-1.7519955	2.1542013
H	1.8637050	-3.9954257	2.9942600
H	3.6123013	-3.8665782	2.7777370
H	2.8332127	-3.0623560	4.1515255
H	-5.0372746	4.2933677	-1.1970821
H	-2.9925196	0.3249929	-3.2307718
H	-3.7962853	-1.2582697	-3.2201360
H	-4.7670370	0.2162152	-3.2416657
H	-5.0312457	-1.1358000	0.3106727
H	-5.9938430	-0.5395939	-1.0527335
H	-5.0655637	-2.0454305	-1.2089868
H	-3.6103659	3.0260221	3.6113084
H	-2.1370362	3.8572823	4.1550099
H	-3.2290725	4.6450407	3.0045278
H	-0.2520823	3.5410716	0.9046915
H	-1.2304998	4.9528045	1.3488849
H	-0.1769654	4.1890388	2.5566802
C	2.5100313	1.3500558	0.4085792
C	1.6592617	1.1336525	1.3187413
H	1.6772068	1.1523280	2.4011293
C	3.5566098	1.6210719	-0.4686485
C	3.8200042	2.9512448	-0.8968571
C	4.3854083	0.5819867	-0.9687897
C	4.8779510	3.2185218	-1.7524037
H	3.1824423	3.7544808	-0.5402624
C	5.4364796	0.8721078	-1.8285696
H	4.1775906	-0.4420841	-0.6795749
C	5.6981953	2.1858758	-2.2260282
H	5.0653712	4.2441322	-2.0602644
H	6.0561955	0.0595648	-2.1995999
H	6.5216690	2.4040851	-2.8994621

TS3a : higher TS for **1** as Ge/C FLP to PhCCH

90

Energy = -4080.472927894

Ge	0.0054755	0.4516774	0.2149129
P	-0.7137172	1.9900445	-1.4347940
N	1.0994375	-0.9434577	-0.7307864
N	-1.6155586	-0.7495897	0.2296870
C	0.8133081	2.6074513	-1.6982014
C	0.8719086	-2.2415748	-0.4969436
C	2.1508731	-0.5020876	-1.6187068
C	-1.4616488	-2.0604508	0.4421162
C	-2.8884783	-0.1008009	0.3903946
O	1.8223538	3.1075676	-2.0295993
C	-0.2586429	-2.7320554	0.1781110
C	1.8766568	-3.2741587	-0.9489533
C	1.8374391	-0.3429549	-2.9888375
C	3.4320997	-0.1999625	-1.1123013
C	-2.6090591	-2.8603520	1.0051155
C	-3.7008179	0.0671473	-0.7536137
C	-3.2675207	0.4058757	1.6499037
H	-0.2574595	-3.7969495	0.3795784

H	1.3670927	-4.1481648	-1.3606949
H	2.5753221	-2.8749945	-1.6836522
H	2.4470687	-3.6075249	-0.0742562
C	2.8399815	0.1250851	-3.8417766
C	0.4667586	-0.7016184	-3.5421178
C	4.3976164	0.2658218	-2.0129976
C	3.8224823	-0.4105530	0.3415374
H	-3.5707882	-2.5148233	0.6213526
H	-2.4837705	-3.9222624	0.7875191
H	-2.6303104	-2.7323072	2.0939469
C	-4.9313234	0.7099859	-0.5957843
C	-3.2557428	-0.4451202	-2.1153970
C	-4.5073948	1.0486004	1.7519515
C	-2.3806187	0.2974867	2.8794156
C	4.1105134	0.4304883	-3.3620110
H	2.6217963	0.2581404	-4.8965852
C	0.4222782	-2.1626447	-4.0301944
C	0.0127945	0.2370934	-4.6680826
H	-0.2477054	-0.6005923	-2.7184809
H	5.3910130	0.5024878	-1.6430341
C	4.5041675	0.8248772	0.9532571
C	4.7627933	-1.6251830	0.4868974
H	2.9130273	-0.6144567	0.9135191
C	-5.3381557	1.1920053	0.6468859
H	-5.5777755	0.8442790	-1.4572988
C	-3.7037820	0.4674221	-3.2655435
C	-3.7286592	-1.8896202	-2.3673066
H	-2.1599605	-0.4557221	-2.1059895
H	-4.8202064	1.4441276	2.7145427
C	-3.0749327	-0.4572047	4.0275870
C	-1.9302581	1.6908522	3.3552265
H	-1.4813644	-0.2643656	2.6075179
H	4.8739935	0.8000545	-4.0408870
H	0.6562570	-2.8661922	-3.2282125
H	-0.5771083	-2.4021109	-4.4107066
H	1.1442574	-2.3184778	-4.8398249
H	0.0484645	1.2820971	-4.3487689
H	0.6324414	0.1229536	-5.5640302
H	-1.0174801	0.0000099	-4.9513154
H	3.8843463	1.7173725	0.8446304
H	4.6724358	0.6565665	2.0222045
H	5.4784661	1.0067685	0.4855477
H	4.3315758	-2.5354841	0.0636840
H	5.7116971	-1.4335252	-0.0268649
H	4.9791476	-1.8049068	1.5457938
H	-6.2976365	1.6916019	0.7478542
H	-3.3830715	1.5001815	-3.0996770
H	-3.2646227	0.1186808	-4.2054359
H	-4.7920725	0.4574748	-3.3893704
H	-3.3265485	-2.5818068	-1.6232927
H	-4.8225413	-1.9484762	-2.3347428
H	-3.3964432	-2.2266534	-3.3555617
H	-3.4084223	-1.4519832	3.7152892
H	-2.3835300	-0.5731115	4.8689632
H	-3.9525181	0.0931486	4.3843131
H	-1.3955318	2.2241987	2.5646705
H	-2.7939550	2.2941060	3.6568341
H	-1.2584749	1.5952561	4.2143840
C	1.5662944	2.6535898	1.3526535
C	1.2156139	1.4598702	1.6968931
C	1.4942247	0.6191516	2.8656890
C	1.1503636	-0.7380566	2.9338393
C	2.1410490	1.1926137	3.9780228
C	1.4340420	-1.5018734	4.0647251
H	0.6579174	-1.2181702	2.0879496
C	2.4232860	0.4325360	5.1074974
H	2.4116277	2.2426139	3.9248850
C	2.0709961	-0.9203437	5.1604015
H	1.1569712	-2.5524661	4.0856602
H	2.9187752	0.8977644	5.9556759
H	2.2907064	-1.5109735	6.0453009
H	1.2488857	3.2703166	0.5203357

TS3 : TS for PhCCH activation with Bf$_3$/**1** FLP
124
Energy = -6290.031882165

Ge	-2.4341937	0.0987020	-0.8735294
P	-2.1522413	0.3612378	1.5886326
C	-1.5916236	-1.1930561	1.7110426
O	-1.1807070	-2.2843205	1.8485552
N	-3.2861732	1.9173233	-1.1115972
C	-4.3296679	2.0462417	-1.9428587
C	-2.5692315	3.0788338	-0.6501939
C	-5.1629479	0.9781412	-2.3022664
C	-4.6546915	3.3937301	-2.5464209
C	-2.9751558	3.6929078	0.5552101
C	-1.4576474	3.5458495	-1.3774149
C	-5.2389462	-0.2797187	-1.6827650
H	-5.9504617	1.2071177	-3.0119489
H	-5.7335601	3.5663257	-2.5425646
H	-4.1528387	4.2109994	-2.0288995
H	-4.3245695	3.3953201	-3.5923650
C	-2.2833311	4.8320759	0.9754499
C	-4.1436783	3.1527695	1.3665888
C	-0.8019957	4.6934938	-0.9163633
C	-0.9698209	2.8773074	-2.6519902
C	-6.4675789	-1.1052759	-1.9876021
N	-4.2935869	-0.7504360	-0.8653287
C	-1.2177883	5.3430478	0.2391164
H	-2.5791473	5.3269672	1.8947040
C	-5.4776689	3.7999304	0.9482146
C	-3.9279283	3.3158930	2.8781584
H	-4.2192575	2.0809787	1.1482741
H	0.0476579	5.0793853	-1.4729558
C	0.4765858	2.3942381	-2.4782609
C	-1.0857975	3.8165945	-3.8663500
H	-1.5997583	2.0011094	-2.8424298
H	-6.4877806	-2.0386682	-1.4258861
H	-7.3753986	-0.5343935	-1.7762837
H	-6.4732477	-1.3358821	-3.0592010
C	-4.5242320	-1.8899300	-0.0206914
H	-0.6926226	6.2283178	0.5849740
H	-5.7077348	3.6056616	-0.1020202
H	-6.2969403	3.3977143	1.5549549

H	-5.4427006	4.8851209	1.0959900
H	-2.9695982	2.8894408	3.1893416
H	-3.9494423	4.3697336	3.1755341
H	-4.7257475	2.8047644	3.4245057
H	0.5618378	1.7060502	-1.6337980
H	0.8346846	1.8757177	-3.3728230
H	1.1516122	3.2365456	-2.2948734
H	-2.1114667	4.1724931	-4.0020169
H	-0.4387944	4.6919355	-3.7433143
H	-0.7767828	3.2953313	-4.7789849
C	-5.3692094	-1.7638449	1.1027369
C	-3.8543027	-3.1003530	-0.3035651
C	-5.5519683	-2.8857351	1.9187097
C	-6.0793452	-0.4684576	1.4660481
C	-4.0630492	-4.1871813	0.5490463
C	-2.9719831	-3.2459172	-1.5343291
C	-4.9084246	-4.0874958	1.6501963
H	-6.2011026	-2.8077629	2.7865398
C	-5.6995127	-0.0025846	2.8821804
C	-7.6096868	-0.6181219	1.3681563
H	-5.7618561	0.3067003	0.7620197
H	-3.5563390	-5.1255323	0.3507449
C	-3.8143743	-3.6009037	-2.7742648
C	-1.8375114	-4.2602133	-1.3498643
H	-2.5029150	-2.2713554	-1.7324164
H	-5.0561548	-4.9436422	2.3023466
H	-4.6188377	0.1357261	2.9718626
H	-6.1968421	0.9463818	3.1096291
H	-6.0172644	-0.7346191	3.6322585
H	-7.9271268	-0.9380336	0.3727012
H	-7.9684243	-1.3608502	2.0891825
H	-8.0971045	0.3363336	1.5963188
H	-4.5679130	-2.8356910	-2.9767388
H	-3.1714993	-3.6907875	-3.6570289
H	-4.3298103	-4.5560900	-2.6233103
H	-1.2463976	-4.0373120	-0.4572590
H	-2.2204665	-5.2829797	-1.2625650
H	-1.1770774	-4.2324258	-2.2205542
C	0.9456334	-1.2035089	-0.9960662
C	1.0929500	-0.4852625	0.0131276
C	0.9536598	-2.0787504	-2.0852500
C	0.4565794	-1.6469219	-3.3383654
C	1.5053734	-3.3748937	-1.9538236
C	0.5060537	-2.5000359	-4.4290894
H	0.0354222	-0.6512710	-3.4176553
C	1.5837020	-4.2013844	-3.0653558
H	1.8832427	-3.6940430	-0.9908254
C	1.0842318	-3.7687515	-4.2983361
H	0.1187302	-2.1764563	-5.3901522
H	2.0261962	-5.1883727	-2.9747128
H	1.1411342	-4.4248439	-5.1616296
H	0.4338755	-0.0465846	0.7566077
B	2.8902766	-0.1415392	0.4285353
C	2.9747990	-0.8171569	1.9050125
C	3.8282396	-0.8347125	-0.7056561
C	2.8437216	1.4825712	0.4425058
C	2.5263427	-2.1148146	2.1746501
C	3.5020232	-0.1552647	3.0196567
C	3.6656787	-0.5670389	-2.0710118
C	4.8093068	-1.7942512	-0.4335747
C	3.6665971	2.3276206	-0.3115397
C	1.9383910	2.1664042	1.2687424
C	2.5210355	-2.6930081	3.4386530
C	3.5295304	-0.6983011	4.3016744
C	4.3588852	-1.2113816	-3.0868923
C	5.5320614	-2.4661357	-1.4206502
C	3.5642917	3.7174395	-0.2985366
C	1.8178927	3.5474576	1.3329863
C	3.0233195	-1.9746986	4.5186128
C	5.3015845	-2.1804071	-2.7598108
C	2.6298666	4.3351033	0.5238503
F	2.0929903	-2.9105118	1.1664728
F	4.0525042	1.0775416	2.9003013
F	2.7718784	0.3681467	-2.4726196
F	5.1314891	-2.1303331	0.8378086
F	4.6630207	1.8338231	-1.0825768
F	1.1322402	1.4717857	2.1085608
F	2.0646527	-3.9463834	3.6209341
F	4.0459392	-0.0005673	5.3314380
F	4.1246292	-0.9124512	-4.3797723
F	6.4539225	-3.3887818	-1.0841587
F	4.3765915	4.4706664	-1.0652041
F	0.9495641	4.1245008	2.1804247
F	3.0416745	-2.5158018	5.7473657
F	5.9815645	-2.8230102	-3.7236632
F	2.5236438	5.6737951	0.5490100

TS40 : TS for **1** as Ge nucleophile to Mal
94
Energy = -4306.556939977

Ge	-0.0781767	0.3851078	-0.0711745
P	-0.3069786	-1.0930653	-1.9799788
N	1.2575223	-0.5867690	1.1046731
N	-1.6301979	-0.2598990	1.0954414
C	-1.5785908	-0.2111848	-2.5743852
C	1.0104309	-0.7943558	2.4092294
C	2.5644115	-0.9415895	0.5968170
C	-1.4732460	-0.4163694	2.4140415
C	-2.9146517	-0.5170661	0.4888456
O	-2.4994763	0.3373407	-3.0555839
C	-0.2206283	-0.5503422	3.0274856
C	2.0772556	-1.4066475	3.2900961
C	2.7719802	-2.2235224	0.0502206
C	3.6191872	-0.0067748	0.7109617
C	-2.6754660	-0.4818133	3.3272972
C	-3.2090462	-1.8556712	0.1341815
C	-3.8384336	0.5213420	0.2558571
H	-0.2362608	-0.6766194	4.1035387
H	1.9188378	-1.0983082	4.3259418
H	1.9931536	-2.4978329	3.2525280
H	3.0872539	-1.1471942	2.9761280
C	4.0725521	-2.5793131	-0.3281350
C	1.6493800	-3.2328671	-0.1356598
C	4.8983096	-0.4178636	0.3272070

C	3.4052710	1.4159690	1.2160252
H	-3.5819149	-0.7766595	2.7986181
H	-2.4917113	-1.1702091	4.1547874
H	-2.8370037	0.5140047	3.7561542
C	-4.4518871	-2.1309253	-0.4399833
C	-2.2273264	-2.9857522	0.4020668
C	-5.0727603	0.1887396	-0.3173711
C	-3.5693262	1.9715044	0.6227280
C	5.1321813	-1.6946889	-0.1762099
H	4.2528356	-3.5671496	-0.7419977
C	1.6678101	-4.3338955	0.9408847
C	1.6957055	-3.8785615	-1.5318778
H	0.7004836	-2.6946563	-0.0430110
H	5.7274917	0.2765589	0.4145678
C	4.2890398	2.4186121	0.4559212
C	3.6503399	1.5675993	2.7307235
H	2.3621888	1.6763939	1.0148813
C	-5.3832955	-1.1199259	-0.6623558
H	-4.6953883	-3.1520311	-0.7171804
C	-2.1969728	-4.0253252	-0.7272345
C	-2.5216766	-3.6821057	1.7451660
H	-1.2319004	-2.5350881	0.4694495
H	-5.7992442	0.9761352	-0.4967331
C	-4.3741810	2.4025986	1.8647037
C	-3.9164018	2.9205812	-0.5415007
H	-2.5061268	2.0797288	0.8601586
H	6.1371803	-1.9908194	-0.4646321
H	1.5042848	-3.9269216	1.9415933
H	0.8783617	-5.0673487	0.7427375
H	2.6294918	-4.8590668	0.9379084
H	1.7573621	-3.1222802	-2.3194892
H	2.5582298	-4.5466790	-1.6302033
H	0.7925064	-4.4732503	-1.6988462
H	4.1500975	2.3162422	-0.6220308
H	4.0225535	3.4385795	0.7454291
H	5.3494328	2.2828356	0.6958975
H	2.9063204	1.0345662	3.3257339
H	4.6444435	1.1958862	3.0050689
H	3.5973758	2.6267890	3.0072683
H	-6.3455580	-1.3535114	-1.1095502
H	-2.0019415	-3.5486173	-1.6918663
H	-1.4040379	-4.7556399	-0.5344618
H	-3.1400766	-4.5786152	-0.7926248
H	-2.4641181	-2.9857828	2.5846126
H	-3.5253598	-4.1222200	1.7363563
H	-1.7963780	-4.4848216	1.9195602
H	-4.1528967	1.7781110	2.7326681
H	-4.1388493	3.4413689	2.1207010
H	-5.4497529	2.3335273	1.6645503
H	-3.5136191	2.5646038	-1.4946966
H	-5.0016470	3.0118334	-0.6608383
H	-3.5214868	3.9220500	-0.3393746
H	-0.4844696	2.3345948	-3.2304847
C	0.1254069	2.3721154	-2.3350894
C	1.4012804	1.7468970	-2.3591324
C	-0.4716832	2.5981200	-1.0904615
O	1.9467533	1.3027239	-1.3244725
O	1.9388003	1.5254144	-3.5896670
H	-1.5531300	2.7016747	-1.0833722
C	0.1055066	3.3552877	0.0586038
C	3.1064686	0.6711290	-3.5809337
O	-0.4025139	3.4111693	1.1715575
O	1.1978761	4.0790523	-0.2773459
H	3.3819174	0.5555322	-4.6290365
H	3.9165462	1.1370674	-3.0138015
H	2.8607852	-0.2936054	-3.1297189
C	1.6947569	4.9303762	0.7839684
H	1.9625338	4.3313491	1.6569304
H	0.9288119	5.6564671	1.0658776
H	2.5677191	5.4274586	0.3633055

TS4 : TS for **1** as Ge nucleophile to **C** MalBf$_3$
128
Energy = -6516.118126645

Ge	-2.9927308	-0.3136021	-0.2968202
P	-2.0884106	-0.5108313	1.9247527
C	-1.3060031	0.9476089	1.7831010
O	-0.7314993	1.9694224	1.7778867
N	-4.3280891	1.1852855	-0.2410258
C	-5.5638166	1.0289768	-0.7325997
C	-3.8948805	2.4518032	0.3067006
C	-6.1145115	-0.2169159	-1.0648906
C	-6.4564298	2.2278474	-0.9502186
C	-4.1530777	2.7077277	1.6732505
C	-3.2084130	3.3798361	-0.5044573
C	-5.6384308	-1.4843740	-0.7170033
H	-7.1045413	-0.1902297	-1.5055986
H	-7.4498598	2.0389732	-0.5360009
H	-6.0467884	3.1379237	-0.5141337
H	-6.5808697	2.3765828	-2.0287445
C	-3.7282985	3.9291899	2.2024361
C	-4.8957801	1.7140812	2.5542460
C	-2.8061814	4.5869963	0.0794352
C	-2.9177624	3.1418435	-1.9797606
C	-6.5601886	-2.6514081	-0.9710461
N	-4.4267356	-1.6967280	-0.1806693
C	-3.0637880	4.8648253	1.4160164
H	-3.9144694	4.1478755	3.2489942
C	-6.4030193	2.0287895	2.6156954
C	-4.3257178	1.6447536	3.9785981
H	-4.7747329	0.7259392	2.0985544
H	-2.2787488	5.3173384	-0.5264201
C	-1.4848647	3.5691967	-2.3584883
C	-3.9110581	3.8987547	-2.8842555
H	-3.0366120	2.0716150	-2.1874997
H	-6.2713269	-3.5359743	-0.4033544
H	-7.5908493	-2.3807900	-0.7316430
H	-6.5229362	-2.9005799	-2.0377218
C	-4.0161873	-2.9970068	0.2943694
H	-2.7371169	5.8059073	1.8488708
H	-6.8642158	2.0099201	1.6263231
H	-6.9148458	1.2913188	3.2440521
H	-6.5692987	3.0216873	3.0485467
H	-3.2516951	1.4429478	3.9668018

H	-4.5011571	2.5755926	4.5281945
H	-4.8196955	0.8402554	4.5327041
H	-0.7412033	3.2298149	-1.6313841
H	-1.2211719	3.1710677	-3.3435194
H	-1.4048715	4.6594982	-2.4147176
H	-4.9441401	3.5995575	-2.6991618
H	-3.8329714	4.9774785	-2.7088739
H	-3.6807532	3.7045368	-3.9372886
C	-4.1407247	-3.2759802	1.6753227
C	-3.4573788	-3.9291235	-0.6030867
C	-3.6495756	-4.4980844	2.1426819
C	-4.8447652	-2.3125563	2.6201368
C	-2.9812140	-5.1379935	-0.0807776
C	-3.4072770	-3.7044828	-2.1064416
C	-3.0632439	-5.4188769	1.2775065
H	-3.7263218	-4.7337263	3.1989085
C	-4.3002565	-2.3635272	4.0538227
C	-6.3665081	-2.5592664	2.6295220
H	-4.6811510	-1.2999438	2.2340915
H	-2.5472733	-5.8708808	-0.7556804
C	-4.2893741	-4.7347934	-2.8407140
C	-1.9727197	-3.7830936	-2.6485219
H	-3.7906046	-2.7023275	-2.3231708
H	-2.6813831	-6.3596592	1.6638240
H	-3.2155449	-2.2232997	4.0763388
H	-4.7593172	-1.5685457	4.6492097
H	-4.5380074	-3.3155031	4.5406692
H	-6.8036758	-2.4329078	1.6366638
H	-6.5887516	-3.5753087	2.9740905
H	-6.8555270	-1.8513738	3.3077882
H	-5.3118442	-4.7491626	-2.4543940
H	-4.3253441	-4.5037386	-3.9107741
H	-3.8751102	-5.7426249	-2.7262894
H	-1.3279126	-3.0444939	-2.1702404
H	-1.5458191	-4.7773863	-2.4747674
H	-1.9733062	-3.6040516	-3.7277382
H	0.6314149	0.9559552	-0.1550806
C	0.2968502	0.1114881	-0.7473244
C	1.0251071	-1.0883607	-0.5439904
C	-0.7931157	0.2818158	-1.5793760
O	2.2378853	-1.1597880	-0.1413272
O	0.3907795	-2.2349287	-0.7376980
H	-1.2253431	1.2758903	-1.5798111
C	-1.0600971	-0.3934899	-2.8830099
C	1.1596317	-3.4562692	-0.5365868
O	-2.1159816	-0.3220809	-3.4903274
O	0.0566747	-0.9859327	-3.3643744
H	0.4401011	-4.2572018	-0.6916282
H	1.9701562	-3.5002061	-1.2664184
H	1.5617969	-3.4726764	0.4767497
C	-0.0195125	-1.5784611	-4.6868245
H	-1.0588288	-1.6485116	-5.0061360
H	0.4445239	-2.5622255	-4.6104615
H	0.5511224	-0.9442497	-5.3677654
B	3.2590195	0.0174766	-0.1094463
C	4.7142591	-0.7312917	0.0341437
C	3.0441912	0.8022484	-1.5343245
C	3.0737904	0.9090186	1.2501112
C	4.8899803	-1.7316259	0.9956566
C	5.8714738	-0.4042538	-0.6750434
C	3.2313673	0.0923082	-2.7242769
C	2.5849180	2.1074317	-1.7140184
C	2.2156740	0.6327962	2.3135250
C	3.9223154	1.9991672	1.4616538
C	6.0956219	-2.3873042	1.2245568
C	7.1013792	-1.0286020	-0.4741382
C	3.0114713	0.6190791	-3.9901315
C	2.3544181	2.6809720	-2.9627160
C	2.1603451	1.3987284	3.4774581
C	3.9058169	2.7900166	2.6030190
C	7.2157353	-2.0322993	0.4803874
C	2.5680399	1.9313864	-4.1130470
C	3.0078014	2.4885853	3.6240667
F	3.8539861	-2.1029707	1.7899658
F	5.8601677	0.5654230	-1.6211621
F	3.6339206	-1.2032010	-2.6794865
F	2.2695913	2.8903644	-0.6525636
F	1.3595479	-0.4184246	2.2709427
F	4.8244194	2.3294528	0.5044955
F	6.1925533	-3.3502481	2.1641565
F	8.1805691	-0.6701900	-1.1980313
F	3.2041001	-0.1291514	-5.0967416
F	1.8998995	3.9461222	-3.0661786
F	1.2878412	1.0906682	4.4579508
F	4.7414041	3.8393213	2.7342728
F	8.3934356	-2.6474157	0.6864605
F	2.3337522	2.4610344	-5.3264862
F	2.9664888	3.2409811	4.7379487

TS50 : TS for ring-closing at PC=O
94
Energy = -4306.575470788

Ge	-0.0349631	0.1764122	-0.2528902
P	-0.9191558	-0.8331297	-2.1530655
N	1.1881447	-0.6240751	1.0413875
N	-1.6539449	-0.1547131	0.9895652
C	-0.8676168	0.6088918	-3.0821397
C	0.9055695	-0.7637434	2.3587635
C	2.5021912	-1.0789586	0.6084320
C	-1.5306646	-0.1963703	2.3150002
C	-2.9622834	-0.3558234	0.3982404
O	-1.1700343	1.1830301	-4.0745055
C	-0.2994317	-0.4122789	2.9535193
C	1.9297759	-1.4220390	3.2510177
C	2.6341104	-2.3579560	0.0424128
C	3.6228998	-0.2421962	0.8020395
C	-2.7395497	-0.0669344	3.2061690
C	-3.2961343	-1.6845704	0.0393168
C	-3.8735003	0.6984171	0.1932209
H	-0.3325371	-0.4840352	4.0333788
H	1.6101976	-1.3586087	4.2919878
H	2.0201443	-2.4786369	2.9754366
H	2.9247985	-0.9907049	3.1433282
C	3.9200171	-2.8219102	-0.2628342

C	1.4416593	-3.2500974	-0.2553375
C	4.8850887	-0.7547141	0.4916977
C	3.5174675	1.1978874	1.2871262
H	-3.6419569	-0.4576826	2.7339944
H	-2.5716895	-0.5732206	4.1586326
H	-2.8988162	0.9979032	3.4125340
C	-4.5393921	-1.9301927	-0.5437174
C	-2.3435697	-2.8365956	0.3156015
C	-5.1061935	0.3990683	-0.4043465
C	-3.6167384	2.1379434	0.6079697
C	5.0418176	-2.0383690	-0.0245413
H	4.0369963	-3.8138639	-0.6905210
C	1.3970659	-4.4680091	0.6846547
C	1.4345372	-3.6936889	-1.7277954
H	0.5344768	-2.6635056	-0.0821226
H	5.7609659	-0.1303964	0.6460957
C	4.1902097	2.1554695	0.2863402
C	4.1445597	1.4086410	2.6794827
H	2.4570379	1.4564093	1.3425576
C	-5.4417730	-0.8951077	-0.7749540
H	-4.8048945	-2.9437964	-0.8268516
C	-2.3998787	-3.9353047	-0.7540530
C	-2.5901941	-3.4388206	1.7125712
H	-1.3290845	-2.4221503	0.3181794
H	-5.8153761	1.2050259	-0.5726425
C	-4.6331988	2.6115346	1.6660257
C	-3.6884622	3.0855527	-0.6080915
H	-2.6193645	2.2028524	1.0535223
H	6.0337342	-2.4169103	-0.2560736
H	1.3239851	-4.1632676	1.7334271
H	0.5322117	-5.0990923	0.4534385
H	2.3008344	-5.0771843	0.5711221
H	1.4502237	-2.8247179	-2.3915174
H	2.3007268	-4.3247654	-1.9549042
H	0.5304922	-4.2719895	-1.9436127
H	3.7552533	2.0530793	-0.7083815
H	4.0620106	3.1915486	0.6135834
H	5.2657284	1.9581546	0.2205769
H	3.6460354	0.8249589	3.4574159
H	5.2045601	1.1305490	2.6716792
H	4.0781571	2.4652329	2.9621951
H	-6.4029796	-1.1007905	-1.2378856
H	-2.2477589	-3.5186977	-1.7541966
H	-1.6184452	-4.6780681	-0.5638944
H	-3.3593032	-4.4634341	-0.7396654
H	-2.4757985	-2.6898526	2.5003645
H	-3.6044861	-3.8487369	1.7763769
H	-1.8779483	-4.2483792	1.9077110
H	-4.6434280	1.9590652	2.5431614
H	-4.3841820	3.6265084	1.9948667
H	-5.6470107	2.6296051	1.2505323
H	-3.0797520	2.7306408	-1.4457625
H	-4.7196245	3.1682133	-0.9690719
H	-3.3479786	4.0887069	-0.3291637
H	0.0320722	3.1370576	-2.5147009
C	0.3107065	2.3367648	-1.8404932
C	1.4450625	1.5704520	-2.0555633
C	-0.3256532	2.1513923	-0.5067549
O	1.7340442	0.5910124	-1.2649331
O	2.2150173	1.7735986	-3.1473018
H	-1.4117000	2.2206247	-0.5460077
C	0.1035525	2.9785834	0.6693357
C	3.1422772	0.7028789	-3.4560271
O	-0.4871734	3.0061249	1.7400986
O	1.2201559	3.7083221	0.4336161
H	3.6606731	1.0327741	-4.3559919
H	3.8452000	0.5518677	-2.6332634
H	2.5939268	-0.2246481	-3.6406544
C	1.6485620	4.5265238	1.5488564
H	1.9277335	3.8939765	2.3951851
H	0.8437495	5.2000465	1.8511463
H	2.5070507	5.0858244	1.1793153

TS5 : TS for ring-closing at PC=O
128
Energy = -6516.128685746

Ge	-2.9344867	-0.3350947	-0.0359047
P	-1.9474367	-0.9301888	1.9063471
C	-0.7292734	0.2911794	1.6998511
O	0.0089093	1.0421035	2.2314025
N	-4.2247341	1.1395548	0.1127583
C	-5.4853067	1.0542023	-0.3183560
C	-3.6566308	2.3554742	0.6668443
C	-6.0198575	-0.0961744	-0.9169446
C	-6.4253657	2.2180859	-0.1480223
C	-3.5447526	2.4775339	2.0695536
C	-3.1317646	3.3361188	-0.2113706
C	-5.4492570	-1.3604084	-1.0237244
H	-7.0394581	-0.0075579	-1.2728316
H	-7.2288615	1.9288479	0.5374576
H	-5.9240642	3.1002651	0.2474736
H	-6.8918579	2.4578148	-1.1078249
C	-2.8320615	3.5696253	2.5751439
C	-4.2315829	1.5151298	3.0272781
C	-2.4226475	4.4021782	0.3543213
C	-3.3556458	3.3226631	-1.7220840
C	-6.2927930	-2.4454069	-1.6387968
N	-4.1989673	-1.6626559	-0.6142041
C	-2.2547504	4.5108968	1.7298289
H	-2.7168568	3.6741152	3.6488779
C	-5.6263604	2.0489057	3.4142847
C	-3.4163604	1.2467911	4.3012741
H	-4.3634765	0.5605752	2.5062878
H	-1.9933149	5.1560759	-0.2983480
C	-2.0834328	3.6529007	-2.5271567
C	-4.4448401	4.3445857	-2.1138837
H	-3.6994785	2.3254682	-2.0178837
H	-6.0026574	-3.4380545	-1.2932285
H	-7.3489718	-2.2735600	-1.4241171
H	-6.1606714	-2.4127737	-2.7263959
C	-3.7640041	-3.0398647	-0.4519931
H	-1.6810390	5.3349113	2.1432870
H	-6.2611364	2.2082817	2.5399244
H	-6.1289673	1.3379563	4.0794246

H	-5.5335218	3.0043506	3.9423162	C	-1.7180165	0.2435761	-2.6369381
H	-2.3833631	0.9807492	4.0670997	C	0.6196846	-3.0516141	-0.7604347
H	-3.4121807	2.1212511	4.9612806	O	-2.8166877	0.1014389	-3.1552697
H	-3.8668772	0.4174855	4.8563074	O	-0.5481851	0.2518853	-3.2961467
H	-1.2225626	3.0511275	-2.2245818	H	-0.1241739	-3.8090716	-0.9998857
H	-2.2667611	3.4732580	-3.5909334	H	1.4699935	-3.1015668	-1.4421512
H	-1.8070455	4.7051647	-2.4120581	H	0.9602738	-3.1452434	0.2706264
H	-5.3897012	4.1648300	-1.5988985	C	-0.5764792	0.0901613	-4.7388549
H	-4.1121675	5.3579203	-1.8644467	H	-1.6082292	0.0539413	-5.0888682
H	-4.6262395	4.3000082	-3.1931198	H	-0.0489403	-0.8357267	-4.9719070
C	-3.9025141	-3.6152966	0.8323707	H	-0.0428019	0.9446645	-5.1554275
C	-3.1939440	-3.7447358	-1.5286748	B	2.9831556	0.1328990	0.0212119
C	-3.3989202	-4.9042873	1.0273976	C	4.3085664	-0.5609763	-0.6779859
C	-4.6351181	-2.8996306	1.9607452	C	2.6102534	1.6053821	-0.5928046
C	-2.7019903	-5.0318587	-1.2747602	C	3.2659379	0.0778406	1.6349618
C	-3.1688128	-3.2142275	-2.9536522	C	4.5687670	-1.9206039	-0.4780217
C	-2.7874754	-5.6025233	-0.0107689	C	5.2914042	0.1067447	-1.4112343
H	-3.4824504	-5.3662720	2.0053120	C	2.3672743	1.7092582	-1.9665385
C	-4.1072395	-3.2623851	3.3561408	C	2.3319556	2.7648293	0.1330217
C	-6.1512113	-3.1733378	1.8909074	C	2.6003790	-0.7093773	2.5740102
H	-4.4970065	-1.8189327	1.8260072	C	4.3467689	0.7972349	2.1504751
H	-2.2555737	-5.5952149	-2.0894474	C	5.6780225	-2.5863943	-0.9906289
C	-4.0470994	-4.0957378	-3.8663472	C	6.4209268	-0.5169301	-1.9387138
C	-1.7468785	-3.1520808	-3.5344454	C	1.8778099	2.8507291	-2.5875761
H	-3.5721584	-2.1982625	-2.9530563	C	1.8334576	3.9320542	-0.4479074
H	-2.3925446	-6.5990940	0.1646679	C	2.9459950	-0.7518147	3.9243220
H	-3.0203632	-3.1577657	3.4141464	C	4.7307738	0.7849209	3.4851395
H	-4.5553937	-2.5967472	4.1005973	C	6.6157893	-1.8770099	-1.7325897
H	-4.3736057	-4.2890364	3.6290140	C	1.6027902	3.9763951	-1.8168063
H	-6.5862702	-2.8236838	0.9519712	C	4.0180212	-0.0000890	4.3868047
H	-6.3477210	-4.2472891	1.9791475	F	3.7287517	-2.6739923	0.2767887
H	-6.6619273	-2.6606941	2.7131747	F	5.2023878	1.4373003	-1.6540947
H	-5.0634046	-4.2066043	-3.4794323	F	2.5768230	0.6364548	-2.7677486
H	-4.1033098	-3.6559983	-4.8680097	F	2.4865784	2.8127233	1.4743432
H	-3.6158296	-5.0982909	-3.9615609	F	1.5553666	-1.5023044	2.2261956
H	-1.0981447	-2.5095130	-2.9373338	F	5.0792634	1.5792220	1.3169781
H	-1.2979869	-4.1506454	-3.5793662	F	5.8613333	-3.9044694	-0.7644435
H	-1.7851362	-2.7578794	-4.5548032	F	7.3271199	0.1863603	-2.6484804
H	0.2770304	1.4168660	-0.3134084	F	1.6463091	2.8787424	-3.9171524
C	-0.2118546	0.4791989	-0.5176238	F	1.5555295	5.0138656	0.3079504
C	0.5790398	-0.6699280	-0.5887603	F	2.2498013	-1.5234411	4.7857466
C	-1.5389668	0.5359044	-1.1701712	F	5.7771965	1.5189563	3.9155620
O	1.8302600	-0.8302226	-0.3291596	F	7.6982466	-2.4967510	-2.2368577
O	-0.0814431	-1.7907929	-0.9308688	F	1.1149222	5.0921032	-2.3895147
H	-1.9045587	1.5620942	-1.0586588	F	4.3648019	-0.0314821	5.6870373

Reference

[S1] Y. Wu, L. Liu, J. Su, J. Zhu, Z. Ji, Y. Zhao, *Organometallics* **2016**, *35*, 1593-1596.

[S2] H. Hazarika, K. Neog, A. Sharma, B. Das, P. Gogoi, *The Journal of Organic Chemistry* **2019**, *84*, 5846-5854.

[S3] O. V. Dolomanov, L. J. Bourhis, R. J. Gildea, J. A. K. Howard, H. Puschmann, *Journal of Applied Crystallography* **2009**, *42*, 339-341.

[S4] G. Sheldrick, *Acta Crystallographica Section A* **2015**, *71*, 3-8.

[S5] G. Sheldrick, *Acta Crystallographica Section A* **2008**, *64*, 112-122.
